# A qualitative analysis of diagnostic testing, antibiotic selection, and quality improvement interventions for uncomplicated urinary tract infections

**DOI:** 10.1371/journal.pone.0238453

**Published:** 2020-09-02

**Authors:** Mark Pinkerton, Jahnavi Bongu, Aimee James, Jerry Lowder, Michael Durkin

**Affiliations:** 1 Division of Hospital Medicine, Department of Internal Medicine, Washington University in St. Louis, St. Louis, Missouri, United States of America; 2 Division of Infectious Diseases, Department of Medicine, Washington University in St. Louis, St. Louis, Missouri, United States of America; 3 Division of Public Health Sciences, Department of Surgery, Washington University in St. Louis, St. Louis, Missouri, United States of America; 4 Division of Urogynecology and Female Pelvic Reconstructive Surgery, Department of Obstetrics and Gynecology, Washington University in St. Louis, St. Louis, Missouri, United States of America; University of Maryland School of Medicine, UNITED STATES

## Abstract

**Background:**

Uncomplicated urinary tract infections (UTIs) can often be diagnosed based solely on symptoms and should be treated with a short course of narrow spectrum antibiotics. However, clinicians often order urine analyses and prescribe long courses of broad spectrum antibiotics.

**Objective:**

The objectives of our study are: 1) Understand how primary care providers and residents clinically approach UTIs and 2) to understand specific opportunities, based on provider type, to target future antibiotic stewardship interventions.

**Design and participants:**

We conducted semi-structured qualitative interviews of community primary care providers (n = 15) and internal medicine residents (n = 15) in St. Louis, Missouri from 2018–2019. A 5-point Likert scale was used to evaluate participant preferences for possible interventions. Interviews were transcribed, de-identified, and coded by two independent researchers using a combination inductive and deductive approach.

**Key results:**

Several common themes emerged. Both providers and residents ordered urine tests to “confirm” presence of urinary tract infections. Antibiotic prescription decisions were often based on historical practice and anecdotal experience rather than local susceptibility data or clinical practice guidelines. Community providers were more comfortable treating patients over the phone than residents and tended to prescribe longer courses of antibiotics. Both community providers and residents voiced frustrations with guidelines being difficult to easily incorporate due to length and extraneous information. Preferences for receiving and incorporating guidelines into practice varied. Both groups felt benchmarking would improve prescribing practices but had reservations about implementation. Community providers preferred pragmatic clinical decision support systems and nurse triage algorithms. Residents preferred order sets.

**Conclusions:**

Significant opportunities exist to optimize urinary tract infection management among residents and community providers. Multifaceted interventions that include provider education, synthesis of guidelines, and pragmatic clinical decision support systems are needed to improve antibiotic prescribing and diagnostic testing; optimal interventions to improve UTI management may vary based on provider training level.

## Introduction

Urinary tract infections (UTIs) account for over 10 million ambulatory visits and 2–3 million emergency department visits annually and are among the most common conditions for which antibiotics are prescribed in the outpatient setting [1, 2]. Approximately 80% of outpatient visits for UTIs are associated with an antibiotic prescription [3]. For all conditions according to the CDC, over 30% of antibiotic prescriptions are unnecessary and up to 50% of antibiotic use is inappropriate [4]. Inappropriate antibiotic prescriptions cause real harms, and inappropriate antibiotic use contributes to the development of and selection for resistant bacteria, leads to adverse drug events, and increases healthcare costs [5].

Inappropriate antibiotic use for UTIs is of particular concern. For example, inappropriate treatment of asymptomatic bacteriuria (ASB), which is present in up to 16.5% of healthy women can lead to negative outcomes and increase costs [6–8]. The last decade has also seen an increase in frequency of resistance to common antibiotics when treating outpatient UTIs and multidrug-resistant infections are becoming more commonplace, making these infections more difficult to treat [9]. Clinical practice guidelines by the Infectious Diseases Society of America (IDSA), which recommend short treatment courses of narrow spectrum antibiotics, are not routinely followed by clinicians [10]. Grigoryan et al. found that fluoroquinolones remained the most common antibiotic class prescribed for UTIs and the duration of prescriptions was longer than recommended [11]. Durkin et al. used a large national administrative database to show that non-guideline recommended antibiotic prescribing was common and that over 75% of prescriptions were for the wrong treatment duration [12].

The objectives of this study were to identify factors involved in the decision-making process when treating an uncomplicated UTI in primary care settings. Specifically, we aimed to characterize how prescribers define an uncomplicated UTI and assess preferred antibiotics and treatment durations. We interviewed internal medicine residents at an academic medical center and primary care providers in non-academic community clinics. In addition, we also solicited opinions on possible interventions to improve provider adherence to guidelines.

## Materials and methods

We conducted a series of qualitative, semi-structured interviews with community care providers (n = 15) and internal medicine residents (n = 15) in St. Louis, MO. Participants included categorical internal medicine residents practicing in an outpatient clinic as part of their residency curriculum as well as community primary care providers (physicians, physician assistants, and nurse practitioners) in the St. Louis area. All community providers were employees of the same health system. We recruited study participants by sending emails to the residency classes that work in the selected clinic and to physicians in the area community medical groups. This study was approved by the Institutional Review Board (IRB) of Washington University in St. Louis. IRB approval was obtained for written and verbal consent. Verbal consent was obtained when written consent wasn’t possible in the case of phone interviews. The consent form was read to the participant over the phone and verbal consent was obtained. The consent form was then scanned and emailed to the participants. Participants were compensated for their time in the form of a gift card (USD 25 for residents; USD 50 for primary care providers). Residents were interviewed between January and May 2018. Community providers were interviewed between August 2018 and February 2019. Interviews took place either in person or over the phone per participant preference and availability using an interview guide. We began the interview with a hypothetical case: “A 27-year-old non-pregnant female with no significant past medical history presents with two days of dysuria and urinary frequency. She has no allergies or medical conditions.” We invited input on how participants would define the diagnosis and additional questions they would ask the patient. Furthermore, we used probes to evaluate clinician’s views on atypical symptoms, such as foul-smelling urine. See supplementary material for interview guide and specific questions asked during the interviews.

**Fig 1:**
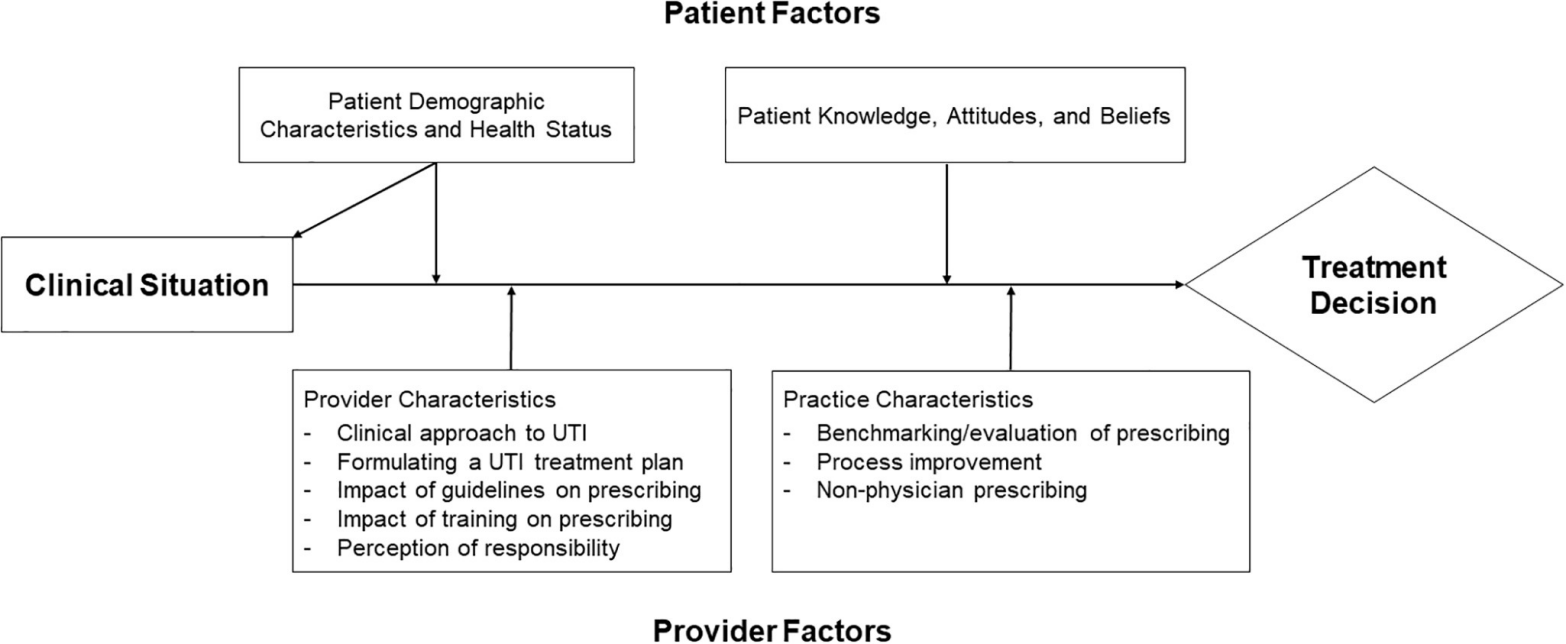
Conceptual model for prescribing decisions for UTIs.

An interview guide and initial deductive codebook were created by the primary investigator based on previous epidemiologic studies on UTI antibiotic prescribing practices and 2 pilot interviews with community providers [11, 12]. We designed the interview guide and codebook based on how patient and provider-level settings may influence antibiotic prescription behavior. This area of focus can be mapped onto the conceptual model for implementation research (CFIR) similar to that used by Zimmerman et al [13, 14]. Fig 1 outlines the conceptual model we created for prescribing decisions for UTIs. The categories of codes and themes (outlined below) are listed under the corresponding factors of the model. Digital audio recordings were made of each interview, which lasted from 15–30 minutes each. These recordings were then transcribed verbatim and de-identified. Transcripts were reviewed to identify codes that were recurrent in the data. These were then used to generate a master codebook using Nvivo 12 qualitative data analysis software (QSR International, 2014) using a combination of both deductive and inductive coding. The deductive portion of the codebook was created by one author (MJD) based on literature review and reviewed by all coauthors before the coding process began. The codebook themes were then shared with two primary care attendings who were not interviewed to obtain additional feedback and was updated to include newer codes on review of transcripts. Two independent researchers (MP and JB) assigned codes to individual transcripts for analysis. Interviews were suspended on reaching thematic saturation, which we identified as a point when no newly identified codes or themes emerged from three consecutive interviews. See Fereday et al. for an example of a hybrid deductive-inductive approach and Saunders et al. for more on thematic saturation [15, 16].

Near the conclusion of each interview, potential antimicrobial stewardship interventions were explored using a 5-point Likert scale with a score of 1 indicating an intervention the participant felt would not be useful in improving prescribing practices and a score of 5 indicating an intervention that was felt to be very useful in improving prescribing practices. Interventions considered included benchmarking (reporting of prescriber’s adherence to guidelines and metrics compared to peers), electronic medical records (EMR) alerts (pop-ups that prompt prescribers to information on guidelines, appropriate antibiotic use, and warnings), EMR order sets (pre-defined order pathways meant to streamline prescribing), educational materials for providers, educational materials for patients, and displays of public commitment (ideal behavior setting by members of or an entire clinic as examples for others). We reported median and interquartile scores. Scores were compared between residents and community providers using the Mann-Whitney U test. Comparisons with a p-value of <0.05 were considered statistically significant. Statistical testing was performed using Microsoft Excel, 2016. The study was approved by the Washington University Human Research Protection Office.

Codes and themes were grouped into five broad categories and are detailed in the results section: 1) Clinical approach to UTI—discussion of the definition of an uncomplicated UTI, when to test, and test interpretation; 2) Formulating a UTI treatment plan—incorporated antibiotic selection, treatment duration, and various factors that affect treatment plans; 3) Impact of guidelines and training effect on UTI management—included topics related to provider training, opinions on current guidelines, and how guidelines should be shared with prescribers; 4) Responsibility and benchmarking—explored opinions on evaluation of prescribing practices and perception of responsibility and; 5) Process improvement and non-physician prescribing—included topics of the prescribing process and non-physician prescribing. Included quotations from individual participants are labeled (RXX) for resident and (CXX) for community provider, where XX indicates participant number.

## Results

Mean resident age was 28.2 ± 2.4 and 47% male. Mean community provider age was 47.3 ± 13.1 and 33% male. Trainees included 2 post-graduate year (PGY)-1 residents, 8 PGY-2 residents, and 5 PGY-3 residents. Community providers included 9 MDs and 6 NPs.

### 1) Clinical approach to UTI

Most residents and community providers correctly described the case as uncomplicated. We asked what symptoms they ask patients when taking a history for suspected UTI, participants most commonly mentioned dysuria, frequency, fevers, chills, abdominal and flank pain, hematuria, urinary odor, hesitancy, and urgency. They also asked about prior UTIs, vaginal discharge, nephrolithiasis, and sexual activity. When asking about complicated vs uncomplicated, participants mentioned male gender, pregnancy, altered anatomy, indwelling catheters, fevers, and systemic symptoms as complicating factors. There was no clear “most common symptom” or “complicating factor” from the community providers based on the coding, but many were in common with the residents.

Nearly all community providers and residents mentioned getting urine testing in the hypothetical case, sometimes to “confirm” the diagnosis. For those asked if they always get testing, most indicated that they try to test everyone and avoid treating without urine testing. One resident (R05) commented in response to the case, “Get a UA and probably check a urine culture. Some people just get the UA and treat the symptoms alone, but I like to have the data for treatment—feel more confident with what I am doing. But, it’s a fast test to come back and I would probably prescribe before the test came back.” Some mentioned case exceptions like a patient away on vacation or if prescribing over the phone. When interpreting urine testing as positive for UTI, residents most often mentioned positive nitrites and microscopy results. Community providers most often mentioned leukocyte esterase activity, nitrite positivity, blood, and white blood cell count.

### 2) Formulating a UTI treatment plan

Residents most often mentioned trimethoprim-sulfamethoxazole (TMP-SMX), nitrofurantoin, and ciprofloxacin in descending order of frequency. The community providers mentioned the same three agents, though in roughly equal number of instances. Less commonly discussed were fosfomycin, cephalexin, and ampicillin. Cefdinir, amoxicillin-clavulanic acid, and levofloxacin were mentioned once each.

We discussed perceived antibiotic coverage with both groups. Several residents mentioned they took into consideration whether a patient has had recurrent UTIs or if they would have risk factors for harboring a more resistant infection. One resident (R14) commented, “Cipro tends to have really good coverage. I think there is like increasing incidence of *E*. *coli* resistance to cipro…” This sentiment was echoed by some community providers, who mentioned seeing more resistance in general. No residents or community providers reported incorporating local antibiogram (antibiotic susceptibility) data into antibiotic prescribing decisions for UTIs.

When asked if a community provider (C11) used local susceptibility data, “It would be useful if it could be easily accessible. UpToDate© is super easy to access, but if it’s something within Epic that automatically comes up with a suggestion, that would be super helpful. Otherwise if it’s something that comes in our email and it’s not right in front of me, I’m not going to remember it.” Another community provider (C12) commented, “We have one, but it’s not something I look at very often… It’s definitely helpful if you’re not sure which one to give. With respect to UTIs, I don’t use it that much. You kind of have to start them on something, so I start them on traditional first line treatment and then I’ll get a culture to see if I need to change the antibiotic. I don’t know that that would be really helpful in this situation.”

We discussed with residents and community providers if possible adverse events or effects from antibiotics affect the decision to use certain agents. Both groups brought up concerns for patient allergy to antibiotics when prescribing. Both groups discussed concerns with fluoroquinolone use such as QT interval prolongation. Community providers tended to mention the black box warning and concern for tendon rupture. Both groups discussed their choice of agent depended in part on the patient’s renal function and possible concern for acute kidney injury and hyperkalemia. The community providers brought up microbial resistance in more instances than the residents both in seeing increasingly resistant strains in their practice and in trying to decrease emergence of resistance. Neither residents nor community providers voiced opinions about certain antibiotics being “stronger” or “more potent” than others.

We received a wide variety of responses when discussing treatment duration. Residents most often mentioned courses from 3–5 days with one resident mentioning a 5–7 day course. There was a wider range from community providers from a single dose to 10 days with most responses between 3 and 7 days. From one conversation with a community provider (C08): “Do you have a preferred duration that you give to patients when you’re prescribing cipro or bactrim?” “Yeah. I would say usually 5, even though I know probably 3 is probably enough, I tend to go 5. I think 5 is mostly what I’m familiar with. Certainly, I guess, if it was more complicated, it would need to be potentially longer, but I… Just, based on no good evidence, I feel like there might not be enough, so I always just used to go with 5. I’m sure that makes you quake as an infectious disease doctor. I’m sorry.” Community providers also mentioned continuing to provide long courses because patients were more familiar with historically longer treatment durations and a shorter course may be misperceived by patients as providing inferior care or trigger a long conversation.

We asked what patient factors were considered when prescribing. Gender and age were brought up most frequently. Both groups mentioned older adults may not have classic signs or symptoms of a UTI and took anatomical abnormalities, pregnancy status, sexual activity, and medical comorbidities into consideration. Multiple participants mentioned treating patients differently if they were well known to the provider and if they had a history of prior UTIs.

We discussed with residents and community providers if patient preferences influence their prescribing practices. Both groups commented that patients have specifically requested antibiotics or expected a prescription when making an appointment. One community provider mentioned having patients that would likely go to the emergency department or urgent care if those expectations were not met, which could negatively affect care metrics. Some community providers mentioned that patients may have preferences for the exact antibiotic they receive based on past experience or may call or return to clinic with questions after reading about possible side effects online. Community providers mentioned that patients believed fluoroquinolones tended to work “better” for them compared to other antibiotics. Such patient preferences were strong influencers in antibiotic selection.

### 3) Impact of guidelines and training effect on UTI management

Residents and community providers expressed frustrations with current guidelines. Both groups mentioned there can be extraneous detail and that long formats make using them cumbersome. A common sentiment was difficulty keeping up with an ever-growing number of guidelines. The residents felt this was especially true when caring for complex and underserved patients who often have long visits with multiple competing symptoms and comorbid conditions.

We asked how guidelines should be shared with primary care providers. Residents felt that guidelines should be in an easy to access and quickly readable format. Usefulness of email newsletters, phone apps, traditional lectures, and pocket cards varied among residents. Most community providers leaned toward email newsletters and were split on usefulness of handouts and posters. Both groups mentioned that clinic or hospital group leadership updates would be helpful. The residents discussed using an existing online portal to post clinic updates and new guidelines. One community provider (C01) stated “We have a big practice here and I’ve known these people and everybody is so different, so some people are really into, you know, following these guidelines and these metrics, and there’s some people that are like, ‘I’m going to do things my way, and I don’t care.’ And it’s so difficult to say what is the best way, but I do think if there were emails, educational—you know—opportunities. And as a practice, you could say you know we’re going to try and set up these guidelines to follow. It’s just difficult.”

We discussed the influence provider training has on current prescribing practices. Some residents felt that the decision to treat and agents selected were based on the prescribing habits of their attendings, which may not always be within guidelines. From a discussion with one resident (R02), “…[a] lot of times when you talk with different attendings, what they say is based more off of their own practice and not based on guidelines for until those habits become learned habits.” One resident said it was difficult to disagree with attending recommendations early in training. Residents mentioned that lectures during residency and continuing medical education on the topic for which one could earn credit following graduation could be helpful. Some community providers mentioned prescribing based on their training. One, who previously worked at a retail pharmacy care clinic, mentioned being required to follow an algorithm for UTI prescribing. That community provider found the algorithm useful to follow, but also mentioned feeling constrained by the algorithm. Another community provider (C15) discussed fatigue in keeping up with guidelines, “…And it’s based on my experience. I have been doing this for 30 years and I now remember when I came to work with this clinic and the old doctors would say, ‘my experience the last 30 years,’ and I would say, ‘Why don’t you read the literature?’ After 30 years, believe me, you would say, ‘I don’t want to read anymore,’ I’m too tired.”

### 4) Responsibility and benchmarking

We asked residents and community providers where responsibility for prescribing and keeping up to date with guidelines should fall. Both groups brought up shared responsibility between different parties. Both felt that clinic or practice leadership as well as guideline publishing societies should have partial responsibility in updating prescribers with major changes. Residents felt near universally that ultimate responsibility should be with the prescriber and with clinic attendings sharing partial responsibility. One community provider (C09) commented, “I think that if we’re worried about antibiotic resistance going into the future, and research shows that outcomes for the general population, then yes… people should be held accountable to a certain extent. Whether or not they’re doing what’'s been well documented and proven to be the right thing to do.”

We discussed benchmarking with both groups and whether it would improve prescribing practices. The residents felt that benchmarking could bring attention to practices not in keeping with prescribing guidelines, but that their clinic patient population often made prescribing within guidelines challenging. The community providers also voiced concerns over individual clinical scenarios that required guideline deviation. Both groups noted they receive many benchmarking reports already and would be uncertain of the benefit of adding another. One resident was wary about report privacy when compared to peers. Two community providers mentioned keeping a culture of patient satisfaction and surveys in mind, especially if they would be prescribing less antibiotics. Some felt it would improve quality of care and may be beneficial from an antibiotic stewardship perspective. Individuals felt benchmarking would have the highest chance for success if done from an angle of quality improvement rather than in a punitive, invasive, or condescending manner. One community provider (C06) said, “I just hear other people talk about best-in-class [peer benchmarking] and some of those other standards that primaries are held to, and it’s just… I know, they get annoyed with it. It feels like it doesn’t matter what I do, it doesn’t matter that I’m trying to do what’s best for the patient, if I don’t do it just the way someone else wants me to, it doesn’t matter and that puts a lot of stress on everyone trying to meet a benchmark instead of making [it] patient-centered.”

### 5) Process improvement and non-physician prescribing

We asked how residents and community providers receive messages from patients with complaints of urinary symptoms and if they felt comfortable treating over the phone. Residents mentioned their call center was not staffed by medical professionals, which would lead to inaccurate histories and occasionally to emergencies that were inappropriately triaged. They also mentioned many competing obligations and one resident (R10) commented, “…we get a lot of tasks and so, you know, busy rotations and things like that. It can be hard to keep up and then some of the little, small tasks get buried in with some really like important ones and people are like without insulin or you know, big things.” Improving the call system may represent an opportunity to implement a triage-based algorithm to expedite care and improve guideline adherence. Resident comfort level varied in over the phone treatment for uncomplicated UTI. The deciding factor most often was if the patient was well known and had low concern for complicated or resistant infections. Some would refer to the urgent care portion of the clinic prior to prescribing treatment.

Some community providers voiced similar concerns about inaccurate histories taken by call center staff. Others stated that they would specifically train their staff on how to assess UTI symptoms to improve the accuracy of data collection. Some community providers felt comfortable treating over the phone, with some recommending that the patient submit a urine specimen for further evaluation.

We discussed non-traditional models of prescribing including call center algorithms run by non-physicians and nurse practitioners, electronic visits, and prescription kiosks. Some reported their own clinic had implemented an algorithm for weekends and after hours without issue and potential benefits mentioned included saved time and patient convenience. Community providers noted they would be more comfortable if there was a team/practice consensus on the exact algorithm with a protocol for oversight and algorithm deviation.

For electronic visits, there were concerns raised over lack of urine testing and physical exam and with some patients being unable or unwilling to adopt an electronic format. Regarding self-prescribing kiosks, which have been pilot tested in emergency departments in California [17, 18], there were concerns over the kiosk’s ability to handle protocol deviations. From an interview with community provider (C02), “[It] may be better with the nurse. I wouldn’t like the kiosk so much because there are other questions. At least with the nurses they’ve been trained, okay if this, then we need to go off [in] this direction that’s these questions, where for a kiosk may not be able to recognize that…” Similar concerns were raised over lack of urine testing, safety concerns, and lack of oversight, but there might be potential benefits for patient access to care.

Quantitative 5-point Likert scale of participant opinions on antibiotic stewardship interventions are displayed in Table 1. Community providers tended to favor benchmarking, order sets, and displays of public commitment while residents preferred EMR order sets, though there was considerable provider to provider variability. When discussing with one community provider (C09) about order sets and pathways, “Well, I think if you can make it simple enough, to the point, then I think it’s okay. There should be some room for personalization. Let’s say there’s a specific test you wanna do, an antibiotic you want to order, attached to some ICD code, there should be some flexibility…” One resident (R15) commented, “I think order set notifications… like if I have a diagnosis code of urinary tract [infection], all these things would automatically pop up that would, you know, say all we can do these things. In general, that will ease the process of making right decisions.” When the interviewer asked, “What about materials given to patients, either handouts on urinary tract infections, posters put up in clinic…,” one resident (R03) replied, “I would love to believe it, but I think our clinic population health literacy is very poor. I probably give that one a 1.”

**Table 1:** Median 5-point Likert scale responses.

Practices assessed for improvement of adherence to guidelines	Residents (n = 15)	Providers (n = 15)	p value
Benchmarking	4 (4–4)	4 (3.25–4.75)	p = 0.60
EMR alerts	3.5 (2.5–4)	3 (2.5–4.25)	p = 0.73
EMR order sets	4.5 (4–5)	4 (3–4)	p = 0.01
Educational materials for providers	3 (2.75–3.75)	3 (2–4)	p = 0.92
Educational materials for patients	2 (1.25–3)	3 (2.75–4)	p = 0.01
Displays of public commitment	3 (2–4)	4 (3–4.75)	p = 0.06

*The above table presents median scores and interquartile range (IQR) in parentheses among residents and providers. A score of 1 on the Likert scale indicates an intervention the participant felt would not be useful in improving prescribing practices and a score of 5 indicates an intervention that was felt to be very useful in improving prescribing practices.

## Discussion

In a series of semi-structured qualitative interviews, we discovered that residents and community providers approach the management of UTIs largely from anecdotal experiences and previous training. Although those interviewed were vaguely aware that guidelines existed and could accurately speculate on the spirit of the recommendations, many in both groups were still selecting non-first line treatments, prescribing for prolonged courses, and were over-utilizing urine testing. We also identified barriers with implementing UTI clinical practice guidelines in outpatient settings. Specifically, residents and community providers found the guidelines to be impractical to quickly read and follow. Furthermore, local UTI susceptibility data, use of which is recommended in the guidelines, was not known or made easily accessible to outpatient residents or primary care providers. These observations match with studies that demonstrated poor adherence to most recent guidelines [12, 19]. Residents and community providers were generally supportive of antibiotic stewardship interventions to facilitate optimal antibiotic prescribing for UTIs.

In recent years, the US Food and Drug Administration (FDA) has continued to add warnings regarding safety concerns with fluoroquinolone use [20–22], most recently for association with aortic aneurysm and dissection [23]. The FDA specifically recommends not using this class for uncomplicated UTIs [22]. One study showed no change in fluoroquinolone prescribing for outpatient uncomplicated UTIs following the 2016 boxed warning [24], however another study demonstrated a decreased number of fluoroquinolone prescriptions in the inpatient setting for all infections [25]. In our qualitative analysis, residents and community providers seemed to be aware of these warnings, however they did not appear to have a major influence on prescribing patterns. Fluoroquinolones were still mentioned third most frequently by residents and were among the most common agents mentioned by community providers. Our results are similar to qualitative findings reported by Grigoryan et al [26]. However, in their study, providers generally selected fluoroquinolones because they were perceived to be superior agents than TMP-SMX and nitrofurantoin. In this study, resident’s and community provider’s historical practices seemed to be the main driver for antibiotic selection.

Urine testing for patients with a high pre-test probability for UTI is unnecessary with adequate patient history and even a negative test may not reliably “rule out” an infection [27]. Inappropriate testing can lead to inappropriate treatment for asymptomatic bacteriuria and increased costs [6, 7]. In our study, providers frequently wanted to obtain urine testing on patients to “confirm” the diagnosis. This may represent an over-reliance on urine testing, as many participants mentioned getting urine testing on our hypothetical case. Such practices demonstrate an opportunity for diagnostic antimicrobial stewardship.

We found that both residents and community providers were often prescribing longer treatment courses than recommended by the guidelines. Community providers mentioned in some cases prescribing longer courses of antibiotics due to patient expectation and prior experience with longer courses. It was noted that patient satisfaction scores, which can be tied to salary and bonus structure, play a role in prescribing decisions.

Although guidelines recommend that local susceptibility patterns should be used to select empiric treatment for uncomplicated UTIs [10], participants interviewed did not discuss incorporating local antibiotic susceptibility data into their decisions; many of those interviewed did not know that the data existed or how to access it. Of note, antibiogram data for urinary *E*. *coli* susceptibility is reported within our healthcare system through a password protected online portal. Better educating outpatient community providers on local susceptibility data may improve antibiotic prescribing [28].

Residents and community providers expressed frustration with the number of guidelines and in availability and formatting. Every resident and many providers mentioned UpToDate© as a major means of accessing guidelines, including those for UTIs. However, given the overall variety of responses, we suspect prescribers infrequently seek out updates on conditions that they feel comfortable managing on a day-to-day basis, including UTIs. This practice may inadvertently lead to a decreased adherence to clinical practice guidelines for common conditions.

Given considerable provider to provider variability, we suspect different intervention approaches may work better for residents compared to community providers as different providers approach diagnosis and treatment from different angles. These are often driven by historical practice and training. A combination or multifaceted approach may prove the most effective. Other interventions that have been successful in stewardship studies and reducing healthcare acquired infections, including having a “champion” [29, 30] to model and encourage behavior or using audit and feedback [31] could be considered for adaptation to the outpatient practice.

Despite community providers having mixed reactions to non-traditional prescribing models, studies have demonstrated the effectiveness of nurse telephone triage-based algorithms as an alternative to formal office visits [32, 33]. In the urgent care and emergency department setting, studies have evaluated the use of an interactive kiosk model to identify high probability cases and direct for expedited care or prescribe antibiotic treatment without provider examination [17, 18]. Although such programs can be successful, significant issues can arise when patients fall out of algorithms [34].

Our study has several limitations. We only used a straightforward case during the interview. Including a second scenario of asymptomatic bacteriuria to gauge differences in participant responses may have helped better evaluate inappropriate antibiotic treatment. We interviewed a relatively limited number of participants restricted to a specific geographical area and a single residency training program. This may have impacted our ability to detect quantitative differences in preferred antimicrobial stewardship interventions between residents and community providers. However, in both participant groups we reached qualitative thematic saturation, which we identified as no newly identified themes from three consecutive interviews. Furthermore, our findings about antibiotic selection were similar to those reported in a recently published smaller study [26]. Our qualitative approach to interviews in order to allow for variability and open-ended discussions yielded frank responses to our questions to give a real-world representation of attitudes towards antibiotic prescribing.

## Conclusions

In our study, we explored how residents and community providers clinically approach UTIs and solicited opinions on proposed practice interventions to improve guideline adherence and prescribing practices. We identified opportunities to optimize antibiotic selection, treatment duration, and urine testing practices. Residents and community providers did not report a single solution to improve care. Instead, multifaceted interventions that include provider education, synthesis of guidelines, and pragmatic clinical decisions support tools are needed to optimize antibiotic prescribing and diagnostic testing. Two particularly promising opportunities include improving the formatting of evidence-based UTI guidelines and better dissemination of local antibiotic susceptibility data.

## Supporting information

S1 File Interview guide. (DOCX)

S2 File Likert scale. (DOCX)

S3 File Coding data. (DOCX)
