# Supplementary material for: A qualitative analysis of diagnostic testing, antibiotic selection, and quality improvement interventions for uncomplicated urinary tract infections
Source: PLoS One. 2020 Sep 2;15(9):e0238453. doi: 10.1371/journal.pone.0238453 (PMC7467288; doi:10.1371/journal.pone.0238453)
Supplement: S1 File — (DOCX) [file pone.0238453.s001.docx]

Sample interview guide

**Demographics**:

What is your age?

What is your gender?

**Case:** **“I’d like to start with a hypothetical case.”**

A 27-year-old non-pregnant female with no significant past medical history presents with two days of dysuria and urinary frequency. She has no allergies or medical conditions.

**Question 1:** “Walk me through how you find out if a patient has a UTI. How do you initially find out? Do you get a task, do they come to clinic?”

**Question 2:** “What symptoms do you generally think of for a UTI? Are there certain combinations of these symptoms that make them more or less likely to have a UTI?”

**Question 3:** “What factors do you consider when deciding when and when not to treat a UTI?”

**Question 4:** "Do you use any specific resources or guidelines when picking antibiotics and duration?”

**Question 5:** “If clinical guidelines existed on how to treat uncomplicated UTI, how should they be shared with primary care providers?”

**Question 6:** If clinical guidelines for UTIs existed, do you think that providers should be evaluated by their adherence to them?

**Question 7:** Researchers who believe that adherence to clinical practice guidelines are a marker for high quality care have tried to improve provider adherence to guidelines in a variety of ways. I would like to get your thoughts about these interventions. If someone were to come to your clinic and try to improve antibiotic prescribing for uncomplicated UTIs, which interventions would you like the most? I will list a series of interventions. Please rate each one on a scale of 1 to 5 with 1 being unlikely to improve adherence, 3 being neutral, and 5 being most likely to improve adherence.

- Provider benchmarking. (Reporting of performance/adherence compared to peers)
- Electronic medical record notifications or alerts
- Electronic medical record order sets or pathways
- Educational materials for providers like handouts and posters
- Educational materials for patients like handouts and posters
- Displays of public commitment by providers stating that they will follow guidelines

**Question 8:** Some of the listed interventions can be considered intrusive by busy residents. If you were designing a study to improve adherence to best practice guidelines, which intervention or interventions would strike the best balance between improving the quality of patient care and being helpful for providers?

**Question 9:** Previous studies have demonstrated that nursing staff can successfully follow simple algorithms for UTIs – thus freeing up clinician time for more complicated decisions. What are your thoughts about this?
