# Supplementary material for: A qualitative analysis of diagnostic testing, antibiotic selection, and quality improvement interventions for uncomplicated urinary tract infections
Source: PLoS One. 2020 Sep 2;15(9):e0238453. doi: 10.1371/journal.pone.0238453 (PMC7467288; doi:10.1371/journal.pone.0238453)
Supplement: S2 File — (DOCX) [file pone.0238453.s002.docx]

|  | **Likert scale (1-5) ratings for interventions to improve adherence to guidelines.** | | | | |  |
| --- | --- | --- | --- | --- | --- | --- |
|  | **Benchmarking** | **EMR alerts** | **EMR order sets** | **Educational materials for providers** | **Educational materials for patients** | **Displays of public commitment** |
| **1** | **3** | **4** | **4 to 5** | **2** | **2** | **2** |
| **2** | **1** | **1** | **4** | **3** | **Dangerous idea** | **1** |
| **3**  **RESIDENTS** | **4** | **2** | **4 or 5** | **2** | **1** | **3** |
| **4** | **5** | **5** | **5** | **4** | **2** | **4** |
| **5** | **3** | **1** | **4** | **2 or 3** | **1 to 2** | **2** |
| **6** | **4** | **4** | **5** | **3** | **3** | **2** |
| **7** | **4** | **4** | **5** | **3** | **3** | **2** |
| **8** | **4** | **3 or 4** | **4** | **3 or 4** | **Depends on patient** | **2 or 3** |
| **9** | **4** | **5** | **4** | **3½** | **3** | **3** |
| **10** | **5** | **3** | **5** | **4** | **1** | **4** |
| **11** | **4** | **2** | **5** | **1** | **Not sure** | **4** |
| **12** | **4** | **4** | **5** | **5** | **3** | **4 or 5** |
| **13** | **4** | **3** | **4** | **3** | **2** | **3** |
| **14** | **5** | **4** | **4** | **4** |  | **4** |
| **15** | **4** | **3** | **4** | **3** | **2** | **1** |
| **1**  **COMM. PROVIDERS** | 4 | 3 | 3 | 4 (email) | 3 | 4 |
| **2** | 2 | 3 | 4 | 2 | 0 | 3 |
| **3** | 3.5 | 3 | 4 | 4 | 3.5 | 4.5 |
| **4** | 3 | 1 | 3 | 1 or 2 | 2 or 3 | 1 |
| **5** | 3 or 4 | 5 | 5 | 4 or 5 | 3 | 3 |
| **6** | 4 | 2 | 5 | 4 | 3 | 4 |
| **7** | 5 | 4 | 4 | 3 to 4 | 5 | 5 |
| **8** | 4 or 5 | 4 or 5 | 4 or 5 | 2 | 2 | 3 |
| **9** | 5 | 0 | 3 | 3 | 4 | 5 |
| **10** | 1 | 1 | 1 | 3 | 5 | 4 |
| **11** | 4 | 5 | 3 | 2 | 4 | 3 |
| **12** | 1 | 3 | 4 | 5 | 3 | 2 |
| **13** | 4 | 3 | 2 | 1 | 1 | 3 |
| **14** | 5 | 3 | 3 | 4 | 4 | 5 |
| **15** | 5 | 5 | 4 | 3 | 5 | 5 |
