# Supplementary material for: A qualitative analysis of diagnostic testing, antibiotic selection, and quality improvement interventions for uncomplicated urinary tract infections
Source: PLoS One. 2020 Sep 2;15(9):e0238453. doi: 10.1371/journal.pone.0238453 (PMC7467288; doi:10.1371/journal.pone.0238453)
Supplement: S3 File — (DOCX) [file pone.0238453.s003.docx]

Resident Codes

**CLINICAL APPROACH TO UTI**

Treat over phone

<Files\\1_11_18_002> - § 1 reference coded [0.58% Coverage]

Reference 1 - 0.58% Coverage

Would you ever consider sending an antibiotic without talking?

No

<Files\\1_11_18_003> - § 2 references coded [3.46% Coverage]

Reference 1 - 1.12% Coverage

You know I think that it is a dipstick in the clinic looked concerning I would treat I would give treatment and then you know still send off the other confirmatory test.

Reference 2 - 2.34% Coverage

I think if someone I knew and that I trusted I would still feel comfortable writing a prescription. It was someone that I wasn't sure ,was concerned that they could have other complications then would probably leave a note that says if they call again please have them provide a phone number and time where I can reach them and then try to get on that way.

<Files\\1_11_18_004> - § 1 reference coded [1.89% Coverage]

Reference 1 - 1.89% Coverage

Yes, call them back and ask more about their symptoms. If they're pretty healthy and don't have a lot of complaints I might I might go ahead and start them something empirically but ideally I would like to get like a UA and some kind of documentation before I start someone on antibiotics.

<Files\\1_18_18_006> - § 1 reference coded [1.92% Coverage]

Reference 1 - 1.92% Coverage

Look up your UTI would see if she is febrile, she had like flank pain or anything on exam, get a UA, on medical records see if she's had a UTI in the past and then if they're like resistant bugs then I might start something beyond the normal like Cipro or Bactrim or something.

<Files\\3_6_18_010> - § 1 reference coded [3.02% Coverage]

Reference 1 - 3.02% Coverage

-I guess if I knew the patient really well and kind of felt like they were reliable someone that was going to be ,someone I could keep in touch with and talk about their symptoms I would consider it ,if it was someone that had a lot of other well I guess in this situation she doesn't have a lot of other medical problems so I probably would feel more comfortable with her managing it on phone,if it was someone I knew, but if I seen them one time 2 years ago then that would be less likely to do that.

<Files\\Transcript_0001> - § 1 reference coded [3.88% Coverage]

Reference 1 - 3.88% Coverage

- do you ever treat them over the phone without seeing them or do u always require an office visit?

001- hmm , For UTI if someone and then essentially pretest probability UTI before pretest probability isprobably like 90% good to treat over phone. But never came across such

M- if pretest probabilitiy is high enough would you do any testing or just give ab’s?

001 if they have history of uti year something

When to test

<Files\\04_05_18_011> - § 4 references coded [3.36% Coverage]

References 1-2 - 0.87% Coverage

Doesn’t have any medical conditions what do you do?

11-Usually do urinalysis reflex to culture.

References 3-4 - 2.49% Coverage

is there any chance I guess once they come to the clinic and you would sort to go to the process of evaluating ordering lab studies and all those things

11-I can only think of my patient with complicated UTI. I would get a straight cath from them and urine cultures before treatment.

<Files\\04_05_18_12> - § 5 references coded [5.54% Coverage]

References 1-2 - 1.92% Coverage

Based on the things that she tells you she says it's just the dysuria and of urinary frequency ,what would you do next in terms of workup, treatments ?what would you do sort of after that?

12—I would do a UA, microscopy and probably send her home with Nitrofuarantoin.

References 3-4 - 0.83% Coverage

M—Would u always get a urinary study?

12—To be honest I would and anything I find that would change my management.

Reference 5 - 2.78% Coverage

is there ever a threshold that you would get to in terms of urinary symptoms that you would feel like urine studies would not be necessary before trt?

12--Kind of like some of the other thing like she's having dysuria, urinary frequency , hematuria, you know all those things like bladder pressure, pelvic pressure all those things you know it's pretty like a pretty straightforward to me

<Files\\04_05_18_13> - § 2 references coded [1.43% Coverage]

References 1-2 - 1.43% Coverage

would you choose to treat this case with antibiotics?

13—U have to look at urine analysis first.

M—Do u always get urine analysis?

13--Yes

<Files\\05_14_18_15> - § 4 references coded [8.32% Coverage]

References 1-2 - 1.28% Coverage

so I'll tell you that the textbook answer would be u don’t have to do any testing just treat but honestly speaking I would actually still get like a UA with microscopy but I wouldn't do the culture.

Reference 3 - 3.09% Coverage

if they if they don't have any risk factors of like your problems with developing resistance for urine tract infections or they haven't had your recurrent urinary tract infections or thing u need culture for then I can tell them to honestly speaking I wouldn't really go with the getting of urinalysis, just call them here’s like a short course for 3-5 days and just treat that. If they are high risk then get a culture so I can get some sensitivities in case they have resistance.

Reference 4 - 3.95% Coverage

what sort of risk factors do u look for?? For them to get urine studies or not..

15-- I think about is really the red flag, so that would be like the chills, nausea, the other one is like if they have any change in their urinary system, then like light-headed and dizzy when you think about low blood pressure, back pain as well that's pretty much how sick does this person look, like blood pressure like they sometimes I think should you check your blood pressure as well and if there's any kind of concern. If UTI is recurring then I would tell the patient to come in for a clinic or go to the emergency Department.

<Files\\1_11_18_002> - § 4 references coded [4.93% Coverage]

References 1-2 - 1.80% Coverage

Would you choose to get any urine studies in this case.

You said she's calling in or she's coming into clinic ?

She is in the clinic.

Would probably get some urine studies.

Which urine studies?

Just a UA

References 3-4 - 3.13% Coverage

When do you decide when not to get urine studies like UA?

If it's someone who's had frequent UTIs or drug resistance I usually tell then to get urine studies. If it's uncomplicated like never had a history of UTIs probably dont. If it sounds like they're having like fevers, chills or could be some concern for pyelo I usually have them come in for urine studies.

<Files\\1_11_18_003> - § 6 references coded [7.40% Coverage]

References 1-2 - 1.51% Coverage

So any particular next step that you would do just based on that history?

Get that dipstick you know just to look at there and then if it looks concerning you know we treat them, then get a formal UA, micro, macro and culture.

References 3-4 - 3.50% Coverage

Have you had any instances where you would want to get the urine studies before treating?

Good question and I think I'm not sure there are. Anyone I am concerned about complicated urinary tract infection or patients that have had multiple infections in the past with different organisms or if they've ever had resistance in the organisms and I think it's important to get studies .Patients that obviously if they had some sort of you know Foley or other things you know important to get cultures on it. I guess other comorbidities.

References 5-6 - 2.38% Coverage

Do you have a threshold for the symptoms when you would get a urine study or we're not like that's what would be your threshold for getting a urine study?

I don’t know that I have a threshold. I would get a study a urine study on anyone that it's different from their baseline. But you know anything that's a change and dysuria I would always get urine studies

<Files\\1_11_18_004> - § 7 references coded [15.20% Coverage]

References 1-2 - 2.18% Coverage

We know she's not pregnant so more about the symptoms if they started occurring like after sexual activity, if she's had them a lot of them in the past, and I'll probably get like a pregnancy test to confirm she's not pregnant and then a UA and a urine culture and actually see if she has a UTI before like prescribe anything for her.

References 3-4 - 4.00% Coverage

And you mentioned a couple of urine studies as well, any particular reason you would get those two certain do certain symptoms or patients or anything I guess trigger you to get urine studies vs. not getting urine studies?

Because sometimes people just have like symptoms like dysuria like cause by other reasons. I don't like to prescribe without any documented reason to prescribe. If I see some things on a UA for I even consider prescribing it. And then if I do prescribe I would like to get some culture data in case patient comes back then I can know what patient grew in the past and give ABx accordingly.

Reference 5 - 2.39% Coverage

Is there any case where you would get a UA versus not get a UA and urine studies for patients that call you?

If they if they have a means to get to the get here in to give me those samples I will get them done by like some of our patients can barely get here if they had no way to get here then maybe I'll be okay with that. But I would prefer some test before meds.

Reference 6 - 4.24% Coverage

Would you always want them to come to the clinic or just the urine studies?

I will be OK with just the urine studies like UA or culture. If they have more worrisome complaints like chills, vomiting then I want them to come in and be evaluated.

If you had a situation where someone calls leaves you a task seeing this patient complaining of dysuria and things like that and you try and call them back and can't get a hold of them. What do u do?

I will just keep calling back I mean I feel like if they're symptoms of bothersome they should be able to answer the phone or try to get back in contact with me so we can talk a little bit more about it.

Reference 7 - 2.39% Coverage

If patient comes and tell you they have UTI and has atypical symptoms like vaginal discharge, dark urine etc what would you do?

We would do a pelvic exam and will get urine studies like a urine culture before I start antibiotics. I know patient feels like they know their body but just to be a good antibiotic steward I would like to just get some objective data.

<Files\\1_11_18_005> - § 7 references coded [12.23% Coverage]

References 1-3 - 3.71% Coverage

What you think about the case what would you do?

Get a UA and probably check a urine culture. Some people just get the UA and treat the symptoms alone. But I like to have the data for treatment. Fell more confident with what I am doing. But its a fast test to come back and I would probably prescribe before the test came back.

And then for urine testing you do like a dip,micro, macro ?

So usually I do if I am in clinic then order a culture because I can't figure out how to order it another way, if I am in ER then reflex, the macro and then reflex to micro. If inpatient then order culture if that comes back positive because you know they're still there but here I usually order it upfront so it's fair not come back and do something.

References 4-5 - 3.44% Coverage

So you talk to them and they do have a UTI and when you treat him over the phone then or would you require them to come in or would you?

If they are already at home than I usually trt them over the phone. I would not ask them to come to the office to a leave a UA. Would just trt over the phone with Bactrim or look for prior culture data and use that.

Would you want a culture? Would u tell I will treat over phone but want UA/culture?

Initially nothing but symptoms same after two days them have to come in and get labs and see what's actually going on and if I don't think they have a UTI then would ask them to make an appt with urgent care or go to the ED for concerning symptoms.

Reference 6 - 1.85% Coverage

If I think they don’t have a UTI then I'll get the UA and in clinic a urine culture anyway and then will not prescribe something and tell them like I'll follow up with you by phone and prescribe something if this comes back concerning for an infection, but I usually tell him to keep a journal if its urinary frequency and urgency, stress incontinence or urge incontinence

Reference 7 - 3.22% Coverage

you'll do your best to try to educate them it sounds like that they don't have it but you also use a UA sometimes to convince yourself then that they were having UTI?

Some patients have vague symptoms and they are not good story tellers and you know their story will vary in the conversation you had with them you know within a 5 minute. Just for documentation purposes a UA for UTI vs not or even for future visits or even when they call in for team members to see if they had UTI or not or chronic complaint that they have and concern for UTIs actually pretty low and maybe I can empirically prescribe an antibiotic and less likely to be useful.

<Files\\1_18_18_006> - § 7 references coded [10.47% Coverage]

References 1-2 - 1.30% Coverage

get a UA, on medical records see if she's had a UTI in the past and then if they're like resistant bugs then I might start something beyond the normal like Cipro or Bactrim or something.

References 3-4 - 2.90% Coverage

And then u mentioned getting testing to confirm the diagnosis for most part? Do u always do that?

I usually wait to see the UA bec it comes back pretty quickly before I treat it, unless its someone I know like has recurrent UTI and with someone if they're like brand-new then like it's not they don't have other like they don't see her or like other concerning issues then I'll just wait to see UA and call in something.

Reference 5 - 1.14% Coverage

I've had people call and say oh someone else thought I might have had a urine urinary tract infection, ok so we'll just order UA and then you can come in and get it.

Reference 6 - 2.04% Coverage

I know some of the stuff is almost like protocol I think what we're allowed to order in the hospital. In The ICU I remember we couldn't order a culture unless there's more than 10 white blood cells in the in the micro so

like when I look at micro if it's less than 10 white blood cell then okay

Reference 7 - 3.09% Coverage

Do u find that useful or burdensome that someone else tell you you shouldn't get a culture on this?

I just wasn't sure what it was, like a charge nurse kept telling us that, okay. it wasn't like burdensome it was just more like well okay I guess that's where to make sense if there's not a ridiculous amount of white cells and they're probably not to like infected. But I guess I don't know if that like impression I have now is correct or not.

<Files\\1_18_18_007> - § 5 references coded [10.19% Coverage]

References 1-3 - 3.73% Coverage

When would you treat with antibiotics not treat her get testing get urine testing?

I would ask about STD exposure too and discharge, get a UA and then if I mean I would just give Bactrim too at the same time or call it in after I get the UA results.

So it was part of your routine practice to get UA on all the patients then or do you only get them on some?

With dysuria I usually get on all patients.

Do you usually get a culture with it too or just the UA alone?

A UA with reflex

References 4-5 - 6.45% Coverage

Can you walk me through in the clinic here how you find out when a patient has a UTI? Do they present to you or message or phone call or what?

You order UA and you get the results in your box should be like a couple hours later and then I think 3 days maybe 24 hours later you get initial culture results and then like then you get the final culture results later and then.

Do you always get urine testing on patients that you're worried about for UTI?

Yes mostly?

So if they call and say they have symptoms for a UTI , do u ask them to come to Clinic to be seen or just come in for to drop off a urine specimen or you sometimes if they if you just get a message only is that sufficient or do you feel like you to call him back and say hey let me clarify your symptoms and you evaluate them.?

I have rarely done phone prescriptions for UTIs.

<Files\\3_6_18_008> - § 2 references coded [2.32% Coverage]

References 1-2 - 2.32% Coverage

She has two out of the three symptoms that I usually ask for which are urinary frequency, urgency and dysuria. So I would send a UA,always do reflex to macro and a culture but I know the unit per person we look at it first. Those are my initial and then I would wait for test results to come back

<Files\\3_6_18_009> - § 4 references coded [4.79% Coverage]

References 1-2 - 1.29% Coverage

So she don’t have discharge and no new sexual partners, what would you do sort of next?

9--I think I would get a urinalysis with reflex to culture probably and then see what it showed.

References 3-4 - 3.50% Coverage

-Any sort of for getting urinary testing? Feel like u use them in some cases when you're less sure for history or do you typically urine samples on anyone with any of these symptoms.?

9--yeah I think I tend to get urinalysis and if even if ,I don't necessarily think they have an infection they might think they have an infection and it is helpful to say hey look you don't have any evidence of an infection and that's why we're treating it with antibiotics , so sometimes it is helpful in that regard.

<Files\\3_6_18_010> - § 2 references coded [0.41% Coverage]

References 1-2 - 0.41% Coverage

What would you do next?

10-In clinic? I would do UA and culture.

<Files\\5_14_18_14> - § 3 references coded [3.78% Coverage]

Reference 1 - 0.90% Coverage

Anything else u want to ask her or do anything else while she is there?

14---I would check some urine.

References 2-3 - 2.88% Coverage

M—Can u treat or not treat based on what she is telling u or would u wait for the urine studies?

14—I like to just check them but I like don't think I have wait for them to come back. I think that my primary consideration for getting a UA and your culture would be to see if there could be some kind of resistant microbe there.

<Files\\Transcript_0001> - § 3 references coded [2.78% Coverage]

References 1-2 - 1.56% Coverage

sure, so just based on that history of dysuria and urinary frequency what would you do next? Ua, urine culture ?

001-ya so especially, I would do a ua with culture

Reference 3 - 1.22% Coverage

if pretest probabilitiy is high enough would you do any testing or just give ab’s?

001 if they have history of uti year something

UTI definition

<Files\\04_05_18_011> - § 4 references coded [14.90% Coverage]

References 1-2 - 5.11% Coverage

So initially concerned for a UTI? based on the dysuria the urinary frequency…Would u define it as complicated/uncomplicated?

11—complicated, depends on what your UA shows.

M- U get ur UA back, what would make you more lean towards urinary tract infection would make you more likely to treat ?

11- on the microscopic I'll look at the esterase, nitrites, and then if both of them are positive actually be there very popular reflex microscopic, greater than 10 white blood cells, bacteria in it. If it has all four then treat. If it had three of them usually nitrates will treat as well.

Reference 3 - 5.78% Coverage

For uncomplicated cases what sort of questions do u ask? what are some of the questions you ask for in history like what sort of symptoms make you more concerned for urinary tract infection or what what symptoms would make you more likely to treat?

11—burning, frequency, usually frequency with burning without changes and other dietary habits and then how often when was the last one and usually if that they noticed that its associated with like sexual activity.

M—what if they're having atypical symptoms like vaginal discharge, dark urine…what would u do?

11- if they have vaginal discharge which is like a separate complaint then try ruling out trich etc

Reference 4 - 4.01% Coverage

Patient factors like age, any comorbidities, is anything that that goes into your your thinking from a patient perspective making more likely to treat ?

11-usually for uncomplicated, there's no like risk factor or like demographic I'm looking at that would make me unlikely to trt them.

M—gender makes a difference? a male comes in saying you're having dysuria and burning versus a female of the same age?

11-Yes, definition of a complicated UTI is a male

<Files\\04_05_18_12> - § 3 references coded [4.99% Coverage]

Reference 1 - 2.39% Coverage

I would ask her if she had any foul smelling, any cloudiness with the urine, has ever happened before, any fevers, chills, nausea, any flank pain, any blood in her urine and ask her if she sexually active, any change in consistency or smell of her discharge or anything like that

M--what kind of diagnosis are you thinking of?

12--UTI

Reference 2 - 0.92% Coverage

but one thing that I'll look for as bacteria, nitrites, RBCs and then microscopy for like white blood cells and stuff like that

Reference 3 - 1.68% Coverage

Kind of like some of the other thing like she's having dysuria, urinary frequency , hematuria, you know all those things like bladder pressure, pelvic pressure all those things you know it's pretty like a pretty straightforward to me

<Files\\04_05_18_13> - § 4 references coded [13.77% Coverage]

References 1-2 - 8.68% Coverage

I mean right off the bat you were thinking UTI but you want to make sure this is uncomplicated and not complicated, so ask if she's had any fevers or systemic symptoms, I'd ask if she's had any back or flank pain, if she has any hematuria might be suggested for nephrolithiasis. Also to make sure that there's nothing else going on I'd ask her about like her last menstrual periods to rule out concerns with pregnancy and I'd ask her about any change in her vaginal discharge, get a brief sexual history as well and I would start like this.

M—How would u define complicated and uncomplicated?

13--So I mean uncomplicated is just cystitis in someone who isn't pregnant, who is a woman who hasn't had any urinary procedures recently no evidence of pyelonephritis no evidence that there maybe like some concurrent nephrolithiasis there too, no indwelling catheter too.

Reference 3 - 2.90% Coverage

What sort of symptoms do u think of when thinking about UTI?

13—Dysuria, increased frequency, like a sensation of incomplete Void despite increased frequency. Less common is gross hematuria some people have some suprapubic discomfort, fever and flank pain if it ends up being complicated.

Reference 4 - 2.19% Coverage

I mean like like the classic, you know dysuria, increased frequency, sensation of incomplete void you know if they are febrile, those would all make me more inclined to treat than someone who's like I'm having polyuria.

<Files\\05_14_18_15> - § 2 references coded [8.77% Coverage]

Reference 1 - 6.74% Coverage

what you think about is someone with your frequency you start to think about urinary tract infection, other things on the differential as well could have a sexual transmitted illnesses and then you always have to worry with a pregnancy status cuz you always have to have that as well but with urinary tract infection so then you think about cystitis versus pyelonephritis on top of that and then there's a couple of a red flag that you want to ask in general so that's like fevers ,chills ,back pain. What do you mean by dysuria, is it burning on urination or frequency? How much do you make on a regular basis or she just drinking a lot of water, things like vaginal discharge, color, odor etcetera and then what else back pain I said that kind of stuff. you also ask about like risk factors for UTI, u can ask them like do you to have sex, do you like urinate after, some of those like health maintenance kind off options in general and then it's like vital signs , if hypertensive triage them to ED going forward. That's the general gist I would say.

Reference 2 - 2.02% Coverage

--you mention uncomplicated UTI how do u specifically define uncomplicated UTI?

15—So pretty much the presence of pyelonephritis and then also pregnancy as well and then sometimes depending on how sick they are as well, they had to be admitted then that makes it automatically complicated I believe something like.

<Files\\1_11_18_002> - § 5 references coded [5.52% Coverage]

References 1-2 - 0.56% Coverage

Sounds like it's probably a UTI, for her since it's uncomplicated

References 3-4 - 3.01% Coverage

About 50% of the time, usually its pretty uncomplicated just urinary frequency and dysuria in an uncomplicated patient. If it's at all complicated or if they have fevers and chills or I think there is something else going on or if it sounds like they've been having urinary frequency and dysuria for far longer than appropriate then I make them come in

Reference 5 - 1.96% Coverage

Urinary frequency, Dysuria, foul smelling urine, not a lot of quantity but frequent urination,

concerned about pyelo, fevers ,chills, back pain, nausea, vomiting. Usually demographic wise, young females who are sexually active.

<Files\\1_11_18_003> - § 3 references coded [3.97% Coverage]

References 1-2 - 1.34% Coverage

Sounds Uncomplicated just want to make sure you know that she didn't have symptoms that suggested extension beyond the bladder like back pain, fever, nausea and vomiting concerning for pyelo or something.

Reference 3 - 2.63% Coverage

dysuria either like increased frequency, hesitancy, and hematuria. What else I can think of and then like complicated things I had mentioned abdominal pain ,back pain, fever ,chills ,nausea ,vomiting would make me think more complicated. I don't really know of any combination, anyone that has a dysuria or a combination of two would make me concerned and any of the 3 would make me more concerned.

<Files\\1_11_18_004> - § 5 references coded [5.83% Coverage]

Reference 1 - 0.53% Coverage

Maybe like some cystitis.

Uncomplicated or complicated cystitis?

Uncomplicated.

References 2-4 - 2.92% Coverage

Talking about UTIs in general what symptoms do you typically associate with UTIs?

Increased frequency, dysuria ,sometimes abdominal pain, sometimes fever, sometimes chills, they can have CVA tenderness I guess if they have like pyelo if it's complicated, maybe some nausea, malaise, old people get altered with UTI

Any combinations of those symptoms would make UTI more probable diagnosis?

Dysuria, increase frequency, and maybe like urgency.

Reference 5 - 2.37% Coverage

If patient comes and tell you they have UTI and has atypical symptoms like vaginal discharge, dark urine etc what would you do?

We would do a pelvic exam and will get urine studies like a urine culture before I start antibiotics. I know patient feels like they know their body but just to be a good antibiotic steward I would like to just get some objective data.

<Files\\1_11_18_005> - § 5 references coded [8.83% Coverage]

References 1-2 - 1.05% Coverage

27 year old woman with no medical history or anything would you consider that kind of an uncomplicated or complicated UTI ?

Uncomplicated, no urinary anatomy not pregnant no risk factors no systemic symptoms.

References 3-4 - 6.59% Coverage

What symptoms do you think of for UTI? Dysuria, urinary frequency?

Any like change in the color odor of urine, abdominal pain, pelvic pain ,bladder pain

Are there any combinations you think of are better for UTI?

Not really I feel like dysuria is probably the one that makes it stick out the most in my mind. urinary frequency most of my patients have urinary frequency do not have UTIs many UAs to prove that they do not have a UTI. Urinary urgency too do not have a UTI. Most people say yes I think it smells different It look different which is relatively meaningless. So dysuria is the most robust

So what do you do in those situations when patients tells you of having foul smelling urine telling you I think I have a UTI?

If I think they don’t have a UTI then I'll get the UA and in clinic a urine culture anyway and then will not prescribe something and tell them like I'll follow up with you by phone and prescribe something if this comes back concerning for an infection, but I usually tell him to keep a journal if its urinary frequency and urgency, stress incontinence or urge incontinence. This one patient drinks Lots of water and keeps by bed overnight and then like has to like urinate overnight because you're drinking like before going to bed. Have to talk about behavioral modifications with them.

Reference 5 - 1.18% Coverage

I don't know if it was someone who is like really elderly and high risk for complications I might be more likely to treat empirically even if I was not super convinced but I don't think I'm like more likely to treat more men versus women.

<Files\\1_18_18_006> - § 3 references coded [5.53% Coverage]

Reference 1 - 1.92% Coverage

Look up your UTI would see if she is febrile, she had like flank pain or anything on exam, get a UA, on medical records see if she's had a UTI in the past and then if they're like resistant bugs then I might start something beyond the normal like Cipro or Bactrim or something.

Reference 2 - 1.61% Coverage

Usually the ones I ask are dysuria, hematuria and then like the screening questions should be those two and then if we get into it then to be like frequency, urgency, some people complained of the flank pain and stuff like that too.

Reference 3 - 2.00% Coverage

If the patients male treat as complicated. I don’t see a whole lot of those in clinic but I know like in the hospital we just got it. In clinic so far usually it's I've had like flank pain and then trying to decide if we should like like how urgently and how aggressive me to treat them.

<Files\\1_18_18_007> - § 1 reference coded [2.61% Coverage]

Reference 1 - 2.61% Coverage

What symptoms do you usually think of for UTI you mentioned a few already?

Dysuria, foul smelling urine, frequency, suprapubic pain

Certain combinations that you think make it more or less likely to have a UTI?

I think of it very highly when I hear dysuria, less likely when I hear frequency cuz there's a bunch of other reasons for them

<Files\\3_6_18_008> - § 6 references coded [15.60% Coverage]

Reference 1 - 1.53% Coverage

she is not pregnant so we're not worried about asymptomatic urinary tract infection. She has two out of the three symptoms that I usually ask for which are urinary frequency, urgency and dysuria.

Reference 2 - 3.19% Coverage

-do the diagnosis complicated vs. uncomplicated UTI? do you take the time to go try and fit them into one of those categories to even think about that when your prescription

8- so I do but mostly based on the past medical history that I know I guess because of her age I would expect her to Fall under the uncomplicated category or sort of depends on past medical history , how much I go into additional details

References 3-4 - 2.88% Coverage

What sort of things will u look into to call it a complicated UTI?

8- usually ill ask how many times they've had urinary tract infections before , whether they had indwelling Foleys, whether they've had other prior complications, whether they've had prior treatment with for sort of unusual organisms or drug-resistant organisms. I guess in males there always complicated

Reference 5 - 4.73% Coverage

You mentioned urine analysis before ,what are some of the things that you look for on the the urine testing?

8- I look for number of squames?? If its greater than 15 ish then I'll sort of question a little bit more but if they have symptoms I'll probably treat anyway and then I look up nitrites in the ……..as well as the whites on the micro, if they haven't had a history of prior UTI and then I usually don't wait for the culture to come back but I do follow up on the culture and if the organism is not sensitive to whatever I prescribe then I'll change the antibiotic regimen if they're still symptomatic

Reference 6 - 3.27% Coverage

the basic three symptoms I'll ask about would be dysuria, urgency, frequency and then also see if they have the systemic symptoms- fever ,chills, lightheadedness or sort of symptoms where I might have to send them to the ER and get IV antibiotics versus of course a PO antibiotics. Flank pain that is probably it.It could be someone with complicated GU anatomy, pregnant women, complicated in people with indwelling Foley.

<Files\\3_6_18_009> - § 3 references coded [5.32% Coverage]

Reference 1 - 1.52% Coverage

What your sort of thoughts?

9--I've been thinking about urinary tract infections and sexually transmitted infections. Probably ask a little bit more about her sexual history and clarify some of her urinary complaints.

Reference 2 - 1.02% Coverage

I'd ask her about flank pain ,suprapubic pain, fevers and then as far as the sexual history if she's had a new sexual contacts or vaginal discharge

Reference 3 - 2.79% Coverage

U mentioned for uncomplicated there's also the notion of complicated vs. uncomplicated, anything to use to Define what a complicated is versus non complicated?

9-well of course that affects the duration and then the males by definition I think that leaves with is complicated and then the other thing and maybe if they've had like instrumentation, recent Urological procedures or something like that.

<Files\\3_6_18_010> - § 3 references coded [5.54% Coverage]

References 1-2 - 4.13% Coverage

How do u delineate complicated vs. uncomplicated urinary tract infection?

10-In inpatient setting its mostly complicated

M- Other things you think of with a complicated one like what are certain things in the patient or otherwise that make you say this is a complicated.

10-Most males if they don't have any kind of predisposing conditions. Indwelling catheters and things like that that most males I think would be considered to be complicated, believe pregnancy is a complication as well and then like a stone or like an indwelling kind of stent or something like that make me think it's complicated.

M—This case would u call it complicated or uncomplicated?

10- Uncomplicated.

Reference 3 - 1.41% Coverage

I mean I guess if they were having lower pain suprapubic pain a little different than like dysuria then that would be something I would associate with, but otherwise the other things would be more concerned things like fevers or chills

<Files\\5_14_18_14> - § 3 references coded [8.01% Coverage]

Reference 1 - 2.85% Coverage

-I mean so it's certainly could be uncomplicated UTI you know, in like the absence of like weird other you know like vaginal discharge or something else that makes me think it's more gynecologic complaint then you know, if the dysuria and urinary frequency really are urinary symptoms I would think it was a uncomplicated UTI

References 2-3 - 5.16% Coverage

So uncomplicated UTI and complicated, what do u look at to distinguish?

14---Yeah so to see if there's any urinary tract abnormalities, any previous like urologic procedures, anything where let's say that the urine is not going through a normal path, has a supra pubic catheter so typically I myself don't count males automatically as complicated but I've heard some other people count them as complicated but I think if it were you know a guy otherwise like even if he did have BPH I would still consider him uncomplicated so long as there was no like actual instrumentation or something .

<Files\\Transcript_0001> - § 4 references coded [7.16% Coverage]

References 1-2 - 1.98% Coverage

ya..saying this as a urinary tract infection will this be a complicated or an uncomplicated one?

001-it will be an uncomplicated urinary tract infection and other things will just be has she had this before? Do

References 3-4 - 5.17% Coverage

just speaking generally with UTI what symptoms do u associate with them

001- urinary freq, pain with urination, urinary incontinence with older patient, systemic symptoms

M – do u think there is a combination of those symptoms with make u treat them with abs versus not

001- hmm, well if someone just came in with like incontinence or frequency I would be more worried more than a primary issue stress incontinence. Cant think of particular algorithm. Description of problem, age, comorbidities how much infection vs different urinary tract issue

Interpretation of test

<Files\\04_05_18_011> - § 2 references coded [4.01% Coverage]

References 1-2 - 4.01% Coverage

complicated, depends on what your UA shows.

M- U get ur UA back, what would make you more lean towards urinary tract infection would make you more likely to treat ?

11- on the microscopic I'll look at the esterase, nitrites, and then if both of them are positive actually be there very popular reflex microscopic, greater than 10 white blood cells, bacteria in it. If it has all four then treat. If it had three of them usually nitrates will treat as well.

<Files\\04_05_18_12> - § 3 references coded [5.85% Coverage]

References 1-2 - 2.76% Coverage

What sort of things are you looking for on the Urinary studies?

12-- to make sure there is no swaim's like if it says greater than 20 and taking it for a grain of salt. Given the history and giving you know her presenting symptoms I'm probably going to treat but one thing that I'll look for as bacteria, nitrites, RBCs and then microscopy for like white blood cells and stuff like that

Reference 3 - 3.09% Coverage

-You know sometimes what will be an inpatient setting and we will urine studies for whatever reason and in a female she'll pop up having like some bacteria, I’m not going to treat. But if it's a male little bit of a different story just because of the anatomy ,Females have bit of a shorter urethra male have a bit of a longer urethra. I wouldn't expect to see bacteria (in femals)in the urine so then I'll be a little bit different.

<Files\\04_05_18_13> - § 4 references coded [5.85% Coverage]

References 1-2 - 1.62% Coverage

what are the some of the things that you look for on that that would make you more likely to treat?

13—Look for blood there, leuk esterase and nitrites positive.

Reference 3 - 2.25% Coverage

sometimes we treat folks for UTI inpatient that maybe don't really have symptoms and UA equivocal but they're a little bit altered to me like high white count even though there might be a lot of other explanations for that.

Reference 4 - 1.98% Coverage

Yeah those are the main resources and then if a culture is performed and there's a specific bacteria isolate, I look at dorsata??? Sometimes if it is not I know there is Barnes resistance patterns.

<Files\\05_14_18_15> - § 2 references coded [4.55% Coverage]

References 1-2 - 4.55% Coverage

U mentioned urine analysis, any specifics u r looking in them?

15—so usually what helps to promote is like Leuk esterase, white blood cells as well sometimes you can look for blood?? Just to make sure that that clears off by the end. I'm just confirmed that if there is bacteria in the microscopy then should be looking for squamous epithelial cells if its contamination if it's a good specimen overall because his lady doesn't have recurrent infections and not worry about her develop any kind of resistance that's why I wouldn't send her for a culture itself but that's pretty much mostly it and then even if let's say if UA is dirty I wouldn’t really ask her to come back and I'll just treat her with abx.

<Files\\1_11_18_004> - § 1 reference coded [1.49% Coverage]

Reference 1 - 1.49% Coverage

If I see some things on a UA for I even consider prescribing it. And then if I do prescribe I would like to get some culture data in case patient comes back then I can know what patient grew in the past and give ABx accordingly.

<Files\\1_18_18_006> - § 7 references coded [16.34% Coverage]

References 1-2 - 2.89% Coverage

I know some of the stuff is almost like protocol I think what we're allowed to order in the hospital. In The ICU I remember we couldn't order a culture unless there's more than 10 white blood cells in the in the micro so

like when I look at micro if it's less than 10 white blood cell then okay. I leant that from ICU and that became a habit after a while cuz we weren't able to even order them if they didn’t show up.

Reference 3 - 3.09% Coverage

Do u find that useful or burdensome that someone else tell you you shouldn't get a culture on this?

I just wasn't sure what it was, like a charge nurse kept telling us that, okay. it wasn't like burdensome it was just more like well okay I guess that's where to make sense if there's not a ridiculous amount of white cells and they're probably not to like infected. But I guess I don't know if that like impression I have now is correct or not.

Reference 4 - 2.35% Coverage

I think maybe having some sort of resource somewhere that gives us maybe like a more

I don't know a more definite way of interpreting UA cuz it doesn't have to be something that like flags for us or something, some attendings will be that aggressive like to two white blood cells and then other people will be like oh dosent mean anything.

Reference 5 - 2.13% Coverage

I think early on for me sometimes it's like I disagree but I don't feel like I can say anything but then I feeling especially with the interpretation. I think usually in clinic when we've actually treated it's been pretty straightforward, but there have been situations where that seemed a little aggressive.

References 6-7 - 5.88% Coverage

So when you're off on your own in a couple years and you're interpreting UA and you don't have someone telling , where are you you're going to fall on the on the Spectrum do you know or what it what criteria are going to use since it's kind of a murky area?

I think I would end up….I don’t know… somewhere in the middle. I wouldn't say I'm like super

aggressive about like treating people but wouldn’t like ignore things either.

And then culture and UA, would you do with discordant results if the UA is positive and cultures negative?

I think usually at that point we I feel like it's so at that point they're already on the antibiotics so I think I usually just let them finish it …finish the course…

And if they have a susceptibility, how well do people follow up susceptibilities?

Not well. Not sure we get another notification about that.

<Files\\3_6_18_008> - § 2 references coded [4.74% Coverage]

References 1-2 - 4.74% Coverage

You mentioned urine analysis before ,what are some of the things that you look for on the the urine testing?

8- I look for number of squames?? If its greater than 15 ish then I'll sort of question a little bit more but if they have symptoms I'll probably treat anyway and then I look up nitrites in the ……..as well as the whites on the micro, if they haven't had a history of prior UTI and then I usually don't wait for the culture to come back but I do follow up on the culture and if the organism is not sensitive to whatever I prescribe then I'll change the antibiotic regimen if they're still symptomatic

<Files\\3_6_18_009> - § 2 references coded [1.30% Coverage]

References 1-2 - 1.30% Coverage

say you get the UA what do u look for?What will lead you to treat or not one way or the other

9--White blood cells and leuk esterase, nitrites. If all abnormal then would think as a UTI.

<Files\\3_6_18_010> - § 2 references coded [1.94% Coverage]

References 1-2 - 1.94% Coverage

anything on those that you're looking to kind of help treatment plans ?

10-well just initially like getting the urinalysis and looking at the micro if there's bacteria if there's white blood cell count ,Leck esterase, nitrates that kind of thing and then if of course if there's a organism identify then that guides you

<Files\\Transcript_0001> - § 1 reference coded [3.28% Coverage]

Reference 1 - 3.28% Coverage

Earlier u mentioned macrobid, Bactrim. Anything that sways u from one antibiotic to other?

001- one thing that sways me are if the patient has prev UTI, we get culture data or susceptibilities of those patterns in the past also if someone was having recurring symptoms, treatment failure then I would want to change antibiotics, honestly allergies.

**FORMULATING A UTI TREATMENT PLAN**

Treatment Duration

<Files\\04_05_18_011> - § 2 references coded [0.71% Coverage]

References 1-2 - 0.71% Coverage

so the guidelines usually say 3 days for uncomplicated. up to date to choose abx

<Files\\04_05_18_13> - § 1 reference coded [3.68% Coverage]

Reference 1 - 3.68% Coverage

First line is Macrobid for 5 days. You know some people also do Bactrim for 3 but I think the first line is macrobid.

M—Do u think one abx works better than the others or y do u pick one over the other?

13—I usually do Macrobid first I guess that's the recommendation to prevent folks from developing for both individual patient and community to prevent resistance.

<Files\\05_14_18_15> - § 1 reference coded [0.64% Coverage]

Reference 1 - 0.64% Coverage

For uncomplicated it should say like 3 to 5 days for uncomplicated UTI so I would go with Macrobid.

<Files\\1_11_18_002> - § 2 references coded [0.44% Coverage]

References 1-2 - 0.44% Coverage

Any particular duration you would choose?

3-5 days

<Files\\1_11_18_003> - § 2 references coded [0.11% Coverage]

References 1-2 - 0.11% Coverage

How long?

5 days

<Files\\1_11_18_004> - § 2 references coded [0.25% Coverage]

References 1-2 - 0.25% Coverage

How long a course would you do?

5 days

<Files\\1_11_18_005> - § 4 references coded [2.89% Coverage]

References 1-2 - 0.31% Coverage

I usually do Bactrim like 3 days of Bactrim double strength

Reference 3 - 1.56% Coverage

I Usually use Bactrim its my first line yes. Bactrim for the short course you know so what time for them to forget and run out of forget to take her medication and things like that just like you know the ID for 3 days bec macrobid is like 5 to 7 days or something like that and I don't know I never used fosfomycin

Reference 4 - 1.02% Coverage

SO you will use the same first line agents u talked about and then treatment duration would it be the same then too if they're older they're men or would it be different?

I usually use the same duration

<Files\\Transcript_0001> - § 2 references coded [1.43% Coverage]

References 1-2 - 1.43% Coverage

Any particular duration that you go for?

001-Generally depends on the antibiotics- Bactrim is usually a three day course and …….. is a five day course

Patient preference

<Files\\04_05_18_011> - § 1 reference coded [1.27% Coverage]

Reference 1 - 1.27% Coverage

the only issue is that they always say that the patient request for antibiotics. All the notes are very clear that they want abx to be prescribed.

Patient Factor

<Files\\04_05_18_011> - § 4 references coded [9.57% Coverage]

Reference 1 - 2.20% Coverage

Usually I put women through the course of Bactrim. I will also use Macrobid, nitrofurantoin.

M-Do u think any abx is better than other for UTI? Why u go to those first?

11-Nitrofurantoin, usually used as first line and then use the other one if failed

References 2-3 - 4.01% Coverage

Patient factors like age, any comorbidities, is anything that that goes into your your thinking from a patient perspective making more likely to treat ?

11-usually for uncomplicated, there's no like risk factor or like demographic I'm looking at that would make me unlikely to trt them.

M—gender makes a difference? a male comes in saying you're having dysuria and burning versus a female of the same age?

11-Yes, definition of a complicated UTI is a male

Reference 4 - 3.36% Coverage

For men I wouldn’t take a fluoroquinolone. in case that there is if it's complicated it's more likely either ……..obstruction or other problems like bladder problems or prostate problems. For FQ penetration problems will use bactrim and then for women no I really just pick them however. whatever like whatever they were on last time or if they want to change pick something different.

<Files\\04_05_18_12> - § 3 references coded [6.02% Coverage]

Reference 1 - 1.83% Coverage

-you mentioned Nitrofurantoin…Any particular reason for it?

12--its pretty cheap and people usually don't have any problems with it. I like to reserve like other bigger guns for like resistance or like worsening disease she has like a pyelo or something.

Reference 2 - 3.07% Coverage

You know sometimes what will be an inpatient setting and we will urine studies for whatever reason and in a female she'll pop up having like some bacteria, I’m not going to treat. But if it's a male little bit of a different story just because of the anatomy ,Females have bit of a shorter urethra male have a bit of a longer urethra. I wouldn't expect to see bacteria (in femals)in the urine so then I'll be a little bit different.

Reference 3 - 1.13% Coverage

-You know that's a good question in like younger females definitely want to treat, older people not necessarily, I mean I will treat if they're symptomatic.

<Files\\04_05_18_13> - § 4 references coded [13.17% Coverage]

References 1-2 - 5.81% Coverage

any patients factors that would make you more likely or less likely to treat like age, gender, others u could think of?.

13--I mean certainly if if someone is immunosuppressed I would be more inclined to treat. I mean pregnancy even if asymptomatic hematuria??? you need to treat them. Age I mean like in the inpatient setting than the outpatient setting sometimes we treat folks for UTI inpatient that maybe don't really have symptoms and UA equivocal but they're a little bit altered to me like high white count even though there might be a lot of other explanations for that.

Reference 3 - 4.03% Coverage

allergies a big one. My recent UTI patient was on Bactrim prophylaxis for rheumatologic condition so we did not pick bactrim and then if they have UTIs before but there is a resistance patterns in the past and then obviously like you know is this a complicated or uncomplicated UTI, it's a man or a woman, more inclined to choose only like Cipro in a man yeah those are kind of big ones cuz I might have

Reference 4 - 3.34% Coverage

-Yeah I mean I don't think that's a bad idea you know there are a lot of patient factors that may not be I guess adequately assessed by whoever is looking at your you know whether or not you fit into guidelines so I could see how that it may not be like totally adequate but I think it be a good way for just like practice improvement

<Files\\05_14_18_15> - § 2 references coded [5.82% Coverage]

Reference 1 - 2.15% Coverage

When u ask guys and girls u ask specific questions so that’s that, age really not too much, age ok I would say in older people with incontinence it makes a lot different and so with younger people where more unlikely to have any of those medical problems so more likely for them to have UTI maybe I wouldn’t know of the top off my head.

Reference 2 - 3.67% Coverage

What ends up happening is that I mean it it's hard to stratify patients to be the same way and that we use more healthcare dollars and more aggressive practices individuals that you know it's not even show African American, underserved populations , Medicaid patients overall, so the question how do you stratify? How do you stratify specifically for this? Could be a lot of ????to confirm that that is the case and you can find many reasons why I can't go against the grain. In general is going to be hard to try to stratify that specially on a resident level that as well.

<Files\\1_11_18_002> - § 3 references coded [1.45% Coverage]

Reference 1 - 0.49% Coverage

I'll probably check make sure she's not sexually active.

Reference 2 - 0.57% Coverage

Usually demographic wise, young females who are sexually active.

Reference 3 - 0.39% Coverage

diabetics around on some of the newer agents.

<Files\\1_11_18_003> - § 5 references coded [8.82% Coverage]

Reference 1 - 1.62% Coverage

I think if the patient that I know that has had UTIs that have been confirmed and then you know they know the symptoms are very similar I feel I feel pretty comfortable if they call in and say I’m having the same symptoms then I would treat them

Reference 2 - 1.57% Coverage

Anyone I am concerned about complicated urinary tract infection or patients that have had multiple infections in the past with different organisms or if they've ever had resistance in the organisms and I think it's important to get studies

Reference 3 - 0.99% Coverage

Patients that obviously if they had some sort of you know Foley or other things you know important to get cultures on it. I guess other comorbidities.

Reference 4 - 1.24% Coverage

I think if someone I knew and that I trusted I would still feel comfortable writing a prescription. It was someone that I wasn't sure ,was concerned that they could have other complications

Reference 5 - 3.41% Coverage

Any particular patient factors u consider when deciding to treat vs not treat for a UTI? Age, gender, comorbidities that would sway your decision?

I don't know I think. I can't think of any. If there's someone that has frequent complaints and I'm not sure you know how much of a change it is that I would wait for confirmatory study if it's some of that frequently is complaining that they have some lower abdominal pain or that they are having to urinate more often than I just would wait for the confirmatory study.

<Files\\1_11_18_004> - § 6 references coded [6.77% Coverage]

Reference 1 - 1.52% Coverage

If they're pretty healthy and don't have a lot of complaints I might I might go ahead and start them something empirically but ideally I would like to get like a UA and some kind of documentation before I start someone on antibiotics

References 2-5 - 4.42% Coverage

What sort of age group for age range?

Older…60 and above I'll be more prone to treat them

What about gender?

Male, if men have like female can have those symptoms and it could just be like having atropic vaginal mucosa so they have multiple like etiologies for those symptoms. If male has that then it's more complicated and i will make me more worried so I'll be more likely to treat them.

Any particular patient risk factors or other things going on with the patient that would influence your decision?

Somebody who is immunocompromised, patients with anatomical anomalies, men who have sex with men they will be at an increased risk, patients with like kidney stones.

Reference 6 - 0.84% Coverage

Men more likely to be complicated so I am more prone to do Cipro and I'll do bactrim and Cipro in the lady that's not pregnant.

<Files\\1_11_18_005> - § 4 references coded [2.86% Coverage]

References 1-2 - 1.18% Coverage

I don't know if it was someone who is like really elderly and high risk for complications I might be more likely to treat empirically even if I was not super convinced but I don't think I'm like more likely to treat more men versus women.

Reference 3 - 1.08% Coverage

At least for patient population we see in our Clinic. I think for a patient populations where it may be more health literacy are you know if you were more serious like problems they might be able to process more info.

Reference 4 - 0.59% Coverage

Our patients here are overloaded with lots of other stuff going on this is pretty far down in their list of priorities.

<Files\\1_18_18_006> - § 1 reference coded [2.00% Coverage]

Reference 1 - 2.00% Coverage

If the patients male treat as complicated. I don’t see a whole lot of those in clinic but I know like in the hospital we just got it. In clinic so far usually it's I've had like flank pain and then trying to decide if we should like like how urgently and how aggressive me to treat them.

<Files\\1_18_18_007> - § 3 references coded [3.81% Coverage]

Reference 1 - 2.24% Coverage

do you routinely think about the black box warnings for other things will just get into your medical decision-making?

For ………? Not really but like the delirium the encephalopathy type of person

Would u include younger patients too?.

Not as worried in younger patients. Oh okay and then.

References 2-3 - 1.57% Coverage

Comorbidities matter, how many times they have been in the hospital and previous culture results matter and then obviously allergies and previous drugs and then some people have a very complicated anatomy…

<Files\\3_6_18_008> - § 4 references coded [7.67% Coverage]

Reference 1 - 0.33% Coverage

I guess in males there always complicated.

References 2-3 - 2.52% Coverage

Any sort of patient factors like demographics that make you more likely to treat with antibiotics? you feel like age, gender ,any specific risk factors that relate to the patient themselves that would make you more likely to treat?

8- so male, pregnant, older. I guess older was also one of the complicated UTI risk factors

Reference 4 - 4.83% Coverage

Depends on patients. so patients who are more well yeah more well-informed and able to use the information I think they would benefit from it. patients with low health literacy or who you don't just jump on the bandwagon for those prescription commercials they see on TV its Probably less useful and probably would. I bet a lot of Physicians wouldn't like it just because you know depending on depending on whether the notion is correct if if the patient misunderstands what is given to them then it takes a long time to sort of reeducate them about why you're doing what you're doing. so I guess it depends on the patent

<Files\\3_6_18_009> - § 2 references coded [3.59% Coverage]

References 1-2 - 3.59% Coverage

anything from patient stand point that would make you more likely or less likely to treat them things like things like age, gender ,comorbidities. what do you think about them?

9-- yeah I mean that I would consider to have like low Reserve , you know if their diabetes is poorly controlled, cancer and coronary disease and CABG , if they just have a bunch of stuff going on you know I always will treat someone with comorbidities. I guess males I'm more inclined to treat. But females mostly complain with these symptoms

<Files\\3_6_18_010> - § 4 references coded [10.38% Coverage]

References 1-3 - 4.87% Coverage

are there any sort of patient-specific factors that you think about when trying to treat versus not treat any sort of things about the patient themselves, age, gender or anything like that that would make you more likely or less likely to treat?

10-I think age would play a factor and like I said they're just ability to carry this plan out on their own without kind of guidance,if they have a way to get to the pharmacy and someone that's going to be like taking helping them take her antibiotics make sure they finish the course that kind of thing.

M-If you are saying age then which kind of age are you thinking?

10- in older patients I think they're probably more prone to urinary tract infections so I would be more likely to to treat them but it's also pot is a pretty common problem young people too

Reference 4 - 5.51% Coverage

a lot of our patients are resource-limited and so some of the things that we want for them to do and maybe even they want to do for themselves or not able to accomplish for a number of reasons so I think you know especially our patient population that that those things could play into the fact that they can't get to the colo, cannot get a ride home and then of course there's also situations where your judgement has to play in and so that doesn't really those benchmarking tools are like calculate for the anomaly patients who may not for whatever reason you know not be able to fit into the guidelines and not every patient does so I mean that would be a downside is that you don't get really get credit for needing to strive on the guidelines which they are not the end-all and be-all but if there's a somewhat of a wiggle room then I think it's reasonable to make sure that people are not like going rogue and

<Files\\5_14_18_14> - § 4 references coded [11.92% Coverage]

Reference 1 - 3.01% Coverage

I think it depends on the patient, if I think it's someone who is able to give me an accurate history and maybe I've seen them before then I would be more inclined to treat over the phone but if it was someone on who I had doubts of their ability to report things accurately to me or someone that I like have no relationship with probably not.

References 2-3 - 6.57% Coverage

Are there any I guess patient factors ….so you mentioned that sometimes you're more worried about poor health literacy…any other factors like location factors or demographic factors that you consider when treating or not treating people?

14—I guess situations like straight cathing themselves at home for whatever I probably be more inclined to treat. I guess if someone is very old that might make me more yeah I mean I think that would make me more likely to treat them like it was like not like a little old lady or whatever

M--what about recent hospital stays or something like that say they have had infections before they were exposed to abx etc, does that alter ur choice for abx?

14—Would see if they have previous history from previous data

Reference 4 - 2.33% Coverage

I think so so many of our patients have poor health literacy to begin with and that's not to say that we shouldn't try to change that but I don't think that giving the patient a handout would

change the way that the person that provider is evaluating them and yeah

<Files\\Transcript_0001> - § 3 references coded [4.85% Coverage]

References 1-3 - 4.85% Coverage

Patient factors age sex effect ur decision at all

001- Hmm, it helps decide whether or not needs other investigation. For eg a young guy has UTI ita very odd so further investigation is necessary to see what’s going on and likewise kidney stones or other issue. Things do matter, gender do matter, age matters…… other things going on

M- any other patient factors like age or sex

001- Hmm I think age and gender are the big ones..comorbidities…matter are if the patient is pregnant then treat wouldn’t necessarily

Antibiotic selection

<Files\\04_05_18_011> - § 3 references coded [5.55% Coverage]

References 1-2 - 2.20% Coverage

Usually I put women through the course of Bactrim. I will also use Macrobid, nitrofurantoin.

M-Do u think any abx is better than other for UTI? Why u go to those first?

11-Nitrofurantoin, usually used as first line and then use the other one if failed

Reference 3 - 3.34% Coverage

For men I wouldn’t take a fluoroquinolone. in case that there is if it's complicated it's more likely either ……..obstruction or other problems like bladder problems or prostate problems. For FQ penetration problems will use bactrim and then for women no I really just pick them however. whatever like whatever they were on last time or if they want to change pick something different.

<Files\\04_05_18_12> - § 5 references coded [4.36% Coverage]

Reference 1 - 0.55% Coverage

I would do a UA, microscopy and probably send her home with Nitrofuarantoin.

References 2-3 - 1.83% Coverage

-you mentioned Nitrofurantoin…Any particular reason for it?

12--its pretty cheap and people usually don't have any problems with it. I like to reserve like other bigger guns for like resistance or like worsening disease she has like a pyelo or something.

References 4-5 - 1.98% Coverage

what other abx would u use?

12--I've definitely used Cipro before I've used like weird things for people in the hospital if they have steps of course but Nitrofurantoin and Cipro probably like where I like to start.

M—Is one better than the other for uncomplicated UTI?

12--No

<Files\\04_05_18_13> - § 4 references coded [7.70% Coverage]

References 1-3 - 3.68% Coverage

First line is Macrobid for 5 days. You know some people also do Bactrim for 3 but I think the first line is macrobid.

M—Do u think one abx works better than the others or y do u pick one over the other?

13—I usually do Macrobid first I guess that's the recommendation to prevent folks from developing for both individual patient and community to prevent resistance.

Reference 4 - 4.03% Coverage

allergies a big one. My recent UTI patient was on Bactrim prophylaxis for rheumatologic condition so we did not pick bactrim and then if they have UTIs before but there is a resistance patterns in the past and then obviously like you know is this a complicated or uncomplicated UTI, it's a man or a woman, more inclined to choose only like Cipro in a man yeah those are kind of big ones cuz I might have

<Files\\05_14_18_15> - § 6 references coded [3.76% Coverage]

References 1-2 - 0.63% Coverage

For uncomplicated it should say like 3 to 5 days for uncomplicated UTI so I would go with Macrobid.

References 3-4 - 1.69% Coverage

say she had an issue with Macrobid in the past like right horrible nausea, any other Abx that u would pick?

15--Okay so the I know the other two things we can do are Bactrim and one which we rarely use is fosfomycin, that's just like the injection but one time.

References 5-6 - 1.43% Coverage

what about picking between the options of antibiotics, are there certain patient factors that make you pick one over the other?

15--I can say this I haven’t used fosfomycin. Between macrobid and Bactrim I don’t really know

<Files\\1_11_18_002> - § 3 references coded [4.81% Coverage]

Reference 1 - 0.38% Coverage

I probably would just prescribe Macrobid.

Reference 2 - 1.34% Coverage

patient characteristics that would I guess sway you more towards diagnosing a UTI and treating?

Yeah sure, so diabetics around on some of the newer agents.

Reference 3 - 3.08% Coverage

Do you feel like there's certain patient populations you would pick a different antibiotic for different treatment for ?

So men I would probably pick something different, pregnant women I would pick probably something different,if this is their third fourth UTI or we tried Macrobid and it didn't work there still having symptoms if I pick something different

<Files\\1_11_18_003> - § 4 references coded [4.08% Coverage]

Reference 1 - 0.25% Coverage

No allergies no meds probably macrobid

References 2-3 - 1.48% Coverage

You mentioned Macrobid earlier. Any particular reason why Macrobid versus any other antibiotics?

It’s cheap I think, fairly no real side effects, fairly short treatment duration twice a day I guess and not too bad to take.

Reference 4 - 2.34% Coverage

I like Bactrim too. I don’t know I often use Macrobid over Bactrim. I try to avoid Cipro just because you know Cipro is associated with more side effects, has an increased risk of C diff and so I don't like to prescribe this unless they have complicated UTIs with resistant organisms in the past, then then that's when I used Cipro otherwise I try to avoid

<Files\\1_11_18_004> - § 7 references coded [4.72% Coverage]

Reference 1 - 0.54% Coverage

If you choose to treat, what antibiotic would you choose?

Bactrim if no allergies

Reference 2 - 0.37% Coverage

From Up to date , it's like first line for uncomplicated.

References 3-4 - 2.12% Coverage

Any other reason for Bactrim over everything else?

Bactrim or Cipro. Of course inpatient I will use ceftriaxone. Inpatient if they have a resistant bug then tailor abx based on what they grow.

Do you feel like some abx are better than others?

Cipro covers at the usual bugs ecoli, klebsiella. I like bactrim and Cipro

Reference 5 - 0.86% Coverage

I know patient feels like they know their body but just to be a good antibiotic steward I would like to just get some objective data

References 6-7 - 0.84% Coverage

Men more likely to be complicated so I am more prone to do Cipro and I'll do bactrim and Cipro in the lady that's not pregnant.

<Files\\1_11_18_005> - § 13 references coded [10.37% Coverage]

Reference 1 - 0.30% Coverage

I usually do Bactrim like 3 days of Bactrim double strength

References 2-3 - 0.35% Coverage

First Line Macrobid, fosfomycin. Like maybe B lactams, cephalosporin.

Reference 4 - 1.39% Coverage

FQ are excessively broad and side effects like Qtc prolongation, uterine rupture and other interactions. So sort of like if someone is high risk , resistant org or prior culture to guide you then maybe think about it earlier but to think about it as first line or second line.

References 5-6 - 1.56% Coverage

I Usually use Bactrim its my first line yes. Bactrim for the short course you know so what time for them to forget and run out of forget to take her medication and things like that just like you know the ID for 3 days bec macrobid is like 5 to 7 days or something like that and I don't know I never used fosfomycin

References 7-8 - 3.55% Coverage

Used that in the ED for a fair number of cases..

So ED uses a lot of fosfomycin?

No some people use it..

Does it show up on a order set or something or how do u know it?

Just people talking on what can be used first line, the three I am aware of…situation like inpatient where someone was like started on something broader you know get started on like a ceftriaxone or something for an IV medication. If patient got better then we'll just continue on them on like a cephalosporin from the rationale of like they're on the Ceph and they got better on a cephalosporin you know we didn't have culture data before any abx or culture data to guide us so just continue and discharge them on cefdinir or something.

Reference 9 - 0.22% Coverage

Would just trt over the phone with Bactrim

Reference 10 - 0.19% Coverage

I like Bactrim bec of the short course.

Reference 11 - 0.35% Coverage

Not many contraindications too, no drug interactions and AKI and stuff

References 12-13 - 2.45% Coverage

you mentioned about your prescribing practices what do you think other residents do like when you see their patients later on or you see people in the hospital, What agents do you think they prescribe for the most part?

I would guess somewhere but I don't know. I don't really do much of a frame of reference to work to what all other people been doing. On the floor, people get ceftriaxone in the ED and we continue like a cephalosporin on the floor and at discharge oral, but I don't know.

<Files\\1_18_18_006> - § 6 references coded [10.42% Coverage]

Reference 1 - 1.92% Coverage

Look up your UTI would see if she is febrile, she had like flank pain or anything on exam, get a UA, on medical records see if she's had a UTI in the past and then if they're like resistant bugs then I might start something beyond the normal like Cipro or Bactrim or something.

References 2-3 - 3.50% Coverage

personal thought process and rationale for antibiotic?

I think a lot of times we end up going with things like Cipro, bactrim and Keflex for for the I have seen first line used a lot of times and then I wouldn't use Bactrim if they had like they have like CKD, a recent AKI or something like that, and then looking at allergies and then seeing what they've been on before if it's like they've been treated already with one of them and were switch to another. Asking what they've had in the past for it.

Reference 4 - 3.55% Coverage

Often told that Cipro wasn't good in terms of the like resistance profile here although when I looked it up I think Cipro and Bactrim were the same for Ecoli? 60 something percent or something.

and then Keflex for some reason doesn't really hit my mind as the UTI drug until later I just don't think about it and then Macrobid is there too but I like for some reason I see it more as like a preventative measure than it's like good treatment measure I know you're not supposed to use it like with kidney disease.

References 5-6 - 1.45% Coverage

is there certain antibiotics in your mind that you've already got kind of plan for those patients I guess based on your default that you prefer over the other ones?

I think I probably prefer Bactrim usually.

<Files\\1_18_18_007> - § 9 references coded [13.09% Coverage]

References 1-2 - 1.23% Coverage

ask about STD exposure too and discharge, get a UA and then if I mean I would just give Bactrim too at the same time or call it in after I get the UA results.

Reference 3 - 0.97% Coverage

I could use Macrobid also but I usually don't. I think the teaching is if u suspect of more complicated infection do Cipro and.

Reference 4 - 2.88% Coverage

you said you don't like Macrobid is it don't use it as often what's the do you have any beliefs about Macrobid?

it's sort of but we usually see is not 27 year old people that are healthy but like people would like recurring UTIs and have been in the hospital and usually have other like usually a lot of the UA's I've seen that the cultures have been resistant to Macrobid.

Reference 5 - 1.85% Coverage

why do you like bactrim more than some of the other agents more than Cipro?

I don't know I just remember like that's how I learned it. Also like besides for people with kidney dysfunction there's not a lot of side effects, its also only 3days

Reference 6 - 3.16% Coverage

you talked a little bit about Cipro and how you avoid avoid it unless you are worried about complicated infections. why is that?

Because there’s a lot of effects in the elderly people and then I think there's something new about a black box warning or something but I can't recall right now. That's it it's just because of the side effects in the elderly I don't think we have a lot of resistance in our country.

References 7-8 - 2.30% Coverage

do you think that certain ones are like more powerful or more likely to work for treating UTI compared to other ones?

Yeah like meropenem will work.

For outpatient?

I don't see a difference between …………..? and cipro just that I'd like just that I know that I'm supposed to use it for complicated

Reference 9 - 0.70% Coverage

IF they sound sick then I would admit them for IV or IM but otherwise Lexapro or bactrim .

<Files\\3_6_18_008> - § 4 references coded [4.73% Coverage]

References 1-2 - 1.31% Coverage

what antibiotic do you typically go to First?

8-Macrobid

M- Particular reason why Macrobid?

8- Feel like that's just what attending we staff with tend to suggest.

Reference 3 - 1.06% Coverage

no, although I know I think it was its Macrobid where if they don't make urine I think I heard in ICU that it doesn't really work as well

Reference 4 - 2.36% Coverage

so Cipro is very commonly used as well, if they have QT prolongation though I think we tend to try to avoid it. Macrobid think I mentioned before if you're not producing urine all the way to get to double-check that statement. Bactrim unless they have like a urinary or sort of a chronic kidney disease.

<Files\\3_6_18_009> - § 5 references coded [4.95% Coverage]

References 1-2 - 0.48% Coverage

-I probably treated with bactrim unless you had some reason not to be

Reference 3 - 1.19% Coverage

The FQ are falling out of favor for side effects on them so I tend to avoid those and then you could also consider Fosfomycin for not sick and less comorbidities patients.

Reference 4 - 1.37% Coverage

like with fosfomycin you wouldn't want to use that somebody who's sick. IF it's really an uncomplicated cystitis I think it might be reasonable in certain patients. bactrim is okay for most people.

Reference 5 - 1.92% Coverage

I don't remember what it looks like in that section, but I think they have some recommendations like they give a few different antibiotics like here's the first line and I think its Bactrim and I think fosfomycin and maybe Nitro nitrofurantoin maybe, which I don't know use.

<Files\\3_6_18_010> - § 3 references coded [1.66% Coverage]

References 1-2 - 1.02% Coverage

In my limited clinic experience I have treated only a few UTI, the one person I can think of offhand I use bactrim but you could also think about Macrobid ,Cipro as well

Reference 3 - 0.64% Coverage

I don't have any particular favorites and I don't I'm not aware of it there is a favorite or preferred one

<Files\\5_14_18_14> - § 4 references coded [8.54% Coverage]

References 1-2 - 2.53% Coverage

say you gotten the urine analysis back it looked consistent you felt like with her story and it felt like it was a urinary tract infections an uncomplicated urinary tract infection, what’s your usual go to antibiotic?

14—Ill probably give Cipro for like someone's young and not pregnant.

Reference 3 - 3.77% Coverage

any reason why cipro over other abx?

14—cipro tends to have really good coverage, I think there is like increasing incidence of E coli resistance to Cipro. I don't usually like bactrim as first time just because I feel like I've seen a lot of like hyperkalemia or Aki associated with that and then like really like some of the 3rd gen cephalosporins could be used as well. Other thing with Cipro is u get good urinary concentration

Reference 4 - 2.24% Coverage

any other antibiotics that you've used before for someone has a prolonged QTC or something like that.

14—consider Macrobid, I'm trying to think of what else have used.Those are probably like the major players that I would think. of after that complaint.

<Files\\Transcript_0001> - § 2 references coded [2.46% Coverage]

Reference 1 - 0.28% Coverage

Bactrim, macrobid and kephlex

Reference 2 - 2.18% Coverage

one thing that sways me are if the patient has prev UTI, we get culture data or susceptibilities of those patterns in the past also if someone was having recurring symptoms, treatment failure then I would want to change antibiotics,

Antibiotic coverage

<Files\\04_05_18_12> - § 1 reference coded [1.83% Coverage]

Reference 1 - 1.83% Coverage

-you mentioned Nitrofurantoin…Any particular reason for it?

12--its pretty cheap and people usually don't have any problems with it. I like to reserve like other bigger guns for like resistance or like worsening disease she has like a pyelo or something.

<Files\\04_05_18_13> - § 2 references coded [7.77% Coverage]

Reference 1 - 4.03% Coverage

allergies a big one. My recent UTI patient was on Bactrim prophylaxis for rheumatologic condition so we did not pick bactrim and then if they have UTIs before but there is a resistance patterns in the past and then obviously like you know is this a complicated or uncomplicated UTI, it's a man or a woman, more inclined to choose only like Cipro in a man yeah those are kind of big ones cuz I might have

Reference 2 - 3.75% Coverage

Univ of Michigan UTI I think that I read through before. Uptodate is a favorite, and admit I have not read any of the idsa things on UTI but I know those are available to me. Yeah those are the main resources and then if a culture is performed and there's a specific bacteria isolate, I look at dorsata??? Sometimes if it is not I know there is Barnes resistance patterns.

<Files\\05_14_18_15> - § 1 reference coded [6.03% Coverage]

Reference 1 - 6.03% Coverage

a lot of times especially very recently I would say you know and they haven't had a recent urinary tract infections going for an overall that I would say you know maybe we can treat this over the phone ,it depends on if there are like at high risk for developing resistance then I am like hey you need to come back and you don't have to be seen over cuz I can do all the testing know that they're otherwise okay, triage by the questions I asked you earlier, so if they if they don't have any risk factors of like your problems with developing resistance for urine tract infections or they haven't had your recurrent urinary tract infections or thing u need culture for then I can tell them to honestly speaking I wouldn't really go with the getting of urinalysis, just call them here’s like a short course for 3-5 days and just treat that. If they are high risk then get a culture so I can get some sensitivities in case they have resistance.

<Files\\1_11_18_002> - § 4 references coded [5.49% Coverage]

Reference 1 - 1.30% Coverage

newer antibiotics like FQ other than macrobid that you would consider?

If this is her first time with a UTI with no prior cultures I probably wouldnt.

Reference 2 - 1.46% Coverage

If it's someone who's had frequent UTIs or drug resistance I usually tell then to get urine studies. If it's uncomplicated like never had a history of UTIs probably dont.

Reference 3 - 2.04% Coverage

So men I would probably pick something different, pregnant women I would pick probably something different,if this is their third fourth UTI or we tried Macrobid and it didn't work there still having symptoms if I pick something different

Reference 4 - 0.69% Coverage

the VA will make you get an ID or antimicrobial stewardship consult for levoquin.

<Files\\1_11_18_004> - § 2 references coded [1.78% Coverage]

References 1-2 - 1.78% Coverage

Bactrim or Cipro. Of course inpatient I will use ceftriaxone. Inpatient if they have a resistant bug then tailor abx based on what they grow.

Do you feel like some abx are better than others?

Cipro covers at the usual bugs ecoli, klebsiella. I like bactrim and Cipro

<Files\\1_11_18_005> - § 1 reference coded [1.39% Coverage]

Reference 1 - 1.39% Coverage

FQ are excessively broad and side effects like Qtc prolongation, uterine rupture and other interactions. So sort of like if someone is high risk , resistant org or prior culture to guide you then maybe think about it earlier but to think about it as first line or second line.

<Files\\1_18_18_006> - § 3 references coded [5.47% Coverage]

Reference 1 - 1.92% Coverage

Look up your UTI would see if she is febrile, she had like flank pain or anything on exam, get a UA, on medical records see if she's had a UTI in the past and then if they're like resistant bugs then I might start something beyond the normal like Cipro or Bactrim or something.

References 2-3 - 3.55% Coverage

Often told that Cipro wasn't good in terms of the like resistance profile here although when I looked it up I think Cipro and Bactrim were the same for Ecoli? 60 something percent or something.

and then Keflex for some reason doesn't really hit my mind as the UTI drug until later I just don't think about it and then Macrobid is there too but I like for some reason I see it more as like a preventative measure than it's like good treatment measure I know you're not supposed to use it like with kidney disease.

<Files\\1_18_18_007> - § 3 references coded [5.19% Coverage]

References 1-2 - 2.88% Coverage

you said you don't like Macrobid is it don't use it as often what's the do you have any beliefs about Macrobid?

it's sort of but we usually see is not 27 year old people that are healthy but like people would like recurring UTIs and have been in the hospital and usually have other like usually a lot of the UA's I've seen that the cultures have been resistant to Macrobid.

Reference 3 - 2.30% Coverage

do you think that certain ones are like more powerful or more likely to work for treating UTI compared to other ones?

Yeah like meropenem will work.

For outpatient?

I don't see a difference between …………..? and cipro just that I'd like just that I know that I'm supposed to use it for complicated

<Files\\5_14_18_14> - § 2 references coded [3.77% Coverage]

References 1-2 - 3.77% Coverage

any reason why cipro over other abx?

14—cipro tends to have really good coverage, I think there is like increasing incidence of E coli resistance to Cipro. I don't usually like bactrim as first time just because I feel like I've seen a lot of like hyperkalemia or Aki associated with that and then like really like some of the 3rd gen cephalosporins could be used as well. Other thing with Cipro is u get good urinary concentration

Adverse events in selection

<Files\\04_05_18_011> - § 1 reference coded [3.34% Coverage]

Reference 1 - 3.34% Coverage

For men I wouldn’t take a fluoroquinolone. in case that there is if it's complicated it's more likely either ……..obstruction or other problems like bladder problems or prostate problems. For FQ penetration problems will use bactrim and then for women no I really just pick them however. whatever like whatever they were on last time or if they want to change pick something different.

<Files\\04_05_18_12> - § 2 references coded [1.20% Coverage]

References 1-2 - 1.20% Coverage

when you're picking between antibiotics is there any particular patient factors that make you pick one over the other?

12--Allergies will probably be the biggest thing

<Files\\04_05_18_13> - § 2 references coded [4.16% Coverage]

References 1-2 - 4.16% Coverage

yeah I mean allergies a big one. My recent UTI patient was on Bactrim prophylaxis for rheumatologic condition so we did not pick bactrim and then if they have UTIs before but there is a resistance patterns in the past and then obviously like you know is this a complicated or uncomplicated UTI, it's a man or a woman, more inclined to choose only like Cipro in a man yeah those are kind of big ones cuz I might have.

<Files\\1_11_18_002> - § 2 references coded [0.68% Coverage]

References 1-2 - 0.68% Coverage

Any reason why Macrobid over other antibiotics?

Least amount of side effects.

<Files\\1_11_18_003> - § 3 references coded [2.63% Coverage]

Reference 1 - 0.26% Coverage

No allergies no meds probably macrobid.

References 2-3 - 2.36% Coverage

I like Bactrim too. I don’t know I often use Macrobid over Bactrim. I try to avoid Cipro just because you know Cipro is associated with more side effects, has an increased risk of C diff and so I don't like to prescribe this unless they have complicated UTIs with resistant organisms in the past, then then that's when I used Cipro otherwise I try to avoid.

<Files\\1_11_18_005> - § 3 references coded [1.74% Coverage]

References 1-2 - 1.39% Coverage

FQ are excessively broad and side effects like Qtc prolongation, uterine rupture and other interactions. So sort of like if someone is high risk , resistant org or prior culture to guide you then maybe think about it earlier but to think about it as first line or second line.

Reference 3 - 0.35% Coverage

Not many contraindications too, no drug interactions and AKI and stuff

<Files\\1_18_18_006> - § 3 references coded [4.86% Coverage]

References 1-2 - 3.50% Coverage

personal thought process and rationale for antibiotic?

I think a lot of times we end up going with things like Cipro, bactrim and Keflex for for the I have seen first line used a lot of times and then I wouldn't use Bactrim if they had like they have like CKD, a recent AKI or something like that, and then looking at allergies and then seeing what they've been on before if it's like they've been treated already with one of them and were switch to another. Asking what they've had in the past for it.

Reference 3 - 1.36% Coverage

Macrobid is there too but I like for some reason I see it more as like a preventative measure than it's like good treatment measure I know you're not supposed to use it like with kidney disease.

<Files\\1_18_18_007> - § 5 references coded [7.26% Coverage]

References 1-2 - 1.86% Coverage

why do you like bactrim more than some of the other agents more than Cipro?

I don't know I just remember like that's how I learned it. Also like besides for people with kidney dysfunction there's not a lot of side effects, its also only 3days

References 3-4 - 3.16% Coverage

you talked a little bit about Cipro and how you avoid avoid it unless you are worried about complicated infections. why is that?

Because there’s a lot of effects in the elderly people and then I think there's something new about a black box warning or something but I can't recall right now. That's it it's just because of the side effects in the elderly I don't think we have a lot of resistance in our country.

Reference 5 - 2.24% Coverage

do you routinely think about the black box warnings for other things will just get into your medical decision-making?

For ………? Not really but like the delirium the encephalopathy type of person

Would u include younger patients too?.

Not as worried in younger patients. Oh okay and then.

<Files\\3_6_18_008> - § 4 references coded [3.44% Coverage]

References 1-2 - 1.08% Coverage

no, although I know I think it was its Macrobid where if they don't make urine I think I heard in ICU that it doesn't really work as well

References 3-4 - 2.36% Coverage

so Cipro is very commonly used as well, if they have QT prolongation though I think we tend to try to avoid it. Macrobid think I mentioned before if you're not producing urine all the way to get to double-check that statement. Bactrim unless they have like a urinary or sort of a chronic kidney disease.

<Files\\3_6_18_009> - § 2 references coded [1.90% Coverage]

Reference 1 - 1.20% Coverage

The FQ are falling out of favor for side effects on them so I tend to avoid those and then you could also consider Fosfomycin for not sick and less comorbidities patients.

Reference 2 - 0.70% Coverage

I kind of mentioned it earlier like with fosfomycin you wouldn't want to use that somebody who's sick.

<Files\\3_6_18_010> - § 2 references coded [2.32% Coverage]

References 1-2 - 2.32% Coverage

For Bactrim , obviously if they have renal insufficiency or issues with hyperkalemia or something like that that would not be an option otherwise other things like allergies of course but that seems pretty obvious and things not worked for them in the past if they even had urinary tract infections in the past that worked with a certain medication that would influence my decision

<Files\\5_14_18_14> - § 4 references coded [6.03% Coverage]

References 1-2 - 3.80% Coverage

any reason why cipro over other abx?

14—cipro tends to have really good coverage, I think there is like increasing incidence of E coli resistance to Cipro. I don't usually like bactrim as first time just because I feel like I've seen a lot of like hyperkalemia or Aki associated with that and then like really like some of the 3rd gen cephalosporins could be used as well. Other thing with Cipro is u get good urinary concentration.

References 3-4 - 2.24% Coverage

any other antibiotics that you've used before for someone has a prolonged QTC or something like that.

14—consider Macrobid, I'm trying to think of what else have used.Those are probably like the major players that I would think. of after that complaint.

<Files\\Transcript_0001> - § 1 reference coded [0.61% Coverage]

Reference 1 - 0.61% Coverage

,so allergies………5.15-5.20……………… - Bactrim, macrobid and kephlex

**IMPACT OF GUIDELINES AND TRAINING EFFECT ON UTI MANAGEMENT**

Provider training

<Files\\04_05_18_011> - § 1 reference coded [4.09% Coverage]

Reference 1 - 4.09% Coverage

 so I guess for physicians CME credits would be educational and helpful. As far as the residency goes it's not really like a competency level for how to prescribe antibiotics for UTI is so, addressing it a little bit more every time you see a patient with UTI, it's more of like you said earlier I have to make a commitment to knowing what the guidelines are in order to follow them. Otherwise no I can't think of any great way of reinforcing good practice guidelines.

<Files\\04_05_18_12> - § 1 reference coded [2.78% Coverage]

Reference 1 - 2.78% Coverage

-Maybe kind of as incentive kind of offer CME credit for it. You know that way it kind of gives people a reason to kind of step away. CME is not something that's kind of always on our minds as residents but I know in the future practicing folks keep an eye on, may be do it that way. It won't be as intrusive or feel like someone’s like breathing down your neck, least that's what I think.

<Files\\04_05_18_13> - § 1 reference coded [3.04% Coverage]

Reference 1 - 3.04% Coverage

U mentioned apps but what are the other ways that providers could stay uptodate with guidelines?

13—Like email newsletters, apps are a good way and CME activities, we have formal lectures and things I'm not sure of what happens like after residency but some things I can think of off the top of my head

<Files\\05_14_18_15> - § 1 reference coded [3.67% Coverage]

Reference 1 - 3.67% Coverage

What ends up happening is that I mean it it's hard to stratify patients to be the same way and that we use more healthcare dollars and more aggressive practices individuals that you know it's not even show African American, underserved populations , Medicaid patients overall, so the question how do you stratify? How do you stratify specifically for this? Could be a lot of ????to confirm that that is the case and you can find many reasons why I can't go against the grain. In general is going to be hard to try to stratify that specially on a resident level that as well.

<Files\\1_11_18_002> - § 1 reference coded [2.30% Coverage]

Reference 1 - 2.30% Coverage

we'd like to think or talk guideline to finding you know guideline based medicine lot of times when you talk with different attendings what they say is based more off of their own practice and not based off of guidelines for until those habits become learned habits.

<Files\\1_11_18_003> - § 1 reference coded [0.78% Coverage]

Reference 1 - 0.78% Coverage

I think also that you know the attending’s that we staff with should also you know because they are responsible as well

<Files\\1_11_18_005> - § 2 references coded [0.53% Coverage]

References 1-2 - 0.53% Coverage

And when u think u looked it up? when you were an intern or medical student ?

Sometime during intern year.

<Files\\1_18_18_006> - § 1 reference coded [2.44% Coverage]

Reference 1 - 2.44% Coverage

SO u get a lot of variation with attendings?

I think early on for me sometimes it's like I disagree but I don't feel like I can say anything but then I feeling especially with the interpretation. I think usually in clinic when we've actually treated it's been pretty straightforward, but there have been situations where that seemed a little aggressive

<Files\\1_18_18_007> - § 2 references coded [0.97% Coverage]

Reference 1 - 0.63% Coverage

I think the teaching is if u suspect of more complicated infection do Cipro and.

Reference 2 - 0.34% Coverage

I just remember like that's how I learned it.

<Files\\3_6_18_008> - § 2 references coded [3.30% Coverage]

Reference 1 - 1.31% Coverage

what antibiotic do you typically go to First?

8-Macrobid

M- Particular reason why Macrobid?

8- Feel like that's just what attending we staff with tend to suggest.

Reference 2 - 1.99% Coverage

But honestly a lot of the times we practice what our attending’s practice to a certain extent so I don't remember if you mention like in service lectures or something like that, but in service lectures to attendings and or us, I think those would be useful

Provider resources

<Files\\04_05_18_011> - § 3 references coded [1.66% Coverage]

References 1-2 - 0.71% Coverage

so the guidelines usually say 3 days for uncomplicated. up to date to choose abx

Reference 3 - 0.95% Coverage

u mentioned uptodate, any other resources or things along the line same lines that you use?

11-- ……..Com?

<Files\\04_05_18_12> - § 2 references coded [2.64% Coverage]

References 1-2 - 2.64% Coverage

Do u use any specific resources, guidelines to help you make decisions about picking abx , dose,duration?

12--in general I lookup uptodate if you have any questions about antibiotics and you know sometimes I may look up in my like trustee pocket medicine book as well, these guidelines are sometimes in uptodate as well. If there's a interesting article I'll probably

<Files\\04_05_18_13> - § 3 references coded [8.04% Coverage]

References 1-2 - 4.25% Coverage

I have not read them in a while but there is like Univ of Michigan UTI I think that I read through before. Uptodate is a favorite, and admit I have not read any of the idsa things on UTI but I know those are available to me. Yeah those are the main resources and then if a culture is performed and there's a specific bacteria isolate, I look at dorsata??? Sometimes if it is not I know there is Barnes resistance patterns.

Reference 3 - 3.80% Coverage

How do you feel like that information should be shared with primary care doctors?

13--I mean I I like apps for lot . I use my uptodate app, appocrates app all the time, yeah I think apps are like this the fastest practical way for those guidelines actually be put into use otherwise I mean I think you know guideline statement that can be accessed online are also good to have.

<Files\\05_14_18_15> - § 3 references coded [8.35% Coverage]

References 1-2 - 0.30% Coverage

Up to date. Most of my information from there.

Reference 3 - 8.05% Coverage

-I mean like I know they have like idsa guidelines, I read those like a year or two ago. I've read Harrison's for a UTI and cystitis and it's been a while back. how these should be communicated or distributed?

M—How should primary care docs be informed? Is it upto pcp to stay uptodate or? Clinic leadership or Hospital leadership?

15--my belief is that the doctor is you know it's up to the doctors to make sure that they give the proper care and stay uotodate with the literature. unfortunately the case is that an individual or population that there are people that and I’d say a lot probably that are lacking so that it becomes more maybe if the individual can't fix problem clean environment that will help to fix the problem and then I think it comes down to the systems that in terms of costs and benefits and the system that doctors are part of that the doctors are presented as well, more people involved for distributing the resources the better, so if I was a primary care doc and if we had like the newsletter or like need like it's kind of updates updates or something like guidelines like these are summary of what the guidelines are. you know it's hard for doc to go through if the information is too long or maybe summarize and bring it out.

<Files\\1_11_18_002> - § 4 references coded [2.83% Coverage]

References 1-3 - 1.93% Coverage

Do you to any specific resources or guidelines to help make your decisions as far as when and when not to treat, what antibiotics to use ?

Up to date

Any other resource?

so I looked at the ISDA but I don't use it regularly

Reference 4 - 0.90% Coverage

You know I'm not sure, so up-to-date is very wordy and redundant, can get a little trasking to read over.

<Files\\1_11_18_003> - § 4 references coded [1.04% Coverage]

References 1-2 - 0.21% Coverage

I frequently review up to date.

References 3-4 - 0.83% Coverage

Not much for UTIs. May be look at antibiograms that Barnes sends out just to look at different bugs and antibiotic resistance.

<Files\\1_11_18_004> - § 5 references coded [1.75% Coverage]

References 1-2 - 0.70% Coverage

I guess why Bactrim over any other antibiotic?

From Up to date , it's like first line for uncomplicated.

References 3-4 - 0.25% Coverage

MAcromedics, uptodate and U Central

Reference 5 - 0.80% Coverage

Uptodate more often

What about Uptodate u like?

Have an app on my phone and its more accessible, use it anywhere anytime.

<Files\\1_11_18_005> - § 5 references coded [2.35% Coverage]

References 1-2 - 0.61% Coverage

I probably looked it up in up-to-date at one point in time and so I was like the shortest course and I'm stuck with that.

References 3-4 - 0.99% Coverage

So beyond up-to-date is there anything else that you used to or not?

For like UTI antibiotics for like other things….. I've used the toolbook for other things I don't really use it for UTIs at all.

Reference 5 - 0.76% Coverage

Or if they want us to use the ACP guideline or that’s what they recommend or ID guidelines or any

particular set of guidelines they feel as strong.

<Files\\1_18_18_006> - § 4 references coded [4.60% Coverage]

Reference 1 - 1.92% Coverage

Look up your UTI would see if she is febrile, she had like flank pain or anything on exam, get a UA, on medical records see if she's had a UTI in the past and then if they're like resistant bugs then I might start something beyond the normal like Cipro or Bactrim or something.

Reference 2 - 0.61% Coverage

I have looked up before like the antibiotic gram just to make sure it seems reasonable.

References 3-4 - 2.07% Coverage

Uptodate or Ap..ese

It’s usually the one on your phone that I imagine what all the residents do it? are there other guidelines or resources some people will use like you know ACP or idsa or like pocket medicine or other things it sounds like mostly your go to are uptodate?

Have not see a UTI page…

<Files\\1_18_18_007> - § 5 references coded [4.85% Coverage]

References 1-2 - 1.89% Coverage

Yeah I mostly use uptodate. I don't really check the antibiogram.

Do you check it every time or at this point u know all the information?

I check it every time.

Any other resources outside of up-to-date or is that the main ?

Use up todate.

Reference 3 - 2.22% Coverage

Infection specifically….I sometimes go to the IDSA website ,so that would be a good place to put them.

and as far as who's responsible maybe just the societies that are incharge maybe

So it's the responsibility of the physician to study information periodically or going on up to date..

References 4-5 - 0.74% Coverage

I look at the new England journal. If UTI stuff makes it there then I will not miss but otherwise

<Files\\3_6_18_008> - § 5 references coded [8.79% Coverage]

References 1-2 - 0.86% Coverage

I usually check up to date, see if they have initial recommendation, up to date for the dosing recommendations.

Reference 3 - 1.64% Coverage

it's just easy to get to, sort of an online copy, the URL is short. I don't have Micro like I still have to set up micromedics. Apocrates, people don’t like it and I don’t have a subscription for that either.

Reference 4 - 4.15% Coverage

I guess there's probably guidelines on the idsa website. I mean I think people probably should know that they are the idsa guidelines, I don't know if there's a more easily I mean I think people tend to go to up to date sort of as a reflex so as long as up to date is current with idsa guidelines im sure that people would use it, I think they probably use up to date more cuz it's a little bit easier to consume information. The idsa guidelines are usually inside of a big long PDF and I have to look through it a little bit more.

Reference 5 - 2.14% Coverage

yeah so all of the idsa guidelines are available on PDF on the website but like with our computers random things get blocked I'm sure we can download the PDF but if they had like a summary just like on an HTML website that would be easier than downloading something opening It

<Files\\3_6_18_009> - § 4 references coded [4.70% Coverage]

References 1-2 - 0.92% Coverage

I look at up to date and then the idsa guidelines have a catheter associate UTI section which tends not to be relevant to outpatient

Reference 3 - 2.50% Coverage

Convenience, ease of use, any particular reasons why you gravitate towards that?

9-- it tends to be up to date, they do update it and and it's usually current information. I perceive it to be evidence based. and it is very convenient on my phone, it's on the computer. Its easily searchable. If u use google, it's hard to find what you're looking for sometimes

Reference 4 - 1.28% Coverage

I saw something and I think ACP magazine recently about something like this about somebody's opinion that we are way over treating UTIs so it's something I think about a little more.

<Files\\3_6_18_010> - § 4 references coded [4.20% Coverage]

References 1-2 - 0.77% Coverage

Do u use any specific resources or guidelines when looking up antibiotics and looking up duration?

10—Usually I use uptodate

References 3-4 - 3.43% Coverage

the Sanford guide is something that I've seen a lot of people use and I should know this but I assume up-to-date and Stanford guide are kind of congruent in their recommendations but actually that's information I don't know off the top of my head and also the IDSA, I mean sort of broad infection kind of recommendations I know the idsa has guidelines but I can't say that every time I review that but I'm aware of them and you know if I had someone probably with something more complicated than just a regular UTI infection I would maybe think about looking at those.

<Files\\5_14_18_14> - § 4 references coded [4.90% Coverage]

References 1-3 - 2.03% Coverage

the Johns Hopkins abx guide.

M—For things in general, in residency as far as things that are directly like UTI or antibiotic-related ?

14—I have Ucentral so it has Wash U manual and John Hopkins on my phone and I use that a lot.

Reference 4 - 2.86% Coverage

I've seen the idsa guidelines for some things and it tends to be like very comprehensive it like a long document with like all possible scenarios and I don't see like many Primary Care people going through all of that, so maybe kind of like a one-page like summarization like general things and then like when to refer something

<Files\\Transcript_0001> - § 2 references coded [0.45% Coverage]

References 1-2 - 0.45% Coverage

Generally go to upto date if I have questions

Opinions guidelines useful

<Files\\3_6_18_010> - § 1 reference coded [2.76% Coverage]

Reference 1 - 2.76% Coverage

then yeah as far as like specific guidelines, trying to read those it seems like it for me it's kind of more situational like when I'm faced with a situation then I review review them but I don't have a specific like study practice of just opening up kind of guidelines at whim but that would be smart I guess to try to just independent because sometimes you don't know when you know these things are going to come up and then you hadn’t read them in a year.

Opinion guidelines not useful

<Files\\05_14_18_15> - § 1 reference coded [8.05% Coverage]

Reference 1 - 8.05% Coverage

-I mean like I know they have like idsa guidelines, I read those like a year or two ago. I've read Harrison's for a UTI and cystitis and it's been a while back. how these should be communicated or distributed?

M—How should primary care docs be informed? Is it upto pcp to stay uptodate or? Clinic leadership or Hospital leadership?

15--my belief is that the doctor is you know it's up to the doctors to make sure that they give the proper care and stay uotodate with the literature. unfortunately the case is that an individual or population that there are people that and I’d say a lot probably that are lacking so that it becomes more maybe if the individual can't fix problem clean environment that will help to fix the problem and then I think it comes down to the systems that in terms of costs and benefits and the system that doctors are part of that the doctors are presented as well, more people involved for distributing the resources the better, so if I was a primary care doc and if we had like the newsletter or like need like it's kind of updates updates or something like guidelines like these are summary of what the guidelines are. you know it's hard for doc to go through if the information is too long or maybe summarize and bring it out.

<Files\\1_11_18_002> - § 1 reference coded [0.71% Coverage]

Reference 1 - 0.71% Coverage

, so up-to-date is very wordy and redundant, can get a little trasking to read over

<Files\\1_11_18_003> - § 2 references coded [3.59% Coverage]

References 1-2 - 3.59% Coverage

Is there any particular adherence range that would be acceptable?

Social question let me see. I don't know what would be fair I would say that maybe if you were to

So let's say survey all physicians and have the median with the standard deviation I think we should fall within and you should not be outside two standard deviations of not following compared to other Physicians but has like a minimum. What's the maximum, I don’t know there are so many guidelines out there it's always hard to be updated on all of them you know so I don't know.

<Files\\1_11_18_004> - § 1 reference coded [1.36% Coverage]

Reference 1 - 1.36% Coverage

The order sets will be really helpful and we can have the EPIC like EMR the alert, if you're not prescribing again according to best practices with like the correct like practice I think that will be helpful.

<Files\\1_11_18_005> - § 3 references coded [9.77% Coverage]

Reference 1 - 2.71% Coverage

I know there's a bunch of guidelines and other things that sometimes are out there for various conditions but it can get confusing right so any of them it's hard to keep up with them so if they existed on how to treat a UTI how do you think they should be shared with it with especially with there being so many guidelines out there I know at least when I was a resident they had like you know ACP released guidelines , the US preventive Services Task Force released guidelines but they might be different than you know the Hemeonc guidelines ?

Reference 2 - 3.53% Coverage

I think publishing in a journal wont reach a ton of people, I think cards are great I think also we have like a Clinic tool book that's online that I think could be more useful than it is and stuff like that yeah I don't need that all the evidence presented to me when I'm just trying to make this decision you know you can have a link to the evidence so basically I just wanted to know like which antibiotics is first line Second Line third line and like what are the contraindications what are the side effects I can have that.

actionable decision affecting information kind of right there yeah not in like a long paragraph or like a 10-page list of recommendations and summarizing the evidence and stuff.

Reference 3 - 3.53% Coverage

So that’s actually useful information people can have and maybe they have their own reasons why they are prescribing these things but I think at least being aware like we all know they're prescribing so much they know they're not in accordance with the guidelines or maybe think guidelines are garbage, but you know they would have the information and be acting on information that they have rather than just kind of a habit of prescribing Cipro first line.

Some people think guidelines are garbage so that's a good point. Sometimes they're made up by people that sit in their Ivory Tower and aren't seeing patients all the time don't see all of the different clinical scenario that make it more challenging

<Files\\1_18_18_007> - § 1 reference coded [9.08% Coverage]

Reference 1 - 9.08% Coverage

I don't know I mean, there's so much like more than just the like when providers make different decisions if there's so much more information that goes into them then guidelines so I sometimes get frustrated with you the things that are in place right now to evaluate , like my diabetes metrics for example…Did u have them too? Every 6 months we have to like see if we've documented a foot exam and if they have an upto date for the exam and if they have any like if their A1C is going the right direction but. Like there are some patients who are you know they have diabetes on paper but they're just like diabetes is not the thing that's going to affect their life for like they have so many other comorbidities I like I'm trying to get them to do their cancer screening and like like if you add diabetes and like adding a foot exam. it just it doesn't it's not the right way to care for the patient like you shouldn't be aggressive with her diabetes cuz it'll sacrifice care of other things and so those patients have higher diabetes numbers or they don't have a foot exam but I don't think it really affects their care but if you look at the numbers I'm like 50% compliant or something

<Files\\3_6_18_008> - § 2 references coded [6.28% Coverage]

Reference 1 - 4.15% Coverage

I guess there's probably guidelines on the idsa website. I mean I think people probably should know that they are the idsa guidelines, I don't know if there's a more easily I mean I think people tend to go to up to date sort of as a reflex so as long as up to date is current with idsa guidelines im sure that people would use it, I think they probably use up to date more cuz it's a little bit easier to consume information. The idsa guidelines are usually inside of a big long PDF and I have to look through it a little bit more.

Reference 2 - 2.14% Coverage

yeah so all of the idsa guidelines are available on PDF on the website but like with our computers random things get blocked I'm sure we can download the PDF but if they had like a summary just like on an HTML website that would be easier than downloading something opening It

<Files\\3_6_18_009> - § 1 reference coded [2.50% Coverage]

Reference 1 - 2.50% Coverage

Convenience, ease of use, any particular reasons why you gravitate towards that?

9-- it tends to be up to date, they do update it and and it's usually current information. I perceive it to be evidence based. and it is very convenient on my phone, it's on the computer. Its easily searchable. If u use google, it's hard to find what you're looking for sometimes

<Files\\3_6_18_010> - § 1 reference coded [2.15% Coverage]

Reference 1 - 2.15% Coverage

so it takes a little bit of time sometimes to find like the exact have it that you want because there's four different articles for the same thing that just break it down into like epidemiology and then that's like the one article so but overall I mean I like it and I don't really thankful that we have it as part of our package and that is included for us

<Files\\5_14_18_14> - § 2 references coded [2.89% Coverage]

References 1-2 - 2.89% Coverage

I've seen the idsa guidelines for some things and it tends to be like very comprehensive it like a long document with like all possible scenarios and I don't see like many Primary Care people going through all of that, so maybe kind of like a one-page like summarization like general things and then like when to refer something.

Guidelines role in selection

<Files\\04_05_18_011> - § 1 reference coded [0.21% Coverage]

Reference 1 - 0.21% Coverage

up to date to choose abx

<Files\\04_05_18_12> - § 1 reference coded [1.85% Coverage]

Reference 1 - 1.85% Coverage

--in general I lookup uptodate if you have any questions about antibiotics and you know sometimes I may look up in my like trustee pocket medicine book as well, these guidelines are sometimes in uptodate as well. If there's a interesting article I'll probably

<Files\\04_05_18_13> - § 1 reference coded [4.25% Coverage]

Reference 1 - 4.25% Coverage

I have not read them in a while but there is like Univ of Michigan UTI I think that I read through before. Uptodate is a favorite, and admit I have not read any of the idsa things on UTI but I know those are available to me. Yeah those are the main resources and then if a culture is performed and there's a specific bacteria isolate, I look at dorsata??? Sometimes if it is not I know there is Barnes resistance patterns.

<Files\\05_14_18_15> - § 1 reference coded [0.30% Coverage]

Reference 1 - 0.30% Coverage

Up to date. Most of my information from there.

<Files\\1_11_18_002> - § 1 reference coded [0.73% Coverage]

Reference 1 - 0.73% Coverage

Up to date

Any other resource?

so I looked at the ISDA but I don't use it regularly

<Files\\1_11_18_004> - § 1 reference coded [0.23% Coverage]

Reference 1 - 0.23% Coverage

MAcromedics, uptodate and U Central

<Files\\1_11_18_005> - § 1 reference coded [0.62% Coverage]

Reference 1 - 0.62% Coverage

I probably looked it up in up-to-date at one point in time and so I was like the shortest course and I'm stuck with that.

<Files\\1_18_18_007> - § 2 references coded [0.77% Coverage]

Reference 1 - 0.52% Coverage

Yeah I mostly use uptodate. I don't really check the antibiogram.

Reference 2 - 0.25% Coverage

I look at the new England journal

<Files\\3_6_18_008> - § 1 reference coded [0.86% Coverage]

Reference 1 - 0.86% Coverage

I usually check up to date, see if they have initial recommendation, up to date for the dosing recommendations.

<Files\\3_6_18_010> - § 1 reference coded [3.40% Coverage]

Reference 1 - 3.40% Coverage

Sanford guide is something that I've seen a lot of people use and I should know this but I assume up-to-date and Stanford guide are kind of congruent in their recommendations but actually that's information I don't know off the top of my head and also the IDSA, I mean sort of broad infection kind of recommendations I know the idsa has guidelines but I can't say that every time I review that but I'm aware of them and you know if I had someone probably with something more complicated than just a regular UTI infection I would maybe think about looking at those.

<Files\\5_14_18_14> - § 2 references coded [1.03% Coverage]

Reference 1 - 0.24% Coverage

the Johns Hopkins abx guide

Reference 2 - 0.79% Coverage

I have Ucentral so it has Wash U manual and John Hopkins on my phone and I use that a lot.

Dissemination of guidelines

<Files\\04_05_18_011> - § 2 references coded [6.50% Coverage]

Reference 1 - 3.80% Coverage

So based on those guidelines how do you feel like the people that put those guidelines out there should get those guidelines to the primary care doctors and to people prescribing antibiotics? how do you feel like that information should be shared? U think a publication or additional resources that you feel like would be helpful any handout or anything like that?

11- I don't think apps are useful. Also we have handout for everything

Reference 2 - 2.70% Coverage

As far as the residency goes it's not really like a competency level for how to prescribe antibiotics for UTI is so, addressing it a little bit more every time you see a patient with UTI, it's more of like you said earlier I have to make a commitment to knowing what the guidelines are in order to follow them.

<Files\\04_05_18_12> - § 3 references coded [4.62% Coverage]

References 1-3 - 4.62% Coverage

Good question. Best way to get it out I mean I would probably be probably be in published in the pretty well-read journal, if not already, so put it in Jama ,put in New England Journal .

M—Do u think publication is enough? Do u think providers should be responsible for looking infoemation?

12—Probably wouldn't be enough; probably wouldn't be enough, a lot of people use social media now to kind of disseminate information. I follow this like guy on Instagram who puts up like this renal guy puts up like slides for like renal casts so I got to know if maybe like you know Twitter whatever they can just put a link to their most recent guidelines.

<Files\\04_05_18_13> - § 4 references coded [6.84% Coverage]

References 1-2 - 3.80% Coverage

How do you feel like that information should be shared with primary care doctors?

13--I mean I I like apps for lot . I use my uptodate app, appocrates app all the time, yeah I think apps are like this the fastest practical way for those guidelines actually be put into use otherwise I mean I think you know guideline statement that can be accessed online are also good to have.

References 3-4 - 3.05% Coverage

U mentioned apps but what are the other ways that providers could stay uptodate with guidelines?

13—Like email newsletters, apps are a good way and CME activities, we have formal lectures and things I'm not sure of what happens like after residency but some things I can think of off the top of my head

<Files\\05_14_18_15> - § 3 references coded [9.55% Coverage]

References 1-2 - 8.05% Coverage

-I mean like I know they have like idsa guidelines, I read those like a year or two ago. I've read Harrison's for a UTI and cystitis and it's been a while back. how these should be communicated or distributed?

M—How should primary care docs be informed? Is it upto pcp to stay uptodate or? Clinic leadership or Hospital leadership?

15--my belief is that the doctor is you know it's up to the doctors to make sure that they give the proper care and stay uotodate with the literature. unfortunately the case is that an individual or population that there are people that and I’d say a lot probably that are lacking so that it becomes more maybe if the individual can't fix problem clean environment that will help to fix the problem and then I think it comes down to the systems that in terms of costs and benefits and the system that doctors are part of that the doctors are presented as well, more people involved for distributing the resources the better, so if I was a primary care doc and if we had like the newsletter or like need like it's kind of updates updates or something like guidelines like these are summary of what the guidelines are. you know it's hard for doc to go through if the information is too long or maybe summarize and bring it out.

Reference 3 - 1.49% Coverage

If we have a committee that send out like updates like maybe like once a week to send like email but like I probably maybe once a week or once a month high-profile like changes and trends in medical care that's really not a bad idea.

<Files\\1_11_18_002> - § 4 references coded [5.25% Coverage]

References 1-2 - 3.24% Coverage

I don't think our Clinic has like the best way to give us guidelines, we talk about guidelines during lectures and when you're on the third for the day nothing's getting absorbed, so if there was a better way I think if you know we have the SharePoint document and if there was like literally like 10 lines or an algorithm that makes it really simple that would be the best way.

Reference 3 - 0.76% Coverage

I think it’s the providers. If you are prescribing then you should look up for guidelines

Reference 4 - 1.26% Coverage

What about educational materials, posters hand out, that were distributed in a regular basis to the resident.?

3. I also have presentation fatigue

<Files\\1_11_18_003> - § 4 references coded [2.92% Coverage]

References 1-2 - 1.27% Coverage

It probably would be best to have to send it out to everyone and then after that to just have it made available and posted in the clinic. If I know its available may be take a quick look at it.

Reference 3 - 0.79% Coverage

I think also that you know the attending’s that we staff with should also you know because they are responsible as well.

Reference 4 - 0.86% Coverage

What about educational materials given to residents in clinics. Handouts, posters etc.

I say that probably not as helpful about 2.

<Files\\1_11_18_004> - § 4 references coded [1.94% Coverage]

References 1-2 - 1.21% Coverage

If clinical guidelines existed on best practice to treat non complicated UTI, how should they be shared with primary care physicians?

I like apps that would be nice, may be pocket card.

References 3-4 - 0.72% Coverage

I think ultimately the prescribers are responsible but having a noon conference or didactics will be helpful.

<Files\\1_11_18_005> - § 9 references coded [12.75% Coverage]

References 1-2 - 1.59% Coverage

It would be most useful for like institution guidelines especially for the antibiotic where you know the susceptibilities may vary from location to location and you know what antibiotics are going to be on the shortage at any one given time. Lot of emails on shortage…I don't read them anymore unless I feel relevant.

References 3-4 - 3.53% Coverage

I think publishing in a journal wont reach a ton of people, I think cards are great I think also we have like a Clinic tool book that's online that I think could be more useful than it is and stuff like that yeah I don't need that all the evidence presented to me when I'm just trying to make this decision you know you can have a link to the evidence so basically I just wanted to know like which antibiotics is first line Second Line third line and like what are the contraindications what are the side effects I can have that.

actionable decision affecting information kind of right there yeah not in like a long paragraph or like a 10-page list of recommendations and summarizing the evidence and stuff.

References 5-6 - 2.30% Coverage

I think the clinic tool book for this clinic, a whole lecture will be excessive

I'm not as familiar with the clinic so is it an electronic version?

It’s an electronic PDF , was maybe meant to be printed out and now it's just kind of a scanned PDF document online and there's like a SharePoint website that we all have access to and it's there along with like a bunch of other half forms and things that we might need it in the course of our clinic activities

Reference 7 - 0.78% Coverage

No it is not. It should be but is not. It should be like a webpage scrolling through pages. I think it also be easier for people to update rather than a PDF.

Reference 8 - 2.26% Coverage

Anywhere along the spectrum will be useful like part of annual report will be useful. We have meetings with our PDs and stuff where they go over things twice a year, I guess its similar for any doctor with like a performance review you know someone or whatever and it could be something included in there. Your RVU and Abx adherence can be put in there. People like numbers. People also get competitive I wanna get a higher number than what was last year.

Reference 9 - 2.28% Coverage

So that’s actually useful information people can have and maybe they have their own reasons why they are prescribing these things but I think at least being aware like we all know they're prescribing so much they know they're not in accordance with the guidelines or maybe think guidelines are garbage, but you know they would have the information and be acting on information that they have rather than just kind of a habit of prescribing Cipro first line.

<Files\\1_18_18_006> - § 3 references coded [5.15% Coverage]

References 1-2 - 4.20% Coverage

It would be good to have it in the form of like pocket card and we have a lot of pocket card so it's hard to say we want another one of those but just something, it would be good for I guess the attendings to talk about it but it's something that's so like pervasive that almost I don't think anyone like really wants to spend a whole lot of time dedicated to it. I could imagine like a couple lectures like maybe one for impatiens one for outpatient could be helpful since we see so much on both sides. I know some of the stuff is almost like protocol I think what we're allowed to order in the hospital. In

Reference 3 - 0.95% Coverage

Are there guidelines?

So there are guidelines that were released in an ID journal in 2011 and said they weren't adequately disseminated.

<Files\\1_18_18_007> - § 6 references coded [7.40% Coverage]

Reference 1 - 2.11% Coverage

Up to date , yeah I guess you could put it. where else can you put it ,I mean I wouldn't read the article on UTIs on up to date anymore when I'm working at the dosages for UTI and if they wrote like preferred , like these antibiotics are the first line then would be helpful.

References 2-3 - 2.14% Coverage

What about publishing in a journal you think that's sufficient or and it would be the clinicians responsibility to review the publication ?

That’s fine too, that's how we get a lot of our information now and eventually makes it up makes its way into a board questions but slower.

References 4-5 - 2.22% Coverage

Infection specifically….I sometimes go to the IDSA website ,so that would be a good place to put them.

and as far as who's responsible maybe just the societies that are incharge maybe

So it's the responsibility of the physician to study information periodically or going on up to date..

Reference 6 - 0.94% Coverage

The electronic medical record, yeah when you ask me like how should we disseminate that information an emr is a good place.

<Files\\3_6_18_008> - § 6 references coded [8.21% Coverage]

References 1-2 - 4.15% Coverage

I guess there's probably guidelines on the idsa website. I mean I think people probably should know that they are the idsa guidelines, I don't know if there's a more easily I mean I think people tend to go to up to date sort of as a reflex so as long as up to date is current with idsa guidelines im sure that people would use it, I think they probably use up to date more cuz it's a little bit easier to consume information. The idsa guidelines are usually inside of a big long PDF and I have to look through it a little bit more.

References 3-4 - 2.08% Coverage

Yeah I think so and if associated with large entities it is useful to have lectures ,where they sort of just remind people or just let people know that there have been updates because I think people tend to just go back to the same resource which may be outdated.

References 5-6 - 1.99% Coverage

But honestly a lot of the times we practice what our attending’s practice to a certain extent so I don't remember if you mention like in service lectures or something like that, but in service lectures to attendings and or us, I think those would be useful

<Files\\3_6_18_009> - § 6 references coded [8.23% Coverage]

References 1-2 - 2.81% Coverage

how do you feel like that is sort of entities should get their information out to people like yourself?

9--I guess they don't really do that. up to date doesn't send you a a Weekly Newsletter.

but yeah yeah I don't know I wouldn't mind like getting but I say like a weekly email from up-to-date says hey here's three key clinical facts for this or recommendations for this week or whatever I don't know.

References 3-4 - 3.27% Coverage

other than looking things up on uptodate, do u feel like there's any other way , you mentioned the email with the newsletters, you feel like there any other ways could be used to get this information to primary care doctors?

9--for some reason drug companies came to mind but we don't we don't have a new antibiotics that were using hardly ever for UTIs. For newer cancer drugs and stuff they r in your office telling you how great it is ,but for antibiotics I don't know

References 5-6 - 2.14% Coverage

for me if you just emailed me and said hey this is what we should be doing from for now on look at this research that I'm probably just changed her practice and do it, could be as simple as just an email.

Could do you like a noon conference on it if you really want to drive it home. I'm not sure what else.

<Files\\3_6_18_010> - § 2 references coded [4.21% Coverage]

References 1-2 - 4.21% Coverage

like how those Society should disseminate them? Well I mean it would be nice or it is helpful when there's like review articles that are written by people that are in journals that most primary care doctors would read that would be one way. I don't know emails I'm bad I get a lot of like Medscape and random kind of medically related emails and maybe look at one or two headlines and delete it. That Could be the way maybe for some people for me personally it doesn't always click with me but that would be one way if they could you know get it in sort of popular email chain where maybe people are reading and would be able to click it and save on their computer or print it out whatever they want.

<Files\\Transcript_0001> - § 3 references coded [3.46% Coverage]

References 1-2 - 2.80% Coverage

.Resident clinic something like that, I think like u probably fairy concisely give a rundown of recommendations of preferred antibiotics and durations. May be print and give out a card or paste on the wall where it might be lost with a bunch of similar other things but would be available that way.

Reference 3 - 0.66% Coverage

Potential to be impactful but gets lost among other things. 2 out of 5

**RESPONSIBILITY AND BENCHMARKING**

Provider peer behavior

<Files\\1_11_18_002> - § 2 references coded [5.11% Coverage]

Reference 1 - 2.30% Coverage

we'd like to think or talk guideline to finding you know guideline based medicine lot of times when you talk with different attendings what they say is based more off of their own practice and not based off of guidelines for until those habits become learned habits.

Reference 2 - 2.81% Coverage

Any sort of like display of public commitment maybe not necessarily in the Resident clinic but like attendings in general if they were to come out in their clinic and say I will follow the best practice guidelines for prescribing antibiotics would you feel like that would have an effect on on other providers in prescribing.?

1

<Files\\1_11_18_003> - § 2 references coded [3.77% Coverage]

Reference 1 - 3.02% Coverage

tracking system for each individual resident as far as how they're doing compared to best practice guidelines for prescriptions and a list generated to that in comparison to the rest of the peers weather in a team or in the year in the clinic as a whole?

4. At least for me and I feel like we're pretty driven people as physicians and that I'm always are trying to excel and so if you see that you're doing not better than others then you want that do better.

Reference 2 - 0.75% Coverage

I think that depends on the reputation of the place and if they were a national leader I think it could. Give a 3.

<Files\\1_11_18_004> - § 1 reference coded [0.46% Coverage]

Reference 1 - 0.46% Coverage

5… you want to be around performing as well as your colleagues here.

<Files\\1_11_18_005> - § 4 references coded [8.26% Coverage]

References 1-2 - 3.55% Coverage

Used that in the ED for a fair number of cases..

So ED uses a lot of fosfomycin?

No some people use it..

Does it show up on a order set or something or how do u know it?

Just people talking on what can be used first line, the three I am aware of…situation like inpatient where someone was like started on something broader you know get started on like a ceftriaxone or something for an IV medication. If patient got better then we'll just continue on them on like a cephalosporin from the rationale of like they're on the Ceph and they got better on a cephalosporin you know we didn't have culture data before any abx or culture data to guide us so just continue and discharge them on cefdinir or something.

Reference 3 - 2.26% Coverage

Anywhere along the spectrum will be useful like part of annual report will be useful. We have meetings with our PDs and stuff where they go over things twice a year, I guess its similar for any doctor with like a performance review you know someone or whatever and it could be something included in there. Your RVU and Abx adherence can be put in there. People like numbers. People also get competitive I wanna get a higher number than what was last year.

Reference 4 - 2.45% Coverage

you mentioned about your prescribing practices what do you think other residents do like when you see their patients later on or you see people in the hospital, What agents do you think they prescribe for the most part?

I would guess somewhere but I don't know. I don't really do much of a frame of reference to work to what all other people been doing. On the floor, people get ceftriaxone in the ED and we continue like a cephalosporin on the floor and at discharge oral, but I don't know.

<Files\\1_18_18_006> - § 2 references coded [4.04% Coverage]

Reference 1 - 3.50% Coverage

personal thought process and rationale for antibiotic?

I think a lot of times we end up going with things like Cipro, bactrim and Keflex for for the I have seen first line used a lot of times and then I wouldn't use Bactrim if they had like they have like CKD, a recent AKI or something like that, and then looking at allergies and then seeing what they've been on before if it's like they've been treated already with one of them and were switch to another. Asking what they've had in the past for it.

Reference 2 - 0.55% Coverage

Often told that Cipro wasn't good in terms of the like resistance profile here

<Files\\3_6_18_008> - § 2 references coded [1.24% Coverage]

Reference 1 - 0.52% Coverage

Feel like that's just what attending we staff with tend to suggest

Reference 2 - 0.72% Coverage

But honestly a lot of the times we practice what our attending’s practice to a certain extent

<Files\\3_6_18_009> - § 1 reference coded [6.40% Coverage]

Reference 1 - 6.40% Coverage

So like benchmarking. U r in the real world practicing and you’re compared to other primary care doctors or clinics or practices based on best practice guidelines. do you feel like those should exist?

9--I mean of course I want everyone to practice evidence-based medicine and do then do the best things for their patience but and then what happens with that data you know,

M--what are some kind of complication u would see with a system like that?

9--I wouldn’t mind it I kind of like competition and quality improvement. I think it would help people like oh look what I'm doing maybe this isn't maybe I'm not I don't know what I'm doing or I could do this better. I'm just wondering how that would work in the community. Are you comparing people all over the state and and who is accessing that information, is it is it patients, are they looking and saying this guy doesn’t follow the guidelines. I just I don't know.

<Files\\Transcript_0001> - § 1 reference coded [1.62% Coverage]

Reference 1 - 1.62% Coverage

Any displays of public commitment by providers who will say they will follow guidelines or their clinic will follow guidelines? Do you that can be impactful?

001-2 out of 5

Perception of responsibility

<Files\\04_05_18_011> - § 3 references coded [6.64% Coverage]

References 1-2 - 2.87% Coverage

do you feel like it's the prescribers sort of responsibility to look up with this information regardless of what what the condition is do you feel like it's it's ultimately up to them to go out and find most uptodate information?

11--are you saying is it the prescribed responsibility to pick the right medicine ,because yes.

Reference 3 - 3.77% Coverage

have all of the patients that you're tracking and in particular location ever has whatever ICD-10 code to be funneled into your system and then have to have someone to look up what medication was prescribed and then see if that medication was prescribed was prescribed for the right reasons and prescription then

if it matches the best practice guidelines and then make an answer yes or no whether or not it was the best decision

<Files\\04_05_18_12> - § 2 references coded [5.80% Coverage]

Reference 1 - 3.94% Coverage

any comments on the tasking system that we use, any particular concerns with it, any room for improvement?

12—Its not horrible, it is what it is. It puts a lot of responsibility on the resident and it isn’t a bad thing .But you know I guess I would like maybe a bit of a more immediate kind of you no way to get in contact with patients, for example something really really important happens or really pressing matter maybe we can get like a spoke message. I've gotten spoke for like severe things but over on you know what we have is is fine it works.

Reference 2 - 1.86% Coverage

I definitely think you should incorporate outcomes with the standard, so for example hey this person did this and this outcome happened, first look at how their actions match upto standards . If it falls outside of then then what circumstances in the situation.

<Files\\04_05_18_13> - § 2 references coded [1.86% Coverage]

References 1-2 - 1.86% Coverage

should providers be held responsible for looking up the latest information on prescribing?

13--I mean yeah that's their responsibility to stay current with current current guidelines,

<Files\\05_14_18_15> - § 2 references coded [6.69% Coverage]

References 1-2 - 6.69% Coverage

How should primary care docs be informed? Is it upto pcp to stay uptodate or? Clinic leadership or Hospital leadership?

15--my belief is that the doctor is you know it's up to the doctors to make sure that they give the proper care and stay uotodate with the literature. unfortunately the case is that an individual or population that there are people that and I’d say a lot probably that are lacking so that it becomes more maybe if the individual can't fix problem clean environment that will help to fix the problem and then I think it comes down to the systems that in terms of costs and benefits and the system that doctors are part of that the doctors are presented as well, more people involved for distributing the resources the better, so if I was a primary care doc and if we had like the newsletter or like need like it's kind of updates updates or something like guidelines like these are summary of what the guidelines are. you know it's hard for doc to go through if the information is too long or maybe summarize and bring it out.

<Files\\1_11_18_002> - § 2 references coded [2.12% Coverage]

References 1-2 - 2.12% Coverage

Is there I guess a particular party you feel like should be ultimately responsible for looking up this guidelines individual providers, clinic leadership.

I think it’s the providers. If you are prescribing then you should look up for guidelines.

<Files\\1_11_18_003> - § 3 references coded [6.78% Coverage]

References 1-2 - 4.14% Coverage

Who do you think is ultimately responsible for looking up those guidelines? You think it should be individual providers that are prescribing it, should be clinic leadership and clinic staff? Do you think it should be certain societies and guidelines that published the guidelines in the first place, so they ultimately should be responsible for looking up information?

Ultimately the responsibility falls on the provider who is prescribing it, that they should know why they are prescribing the medicine and I think also that you know the attending’s that we staff with should also you know because they are responsible as well.

Reference 3 - 2.64% Coverage

Is there any particular adherence range that would be acceptable?

Social question let me see. I don't know what would be fair I would say that maybe if you were to

So let's say survey all physicians and have the median with the standard deviation I think we should fall within and you should not be outside two standard deviations of not following compared to other Physicians but has like a minimum.

<Files\\1_11_18_004> - § 3 references coded [3.33% Coverage]

References 1-2 - 2.62% Coverage

Do you feel like it's up to the primary care provider to be the one responsible for looking up? do you feel like Clinic leadership should have a role or your programs leadership to have a role as far as making sure residents are up-to-date on the most current prescribing guidelines for Abx ?

I think ultimately the prescribers are responsible but having a noon conference or didactics will be helpful

Reference 3 - 0.70% Coverage

Do you feel like there's an acceptable percentage adherence that the providers should adhere to?

Like 70%

<Files\\1_18_18_006> - § 1 reference coded [2.00% Coverage]

Reference 1 - 2.00% Coverage

do you think it's fair to have a provider evaluated based on adherence to those?

I guess It’s just a pretty common thing and we should treat it appropriately.

What would be an acceptable adherence rate in your mind if we were to do that?

I guess like the bare minimum be like 60 or 70%

<Files\\1_18_18_007> - § 2 references coded [1.41% Coverage]

References 1-2 - 1.41% Coverage

and as far as who's responsible maybe just the societies that are incharge maybe

So it's the responsibility of the physician to study information periodically or going on up to date..

<Files\\3_6_18_008> - § 1 reference coded [3.49% Coverage]

Reference 1 - 3.49% Coverage

Ultimately do u think it’s the primary care physician and the provider that should be the one that's responsible for looking up information as it gets updated throughout the years?

8- Yeah I think so and if associated with large entities it is useful to have lectures ,where they sort of just remind people or just let people know that there have been updates because I think people tend to just go back to the same resource which may be outdated.

<Files\\3_6_18_009> - § 2 references coded [1.48% Coverage]

References 1-2 - 1.48% Coverage

would u agree and say that primary care doctors or whoever's prescribed medicines are ultimately the ones that are responsible for keeping up-to-date and keeping on top of those guidelines information.?

9--yeah.

<Files\\3_6_18_010> - § 3 references coded [4.57% Coverage]

Reference 1 - 3.60% Coverage

I feel that we obviously should take responsibility for our patients and do the necessary work to make their care possible in the outpatient setting but we get a lot of tasks and so you know rotations and things like that it can be hard to keep up and then some of the little small tasks get buried in with some like really important ones and people are like without insulin or you know big things and then there's like paperwork that probably could wait a month and so just that is a little bit challenging that there's no way to kind of I guess there is a way to make it more urgent and Allscripts.

References 2-3 - 0.97% Coverage

Would you agree in saying that whoever is prescribing the meds, that the responsibility of staying uptodate with that information falls on that person?

10-Oh yes

<Files\\5_14_18_14> - § 3 references coded [4.09% Coverage]

References 1-3 - 4.09% Coverage

Is it a prescriber's responsibility to stay up to date on current information?

14--Yes

M--do you feel like there's any responsibility on the part of these either clinic leadership or hospital leadership on getting that information to the providers?

14-- I think yeah I mean there's some if for example we know that it tend to be higher rates of resistance whatever antibiotics in the community then I think that would definitely be something that should be told.

<Files\\Transcript_0001> - § 2 references coded [1.51% Coverage]

References 1-2 - 1.51% Coverage

Do u feel like PCP should be responsible to look up guidelines on their own?

001- I think UTI is a common problem that all IM docs should have familiarity with.

Perception of evaluation

<Files\\04_05_18_011> - § 3 references coded [7.10% Coverage]

References 1-2 - 4.73% Coverage

something called called Benchmarking. Do u feel like provider should be evaluated based on that?

11—Yes, good reflection of how well you're doing compared to other Physicians,helps you identify gaps in your knowledge helps you identify ways you can approve for better patient care. I think it's all this EPIC training is that you can actually see how many of your patients are actually meeting their blood pressure guidelines or meeting their A1C targets so this would be another easy one that's probably have at prescribing the correct abx

Reference 3 - 2.37% Coverage

Benchmarking…say you get a report saying this is your adherence rate to the best practice guidelines every month quarter years something like that if that was implemented you feel like that would be helpful or not in prescribing and improving prescribing practices?

11--4

<Files\\04_05_18_12> - § 5 references coded [11.56% Coverage]

References 1-2 - 5.63% Coverage

Do you feel like provider should be evaluated based on their adherence to best practice guidelines ?

12—Yes and No because its complicated. In an ideal world every person follows single guideline ,that can't really happen right because every patient is different every situation is different, you know for example if someone if the guidelines were to use nitrofurantoin for every case for every uncomplicated, for persons that had prior reaction to it , frequently get UTIs , history of renal stones or some other complicated factor then you look for more different treatment people. Medicine unfortunately is not like very like straightforward; it’s like like a stream it's always changing. Standards are good but I don't actually know if physicians should be evaluated based on standards.

Reference 3 - 1.85% Coverage

I definitely think you should incorporate outcomes with the standard, so for example hey this person did this and this outcome happened, first look at how their actions match upto standards . If it falls outside of then then what circumstances in the situation

Reference 4 - 2.00% Coverage

Say clinic implements provider bench marking. Every month you get a report saying is your adherence percentage to the best practice guidelines. If that was implemented you feel like that would be helpful or not?

12--Probably more so than not ,so they like it 3 or 4 ,probably four

Reference 5 - 2.07% Coverage

It will be a good way as you know doctors in training will kind of be accustomed to The Benchmark.I mean to the standards and kind of have that kind of ingrained in our heads instead of having to sometimes go off go off the cuff or a reflex. It will be a good thing be good for us to learn.

<Files\\04_05_18_13> - § 3 references coded [15.96% Coverage]

References 1-2 - 14.32% Coverage

should providers be evaluated based on their adherence to best practice guidelines?

13-- in terms of like residency or like compensation?

M—Not residency bit say in a large multicenter provider practice…say u get a report every month or every quarter every year within your practice as far as how often your prescribing within best practices compared to peers within practice or community?

13--Yeah I mean I don't think that's a bad idea you know there are a lot of patient factors that may not be I guess adequately assessed by whoever is looking at your you know whether or not you fit into guidelines so I could see how that it may not be like totally adequate but I think it be a good way for just like practice improvement.

M—Obviously there are also patient factors like our clinic we get diabetes foot exam QI for example where we get that this patient has diabetes but has no foot exam. My response is this patient has b/l BKA???? System has it has issues, do you feel like there's a way to easily capture that information as far as prescribing antibiotics if there's a you feel like there is a way to actually get that information?

13--I mean I guess providers could go through everyone who has like a UTI diagnosis code and create a report on if the patient has factors that would lead them to deviate from kind of standard prescribing guidelines but but yeah I don't know maybe I'll be cool to run some ways to try

Reference 3 - 1.64% Coverage

Provider benchmarking, so the idea that you are sort of compared to your peers as far as how often that your prescribing within best practice guidelines?

13—4 four

<Files\\05_14_18_15> - § 4 references coded [10.65% Coverage]

References 1-2 - 6.99% Coverage

So that's the interesting that was talking about this to doctor??? with a research proposal using benchmarks over all. What ends up happening is that I mean it it's hard to stratify patients to be the same way and that we use more healthcare dollars and more aggressive practices individuals that you know it's not even show African American, underserved populations , Medicaid patients overall, so the question how do you stratify? How do you stratify specifically for this? Could be a lot of ????to confirm that that is the case and you can find many reasons why I can't go against the grain. In general is going to be hard to try to stratify that specially on a resident level that as well. Do I think it is? I think that the way that we're going towards medicine is that that is going to be the case whether we like it or not and I think that's also to assess how residencies are doing well, I mean if you look at surgery, OB complications? How many procedure they do? and honestly comes down to numbers and not make medicine all about numbers but that's going to be the case going forward.

Reference 3 - 1.59% Coverage

First one is provider benchmarking.

15--I think so so I would say it's a four, it would cause me to reassess whether or not and look into the future be more cognizant of the fact that more often and then think back it do I really need to do this.

Reference 4 - 2.08% Coverage

I think benchmarking is a good idea to do cuz that helps people realize that maybe weren't doing it too much and that's kind of idea to that if you're not if you're not aware of what your mistakes are then you don't know that's what you don't knowing not knowing what you don't know is the biggest problem. So that will help.

<Files\\1_11_18_002> - § 3 references coded [9.51% Coverage]

Reference 1 - 4.02% Coverage

Do you feel like providers should be evaluated based on their adherence to certain guidelines or best practice guidelines?

That's a tricky question. So some of it definitely ,other parts of it ,so while we'd like to think or talk guideline to finding you know guideline based medicine lot of times when you talk with different attendings what they say is based more off of their own practice and not based off of guidelines for until those habits become learned habits.

References 2-3 - 5.49% Coverage

Please rate each one of them on a scale of 1 to 5. 1 being very unlikely to help improve prescribing practices and adherence and 5 being the most likely to help in the most helpful and the most likely to result in change?

First one is provider bench marking. A report created comparing one resident to another on how often they are prescribing withing best practice guidelines?

Hmmm…1

Any reason why?

You know those mid year evaluations that are send out and you like put on a time like this kind of this access with where you are in terms of the other residents.We had so many of those that most of the time I don't pay attention to.

<Files\\1_11_18_003> - § 5 references coded [5.45% Coverage]

References 1-2 - 0.99% Coverage

Do you think individual providers should be evaluated based on their adherence to best practice guidelines?

I would say yes, I would agree with that.

Reference 3 - 1.43% Coverage

How do you feel like those deviations should be captured, EMR, outside bodies that are reviewing in clinics anything like that?

I don't know cost too much, probably Emr would be the easiest way most cost-efficient.

References 4-5 - 3.03% Coverage

tracking system for each individual resident as far as how they're doing compared to best practice guidelines for prescriptions and a list generated to that in comparison to the rest of the peers weather in a team or in the year in the clinic as a whole?

4. At least for me and I feel like we're pretty driven people as physicians and that I'm always are trying to excel and so if you see that you're doing not better than others then you want that do better.

<Files\\1_11_18_004> - § 7 references coded [7.67% Coverage]

References 1-2 - 1.60% Coverage

Do you think individual providers should be evaluated based on their adherence to best practice guidelines if available for treating uncomplicated UTI?

I think so, if the provider has been on the guidelines and there are recourses then yes.

References 3-4 - 1.90% Coverage

Yeah if everything is set up if there is like there's been a lecture there's an app an easy way for them to look up the information if there is something feasible then I think so

Do you feel like there's an acceptable percentage adherence that the providers should adhere to?

Like 70%

Reference 5 - 2.08% Coverage

As far as enforcing how do you feel like a system could be set up for actually discovering that people aren't prescribing within best practice guidelines?

Maybe they can be a flag in the EMR for patients that are afraid to have UTI and someone do a chart review to check if there is adherence to clinical guidelines.

References 6-7 - 2.09% Coverage

First scenario is the provider benchmarking. Like a report is generated on every resident on how many times they are prescribing within best practice guidelines for these cases in comparison to other residents in their year in the clinic as a whole?

5… you want to be around performing as well as your colleagues here.

<Files\\1_11_18_005> - § 4 references coded [6.70% Coverage]

References 1-2 - 2.15% Coverage

So if guidelines did exist, u think providers should be evaluated by their adherence to them like if some providers do better job for UTI prescribing versus worst job?

More than evaluation I think feedback. Review like a 100 UTI prescriptions or whatever and we noticed that you've been giving out a lot of Cipro. Do you know are you familiar with the current guidelines and is there a reason for lot of Cipro you've been using?

Reference 3 - 2.26% Coverage

Anywhere along the spectrum will be useful like part of annual report will be useful. We have meetings with our PDs and stuff where they go over things twice a year, I guess its similar for any doctor with like a performance review you know someone or whatever and it could be something included in there. Your RVU and Abx adherence can be put in there. People like numbers. People also get competitive I wanna get a higher number than what was last year.

Reference 4 - 2.29% Coverage

On a scale of 1 to 5, 1 being unlikely to improve adherence and 5 being most likely to improve adherence. I'm going to go through a list of different interventions people done before so one is related to provider benchmarking, so it's kind of what we said here it's marking adherence to antibiotic prescribing for UTIs and then comparing it to your peers. You think that's so 1 is not useful, 5 is very useful how do you think that?

Like a 3

in the middle

<Files\\1_18_18_006> - § 3 references coded [3.10% Coverage]

References 1-2 - 2.02% Coverage

do you think it's fair to have a provider evaluated based on adherence to those?

I guess It’s just a pretty common thing and we should treat it appropriately.

What would be an acceptable adherence rate in your mind if we were to do that?

I guess like the bare minimum be like 60 or 70%

Reference 3 - 1.08% Coverage

What do you think provider benchmarking would be? Benchmarking to be reporting performance and adherence following guidelines compared to their peers.

4

<Files\\1_18_18_007> - § 5 references coded [12.03% Coverage]

References 1-2 - 9.08% Coverage

I don't know I mean, there's so much like more than just the like when providers make different decisions if there's so much more information that goes into them then guidelines so I sometimes get frustrated with you the things that are in place right now to evaluate , like my diabetes metrics for example…Did u have them too? Every 6 months we have to like see if we've documented a foot exam and if they have an upto date for the exam and if they have any like if their A1C is going the right direction but. Like there are some patients who are you know they have diabetes on paper but they're just like diabetes is not the thing that's going to affect their life for like they have so many other comorbidities I like I'm trying to get them to do their cancer screening and like like if you add diabetes and like adding a foot exam. it just it doesn't it's not the right way to care for the patient like you shouldn't be aggressive with her diabetes cuz it'll sacrifice care of other things and so those patients have higher diabetes numbers or they don't have a foot exam but I don't think it really affects their care but if you look at the numbers I'm like 50% compliant or something

References 3-4 - 1.56% Coverage

Yeah it sounds like you made me more in favor of using I guess evaluating guidelines if they if you were able to make exceptions or other things?

I am not against using guidelines just against evaluation

Reference 5 - 1.40% Coverage

You think provider benchmarking where you would get reports of your adherence and performance compared to your peers is a good way to measure kind of adherence to the guidelines?

4

<Files\\3_6_18_008> - § 4 references coded [6.18% Coverage]

References 1-2 - 3.57% Coverage

do you feel like provider should be evaluated by adherence to the guidelines of best practice guidelines if existed by say idsa or someone else as far as recommendations for prescriptions. Do you feel like provider should be evaluated based on those?

8- At face value, yes but I also think that there's a lot of sort of underlying clinical judgment that goes on which I don't know if that can be captured accurately, it depends on how the survey is designed.

Reference 3 - 1.76% Coverage

I mean I don't think there's a quick way to do it those are probably I mean using electronic records and surveys and sort of just trying to get a wide aggregate of data. So I think it's sort of the easiest way that's available.

Reference 4 - 0.85% Coverage

First one is bench marking, we talked about reporting your performance in clinic compared to your peers?

8--4

<Files\\3_6_18_009> - § 3 references coded [8.55% Coverage]

References 1-2 - 6.40% Coverage

So like benchmarking. U r in the real world practicing and you’re compared to other primary care doctors or clinics or practices based on best practice guidelines. do you feel like those should exist?

9--I mean of course I want everyone to practice evidence-based medicine and do then do the best things for their patience but and then what happens with that data you know,

M--what are some kind of complication u would see with a system like that?

9--I wouldn’t mind it I kind of like competition and quality improvement. I think it would help people like oh look what I'm doing maybe this isn't maybe I'm not I don't know what I'm doing or I could do this better. I'm just wondering how that would work in the community. Are you comparing people all over the state and and who is accessing that information, is it is it patients, are they looking and saying this guy doesn’t follow the guidelines. I just I don't know.

Reference 3 - 2.15% Coverage

First one is provider bench marking, say u get a report to how u r doing with your prescriptions and fitting in with the best guidelines compared to your peers ?do you feel like that sort of system would improve overall or not improve or ?

9--I'm going to give it a four, is more likely to to help a little bit

<Files\\3_6_18_010> - § 4 references coded [11.30% Coverage]

References 1-3 - 9.43% Coverage

M--so there's this concept of benchmarking it's the idea that primary care doctors should be evaluated based on their adherence to best practice guidelines , do you feel like that's something that should be done..?

10—In general yes, but I think sometimes it's challenging to do that because there are a lot of factors that play into it..

M—what kind of factors?

10—I mean just specifically for UTIs in general?

M--General

10--yeah if you're supposed to have so many people that get colonoscopies every year or something I mean there's so many people that don't want to do that and so kind of having a buy-in from the patient is is challenging and a lot of our patients are resource-limited and so some of the things that we want for them to do and maybe even they want to do for themselves or not able to accomplish for a number of reasons so I think you know especially our patient population that that those things could play into the fact that they can't get to the colo, cannot get a ride home and then of course there's also situations where your judgement has to play in and so that doesn't really those benchmarking tools are like calculate for the anomaly patients who may not for whatever reason you know not be able to fit into the guidelines and not every patient does so I mean that would be a downside is that you don't get really get credit for needing to strive on the guidelines which they are not the end-all and be-all but if there's a somewhat of a wiggle room then I think it's reasonable to make sure that people are not like going rogue and

Reference 4 - 1.86% Coverage

First one is bench marking,say a system is implemented where you're getting a report on your adherence to best practice guidelines compared to your peers and it within the clinic within the community-at-large, do u feel like that would improve adherence to prescribing practices ?

10--I think that would be 5.

<Files\\5_14_18_14> - § 2 references coded [12.18% Coverage]

References 1-2 - 12.18% Coverage

M--Do you feel like provider should be evaluated based on their adherence to said best practice guidelines if there are guidelines out there saying these are there I'm not recommended treatment duration for these are recommended antibiotics do you feel like provider should be evaluated based on adherence to those?

14--Probably as long as there is a way to account for situations where there might be other factors.

M—This concept of bench marking was bought up before, done for a lot of different things like for diabetes control A1C goals. do you feel like if there was a sort of situations where it say your prescribing outside of certain level of adherence rate to those? You feel like there's a certain level of acceptable level? Do u have a number for those?

14-- like the number that I think they should be at? like 80

M--so a couple of different things have been tried in the past as far as improving adherence to best practice guidelines for UTIs I'm going to go through a couple different things that people have tried before. So please rate the list I will be giving out on a scale of 1 to 5.

Provider benchmarking- Someone came in the clinic and said we're going to capture all of this data and give you a number saying comparing to your peers or other people like in the community or something like that saying you're recommended prescribing practices.

14—Probably a five.

<Files\\Transcript_0001> - § 4 references coded [5.90% Coverage]

References 1-2 - 2.42% Coverage

So I feel like the effort going into tracking that system will be greater than the benefit u could get by changing behaviors but I am not opposed to that, but you can give a quick blurb to someone that hey this is something u can work on, its reasonable.

References 3-4 - 3.47% Coverage

On a scale to 1 to 5, 1 being most unlikely to improve adherence and 3 being neutral and 5 being the most likely to improve adherence. so just a couple of different interventions tried in the past/ proposed in the past to improve adherence to guidelines.

First one is provider benchmarking. Comparing provider with his or her peers on adherence to guidelines.

001- 3

**PROCESS IMPROVEMENT AND NON-PHYSICIAN PRESCRIBING**

Tasking process and its improvement

<Files\\04_05_18_12> - § 2 references coded [4.09% Coverage]

Reference 1 - 0.95% Coverage

so if they call I guess someone an operator or whoever answers the phone will task me and I'll get that task and I'll call the patient

Reference 2 - 3.14% Coverage

Its not horrible, it is what it is. It puts a lot of responsibility on the resident and it isn’t a bad thing .But you know I guess I would like maybe a bit of a more immediate kind of you no way to get in contact with patients, for example something really really important happens or really pressing matter maybe we can get like a spoke message. I've gotten spoke for like severe things but over on you know what we have is is fine it works.

<Files\\04_05_18_13> - § 1 reference coded [3.74% Coverage]

Reference 1 - 3.74% Coverage

I mean sometimes I get tasks that are like glaring things like a patient complaining of suicidal ideation, so it was super inappropriate. And I don't know I mean sometimes they like page for things that aren't that urgent and it's a little bit frustrating, but I don't know I think in general it's an okay system for communicating getting messages to us from our patients.

<Files\\05_14_18_15> - § 1 reference coded [4.99% Coverage]

Reference 1 - 4.99% Coverage

I never thought of that that I guess what ends up happening is that by the time that the patient calls, the secretary whatever it is the secretary notify the doctor, doctor finds out I mean we can be anywhere from three to seven days that's kind of you know concerning for person who has urinary tract infection progression to disease but many people don't have aggression??? In general I think could improve that. Yeah I don't I don't know that off the top of my head right now.

M-- are there any sort of combinations of symptoms that would make you more likely to treat a suspected case of urinary tract infection versus not treating? So we mention the other symptoms, what sort of symptoms are you looking for that would make you more likely to treat urinary tract infections??

<Files\\1_11_18_002> - § 2 references coded [11.31% Coverage]

Reference 1 - 9.92% Coverage

I normally get a phone call, I almost never see someone come in for a UTI complaint.

Can you give like a percentage wise on many times task versus…

99% patient calls/tasks

How does that normally get routed to you? If you can walk us though the process.

So the patient calls in, the secretary picks up the phone call will send me a task saying patient wants to talk to me that's about it. I will call the patient back as soon as I can. We talk about the complaints, see if it's warranted that she comes in or we handle it over the phone.

Do you feel like you get enough information in the typical just blurb in the task about the …

No Never

So you always call the patient back?

Yes

Do you sometimes call antibiotics over the phone without bring them in for an appointment.

Yes

How Frequently?

About 50% of the time, usually its pretty uncomplicated just urinary frequency and dysuria in an uncomplicated patient. If it's at all complicated or if they have fevers and chills or I think there is something else going on or if it sounds like they've been having urinary frequency and dysuria for far longer than appropriate then I make them come in.

Reference 2 - 1.38% Coverage

Anything that you think can be done to improve the task process?

Probably having someone with some medical background answering the phone calls would help a lot.

<Files\\1_11_18_003> - § 2 references coded [4.41% Coverage]

Reference 1 - 1.29% Coverage

Do you get tasks about urinary symptoms or urinary complaints?

I rarely do, I've had a few on patients that have like recurring UTIs and then I'll call in and then it’s not really uncomplicated.

Reference 2 - 3.12% Coverage

There are a lot of things that could be done. I think frequently I'll get tasks to where they don't I don't know who tasked me, a number that I can reach the person that tasked me out that would be helpful. When they do task me frequently the number that they provide is incorrect so if there's some way that we can have them verify a phone number and make sure what they put in there is correct. Maybe get two phone numbers. What else? I can’t think of any more right now.

<Files\\1_11_18_004> - § 1 reference coded [2.34% Coverage]

Reference 1 - 2.34% Coverage

I think the Clinic is having things in it and is having things together to help things out like having a triage nurse would be helpful, to not get task about somebody having like chest pain with shortness of breath lightheadedness and dizziness I given the task box like I think that's something that the patient should be directly directed immediately to ED.

<Files\\1_11_18_005> - § 2 references coded [6.25% Coverage]

Reference 1 - 3.77% Coverage

Task is like patient called sometimes it says she think she has a UTI please return call. Usually its very little information

so what do you do with that information if they don't give you any information you obviously have to call?

You have to call the patient back as you cant rely on the information in the msg and see what they're talking about what their symptoms

So u call back if they say they have a UTI, but what about if you're on the ICU or tough rotations and you're super busy and you always call back ?

I would not prescribe anything without talking to them in person. We have team members are supposed to be keeping an eye on your tasks or something up there is something really urgent but I wouldn't prescribe without talking to them.

Reference 2 - 2.48% Coverage

From my perspective not just for UTIs but tasks in general would be good if there was like that nurse practitioner or float resident or somebody whose job it was a kind of handle these you know there's not too much you got for you not to get too involved in the patient history to figure out what to do in this situation you know it's relatively clear cut what you should do who would you know me having an to get involved, notify me that patient had UTI, we did this, follow-up needs to be done.

<Files\\3_6_18_008> - § 2 references coded [2.29% Coverage]

Reference 1 - 2.05% Coverage

Sometimes its just patient wants to talk to you, sometimes it's patient has a patient thinks they need antibiotics, sometimes it's patient has urinary symptoms something like that. usually it's not specific so we do have to call them back and know what's going on.

Reference 2 - 0.25% Coverage

we need actual nurses to triage

<Files\\3_6_18_009> - § 1 reference coded [4.15% Coverage]

Reference 1 - 4.15% Coverage

relayed to you. do you have any thoughts about the task process ? how can be improved?

9-- well I don’t think u can ever take whatever is said in that task you know word for word. you always got to call to clarify. I would never you know if if somebody if I got a task that said hey this patient thinks he has UTI and they need antibiotics I wouldn't just write for antibiotics ,that I would have I would really want to talk to the patient first. I mean I don't know how they could really improve that process somebody's got to answer the phone it's not going to be a physician. you know I don't know.

<Files\\3_6_18_010> - § 2 references coded [8.32% Coverage]

Reference 1 - 3.76% Coverage

So its challenging I mean I feel that we obviously should take responsibility for our patients and do the necessary work to make their care possible in the outpatient setting but we get a lot of tasks and so you know rotations and things like that it can be hard to keep up and then some of the little small tasks get buried in with some like really important ones and people are like without insulin or you know big things and then there's like paperwork that probably could wait a month and so just that is a little bit challenging that there's no way to kind of I guess there is a way to make it more urgent and Allscripts.

Reference 2 - 4.56% Coverage

well it could be I know it's not a novel idea but you know kind of weeding out some of those tasks that could be done by others that don't require a physician or resident to do would help to offload the load and make let us focus on the things that do need to be done by a physician but I know that's we have a ton of residents and ton of patients so I realize the feasibility of that probably probably hard but yeah I mean or if there was a triage nurse or someone that and I would be actually happening or discussion might be happening of having someone in there that is you know could take this and say hey this is Marvin and admit thing that this person can you talk with him, read through their chart that sort of having someone to be able to feel that.

<Files\\5_14_18_14> - § 1 reference coded [4.01% Coverage]

Reference 1 - 4.01% Coverage

I guess like one thing that I've noticed is like the people who work in the call centers that are taking the calls don't always accurately represent the nature of them so I could get a task that was like patient complaints of dysuria but then on calling back and it's like something different ,so I think maybe if people or some people had like more of a medical assistant background that might be kind of nice yeah, I think that would probably be my major.

<Files\\Transcript_0001> - § 1 reference coded [6.22% Coverage]

Reference 1 - 6.22% Coverage

ok, how are phone calls typically handled at the clinic, if a patient calls in to the clinic with a complaint how does that get routed?

001- goes first to a nurse who will triage the call and then ….advice can ask whatever resident or attending there is in the clinic…………………………… channel the calls and recommend that they come into the clinic or not or forward request if the patient has any other question to answer.

M-given the patient population do u ever get phone calls which sound like UTI just based on the complaints?

001- you know I don’t think that in my clinic I actually have ever had someone just call in and complain of urinary tract infection.

Communication to provider

<Files\\04_05_18_011> - § 1 reference coded [4.49% Coverage]

Reference 1 - 4.49% Coverage

how do you typically get patients that complain of symptoms worrisome for UTI did they mostly come in for a clinic visit you get a phone call to get a task like how what is the most common way these patients come to u?

11—some don’t come primarily for UTI complaint, usually they're here for something else and tell by the way I have this too.

M—what about phone call where patients are asking for antibiotics ?

11- Patients I get usually have chronic catheterization and those are the only phone calls I get.

<Files\\04_05_18_12> - § 2 references coded [7.49% Coverage]

Reference 1 - 3.55% Coverage

this patient came into the clinic just as an appointment, just a regular Clinic appointment, do you ever get cases for patients don't come in. as in tasks, phone calls anything like that?

12--I have not

M--if one of your patients suddenly has urinary symptoms and they decided to call. What is the actual process for getting that that information to you?

12-- so if they call I guess someone an operator or whoever answers the phone will task me and I'll get that task and I'll call the patient

Reference 2 - 3.94% Coverage

any comments on the tasking system that we use, any particular concerns with it, any room for improvement?

12—Its not horrible, it is what it is. It puts a lot of responsibility on the resident and it isn’t a bad thing .But you know I guess I would like maybe a bit of a more immediate kind of you no way to get in contact with patients, for example something really really important happens or really pressing matter maybe we can get like a spoke message. I've gotten spoke for like severe things but over on you know what we have is is fine it works.

<Files\\04_05_18_13> - § 1 reference coded [5.55% Coverage]

Reference 1 - 5.55% Coverage

I mean there's a call with them yeah

M—Have u ever had tasks or calls saying I have a UTI or I'm having dysuria?.

13—I don't have had a task where a patient's been like I have a UTI but I got a recent one that was like where patient was complaining of increased urinary frequency and I spoke to her it sounded of like she had dysuria too.

M—It's a case by case decision but in that use would u prescribe abx over the phone?

13—I never do, she went in for a Urgent Care visit and she did have UTI and I actually wanted to see what was in her urine.

<Files\\05_14_18_15> - § 1 reference coded [3.91% Coverage]

Reference 1 - 3.91% Coverage

- honestly speaking through the office, communications by phone is kind of lacking compared to most and most of my patients come into clinic unless its an emergency, but I have gotten phone calls about UTI, a lot of times especially very recently I would say you know and they haven't had a recent urinary tract infections going for an overall that I would say you know maybe we can treat this over the phone ,it depends on if there are like at high risk for developing resistance then I am like hey you need to come back and you don't have to be seen over cuz I can do all the testing know that they're otherwise

<Files\\1_11_18_002> - § 3 references coded [7.24% Coverage]

Reference 1 - 3.47% Coverage

so that case was a patient that came into Clinic, do you feel like that's typical for how you hear about urinary tract infections or do you sometimes get a task or a phone call from a patient or what is the normal route you get.

I normally get a phone call, I almost never see someone come in for a UTI complaint.

Can you give like a percentage wise on many times task versus…

99% patient calls/tasks

Reference 2 - 2.44% Coverage

So the patient calls in, the secretary picks up the phone call will send me a task saying patient wants to talk to me that's about it. I will call the patient back as soon as I can. We talk about the complaints, see if it's warranted that she comes in or we handle it over the phone.

Reference 3 - 1.33% Coverage

Have you ever had a problem that you get a task complaining of dysuria and you call the patient back and never get hold of them?

Yes

Frequently?

Yes

<Files\\1_11_18_003> - § 5 references coded [11.17% Coverage]

Reference 1 - 1.77% Coverage

I think that most times in my patients it is like an additional complaint. Very infrequently will have someone that just comes in for urinary symptoms it's usually they come in for multiple other complaints and then I’m also having dysuria and then we check the urine.

Reference 2 - 1.30% Coverage

Do you get tasks about urinary symptoms or urinary complaints?

I rarely do, I've had a few on patients that have like recurring UTIs and then I'll call in and then it’s not really uncomplicated.

Reference 3 - 1.56% Coverage

if the patient that I know that has had UTIs that have been confirmed and then you know they know the symptoms are very similar I feel I feel pretty comfortable if they call in and say I’m having the same symptoms then I would treat them.

Reference 4 - 1.37% Coverage

Say you do get a call with a task like this for urinary symptoms like you mentioned do you always call them back do you ever just prescribe antibiotics without talking to them?

I have not, I usually callback

Reference 5 - 5.17% Coverage

What if there was an instance where someone left you a task thing complaining of urinary symptoms and you couldn't get ahold of them and you can call him back? What would you do?

Usually in that case, are we receiving that like I know their medical history or just getting a task

Say you know them seen them for UTI before and call you for similar complaints after a couple of phone calls still can't get in touch with them?

I think if someone I knew and that I trusted I would still feel comfortable writing a prescription. It was someone that I wasn't sure ,was concerned that they could have other complications then would probably leave a note that says if they call again please have them provide a phone number and time where I can reach them and then try to get on that way.

<Files\\1_11_18_004> - § 2 references coded [5.31% Coverage]

Reference 1 - 2.66% Coverage

if you were to get a task that sounded like a urinary tract infection would you always try and call the patient back?

Yes, call them back and ask more about their symptoms. If they're pretty healthy and don't have a lot of complaints I might I might go ahead and start them something empirically but ideally I would like to get like a UA and some kind of documentation before I start someone on antibiotics.

Reference 2 - 2.65% Coverage

If you had a situation where someone calls leaves you a task seeing this patient complaining of dysuria and things like that and you try and call them back and can't get a hold of them. What do u do?

I will just keep calling back I mean I feel like if they're symptoms of bothersome they should be able to answer the phone or try to get back in contact with me so we can talk a little bit more about it.

<Files\\1_11_18_005> - § 2 references coded [3.23% Coverage]

Reference 1 - 0.63% Coverage

Task is like patient called sometimes it says she think she has a UTI please return call. Usually its very little information

Reference 2 - 2.61% Coverage

You have to call the patient back as you cant rely on the information in the msg and see what they're talking about what their symptoms

So u call back if they say they have a UTI, but what about if you're on the ICU or tough rotations and you're super busy and you always call back ?

I would not prescribe anything without talking to them in person. We have team members are supposed to be keeping an eye on your tasks or something up there is something really urgent but I wouldn't prescribe without talking to them.

<Files\\1_18_18_006> - § 3 references coded [8.58% Coverage]

Reference 1 - 1.97% Coverage

people just come in and then they have the symptoms and then we just work it out from there I've had people call and say oh someone else thought I might have had a urine urinary tract infection, ok so we'll just order UA and then you can come in and get it. Usually those two Avenues.

Reference 2 - 2.31% Coverage

do you always call them back or do you sometimes get enough history that you think that UTI ….

I usually call them back…

so you'll call him back and clarify their symptoms ?

And then have them come up here for the UA and then I'll wait for that cuz the results always come back to us usually as the task we can decide what to do.

Reference 3 - 4.29% Coverage

if you can't get ahold of the patient do you ever give up or what do you do in that circumstance do you just tell him just try to keep getting them in for the test thing or what happens then?

I haven’t had the case where I couldn’t completely couldn't reach them. Hypothetically if I don’t reach if I couldn't reach him the first time you should try to like call a couple times in the same like within a couple minutes and I'll try to make it a completely different time of day like around the next day or something and then if after like a few different attempts I might just be like well I'll try to leave a message.

<Files\\1_18_18_007> - § 2 references coded [7.94% Coverage]

Reference 1 - 6.45% Coverage

Can you walk me through in the clinic here how you find out when a patient has a UTI? Do they present to you or message or phone call or what?

You order UA and you get the results in your box should be like a couple hours later and then I think 3 days maybe 24 hours later you get initial culture results and then like then you get the final culture results later and then.

Do you always get urine testing on patients that you're worried about for UTI?

Yes mostly?

So if they call and say they have symptoms for a UTI , do u ask them to come to Clinic to be seen or just come in for to drop off a urine specimen or you sometimes if they if you just get a message only is that sufficient or do you feel like you to call him back and say hey let me clarify your symptoms and you evaluate them.?

I have rarely done phone prescriptions for UTIs.

Reference 2 - 1.49% Coverage

So if they call and u don’t have a clinic coming up then u have them seen by ambulatory resident that is on or something?

Yes if it’s a low risk situation then would just prescribe over the phone

<Files\\3_6_18_008> - § 3 references coded [8.49% Coverage]

Reference 1 - 1.67% Coverage

how do you typically find a patient like this do they come in to Clinic with this complaint you get a call with a task like how does this problem present itself?

8-probably 50-50 task,routine visit ,add on visit

Reference 2 - 5.19% Coverage

Sometimes its just patient wants to talk to you, sometimes it's patient has a patient thinks they need antibiotics, sometimes it's patient has urinary symptoms something like that. usually it's not specific so we do have to call them back and know what's going on.

M- do you ever treat over the phone if someone says I have these symptoms would you ever prescribed antibiotics over the phone without having them coming to Clinic?

8- I have maybe once or twice and this was the patient who had a sort of Prior urinary tract infections but not recently but had a prior urine samples in our database with bacterial sensitivities and they were sort of reluctant to come in

Reference 3 - 1.63% Coverage

has there ever been instances when you get a task complaining about something either generic or something related to UTI where you can't get back in touch with the patient and are unable to get a history?

8-No

<Files\\3_6_18_009> - § 2 references coded [6.27% Coverage]

Reference 1 - 1.44% Coverage

so I can't say that I'm usually I have not been really called with patients saying I think I have a UTI. I have a couple patients come in and say I have UTI since they have UTI before and request trt for it.

Reference 2 - 4.83% Coverage

Just talking about the tasking process in general and how patients are are there complaints are relayed to you. do you have any thoughts about the task process ? how can be improved?

9-- well I don’t think u can ever take whatever is said in that task you know word for word. you always got to call to clarify. I would never you know if if somebody if I got a task that said hey this patient thinks he has UTI and they need antibiotics I wouldn't just write for antibiotics ,that I would have I would really want to talk to the patient first. I mean I don't know how they could really improve that process somebody's got to answer the phone it's not going to be a physician. you know I don't know.

<Files\\3_6_18_010> - § 2 references coded [5.84% Coverage]

Reference 1 - 1.72% Coverage

I haven't had that exact situation of come up but I could imagine a young person like that would probably be calling, maybe they if they had time would make an appointment but if I was in their shoes I wouldn't have had a hesitation calling my position and if insisted I would come in.

Reference 2 - 4.12% Coverage

would you ever consider treating so say if he's in like these called in and say they had urinary symptoms you ever consider treating over the phone without having them come in.?

10--I guess if I knew the patient really well and kind of felt like they were reliable someone that was going to be ,someone I could keep in touch with and talk about their symptoms I would consider it ,if it was someone that had a lot of other well I guess in this situation she doesn't have a lot of other medical problems so I probably would feel more comfortable with her managing it on phone,if it was someone I knew, but if I seen them one time 2 years ago then that would be less likely to do that.

<Files\\5_14_18_14> - § 2 references coded [4.89% Coverage]

Reference 1 - 2.38% Coverage

I'm thinking of when I was in amjar if I specifically saw someone I mean I can't think of a specific scenario but like it wouldn't be.

M—Do u ever get like tasks thru the phone system about urinary symptoms ?

14--I think like maybe less frequently than one might guess.

Reference 2 - 2.51% Coverage

I could get a task that was like patient complaints of dysuria but then on calling back and it's like something different ,so I think maybe if people or some people had like more of a medical assistant background that might be kind of nice yeah, I think that would probably be my major.

<Files\\Transcript_0001> - § 3 references coded [6.71% Coverage]

Reference 1 - 3.92% Coverage

ok, how are phone calls typically handled at the clinic, if a patient calls in to the clinic with a complaint how does that get routed?

001- goes first to a nurse who will triage the call and then ….advice can ask whatever resident or attending there is in the clinic…………………………… channel the calls and recommend that they come into the clinic or not or forward request if the patient has any other question to answer.

Reference 2 - 2.24% Coverage

given the patient population do u ever get phone calls which sound like UTI just based on the complaints?

001- you know I don’t think that in my clinic I actually have ever had someone just call in and complain of urinary tract infection.

Reference 3 - 0.54% Coverage

Hmm, ya people called that they were having cellulitis….

Access to care

<Files\\04_05_18_011> - § 1 reference coded [4.49% Coverage]

Reference 1 - 4.49% Coverage

how do you typically get patients that complain of symptoms worrisome for UTI did they mostly come in for a clinic visit you get a phone call to get a task like how what is the most common way these patients come to u?

11—some don’t come primarily for UTI complaint, usually they're here for something else and tell by the way I have this too.

M—what about phone call where patients are asking for antibiotics ?

11- Patients I get usually have chronic catheterization and those are the only phone calls I get.

<Files\\04_05_18_12> - § 2 references coded [5.82% Coverage]

Reference 1 - 3.55% Coverage

this patient came into the clinic just as an appointment, just a regular Clinic appointment, do you ever get cases for patients don't come in. as in tasks, phone calls anything like that?

12--I have not

M--if one of your patients suddenly has urinary symptoms and they decided to call. What is the actual process for getting that that information to you?

12-- so if they call I guess someone an operator or whoever answers the phone will task me and I'll get that task and I'll call the patient

Reference 2 - 2.27% Coverage

Would you ever consider treating over the phone without a clinic appointment?

12--To be honest I think I would at least like her to come in and seen by amjar, but if she you know was really hesitant, you know whatever reason couldn't make it ,I would feel comfortable prescribing and then having her at least follow up

<Files\\04_05_18_13> - § 1 reference coded [5.55% Coverage]

Reference 1 - 5.55% Coverage

I mean there's a call with them yeah

M—Have u ever had tasks or calls saying I have a UTI or I'm having dysuria?.

13—I don't have had a task where a patient's been like I have a UTI but I got a recent one that was like where patient was complaining of increased urinary frequency and I spoke to her it sounded of like she had dysuria too.

M—It's a case by case decision but in that use would u prescribe abx over the phone?

13—I never do, she went in for a Urgent Care visit and she did have UTI and I actually wanted to see what was in her urine.

<Files\\05_14_18_15> - § 1 reference coded [3.91% Coverage]

Reference 1 - 3.91% Coverage

- honestly speaking through the office, communications by phone is kind of lacking compared to most and most of my patients come into clinic unless its an emergency, but I have gotten phone calls about UTI, a lot of times especially very recently I would say you know and they haven't had a recent urinary tract infections going for an overall that I would say you know maybe we can treat this over the phone ,it depends on if there are like at high risk for developing resistance then I am like hey you need to come back and you don't have to be seen over cuz I can do all the testing know that they're otherwise

<Files\\1_11_18_002> - § 4 references coded [9.91% Coverage]

Reference 1 - 3.47% Coverage

so that case was a patient that came into Clinic, do you feel like that's typical for how you hear about urinary tract infections or do you sometimes get a task or a phone call from a patient or what is the normal route you get.

I normally get a phone call, I almost never see someone come in for a UTI complaint.

Can you give like a percentage wise on many times task versus…

99% patient calls/tasks

Reference 2 - 2.44% Coverage

So the patient calls in, the secretary picks up the phone call will send me a task saying patient wants to talk to me that's about it. I will call the patient back as soon as I can. We talk about the complaints, see if it's warranted that she comes in or we handle it over the phone.

Reference 3 - 1.33% Coverage

Have you ever had a problem that you get a task complaining of dysuria and you call the patient back and never get hold of them?

Yes

Frequently?

Yes

Reference 4 - 2.66% Coverage

Anything that you think can be done to improve the task process?

Probably having someone with some medical background answering the phone calls would help a lot.

Who currently answers the call and triages?

So I actually don't know, I think it's the secretary or the checkout clerks who answer phone calls.

<Files\\1_11_18_003> - § 4 references coded [9.61% Coverage]

Reference 1 - 1.77% Coverage

I think that most times in my patients it is like an additional complaint. Very infrequently will have someone that just comes in for urinary symptoms it's usually they come in for multiple other complaints and then I’m also having dysuria and then we check the urine.

Reference 2 - 1.30% Coverage

Do you get tasks about urinary symptoms or urinary complaints?

I rarely do, I've had a few on patients that have like recurring UTIs and then I'll call in and then it’s not really uncomplicated.

Reference 3 - 1.37% Coverage

Say you do get a call with a task like this for urinary symptoms like you mentioned do you always call them back do you ever just prescribe antibiotics without talking to them?

I have not, I usually callback

Reference 4 - 5.17% Coverage

What if there was an instance where someone left you a task thing complaining of urinary symptoms and you couldn't get ahold of them and you can call him back? What would you do?

Usually in that case, are we receiving that like I know their medical history or just getting a task

Say you know them seen them for UTI before and call you for similar complaints after a couple of phone calls still can't get in touch with them?

I think if someone I knew and that I trusted I would still feel comfortable writing a prescription. It was someone that I wasn't sure ,was concerned that they could have other complications then would probably leave a note that says if they call again please have them provide a phone number and time where I can reach them and then try to get on that way.

<Files\\1_11_18_004> - § 3 references coded [6.92% Coverage]

Reference 1 - 1.87% Coverage

Can you walk me through how you find a patient? Is it tasks or phone calls or patients come in? Kind of walk me through the different ways that a patient with urinary tract infection will find their way to you?

Probably mostly Clinic appointments and I've gotten a task for one patient.

Reference 2 - 2.39% Coverage

Is there any case where you would get a UA versus not get a UA and urine studies for patients that call you?

If they if they have a means to get to the get here in to give me those samples I will get them done by like some of our patients can barely get here if they had no way to get here then maybe I'll be okay with that. But I would prefer some test before meds.

Reference 3 - 2.65% Coverage

If you had a situation where someone calls leaves you a task seeing this patient complaining of dysuria and things like that and you try and call them back and can't get a hold of them. What do u do?

I will just keep calling back I mean I feel like if they're symptoms of bothersome they should be able to answer the phone or try to get back in contact with me so we can talk a little bit more about it.

<Files\\1_11_18_005> - § 3 references coded [5.93% Coverage]

Reference 1 - 0.63% Coverage

Task is like patient called sometimes it says she think she has a UTI please return call. Usually its very little information

Reference 2 - 2.61% Coverage

You have to call the patient back as you cant rely on the information in the msg and see what they're talking about what their symptoms

So u call back if they say they have a UTI, but what about if you're on the ICU or tough rotations and you're super busy and you always call back ?

I would not prescribe anything without talking to them in person. We have team members are supposed to be keeping an eye on your tasks or something up there is something really urgent but I wouldn't prescribe without talking to them.

Reference 3 - 2.70% Coverage

already at home than I usually trt them over the phone. I would not ask them to come to the office to a leave a UA. Would just trt over the phone with Bactrim or look for prior culture data and use that.

Would you want a culture? Would u tell I will treat over phone but want UA/culture?

Initially nothing but symptoms same after two days them have to come in and get labs and see what's actually going on and if I don't think they have a UTI then would ask them to make an appt with urgent care or go to the ED for concerning symptoms.

<Files\\1_18_18_006> - § 2 references coded [6.27% Coverage]

Reference 1 - 1.97% Coverage

people just come in and then they have the symptoms and then we just work it out from there I've had people call and say oh someone else thought I might have had a urine urinary tract infection, ok so we'll just order UA and then you can come in and get it. Usually those two Avenues.

Reference 2 - 4.29% Coverage

if you can't get ahold of the patient do you ever give up or what do you do in that circumstance do you just tell him just try to keep getting them in for the test thing or what happens then?

I haven’t had the case where I couldn’t completely couldn't reach them. Hypothetically if I don’t reach if I couldn't reach him the first time you should try to like call a couple times in the same like within a couple minutes and I'll try to make it a completely different time of day like around the next day or something and then if after like a few different attempts I might just be like well I'll try to leave a message.

<Files\\1_18_18_007> - § 2 references coded [7.94% Coverage]

Reference 1 - 6.45% Coverage

Can you walk me through in the clinic here how you find out when a patient has a UTI? Do they present to you or message or phone call or what?

You order UA and you get the results in your box should be like a couple hours later and then I think 3 days maybe 24 hours later you get initial culture results and then like then you get the final culture results later and then.

Do you always get urine testing on patients that you're worried about for UTI?

Yes mostly?

So if they call and say they have symptoms for a UTI , do u ask them to come to Clinic to be seen or just come in for to drop off a urine specimen or you sometimes if they if you just get a message only is that sufficient or do you feel like you to call him back and say hey let me clarify your symptoms and you evaluate them.?

I have rarely done phone prescriptions for UTIs.

Reference 2 - 1.49% Coverage

So if they call and u don’t have a clinic coming up then u have them seen by ambulatory resident that is on or something?

Yes if it’s a low risk situation then would just prescribe over the phone

<Files\\3_6_18_008> - § 1 reference coded [1.67% Coverage]

Reference 1 - 1.67% Coverage

how do you typically find a patient like this do they come in to Clinic with this complaint you get a call with a task like how does this problem present itself?

8-probably 50-50 task,routine visit ,add on visit

<Files\\3_6_18_009> - § 2 references coded [6.27% Coverage]

Reference 1 - 1.44% Coverage

so I can't say that I'm usually I have not been really called with patients saying I think I have a UTI. I have a couple patients come in and say I have UTI since they have UTI before and request trt for it.

Reference 2 - 4.83% Coverage

Just talking about the tasking process in general and how patients are are there complaints are relayed to you. do you have any thoughts about the task process ? how can be improved?

9-- well I don’t think u can ever take whatever is said in that task you know word for word. you always got to call to clarify. I would never you know if if somebody if I got a task that said hey this patient thinks he has UTI and they need antibiotics I wouldn't just write for antibiotics ,that I would have I would really want to talk to the patient first. I mean I don't know how they could really improve that process somebody's got to answer the phone it's not going to be a physician. you know I don't know.

<Files\\3_6_18_010> - § 3 references coded [9.48% Coverage]

Reference 1 - 1.72% Coverage

I haven't had that exact situation of come up but I could imagine a young person like that would probably be calling, maybe they if they had time would make an appointment but if I was in their shoes I wouldn't have had a hesitation calling my position and if insisted I would come in.

Reference 2 - 4.12% Coverage

would you ever consider treating so say if he's in like these called in and say they had urinary symptoms you ever consider treating over the phone without having them come in.?

10--I guess if I knew the patient really well and kind of felt like they were reliable someone that was going to be ,someone I could keep in touch with and talk about their symptoms I would consider it ,if it was someone that had a lot of other well I guess in this situation she doesn't have a lot of other medical problems so I probably would feel more comfortable with her managing it on phone,if it was someone I knew, but if I seen them one time 2 years ago then that would be less likely to do that.

Reference 3 - 3.64% Coverage

a lot of our patients are resource-limited and so some of the things that we want for them to do and maybe even they want to do for themselves or not able to accomplish for a number of reasons so I think you know especially our patient population that that those things could play into the fact that they can't get to the colo, cannot get a ride home and then of course there's also situations where your judgement has to play in and so that doesn't really those benchmarking tools are like calculate for the anomaly patients who may not for whatever reason you know not be able to fit into the guidelines

<Files\\5_14_18_14> - § 1 reference coded [2.38% Coverage]

Reference 1 - 2.38% Coverage

I'm thinking of when I was in amjar if I specifically saw someone I mean I can't think of a specific scenario but like it wouldn't be.

M—Do u ever get like tasks thru the phone system about urinary symptoms ?

14--I think like maybe less frequently than one might guess.

<Files\\Transcript_0001> - § 2 references coded [2.92% Coverage]

Reference 1 - 2.38% Coverage

ok, based on your clinic how do these sorts of patients typically present? Do you get phone call, do they come in, and do u get tasks, notification from clinic staff about these patients and complaints?

001-they usually come in, I am at the VA clinic

Reference 2 - 0.54% Coverage

Hmm, ya people called that they were having cellulitis….

Provider Codes

**CLINICAL APPROACH TO UTI**

Interpretation of test

<Files\\01_10_19_014> - § 2 references coded [17.04% Coverage]

Reference 1 - 6.64% Coverage

How will you handle the case?

014: With somebody like that, we do the Udip in the office. If there’s anything that seems generally positive, leukocytes or nitrates or anything like that, we’ll go ahead and treat them, if it’s uncomplicated and they don’t do it all the time, I usually do 3-5 days of macrobid. It kinda depends, I stick more towards 5. It might be overkill, but since they usually aren’t in my setting, we’re primary care but we also do walk in, and we’re in a tourist area so we get people we’re never gonna see again. We kinda wanna send them off adequately set up, and then we’ll send that urine for culture. FOr everybody that has insurance we send the culture, sometimes if they don’t we give them the choice. Otherwise we just go with it and tell them to follow up if they don’t have resolution of symptoms.

Reference 2 - 10.40% Coverage

Patient factors, symptoms, age, sex, etc

014: I think I’ll always, even if I don’t have the culture and the Udip is kinda eh, means to me the leuks are trace. If they’re my age and up, maybe even 55, because those sensors don’t work as well and you don’t get as much dysuria, but you can really get sick. If they act sick I’ll treat them. Even if they kinda don’t act sick, but that looks like a UTI even if I don’t have all the information. For a younger group, if it doesn’t look like a UTI and they’re sexually active and have things that could be more irritants, cause younger women have just more stuff going on. They’re exercising, they're wearing tight clothes. I’ll tell them I wanna wait until the culture is back, unless they have a fever. Sometimes, I see under 21s, so with that crowd if they have a fever I’ll start them on something.

J: How about males and females?

014: I get so so few males with UTIs in this current practice that I kind of forget about them. When I was dealing more with older adults, I’d get what sounded like a UTI but probably was prostatitis. I’d give them cipro, but I’d probably have to look it up now. It seems like in my memory the males that complained of something usually acted like they needed medicine and we just went ahead and took care of it.

<Files\\02_07_19_015> - § 10 references coded [11.91% Coverage]

Reference 1 - 0.28% Coverage

There is a difference if she had blood or ____ 0:01:42.8 cells maybe I'd change my mind.

References 2-4 - 3.22% Coverage

Ok. When you get the urine studies, what do you look for on those that would make you more concerned for a urinary tract infection?

015: The PH.

M: Ok.

015: _________04:47 and I will look at the WBC's.

M: Ok.

015: Epithelial cells. The ratio, when I went to school in my training years they used to tell you one to ten was the ratio between epithelial cells and WBC's.

M: Sure.

015: Typically, I'll have a conversation with a new patient. This is how long? He says, no, no, that don't work, I don’t feel comfortable with that. If I see fifteen WBC's and two epithelial cells, it's unequivocal and I will wait to see what the.... I always explain how to do the clean catch, and I explain very well so they get it. They get it right.

M: Good. Yea.

015: Then I'll look to the leukocyte esterase.

M: Ok.

015: And bacteria’s. Now, the specific gravity is going to tell me whether they are dehydrated or not.

M: Sure.

015: Sometimes concentrated urine can cause some irritation and the presence or absence of red cells.

Reference 5 - 2.46% Coverage

What duration?

M: Yes.

015: In the simple uncomplicated, would be a single dose.

M: Ok.

015: It could be a single dose. If it's a little more long....some patients don't feel comfortable, if she is older or...then I might give her three to seven days depending on how I submitted the urine and if I feel that it is going to be very easy...the culture is probably going to be negative, I don't give too many antibiotics.

M: Ok.

015: If it is over the weekend then I going to have to extend antibiotics because I don't want them to go to the Emergency Room.

M: Ah, for sure.

015: So, if the culture is done, say on a Thursday, Friday, then I’ll start with giving them more antibiotics, but if it's done on a Monday through Wednesday then I'll feel more comfortable calling ___ 07:38

References 6-7 - 1.85% Coverage

Ok. For sure. And when you order the specific testing, just kind of curious, just from clinic to clinic, I'm kind of asking, does your office do the urine dipsticks or is there an attached lab that you send the urine studies for? How specifically do you order the urine testing?

015: I would order the urinalysis with reflex culture because if I don't they do the dipstick.

M: Ok.

015: And then if the dipstick is equivocal or positive now the specimen can be contaminated, I don't know. It takes a while before when you get the dipstick and I don't like that.

M: Ok.

015: The dipstick.

Reference 8 - 1.11% Coverage

when I show them the negative, this is the evidence. This is what CBC says, but they’re just usually interested for infection. I have not seen a UTI. ____13:54 . Look at this, This is what they say "Look at the television". So I try to educate them but then the problem is going to be this. If I don't give up, in some patients, they go to the Urgent Care.

Reference 9 - 1.12% Coverage

In my office, if a patient come with symptoms of UTI, the protocol is the patient is to go to the lab to have a urinalysis. Then I see the patient. So by then I have a better understanding what the problem is. In nursing homes the nurses have a different protocol.

M: Okay.

015: With the patients…. And that is to decrease the amount of unnecessary UTI’s.

Reference 10 - 1.87% Coverage

When they do the urinalysis though, do they have someone that is able to interpret the results?

015: Yea. Well, they might call the doctor if they don’t know. I’m not sure. Probably they would call the doctor every time.

M: Okay.

015: Because this came from Medicare. Medicare felt that the nursing homes were over treating and they call it an S doc, I don’t know it, because it’s an S doc….. and the nursing home got an S doc and they have to fix that… and it was fixed within the first two months just by doing the protocol about not thinking of UTI’s just because they have urine smell, color.

<Files\\08_15_18_002> - § 2 references coded [5.28% Coverage]

Reference 1 - 1.57% Coverage

that urine dip, the UA comes back showing leukocytes, nitrites, even trace of blood but I would probably still then that is probably give Bactrim it's inexpensive it's well tolerated it usually works for most people so it makes good sense in all the cultures most of the cultures we had have come back sensitive to bactrim so it's nice and easy.

Reference 2 - 3.72% Coverage

We have had, it comes in waves a test dosent sound like a UTI typical UTI and the way they describe the symptoms that the urine is completely clean the other well hydrated the pH of normal no leukocytes no nitrites it looks beautiful and the way they aaare describing the symptoms just don't sound right and you think it might actually be more bladder spasms so there's a like 30 40 50 year olds usually women who are completely cleaned then I usually do not put them on antibiotics and I talked to them about it might be bladder spasm and then we do like 3 or 4-day course of like one of the methylene blues or something to help with bladder spasms and in the meantime when the culture comes back if it's an infection which is only happened once or twice we are like yes you really have a UTI then we just send out abx.

<Files\\08_23_18_005> - § 2 references coded [5.13% Coverage]

References 1-2 - 5.13% Coverage

: Got it, and then the urine dipstick, is that, do you like to order it just cause it’s part of the practice, or if it’s negative does it change your decision making process, or if you have a low suspicion and it’s positive, kind of walk me through how you interpret that?

005: The dipstick makes a big difference, cause let’s say you have a woman that comes in, or a male that says they have UTI symptoms but the last three point-of-cares that’s resulted in the system were negative, their cultures were negative, and they’re presenting with urinary symptoms and I have a negative point-of-care dipstick, am I gonna treat it with an antibiotic? Odds are no, especially if they had consecutive negative culture results, why put somebody on a negative antibiotic if they don’t need it. I’m really conservative with antibiotic use when it

comes to that, so if they’ve had negative cultures in the past, I mean we wanna go interstitial cystitis, you know a male’s prostatitis there’s a big thought process that comes out and they

need to go see a uro-gyn and not treat it with an antibiotic. Obviously if they’re insistent that they have a UTI, the dipstick is negative, I’m willing to put them on something if they’re insistent until the culture comes back. If the culture’s negative they need to stop then follow-up. It just kinda depends.

<Files\\08_23_18_005 (2)> - § 1 reference coded [5.11% Coverage]

Reference 1 - 5.11% Coverage

: Got it, and then the urine dipstick, is that, do you like to order it just cause it’s part of the practice, or if it’s negative does it change your decision making process, or if you have a low suspicion and it’s positive, kind of walk me through how you interpret that?

005: The dipstick makes a big difference, cause let’s say you have a woman that comes in, or a male that says they have UTI symptoms but the last three point-of-cares that’s resulted in the system were negative, their cultures were negative, and they’re presenting with urinary symptoms and I have a negative point-of-care dipstick, am I gonna treat it with an antibiotic? Odds are no, especially if they had consecutive negative culture results, why put somebody on a negative antibiotic if they don’t need it. I’m really conservative with antibiotic use when it

comes to that, so if they’ve had negative cultures in the past, I mean we wanna go interstitial cystitis, you know a male’s prostatitis there’s a big thought process that comes out and they

need to go see a uro-gyn and not treat it with an antibiotic. Obviously if they’re insistent that they have a UTI, the dipstick is negative, I’m willing to put them on something if they’re insistent until the culture comes back. If the culture’s negative they need to stop then follow-up. It just kinda depends.

<Files\\09_05_18_007> - § 1 reference coded [2.66% Coverage]

Reference 1 - 2.66% Coverage

1. Usually, elderly or demented patients. What about pyuria, do you consider it under microscopy to be considered, or because 0-5 is normal, over 5 is what comes up as a red flag in our lab.

1. Yeah, I will usually use symptoms still. So if they have piuria and their culture is positive, but they don’t have symptoms I still won’t treat them, so I’ll still usually base it on symptoms, as long as I think they can mount symptoms. For patients that are immunosuppressed or other things that may be different, but I think that’s one of the areas where knowing if they have symptoms is gonna be an important part in your decision making process. Our lab here uses a cutoff of 10 in hospital, so they won’t do a reflex culture if there’s fewer than 10. But there’s the practices vary from place to place.

<Files\\11_14_18_009> - § 6 references coded [13.58% Coverage]

Reference 1 - 2.34% Coverage

we don’t treat anybody unless they give us a urinalysis. Except maybe in some very specific cases, you know, that we’d do that without a urinalysis. But the urinalysis results would probably 95% of the time be an indicator to treat or not to treat. But, you know, if there’s somebody who has definitie symptoms that sounds like a UTI, wouldn’t necessarily not treat her either.

Reference 2 - 2.46% Coverage

And then, you mentioned getting a urinalysis, is it usually a urine dipstick that you get in clinic?

009: Yeah, so we’d do a urine dipstick in the clinic, and then if that is abnormal, then we’d send it to the lab for UA and urine culture. Now if somebody has a UTI, their urinalysis is negative on the dip but I still suspect that it’s a UTI, then I’ll still send the urinalysis off to the lab.

Reference 3 - 4.07% Coverage

There’s been instances where the culture came back positive, but the dip was negative, for example.

M: So if you have a strong clinical suspicious but the dip is negative you’ll still send a culture out.

009:Yeah, definitely. Like I said, I read in residency we had a form about this in one of our conferences. Whether you would treat somebody regarding this, and the 2 basic things that they were talking about were that you always wanna get a urinalysis for sure, but a woman’s symptoms sometimes aren’t very accurate, and saying if you have a UTI over a dipstick sometimes, you know? Basically the background knowledge I would use to make a decision

Reference 4 - 2.34% Coverage

do you have certain antibiotics that you prefer to treat UTIs with over others?

009: Yeah, I do. I think that’s not for any specific reason, I think I was just trained, used to prescribing those antibiotics. But definitely look at the culture, the sensitivity, all that stuff. But traditionally we’d do macrobid or cipro are the two tat we would prescribe most of the time.

References 5-6 - 2.38% Coverage

M: Got it. And then you mentioned before different lab values and things being useful for you in terms of UA. Are there specific urine dip results or culture results that you think are more important or more useful?

009: The urinalysis, I’m looking at if there’s any blood in the urine, and if there’s any signs of infection. I mean those are basically the 2 things I’m looking for.

<Files\\12_18_18_010> - § 4 references coded [27.03% Coverage]

Reference 1 - 6.91% Coverage

I would do a physical exam. I’d ask her if there’s blood in her urine, does she think that she’s, sometimes people can link symptoms to something like sitting in a hot tub or sexual intercourse, see if anything has changed from normal activity. I'd get a urine sample from her, and do a urine dip, and if it’s positive or looks like traces of blood, then definitely send for culture. In a young, healthy female, if I’m convinced she has a UTI, we’ll definitely send for culture as well but put her on 3 days of therapy, oral therapy twice a day, maybe septra, depending on her allergies of course. And I usually don’t bring people back for recheck unless symptoms come back, advise them to drink water/fluid avoid caffeine, alcohol, activities that would irritate the urinary tract. If you have any fever, chills, etc.

Reference 2 - 5.59% Coverage

You mentioned giving 3 days of Septra for this patient once you get the test results, are there any other antibiotics that you would choose?

010: I would start either Macrobid or Septra depending on their allergies. I would not reach for Cipro right away, especially if its somebody who doesn’t have a history of recurrence. She’s got a short history of it, it doesn’t sound like. If her dip is negative, I wouldn’t start her on an antibiotic, I’d say to her we’re just gonna wait for the culture to come back. BUt, i wouldn’t use Cipro or anything like that to start out, i’d use septra or macrobid, maybe keflex, especially if she’s naive to antibiotics.

Reference 3 - 7.89% Coverage

since we’re talking about treatment, what factors do you consider when deciding when or when not to treat a UTI? Do you consider symptoms, age, sex or other risk factors?

010: Well, when did it start? If it just started this morning, that’s a bit different than if it’s going on for several days. Especially in older women, they can confuse urinary frequency and even dysuria with a UTI, when it can be atrophic vaginitis, in younger women it can be a vaginal infection, so I’d wanna find out do you have any discharge. But, let’s say it’s an 85 year old woman whose had symptoms for several days and is very uncomfortable, and the dip is positive for blood, anything else, then I would start her right away. If she’s elderly woman and the symptoms are convincing, I might give a day or 2 of antibiotics and see what that culture comes back with. I don’t want her to get uroseptic if she’s fragile. Does that answer the question?

Reference 4 - 6.64% Coverage

You talked about getting a urine sample and doing a dip, then if you find certain values you send it for culture. Can you talk more about what kind of lab work do you get done?

010: Depending on the age, if it’s a young healthy person, even if the urine is clean, a lot of times I’ll send it off to make sure we didn’t miss anything. Sometimes something shows up in the lab, micro culture sensitivity, but I wouldn’t routinely like a CBP and a CME unless somebody had severe symptoms, you know or if they’re an older person who I’m worried is dehydrated. If there’s blood in urine, and they’ve got a history of renal stones and blood in urine, then I might order an ultrasound with SDB. If they have painless hematuria, definitely gonna do a CT, with stone protocol, so does that..?

<Files\\12_19_18_011> - § 3 references coded [5.79% Coverage]

References 1-2 - 2.36% Coverage

J: What should you see on a UA for you to consider it to be a UTI?

011: Definitely positive nitrites, that’s automatic, then depending on the lokoestorates, then i usually send off for culture to confirm that antibiotic use is the correct one.

Reference 3 - 3.43% Coverage

Usually it comes down to if it is just a very light leukoesterase, and it’s a younger patient, then I try to see if there’s some other issue for dysuria, whether it be hygiene, a yeast infection, or something else going on there. That’s when I usually don’t treat it as UTI. If lukesterases are the only things that are positive, I try to look at other options.

<Files\\12_20_18_012> - § 3 references coded [12.93% Coverage]

Reference 1 - 4.06% Coverage

Are there certain antibiotics which you consider better or worse than others?

012: It depends, it they’re having recurrent UTIs then I’d get a culture and it would tell you which antibiotic is better for that strain of bacteria, but I’d usually go with bactrim or cipro or possibly macrobid depending on drug allergies.

Reference 2 - 5.39% Coverage

What factors do you consider when deciding to treat a UTI?

012: Mostly patient symptoms, depending on what their UA shows, lab work shows

J: Do you consider, age, sex, stuff like that?

012: I think that you’re looking at just treating with symptoms. I more do that in females because it’s more common in females. In males you do question it because it’s more common in females, maybe they should come in for a visit.

Reference 3 - 3.49% Coverage

Do you use any specific resources or guidelines?

012: Not really, I usually like get a UA with the culture and I’ll treat them, and if we have recurrent UTIs, then I think about treating for a longer duration. I don’t think there’s any specific guidelines that I’m following

<Files\\8_13_18_001> - § 3 references coded [5.11% Coverage]

Reference 1 - 0.59% Coverage

so obviously we check a urine dip in the office make sure she is not pregnant.

References 2-3 - 4.52% Coverage

One thing that was helpful is I don't know why our labs we seem to get a lot of these false positive blood in the urine and so they they add it and now it looks like they dropped off doing it they were at they were checking for vitamin C you know to see if it wasn't you know and false positive whatever now it doesn't seem like they're doing that as much anymore so and I try to get more urine cultures if I seem to see people that look like it's a UTI and for leukocytes and then it's not ,then I try to get cultures and actually see if really a UTI or somebody’s got interstitial cystitis or something.

UTI Definition

<Files\\02_07_19_015> - § 2 references coded [1.02% Coverage]

References 1-2 - 1.02% Coverage

: For sure, for sure. So you mentioned simple cystitis. Do you think this would be complicated? Uncomplicated? Do you make that sort of distinction when you're thinking about these diagnoses?

015: I think it's uncomplicated because of the shortness duration. She's very healthy. No fever. I suppose the physical exam is normal.

<Files\\08_15_18_004> - § 1 reference coded [0.53% Coverage]

Reference 1 - 0.53% Coverage

Always look for CVA tenderness as its kind of game changer as far as antibiotics.

<Files\\08_23_18_005> - § 3 references coded [12.09% Coverage]

Reference 1 - 1.05% Coverage

Can you walk me through kind of what your thought process is if this person were walking into a clinic?

005: As far as the urinary frequency, um, suspected UTI, ask her if she’s ever had a urinary tract infection before. Obviously get a dipstick point-of-care urinalysis.

Reference 2 - 5.10% Coverage

: Got it, and then the urine dipstick, is that, do you like to order it just cause it’s part of the practice, or if it’s negative does it change your decision making process, or if you have a low suspicion and it’s positive, kind of walk me through how you interpret that?

005: The dipstick makes a big difference, cause let’s say you have a woman that comes in, or a male that says they have UTI symptoms but the last three point-of-cares that’s resulted in the system were negative, their cultures were negative, and they’re presenting with urinary symptoms and I have a negative point-of-care dipstick, am I gonna treat it with an antibiotic? Odds are no, especially if they had consecutive negative culture results, why put somebody on a negative antibiotic if they don’t need it. I’m really conservative with antibiotic use when it

comes to that, so if they’ve had negative cultures in the past, I mean we wanna go interstitial cystitis, you know a male’s prostatitis there’s a big thought process that comes out and they

need to go see a uro-gyn and not treat it with an antibiotic. Obviously if they’re insistent that they have a UTI, the dipstick is negative, I’m willing to put them on something if they’re insistent until the culture comes back. If the culture’s negative they need to stop then follow-up. It just kinda depends.

Reference 3 - 5.94% Coverage

. Um, so, I think the next question is, what factors do you consider when you’re treating someone? Are there certain exam things that you look at? Does age or sex matter? Can you walk me through some of that as well? It sounds like you mentioned a little bit about age and sex earlier too.

005: Yes, as far as, let’s start with females. Have they had a UTI before? Are these their typical urinary tract infection symptoms? Does this feel like something else? Do they have a history of kidney stones? New sexual partners? Are the symptoms severe or tolerable? And then, like you said as far as females, I mean there could be interstitial cystitis, they could be having bladder spasms. Atrophic vaginitis has a tendency to cause similar UTI symptoms in postmenopausal or

women of that age group. And then younger females, you always wanna question new sexual partners, is there a concern for any STIs, and stuff like that. Those are kinda the guidelines as far as that goes.

M: I think that’s great.

005: And then males, it depends on age. I mean generally a male comes in with urinary symptoms, of a certain age range, up until the age of 40, 45, we’re looking STI, do we have a history of any new sexual partners, do we have a history of UTIs, kidney stones, any prostate issues, any prostatitis, any pain with bowel movements, any blood with ejaculation, just those kind of symptoms. And you kinda have to mainstream it to see kind of what else they answer and then urine dipstick shows and then if a culture comes back, so it just kinda depends.

<Files\\08_23_18_005 (2)> - § 3 references coded [12.10% Coverage]

Reference 1 - 1.05% Coverage

Can you walk me through kind of what your thought process is if this person were walking into a clinic?

005: As far as the urinary frequency, um, suspected UTI, ask her if she’s ever had a urinary tract infection before. Obviously get a dipstick point-of-care urinalysis.

Reference 2 - 5.11% Coverage

: Got it, and then the urine dipstick, is that, do you like to order it just cause it’s part of the practice, or if it’s negative does it change your decision making process, or if you have a low suspicion and it’s positive, kind of walk me through how you interpret that?

005: The dipstick makes a big difference, cause let’s say you have a woman that comes in, or a male that says they have UTI symptoms but the last three point-of-cares that’s resulted in the system were negative, their cultures were negative, and they’re presenting with urinary symptoms and I have a negative point-of-care dipstick, am I gonna treat it with an antibiotic? Odds are no, especially if they had consecutive negative culture results, why put somebody on a negative antibiotic if they don’t need it. I’m really conservative with antibiotic use when it

comes to that, so if they’ve had negative cultures in the past, I mean we wanna go interstitial cystitis, you know a male’s prostatitis there’s a big thought process that comes out and they

need to go see a uro-gyn and not treat it with an antibiotic. Obviously if they’re insistent that they have a UTI, the dipstick is negative, I’m willing to put them on something if they’re insistent until the culture comes back. If the culture’s negative they need to stop then follow-up. It just kinda depends.

Reference 3 - 5.94% Coverage

. Um, so, I think the next question is, what factors do you consider when you’re treating someone? Are there certain exam things that you look at? Does age or sex matter? Can you walk me through some of that as well? It sounds like you mentioned a little bit about age and sex earlier too.

005: Yes, as far as, let’s start with females. Have they had a UTI before? Are these their typical urinary tract infection symptoms? Does this feel like something else? Do they have a history of kidney stones? New sexual partners? Are the symptoms severe or tolerable? And then, like you said as far as females, I mean there could be interstitial cystitis, they could be having bladder spasms. Atrophic vaginitis has a tendency to cause similar UTI symptoms in postmenopausal or

women of that age group. And then younger females, you always wanna question new sexual partners, is there a concern for any STIs, and stuff like that. Those are kinda the guidelines as far as that goes.

M: I think that’s great.

005: And then males, it depends on age. I mean generally a male comes in with urinary symptoms, of a certain age range, up until the age of 40, 45, we’re looking STI, do we have a history of any new sexual partners, do we have a history of UTIs, kidney stones, any prostate issues, any prostatitis, any pain with bowel movements, any blood with ejaculation, just those kind of symptoms. And you kinda have to mainstream it to see kind of what else they answer and then urine dipstick shows and then if a culture comes back, so it just kinda depends.

<Files\\08_23_18_006> - § 1 reference coded [6.92% Coverage]

Reference 1 - 6.92% Coverage

1. I would talk to her more about her symptoms, find out if there’s anything else going on, are you sexually active, is there a chance that this could be an STI, is there any chance you could be pregnant, even though you don’t think you are at the moment? Are you having frequency, urgency, hesitancy, is there blood in your urine, you ever have a kidney stone before? Fevers, back pain, abdominal pain, anything else going on? Then I would, the girls are really good about getting the urine-dip, talk to the patient about, well it looks like maybe there might be something, you’ve got some, if she’s got leukocytes or nitrites maybe she’s got something else going on. We can try an antibiotic, I would probably start with macrobid, twice a day for 7 days, unless they wanna balk at it. I would send it for culture no matter what. If I didn’t see anything that was indicative, I would try to talk them out of insisting on an antibiotic. I’d tell them I just really don’t see anything. What’s in the culture, if it comes back we can always start you on something, but it doesn’t really look like that’s where the problem is, so why don’t you follow up with primary or urologist.

<Files\\09_05_18_007> - § 1 reference coded [2.42% Coverage]

Reference 1 - 2.42% Coverage

1. 27 year old, not pregnant, no past medical history, presenting with urinary frequency and urgency?

1. Correct, we’re looking for bread and butter what your thoughts are.

1. She has classic symptoms of urinary tract infection. I would check her vitals, I would ask her if she has any malaise, fatigue, associated with her symptoms. I would ask her if she has any tenderness on her flank area, and with her classic symptoms I would like to treat her with antibiotics. I know that routinely we do a urine dipstick before seeing a patient. That is still, I do it, however I think that classic symptoms in a patient like her can be treated with a short course of antibiotics. In a real world, in my practice, I do do a urine dipstick

<Files\\10_10_18_008 (2)> - § 1 reference coded [3.13% Coverage]

Reference 1 - 3.13% Coverage

Sure. If she was in my clinic I would likely get a UA and depending how symptomatic she was, potentially start her on prophylactic or empiric treatment until I had the results of the UA back.

M: Got it. Okay. So would you consider this a pretty uncomplicated case of urinary tract infection?

8: I would.

M: So you mentioned you’d get urine tests. What kind of tests do you like to do?

8: Oh, I do the UA with reflex to culture.

<Files\\12_18_18_010> - § 1 reference coded [0.87% Coverage]

Reference 1 - 0.87% Coverage

Would you consider this to be an example of a complicated or uncomplicated UTI?

010: Uncomplicated

<Files\\12_19_18_011> - § 1 reference coded [1.83% Coverage]

Reference 1 - 1.83% Coverage

: Okay. Would you consider this to be an example of complicated or uncomplicated UTI?

011: At this point uncomplicated because I don’t know what the UA or vitals say, or any other symptoms.

<Files\\12_20_18_012> - § 1 reference coded [6.89% Coverage]

Reference 1 - 6.89% Coverage

27 year old female came into the clinic. NOthing else has been given out.

012: I would probably want to get a UA before doing anything. And then, depending on what that showed, possibly treat her with antibiotics. What else do you want to know? Which antibiotic?

J: Would you consider this to be an example of complicated or uncomplicated UTI?

012: Probably uncomplicated.

J: Can you explain why?

012: Because of the length of symptoms, as far as the information that’s given, I don’t think there’s enough to call it complicated yet.

<Files\\8_13_18_001> - § 1 reference coded [1.42% Coverage]

Reference 1 - 1.42% Coverage

Obviously there has to be a different algorithm for male because of prostatitis etc. It’s better at first to do only female and start out there because the male UTIs are more complicated.

When to test

<Files\\01_08_19_013> - § 4 references coded [14.67% Coverage]

Reference 1 - 2.54% Coverage

Tell me what your thoughts are, how would you approach this case?

013: I’d ask for more symptoms, see if there’s more evidence for a UTI although I’d probably have enough to get a urinalysis to start. She doesn’t have a lot of symptoms so I’d send of a UA with CNS for 99% of the time then wait for a culture to prescribe antibiotics.

Reference 2 - 3.22% Coverage

: Would your management with this be different if they called in vs showed up to clinic?

013: Normally I’d still recommend they go to a local lab and get a UA with CNS. At times I do treat empirically over the phone, but it’s really only if there’s somewhat unusual circumstances that precludes getting a timely UA with CNS.

M:Just to clarify, CNS are you talking about a reflex, or is that a micro?

013: Reflex, yeah.

Reference 3 - 3.13% Coverage

So if they called in, you’d clarify some questions and have them go to the lab. If they showed up, clarify some questions and have them go to the lab. Would treat them empirically, or would you wait for culture to come back?

013: Depends on symptoms, that’s sort of the art of medicine. The more concerning symptoms are, the more I give a 2 day dose of an antibiotics, sometimes 3 days and call it quits after that.

Reference 4 - 5.78% Coverage

In hospital setting we use antibiograms, in clinic settings I never see that being used. One of the thoughts I’m kicking around, and I’d like your thoughts on it, do you think antibiograms should be incorporated in outpatient settings, do you think it’s appropriate? Some of the guidelines use cutoff points for when a firstline antibiotic should be used based on local susceptibilities. Is that info ever sent to you, are you aware of local susceptibilities?

013: If i were just office setting we would have some trouble some trouble. I think it’s more about resistant infections, I don’t how much an antibiogram would change my opinion about what abx I’m using for a UTI, because you can always get a culture. For a respiratory infection, that’s a little different.

<Files\\01_10_19_014> - § 2 references coded [12.83% Coverage]

Reference 1 - 6.64% Coverage

How will you handle the case?

014: With somebody like that, we do the Udip in the office. If there’s anything that seems generally positive, leukocytes or nitrates or anything like that, we’ll go ahead and treat them, if it’s uncomplicated and they don’t do it all the time, I usually do 3-5 days of macrobid. It kinda depends, I stick more towards 5. It might be overkill, but since they usually aren’t in my setting, we’re primary care but we also do walk in, and we’re in a tourist area so we get people we’re never gonna see again. We kinda wanna send them off adequately set up, and then we’ll send that urine for culture. FOr everybody that has insurance we send the culture, sometimes if they don’t we give them the choice. Otherwise we just go with it and tell them to follow up if they don’t have resolution of symptoms.

Reference 2 - 6.19% Coverage

Some previous studies have demonstrated that nursing staff can successfully follow simple algorithms for UTIs, freeing up clinician time for more complicated decisions. Can you share additional thoughts?

014: Even to the point of ordering medications?

J: Yes

014: I, in my setting, because I have MAs and LPNs, I think they pretty well do everything but prescribing. They interview, I confirm it, but I look at the medicines and double check the allergies, and make sure that what they’re gonna take is okay for them. I just do a little more investigation before I click prescribe. If somebody complains of a UTI, they put them in the restroom, they get the dip, they order the culture without my direction. I don’t know that I’d be comfortable having them prescribe.

<Files\\02_07_19_015> - § 8 references coded [12.69% Coverage]

Reference 1 - 0.81% Coverage

She could have just a very mild cystitis or it could be treated very simply just with over the counter medications, hydration. If she is not having any fever. I like to do... Always I like to do at least an uranalysis to have an idea of what's going on.

M: Ok.

References 2-3 - 1.47% Coverage

M: Yeah, and with her, she really didn't have any of those other sort of symptoms. It was just she was noticing she was urinating much more frequently and it was burning every time that she went. You had mentioned potentially wanting to get some urine studies. Do you typically get urine studies with complaints like this?

015: Well, I feel more comfortable doing the urine studies because patients come to the office with the intention of getting antibiotics immediately.

Reference 4 - 1.68% Coverage

And it's a young person. First time maybe. We don't even know what it is exactly. We think that it's uncomplicated and so if I have a urinalysis that sort of, changes her mind, I could talk to her into just waiting to see what happens and see just symptomatically, and hydration, over the counter medications and wait before we start antibiotics.

M: Ok. Perfect. And what over the counter medications do you typically recommend?

015: The AZO.

M: Which one is that?

015: The AZO. It's called AZO. (spells out) A- Z- O. It's very common.

Reference 5 - 2.46% Coverage

What duration?

M: Yes.

015: In the simple uncomplicated, would be a single dose.

M: Ok.

015: It could be a single dose. If it's a little more long....some patients don't feel comfortable, if she is older or...then I might give her three to seven days depending on how I submitted the urine and if I feel that it is going to be very easy...the culture is probably going to be negative, I don't give too many antibiotics.

M: Ok.

015: If it is over the weekend then I going to have to extend antibiotics because I don't want them to go to the Emergency Room.

M: Ah, for sure.

015: So, if the culture is done, say on a Thursday, Friday, then I’ll start with giving them more antibiotics, but if it's done on a Monday through Wednesday then I'll feel more comfortable calling ___ 07:38

Reference 6 - 2.61% Coverage

Do you ever get phone calls, say, tasks from patients that call into your staff that pass along a message to you? Do you ever get those sorts of messages that patients are calling complaining of either dysuria or sometimes patients are calling and saying that they have a urinary tract infections? Do you get those messages?

015: Yes. We get by phone call, but we also get it through the Epic. The younger patients use the Epic.

M: Ok. The Epic My Chart Messaging.

015: Yes.

M: How do you typically handle those cases? Do you, do you ever treat over the phone, or via the Epic Messaging?

015: I prefer that they just come...if they want to be treated, especially if they want to save money...whatever. They at least should pay for the urinalysis. I want to make sure that before I treat them I know what I am dealing with.

M: Sure.

Reference 7 - 1.80% Coverage

when you order the specific testing, just kind of curious, just from clinic to clinic, I'm kind of asking, does your office do the urine dipsticks or is there an attached lab that you send the urine studies for? How specifically do you order the urine testing?

015: I would order the urinalysis with reflex culture because if I don't they do the dipstick.

M: Ok.

015: And then if the dipstick is equivocal or positive now the specimen can be contaminated, I don't know. It takes a while before when you get the dipstick and I don't like that.

M: Ok.

015: The dipstick.

Reference 8 - 1.87% Coverage

When they do the urinalysis though, do they have someone that is able to interpret the results?

015: Yea. Well, they might call the doctor if they don’t know. I’m not sure. Probably they would call the doctor every time.

M: Okay.

015: Because this came from Medicare. Medicare felt that the nursing homes were over treating and they call it an S doc, I don’t know it, because it’s an S doc….. and the nursing home got an S doc and they have to fix that… and it was fixed within the first two months just by doing the protocol about not thinking of UTI’s just because they have urine smell, color.

<Files\\08_15_18_002> - § 2 references coded [6.70% Coverage]

Reference 1 - 4.85% Coverage

. Here in Convenient Care they come in with the symptoms everyone gets a UA and based on those symptoms even if the UA came back with negative nitrites and even trace leukocytes still probably would treat but we always here we always send cultures unless they are self-pay, so here they get a culture and if self-pay we will go back and forth and talk to them and this is how much it's going to cost but it'll give us these benefits to make sure it is an infection by using the right medicine to treat for and most of the time they do not want the self-paid do not want the culture. People when it's not self-pay their like okay sure we get the cultures back and I'd say probably the majority of the time there is an infection but there's probably 30 to 35% maybe even 40% when there is not an infection and so will call him back and we'll say stop the antibiotics if you're still having symptoms you need to follow up with your primary care or whoever your OB-GYN sometimes if they don't have a PCP but in that case someone who has no medical problems 2 days of symptoms.

Reference 2 - 1.85% Coverage

guys don't usually get UTI so when are coming in we're also and what we they may have bactrim as well but we usually also do what's called a dirty urine which is looking for gonorrhea and chlamydia and anything else because guys don't usually get you don't usually see it in the young guys, older guy certainly could be prostate related but we don't have the ability to do labs here so I didn't check the PSA.

<Files\\08_15_18_003> - § 4 references coded [12.06% Coverage]

References 1-3 - 10.75% Coverage

little more information from the patient, get more adequate history, sexual practices ,may be more specific information about what she means by her complaints, try to get more descriptors qualifiers like that how long has been going on for? Did you notice any symptoms leading up to it? How much is it affecting your day? How bad is it? What u mean by burning? can you be more precise and try to get more details about the complaint. Usually the exam is pretty brief other than just vital signs and the general appearance, may be checking for flank pain, abdominal pain. Urinalysis – get started with that.

M—you do dip in the office or do u do a complete UA? What do you do?

003—urine dips here in the office

M--got it and then can you talk about cultures do you sometimes get cultures never get cultures always what are you practice?

003-Almost always always. There are probably a few exceptions but we we we we like to be consistent with culturing just about anyone with a urinary complaint, there are some where it's really obvious that they don't have urinary tract infection and there's no blood in urine or anything else to really they just wanted to get…..The MA might have ordered it thinking I might want it because they had back pain and was really musculoskeletal so clearly not urinary. so probably 95% of the cases will do a urine culture or there may be an exception where someone like, man I can't afford any and I'm only if you send it out and bill me later I just can't afford it please don't order any tests on me, just a urine dip on them. Majority of them are culture.

M—So if you are really convinced or you're really suspicious about the UTI diagnosis does that change your practices for whether you are going to test them or get a culture or do you always regardless of your kind of pretest probability you always get a urine test?

003-- it in my mind in my mind I'm taking okay this is pretty straightforward but we still do it, yeah in this setting, because if for some reason there is antibiotic resistance then we can go back to the …okay this is what we ordered we got to change the antibiotics and then perhaps not going to work and maybe that's why you r not getting better. And since it's a seven-days-a-week operation I may not be here and I dont know the whole story. Someone might be able look at my chart and kinda see what is going on with it, it may be a better safety net if you want to use that term, making sure that the proprietors here when I'm not here they can just easily look at the the culture and say oh he prescribed Cipro and then can change to bactrim.

Reference 4 - 1.30% Coverage

--if you're calling them and managing over the phone do you still have them come in later on for a urine test or do you just say that's your history is enough for it or what could be ?

003--normally I would say you don't have to come back and follow up just if you're not better please return and let's reevaluate.

<Files\\08_15_18_004> - § 6 references coded [16.84% Coverage]

References 1-2 - 2.89% Coverage

Can you walk me through how you would approach this case of if they showed up to your clinic?

004--Generally we will just see urine after a history and physical obviously I guess not obviously. Always look for CVA tenderness as its kind of game changer as far as antibiotics. Generally what I'll do is send urine for culture if they're in the office and start him on an antibiotic, probably since you said 27 give 5 to 7 days of antibiotics.

Reference 3 - 2.64% Coverage

Got it and then in terms of the testing do you like to test it everybody or do your testing practices change if they call you on the telephone or send a message to you instead.

004--If they're not in person we would probably just in this age group so younger patient without any other symptoms and recognize symptoms as bladder infection I don't hesitate to treat over the phone with antibiotics 5-7 days

References 4-5 - 3.83% Coverage

M--and then you mentioned that you like to get the testing, does that change based on your pretest probability of you have a strong suspicion or low suspicion of a UTI based on the history does that change whether or not you would obtain testing in a culture on them?

004--Yeah it does a little but frankly we don't see them in the office that much so the reason I would get a culture if they actually showed up. That would indicate to me they are feeling sicker than I might otherwise give a phone call saying I have another bladder Infection can I have antibiotics? that happens a lot…

Reference 6 - 7.49% Coverage

can you go walk me through what happens when you receive a message about the patient having a UTI… how does it get to you? What’s the content of the message usually?

004--Nine times out of 10 it is a message it's usually texted to me. Our staff is trained to answer sort of red flag questions like fever, CVA tenderness or other symptoms, hematuria also. But most of my patients are pretty well established right now have had bladder infections, they call up and say I've got another bladder infection …can I have antibiotics? We treat them once and ask them to come in after five days to get cultured.

M-- And then so how useful are the messages that you get from your staff do you think they contain all the key information that allows you to make a decision?

004—90% of the time they do. We have a call center and they have protocols and then my secretary screens all of the calls and she's really good. If we are lacking the right information she will go after it.

M-- Did she ever tell you how often the right information is missing? is it a big burden on on her?

004—This is not a big burden because we hammered this thing in our office.

<Files\\08_23_18_005> - § 7 references coded [18.63% Coverage]

References 1-2 - 1.05% Coverage

Can you walk me through kind of what your thought process is if this person were walking into a clinic?

005: As far as the urinary frequency, um, suspected UTI, ask her if she’s ever had a urinary tract infection before. Obviously get a dipstick point-of-care urinalysis.

References 3-4 - 4.00% Coverage

So, it sounds like you think it's pretty straightforward overall. I made an uncomplicated case for you too. You mentioned some testing, do you always get testing on everybody, or what makes you decide about testing, and then can you tell me about culture practices too?

005: As far as here, I mean, we’re a walk-in clinic, so nobody can call in and say “Hey I think I have a UTI can you send me something.” They all have to be seen in-office. So in every single patient I’m gonna test, we’re gonna get a point-of-care urine dipstick and then go from there. As far as urine cultures, it’s kind of our practice here to, because it’s not a primary office, they don’t call and say let’s switch antibiotics, so I want to say generally unless they don’t have insurance that were doing a urine culture on everyone, because if there is resistance we can call them back and say “hey we switched it, it wasn’t a UTI you need to follow up elsewhere and get

follow-up care.” So generally we do a urine culture on most everybody, it’s just our practice here.

Reference 5 - 5.10% Coverage

: Got it, and then the urine dipstick, is that, do you like to order it just cause it’s part of the practice, or if it’s negative does it change your decision making process, or if you have a low suspicion and it’s positive, kind of walk me through how you interpret that?

005: The dipstick makes a big difference, cause let’s say you have a woman that comes in, or a male that says they have UTI symptoms but the last three point-of-cares that’s resulted in the system were negative, their cultures were negative, and they’re presenting with urinary symptoms and I have a negative point-of-care dipstick, am I gonna treat it with an antibiotic? Odds are no, especially if they had consecutive negative culture results, why put somebody on a negative antibiotic if they don’t need it. I’m really conservative with antibiotic use when it

comes to that, so if they’ve had negative cultures in the past, I mean we wanna go interstitial cystitis, you know a male’s prostatitis there’s a big thought process that comes out and they

need to go see a uro-gyn and not treat it with an antibiotic. Obviously if they’re insistent that they have a UTI, the dipstick is negative, I’m willing to put them on something if they’re insistent until the culture comes back. If the culture’s negative they need to stop then follow-up. It just kinda depends.

Reference 6 - 5.94% Coverage

. Um, so, I think the next question is, what factors do you consider when you’re treating someone? Are there certain exam things that you look at? Does age or sex matter? Can you walk me through some of that as well? It sounds like you mentioned a little bit about age and sex earlier too.

005: Yes, as far as, let’s start with females. Have they had a UTI before? Are these their typical urinary tract infection symptoms? Does this feel like something else? Do they have a history of kidney stones? New sexual partners? Are the symptoms severe or tolerable? And then, like you said as far as females, I mean there could be interstitial cystitis, they could be having bladder spasms. Atrophic vaginitis has a tendency to cause similar UTI symptoms in postmenopausal or

women of that age group. And then younger females, you always wanna question new sexual partners, is there a concern for any STIs, and stuff like that. Those are kinda the guidelines as far as that goes.

M: I think that’s great.

005: And then males, it depends on age. I mean generally a male comes in with urinary symptoms, of a certain age range, up until the age of 40, 45, we’re looking STI, do we have a history of any new sexual partners, do we have a history of UTIs, kidney stones, any prostate issues, any prostatitis, any pain with bowel movements, any blood with ejaculation, just those kind of symptoms. And you kinda have to mainstream it to see kind of what else they answer and then urine dipstick shows and then if a culture comes back, so it just kinda depends.

Reference 7 - 2.53% Coverage

Got it. And then if you weren’t working in a convenient care setting and you got a phone call from a patient let’s say, would you want them to come in to see you still to get a urine dipstick, or would you feel comfortable treating over the phone, what are your thoughts about that?

005: How I’ve been trained, with my experience, mostly I have been walk-in clinic based, I want the dipstick. If it’s a patient and I’m in a family office and I know them and see their history and it’s something I would feel comfortable saying hey we can do this antibiotic if symptoms aren’t better, follow up. It goes against my gut, but have I done it and will I do it? Yes.

<Files\\08_23_18_005 (2)> - § 5 references coded [18.63% Coverage]

Reference 1 - 1.05% Coverage

Can you walk me through kind of what your thought process is if this person were walking into a clinic?

005: As far as the urinary frequency, um, suspected UTI, ask her if she’s ever had a urinary tract infection before. Obviously get a dipstick point-of-care urinalysis.

Reference 2 - 4.00% Coverage

So, it sounds like you think it's pretty straightforward overall. I made an uncomplicated case for you too. You mentioned some testing, do you always get testing on everybody, or what makes you decide about testing, and then can you tell me about culture practices too?

005: As far as here, I mean, we’re a walk-in clinic, so nobody can call in and say “Hey I think I have a UTI can you send me something.” They all have to be seen in-office. So in every single patient I’m gonna test, we’re gonna get a point-of-care urine dipstick and then go from there. As far as urine cultures, it’s kind of our practice here to, because it’s not a primary office, they don’t call and say let’s switch antibiotics, so I want to say generally unless they don’t have insurance that were doing a urine culture on everyone, because if there is resistance we can call them back and say “hey we switched it, it wasn’t a UTI you need to follow up elsewhere and get

follow-up care.” So generally we do a urine culture on most everybody, it’s just our practice here.

Reference 3 - 5.11% Coverage

: Got it, and then the urine dipstick, is that, do you like to order it just cause it’s part of the practice, or if it’s negative does it change your decision making process, or if you have a low suspicion and it’s positive, kind of walk me through how you interpret that?

005: The dipstick makes a big difference, cause let’s say you have a woman that comes in, or a male that says they have UTI symptoms but the last three point-of-cares that’s resulted in the system were negative, their cultures were negative, and they’re presenting with urinary symptoms and I have a negative point-of-care dipstick, am I gonna treat it with an antibiotic? Odds are no, especially if they had consecutive negative culture results, why put somebody on a negative antibiotic if they don’t need it. I’m really conservative with antibiotic use when it

comes to that, so if they’ve had negative cultures in the past, I mean we wanna go interstitial cystitis, you know a male’s prostatitis there’s a big thought process that comes out and they

need to go see a uro-gyn and not treat it with an antibiotic. Obviously if they’re insistent that they have a UTI, the dipstick is negative, I’m willing to put them on something if they’re insistent until the culture comes back. If the culture’s negative they need to stop then follow-up. It just kinda depends.

Reference 4 - 5.94% Coverage

. Um, so, I think the next question is, what factors do you consider when you’re treating someone? Are there certain exam things that you look at? Does age or sex matter? Can you walk me through some of that as well? It sounds like you mentioned a little bit about age and sex earlier too.

005: Yes, as far as, let’s start with females. Have they had a UTI before? Are these their typical urinary tract infection symptoms? Does this feel like something else? Do they have a history of kidney stones? New sexual partners? Are the symptoms severe or tolerable? And then, like you said as far as females, I mean there could be interstitial cystitis, they could be having bladder spasms. Atrophic vaginitis has a tendency to cause similar UTI symptoms in postmenopausal or

women of that age group. And then younger females, you always wanna question new sexual partners, is there a concern for any STIs, and stuff like that. Those are kinda the guidelines as far as that goes.

M: I think that’s great.

005: And then males, it depends on age. I mean generally a male comes in with urinary symptoms, of a certain age range, up until the age of 40, 45, we’re looking STI, do we have a history of any new sexual partners, do we have a history of UTIs, kidney stones, any prostate issues, any prostatitis, any pain with bowel movements, any blood with ejaculation, just those kind of symptoms. And you kinda have to mainstream it to see kind of what else they answer and then urine dipstick shows and then if a culture comes back, so it just kinda depends.

Reference 5 - 2.53% Coverage

Got it. And then if you weren’t working in a convenient care setting and you got a phone call from a patient let’s say, would you want them to come in to see you still to get a urine dipstick, or would you feel comfortable treating over the phone, what are your thoughts about that?

005: How I’ve been trained, with my experience, mostly I have been walk-in clinic based, I want the dipstick. If it’s a patient and I’m in a family office and I know them and see their history and it’s something I would feel comfortable saying hey we can do this antibiotic if symptoms aren’t better, follow up. It goes against my gut, but have I done it and will I do it? Yes.

<Files\\08_23_18_006> - § 1 reference coded [6.92% Coverage]

Reference 1 - 6.92% Coverage

1. I would talk to her more about her symptoms, find out if there’s anything else going on, are you sexually active, is there a chance that this could be an STI, is there any chance you could be pregnant, even though you don’t think you are at the moment? Are you having frequency, urgency, hesitancy, is there blood in your urine, you ever have a kidney stone before? Fevers, back pain, abdominal pain, anything else going on? Then I would, the girls are really good about getting the urine-dip, talk to the patient about, well it looks like maybe there might be something, you’ve got some, if she’s got leukocytes or nitrites maybe she’s got something else going on. We can try an antibiotic, I would probably start with macrobid, twice a day for 7 days, unless they wanna balk at it. I would send it for culture no matter what. If I didn’t see anything that was indicative, I would try to talk them out of insisting on an antibiotic. I’d tell them I just really don’t see anything. What’s in the culture, if it comes back we can always start you on something, but it doesn’t really look like that’s where the problem is, so why don’t you follow up with primary or urologist.

<Files\\09_05_18_007> - § 2 references coded [3.60% Coverage]

Reference 1 - 2.42% Coverage

1. 27 year old, not pregnant, no past medical history, presenting with urinary frequency and urgency?

1. Correct, we’re looking for bread and butter what your thoughts are.

1. She has classic symptoms of urinary tract infection. I would check her vitals, I would ask her if she has any malaise, fatigue, associated with her symptoms. I would ask her if she has any tenderness on her flank area, and with her classic symptoms I would like to treat her with antibiotics. I know that routinely we do a urine dipstick before seeing a patient. That is still, I do it, however I think that classic symptoms in a patient like her can be treated with a short course of antibiotics. In a real world, in my practice, I do do a urine dipstick

Reference 2 - 1.18% Coverage

1. Do you get a culture on them too, or no?

1. I don’t think I would do cultures, because she is presenting as very simple cystitis clinical symptoms without any fever, which is about more than 99.8, so 99.8 or 99.9, or 100 degree temperature, malaise, fatigue, or tenderness, or prior history of a urinary tract infection, I don’t think a culture is needed.

<Files\\10_10_18_008 (2)> - § 1 reference coded [3.13% Coverage]

Reference 1 - 3.13% Coverage

Sure. If she was in my clinic I would likely get a UA and depending how symptomatic she was, potentially start her on prophylactic or empiric treatment until I had the results of the UA back.

M: Got it. Okay. So would you consider this a pretty uncomplicated case of urinary tract infection?

8: I would.

M: So you mentioned you’d get urine tests. What kind of tests do you like to do?

8: Oh, I do the UA with reflex to culture.

<Files\\11_14_18_009> - § 5 references coded [15.69% Coverage]

Reference 1 - 1.32% Coverage

Yeah, so definitely first, I’d a thorough physical on the patient, then I would get a urinalysis dip in the clinic. And then, based off this history and her symptoms and the urine dip, make a decision going forward

Reference 2 - 2.34% Coverage

we don’t treat anybody unless they give us a urinalysis. Except maybe in some very specific cases, you know, that we’d do that without a urinalysis. But the urinalysis results would probably 95% of the time be an indicator to treat or not to treat. But, you know, if there’s somebody who has definitie symptoms that sounds like a UTI, wouldn’t necessarily not treat her either.

Reference 3 - 2.46% Coverage

And then, you mentioned getting a urinalysis, is it usually a urine dipstick that you get in clinic?

009: Yeah, so we’d do a urine dipstick in the clinic, and then if that is abnormal, then we’d send it to the lab for UA and urine culture. Now if somebody has a UTI, their urinalysis is negative on the dip but I still suspect that it’s a UTI, then I’ll still send the urinalysis off to the lab.

Reference 4 - 4.00% Coverage

in terms of patients that don’t show up to your clinic, say they call in with these symptoms, walk me through what you do in those circumstances?

009: Yeah, so let’s say somebody called and said I’ve been going to the dr a lot and it burns. I would basically try to work them in that day, so they could come in, do a urine that day, and get it done. If they’re unable to come in, then typically I’d say you have until the end of the day to go to the lab and give them a sample and we’ll give them antibiotics. So we try to always get a urine sample from them, whatever would be most convenient, but we definitely ask for them to come in first.

Reference 5 - 5.57% Coverage

There’s even some where they have computer kiosks where the patients enter their symptoms and the kiosk will evaluate it and send that information to a provider. THat’s been done in urgent care and emergency conditiins in califiornia, and then the providers sign off. I guess that’s kind of a hybrid where the providers still evaluate, like you’re talkin about. One thing I forgot to ask, for patients that you have a low suspicion based on symptoms for UTIs, do you still have the get cultures? What’s your threshold for getting a culture or urine dip?

009: I would have to suspect that they have a UTI for me to send it off to culture. If I have a low suspicion and urinalysis is negative, I wouldn’t send the culture.

M: What about a urine dip, if you have a low suspicion? Would you still get a urine dip?

009: Yeah if they come in with some symptoms I would definitely get a urine dip.

<Files\\12_18_18_010> - § 3 references coded [18.35% Coverage]

Reference 1 - 6.91% Coverage

I would do a physical exam. I’d ask her if there’s blood in her urine, does she think that she’s, sometimes people can link symptoms to something like sitting in a hot tub or sexual intercourse, see if anything has changed from normal activity. I'd get a urine sample from her, and do a urine dip, and if it’s positive or looks like traces of blood, then definitely send for culture. In a young, healthy female, if I’m convinced she has a UTI, we’ll definitely send for culture as well but put her on 3 days of therapy, oral therapy twice a day, maybe septra, depending on her allergies of course. And I usually don’t bring people back for recheck unless symptoms come back, advise them to drink water/fluid avoid caffeine, alcohol, activities that would irritate the urinary tract. If you have any fever, chills, etc.

Reference 2 - 6.64% Coverage

You talked about getting a urine sample and doing a dip, then if you find certain values you send it for culture. Can you talk more about what kind of lab work do you get done?

010: Depending on the age, if it’s a young healthy person, even if the urine is clean, a lot of times I’ll send it off to make sure we didn’t miss anything. Sometimes something shows up in the lab, micro culture sensitivity, but I wouldn’t routinely like a CBP and a CME unless somebody had severe symptoms, you know or if they’re an older person who I’m worried is dehydrated. If there’s blood in urine, and they’ve got a history of renal stones and blood in urine, then I might order an ultrasound with SDB. If they have painless hematuria, definitely gonna do a CT, with stone protocol, so does that..?

Reference 3 - 4.79% Coverage

If a patient calls in complaining of a UTI to your clinic, who takes the call and how does it get triaged?

010: It goes from phone people, to medical secretary of provider, then forwarded to provider. They find out if the provider wants them to come in, or does the provider want to treat sight unseen. So, I would say 9 times out of 10 I’m gonna say you need to come in so i can get some urine. There’s extenuating circumstances, like if a patient is very immunocompromised and I don’t wanna take a chance, but...so no, that’s how it goes from one to the other.

<Files\\12_19_18_011> - § 2 references coded [8.68% Coverage]

Reference 1 - 2.51% Coverage

2 days of dysuria and urinary frequency.

011: Okay, so I’d probably order a UA, initially, and then do we have nay vital signs, is she febrile or anything?

J: So that’s how you’d manage it? That could be a process?

011: Yeah initially vital signs and UA.

Reference 2 - 6.17% Coverage

How do you think antibiotic prescribing would work in such cases?

011: If the algorithm is made very specifically for the nursing staff then it should be fine, because if they don’t meet that certain criteria then they don’t get antibiotics, so they’d come in and give a urine sample and the provider could make that decision.

J: Is it something you see being used in your clinic in the future?

011: Probably as we fill up. I’m a new NP to this practice, and we have 2 new physicians, so we have room to grow. Maybe as providers and practices get too busy with more complicated cases then I could see that becoming more of a utilization of that.

<Files\\12_20_18_012> - § 3 references coded [20.81% Coverage]

Reference 1 - 4.84% Coverage

27 year old female came into the clinic. NOthing else has been given out.

012: I would probably want to get a UA before doing anything. And then, depending on what that showed, possibly treat her with antibiotics. What else do you want to know? Which antibiotic?

J: Would you consider this to be an example of complicated or uncomplicated UTI?

012: Probably uncomplicated.

Reference 2 - 8.09% Coverage

you said you’d get a UA to confirm your diagnosis and send it further to culture right? Does your patient need to come in or can you manage over the phone?

012: I’d like them to come in because I’d like to do the UA. I’d prefer them to come in.

J: Did you ever have patients just call in, and how do you deal with that?

012: I know that like, UTIs are pretty miserable so I’m pretty flexible if they’re on vacation, I don’t always make them come in, or if they can’t get off work. Some of them will call the office, the girls will take on a note on their system, sometimes I’ll call them in an antibiotic if i feel it’s appropriate.

Reference 3 - 7.88% Coverage

Do you guys ever use antibiograms to look at antibiotics and antibiotic resistance in your area?

012: No, not usually. We have one, but it’s not something I look at very often.

J: How about using that, do you have any thoughts about how that would be helpful for you guys?

012: It’s definitely helpful if you’re not sure which to give. With respect to UTIs, I don’t use it that much You kinda have to start them on something, so I start them on traditional first line treatment, and then I’ll get a culture to see if I need to change the antibiotic. I don’t know that that would really be helpful in this situation.

<Files\\8_13_18_001> - § 2 references coded [7.14% Coverage]

Reference 1 - 2.63% Coverage

. So can you kind of approach how you would deal with that in a clinic setting if you saw that patient?

001-- so obviously we check a urine dip in the office make sure she is not pregnant. You know if she doesn't have any allergies, check for other medications and usually I'll give a 3-day course of Cipro if I'm not concerned that somebody is pregnant

Reference 2 - 4.51% Coverage

One thing that was helpful is I don't know why our labs we seem to get a lot of these false positive blood in the urine and so they they add it and now it looks like they dropped off doing it they were at they were checking for vitamin C you know to see if it wasn't you know and false positive whatever now it doesn't seem like they're doing that as much anymore so and I try to get more urine cultures if I seem to see people that look like it's a UTI and for leukocytes and then it's not ,then I try to get cultures and actually see if really a UTI or somebody’s got interstitial cystitis or something.

**FORMULATING A UTI TREATMENT PLAN**

Adverse events

<Files\\01_08_19_013> - § 2 references coded [6.07% Coverage]

Reference 1 - 1.49% Coverage

M:What are the other antibiotics you usually think about beyond sulfa and ampicillin?

013: Nitrofurantoin for younger people with good or adequate renal function. Ciprofloxacin for third line.

Reference 2 - 4.58% Coverage

You mentioned fluoroquinolones are usually your last line. What makes you reluctant to use them?

013: Increasing resistance pattern is probably number 1, cost is somewhat a factor, they’re not that expensive. Really up there in the hierarchy of antibiotics use they shouldn’t be used for this, try to reserve them for more resistant infections.

M: Do you see a lot of resistance to trimethaprim sulfa then?

013: In older patients really, in younger patients it’s still a pretty good medicine. Or I’ll say non hospital non medically ill. I’ve got some 40 year olds that are 100 years old physiologically.

<Files\\01_10_19_014> - § 2 references coded [7.44% Coverage]

References 1-2 - 7.44% Coverage

So you mentioned a couple antibiotics. Do you consider certain ones better or worse?

014: I guess, we used to give cipro all the time, and then with all the different considerations about the fluoroquinolones now, we’ve really backed off of those. I don’t really have one that I call on more than others, it really kinda depends on the age and whether I think it’s a recurrent complicated thing or no. So, no.

J: So you just mentioned fluoroquinolones and that you’ve backed off using them. Can you elaborate?

014: Sure, really because with the tendonopathy people can get, and it seems like there were more cardiac concerns. Every time I turned around, it seemed like somebody was saying something bad about the fluoroquinolones. I think it’s just that they seem to be, more of a last resort. Or I do still use them if I feel like somebody, if they have kidney stone history or something that doesn’t just seem like a UTI.

<Files\\02_07_19_015> - § 2 references coded [3.15% Coverage]

Reference 1 - 2.67% Coverage

..you're convinced that it's a urinary tract infection and you're planning on treating her, what antibiotics do you typically go to?

015: Bactrim is the first one.

M: Ok.

015: Keflex if they are allergic to Sulfa.

M: Ok.

015: Keflex. And then if they are allergic to Sulfa and the Penicillin then I try Cipro.

M: Cipro. Ok. Do you feel like some antibiotics work better than others?

015: Yes.

M: Ok.

015: I think that in my experience, Bactrim is better than Keflex. Bactrim and Keflex are better than Cipro.

M: Ok.

015: And for some bacteria, especially the E. Coli.

M: Sure.

015: The E. Coli we're seeing a little more resistance now. We were promised they would not be resistant. Before you were born they said there would never... you would never see resistance. Now it is happening.

M: For sure. Seeing it more and more and more.

Reference 2 - 0.48% Coverage

If they want to do something like….. you can use, let’s say Bactrim or sulfa, and my patient is has allergies to sulfa I would say... I won’t use it, so….

<Files\\08_15_18_002> - § 7 references coded [12.74% Coverage]

Reference 1 - 1.82% Coverage

So we will not call out medicine here at Convenient Care, that will not be done even if you were just seeing for that unless they have a reaction to the medication in which case they will call later that day or the next day and say I have a rash all over I just started this, the only thing that changed I need to change my antibiotic. We will probably change their abx unless there is a new symptom.

References 2-4 - 4.88% Coverage

what are the other choices that you usually think about when u cannot use Bactrim?

002-- I am very fond of Macrobid, also very inexpensive , very well tolerated, test if there's a chance they could be pregnant nice to know that it's safe as beyond that I used to do Cipro but the one of the primaries that I worked with she would do super 250 for 3 days never worked. I would get the calls saying I saw this doctor so an so and I've been taking the antibiotic and it didn't get better It didn't change and I look in the chart its Cipro 250th 3 days it didn't work, so I would do Cipro 500 but usually for 5 to 7 days but then this whole thing with the fluoroquinolones in the Black Box warning and then and now try to stay away from any of the fluoroquinolones if I can. So ironically especially if they've already done bactrim or Macrobid go to one of the cephalosporins or even amoxicillin not the best choice but still would hit a lot of the possible bacteria and again very well tolerated very inexpensive usually works pretty well not my first choice but it's an option

Reference 5 - 1.30% Coverage

I used to use a lot of Cipro. Cipro is good for older guys because of prostatitis or anything more than just a typical UTI if it was anything else Cipro the nice option and I still may even go to that on occasion but yeah the Black Box warning for the FQ really did back my use of cipro

Reference 6 - 3.51% Coverage

as far as resources honestly Dr Morris? is a good resource. He stays on top of everything and he does research , he has articles and they have all kinds of stuff and especially when I moved over here there were a lot of I was set in my ways for antibiotics as a PCP I usually have my same ones for this and this is for UTIs. came over here and people would be allergic to Cipro and Macrobid and Bactrim and I'm like what in the world do you use and he's like well… and so he's a huge resource and Cassie and Gina the nurse practitioners they've been doing acute care even longer than I have so you know I consider my acute care started back in January when I moved over here so their resources as well so I'll forget certain antibiotics so and I realize I could use those.

Reference 7 - 1.23% Coverage

just so many unknowns and so many questions that you can't say you strep is going to be treated by amoxicillin because they're even him for allergic to Amoxicillin you're still not going to do azithromax everytime and people don't like that azithromax for lots of reasons.

<Files\\08_15_18_003> - § 3 references coded [8.82% Coverage]

References 1-2 - 4.61% Coverage

when you're seeing the patient and you won't have the culture results cuz you'll be treating them kind of empirically so what what do you prefer usually when you're when you're seeing them here?

003-- of course for non-pregnant non-nursing moms I want to make sure I use the antibiotics that are safe so let's assume it's that case so I might start Keflex or macrobid or sulfa. Those are my top three unless they say I can’t tolerate that, if they had a rash before, nervous about taking a medication so if there's no contraindications to use then those three and perhaps over the years and I think I probably use Cipro a lot more early on but changed over the years with literature coming out saying maybe we need to use this or seeing more cultures that said was seeing a lot of resistance for this than other. And sometimes it's just kind of like people talking or clinicians talking like seeing or trending a lot more resistance to this and we ought to be using cephalexin for the first line or maybe need to get away from the quinolones or watch out for the sulfa reaction. do you want to know about maybe other…….

Reference 3 - 4.21% Coverage

the FDA black box warning? Did that change our decisions at all or not some people that can influence them some people in a left so if some of the side effects they mentioned in the black box yet and they're pretty low too?

003---they are so yeah they influenced patients calling back and saying ….maybe retrospect maybe it did influence me I didn’t want to get that call back and asking me to switch because they didn’t want their tendons to rupture so maybe looking back maybe that did it have some influence on ya on me but more because of the patients are going to read it and call/come back. So as far as that on the quinolones the only thing I was noticing too maybe I'll ask you later when we're done is that the lower dose of the quinolones were failing, like the 250s for minor UTI that is 250 BID for five days and many came back with recurrence and the same with Levaquin 250. One of the Levaquin reps told us we don’t want to talk about levoquin 250 anymore, so maybe those are some influences over the years.

<Files\\08_15_18_004> - § 5 references coded [12.92% Coverage]

References 1-2 - 3.41% Coverage

--So it is that usually when you say your go to antibiotic or do you are there certain characteristics or things that change your mind about which antibiotic you should prescribed for the patient.

004—Cipro or Macrobid and thats based on allergies you know And that's pretty much what we use

M--And then how do you decide between the two?

004—I would confess it is largely random unless they've recently been treated with Cipro I would probably give that first, in young patient in a community the resistance is so high.

References 3-4 - 6.57% Coverage

That's wonderful I'm happy to hear that. So you mentioned historically that you kind of shifted from Septra to Cipro and now more Macrobid antibiotic prescribing… what what's influenced your decisions in terms of your evolution about selecting specific antibiotics?

004—When I started out there was no Cipro there was only Septra. Cipro, levoquin came along and they were better tolerated and carried a little less risk in terms of kidney and no hyperkalemia and the other stuff, allergic reactions. And then more recently using a lot of macrobid. So you asked about a young patient I would definitely use the nitrofurantoin and in older population certainly in a nursing home population because of the high incidence of Cipro or quinolone resistance that we are seeing.I don't have a statistic but we see it a lot.So we've got using a lot more nitrofurantoin.

M--What about the Septra ? You see a lot of resistance to Septra too?.

004--There's resistance to septra, we just moved away from using it much.

Reference 5 - 2.94% Coverage

The only difference I would say is treating older patients with UTI, Itss a different ball point. But that's not really what you're asking its more about young people with uncomplicated bladder infections its pretty straightforward.

M—The older ones can be a little more challenging I guess related to creatinine clearance and other things taking that into account, other medications, QT prolonging drugs etc.

004—Or sometimes not having symptoms.

<Files\\08_23_18_005> - § 5 references coded [8.99% Coverage]

References 1-2 - 3.45% Coverage

Yeah. And then you mentioned that you like to use macrobid as your first line agent. Can you tell me what your thoughts are about different antibiotics, and why you like macrobid compared to others?

005: I just feel like macrobid has a lower side effect profile, less people are allergic to macrobid for some reason, cause there's no other drugs in that class, I feel like unless they’re menopausal, after 50, I feel like most are not resistant to it and it’s just kinda the go -to as far as

evidence-based practice from when I was in school that was kinda just what we were taught, especially with cipro, and their recent side effects and the black box warning and stuff, we’ve kind of stayed away from cipro, but I feel like a lot of our practitioners still use it. I just go with macrobid, morning and night for five to seven days, depends on the severity of symptoms, if they’ve had one recently.

References 3-5 - 5.54% Coverage

And then what other antibiotics come to mind for you other than cipro and macrobid, are those the two main ones?

005: Bactrim, double strength, we’ve used a lot. I mean we can use like a cephalosporin if they’ve shown resistance to something in the past. Generally that would be like a last case, or like a keflex, just depends on allergies, like if somebody has an allergy to bactrim, macrobid hasn’t worked for them in the past, we’re going cipro, we’re going keflex, it just kinda depends. Or if they’ve had any past culture that have shown resistance, gotta steer clear of the ones they’ve been resistant to and I just kinda have to go down the list and see on the culture what its been susceptible to in the past.

M: Got it, so they have like an intolerance to macrobid, but they’re okay with everything else, do you have a second line agent that you like to use?

005: Generally I go with Bactrim double strength. I don’t, like, no idea why I’ve just gotten good coverage over the years and nobody’s called back and said like hey this hasn’t worked. And then once again a lot of patients see the cipro warnings when the pharmacist goes over the side effects with them we get a lot of calls back for cipro from pharmacists like “hey do you realize you put them on cipro” and then the patient calls back with a lot of questions. Bactrim double strength generally doesn’t give me that kickback. We don’t get a lot of phone calls back for it.

<Files\\08_23_18_005 (2)> - § 2 references coded [8.99% Coverage]

Reference 1 - 3.45% Coverage

Yeah. And then you mentioned that you like to use macrobid as your first line agent. Can you tell me what your thoughts are about different antibiotics, and why you like macrobid compared to others?

005: I just feel like macrobid has a lower side effect profile, less people are allergic to macrobid for some reason, cause there's no other drugs in that class, I feel like unless they’re menopausal, after 50, I feel like most are not resistant to it and it’s just kinda the go -to as far as

evidence-based practice from when I was in school that was kinda just what we were taught, especially with cipro, and their recent side effects and the black box warning and stuff, we’ve kind of stayed away from cipro, but I feel like a lot of our practitioners still use it. I just go with macrobid, morning and night for five to seven days, depends on the severity of symptoms, if they’ve had one recently.

Reference 2 - 5.55% Coverage

And then what other antibiotics come to mind for you other than cipro and macrobid, are those the two main ones?

005: Bactrim, double strength, we’ve used a lot. I mean we can use like a cephalosporin if they’ve shown resistance to something in the past. Generally that would be like a last case, or like a keflex, just depends on allergies, like if somebody has an allergy to bactrim, macrobid hasn’t worked for them in the past, we’re going cipro, we’re going keflex, it just kinda depends. Or if they’ve had any past culture that have shown resistance, gotta steer clear of the ones they’ve been resistant to and I just kinda have to go down the list and see on the culture what its been susceptible to in the past.

M: Got it, so they have like an intolerance to macrobid, but they’re okay with everything else, do you have a second line agent that you like to use?

005: Generally I go with Bactrim double strength. I don’t, like, no idea why I’ve just gotten good coverage over the years and nobody’s called back and said like hey this hasn’t worked. And then once again a lot of patients see the cipro warnings when the pharmacist goes over the side effects with them we get a lot of calls back for cipro from pharmacists like “hey do you realize you put them on cipro” and then the patient calls back with a lot of questions. Bactrim double strength generally doesn’t give me that kickback. We don’t get a lot of phone calls back for it.

<Files\\08_23_18_006> - § 4 references coded [12.17% Coverage]

References 1-2 - 4.14% Coverage

1. Got it. So you mention macrobid, that's gonna be your first line antibiotic. Can you tell me your thoughts on different antibiotics and why you prefer macrobid for patients?
2. I like macrobid because I know it’s safe in pregnancy, and I know that it’s safe for most age ranges. I know that it’s really good for urine stuff, and it’s not gonna be one of those that I run into too often with people with allergies. I have had a couple claim it, I don’t like reinventing the wheel so if I know it works well, it’s what I’m gonna go with.
3. Got it. Do you think there’s any downsides with macrobid compared to other antibiotics with UTIs?
4. I haven’t run into it, but that doesn’t mean they don’t exist.

References 3-4 - 8.03% Coverage

1. If you’re thinking about alternatives or other agents, what are the other antibiotics you usually think of?
2. Keflex, I’ll do Keflex 4 times a day for 10 days. Sometimes I’ll use bactrim, that one does

seem to come up more often as one that has resistance so I try to do keflex over it but if I look at somebody and they tell me they’re not gonna take it 4 times a day, okay bactrim it is. I don’t do cipro unless I don’t feel that there’s any other option.

1. Got it. And why do you avoid cipro?
2. It has a black box warning, I don’t like to prescribe things like that if I don’t have to.

1. And is it, why does the black box warning bother you?
2. Because then you get people that get home, they read the side effects, and now they have it, and now it’s your fault. There’s a lot of public distrust of it, and I understand that. I also don’t like the idea, if I start with that where do I go if it doesn’t work?
3. Got it. So if someone’s not being treated with macrobid, or you feel like that’s not effective, then what’s the next antibiotic you go with?
4. If i don’t do macrobid it’ll be the keflex and then the bactrim is kind of my way, and then I’ve done Levaquin a couple times, because people will come in and insist on it. I don’t know why they do that, but they swear that’s what they got the last time and the only thing that works for them.

<Files\\09_05_18_007> - § 3 references coded [5.93% Coverage]

Reference 1 - 1.20% Coverage

1. I usually use either bactrim DS, if there’s no allergy of course, 1 tablet twice a day. I usually do 5 day course, there are times I tell patients to use it 3 days and their symptoms have subsided within 48 hours they can do a short course of 3 days, but 5 days is what I send the patient a prescription. My second choice is macrobid, twice a day for 5 days.

Reference 2 - 1.79% Coverage

1. Then you mentioned cipro as one of the preferred antibiotics. Do they have antibiotics that they recommend, and if so what do they usually say?
2. The preferred antibiotic in my experience in talking to the nurses is most of the time cipro, and patients have taken it in the past for these things, and they say that. I don’t hear a lot about macrobid, unfortunately, I don’t know why. We have a lot of patients who are elderly in our practice, so the bactrim is not a favored choice. We hear a lot about cipro and the next is macrobid.

Reference 3 - 2.95% Coverage

1. RIght, so, you know for the most part we know patients who are low risk without multi-drug resistant infection, like simple cystitis, e.coli is the most common, I personally think that my choice is bactrim, nitrofurantoin, it should take care of them. Either I use nitrofurantoin or bactrim, in terms of which one is my favorite, I guess I think it’s all about allergies, if there’s an allergy I don’t prescribe bactrim obviously. I have not looked into, honestly, if bactrim resistance or e coli is more than nitrofurantoin. I have a habit of looking back, even in young patients, if they ever had a cultures in the system, if I have time to pull that up and see if there is any bacteria that they had a culture for an unknown reason and what was the sensitivity of that. Sometimes I do that, but either both of them is my first choice, not sure which one is more sensitive to e coli.

<Files\\10_10_18_008> - § 1 reference coded [2.68% Coverage]

Reference 1 - 2.68% Coverage

And then, you mentioned that you use empiric antibiotics. Which ones do you typically like to use and why?

8: I would typically use Cipro or Bactrim. I believe that that Bactrim probably has a better E coli coverage currently than Cipro and Cipro has some nasty side effects. I've actually given someone an achilles tendon rupture, but those would be my two initial thoughts.

<Files\\10_10_18_008 (2)> - § 1 reference coded [5.87% Coverage]

Reference 1 - 5.87% Coverage

you mentioned that you use empiric antibiotics. Which ones do you typically like to use and why?

8: I would typically use Cipro or Bactrim. I believe that that Bactrim probably has a better E coli coverage currently than Cipro and Cipro has some nasty side effects. I've actually given someone an achilles tendon rupture, but those would be my two initial thoughts.

M: Got it. And when you're trying to decide between those two, do you think one is stronger than the other or do you think, you mentioned some of the side effects with Cipro that you get worried about. So do you avoid one or some of the antibiotics over the other one, based on, certain patient factors, or what goes into your decision making about the antibiotic? And it's okay if you tell me, you know, and it's completely arbitrary too. That's fine as well.

<Files\\11_14_18_009> - § 2 references coded [1.57% Coverage]

References 1-2 - 1.57% Coverage

is there anything that makes you lean one way or the other?

009: Um, you know uh if they have no allergies or anything, not really. I think it’s just how much it’s gonna cost a patient and if they’ve taken something before that they’ve tolerated well.

<Files\\12_18_18_010> - § 2 references coded [7.15% Coverage]

Reference 1 - 1.56% Coverage

if I’m convinced she has a UTI, we’ll definitely send for culture as well but put her on 3 days of therapy, oral therapy twice a day, maybe septra, depending on her allergies of course.

Reference 2 - 5.59% Coverage

You mentioned giving 3 days of Septra for this patient once you get the test results, are there any other antibiotics that you would choose?

010: I would start either Macrobid or Septra depending on their allergies. I would not reach for Cipro right away, especially if its somebody who doesn’t have a history of recurrence. She’s got a short history of it, it doesn’t sound like. If her dip is negative, I wouldn’t start her on an antibiotic, I’d say to her we’re just gonna wait for the culture to come back. BUt, i wouldn’t use Cipro or anything like that to start out, i’d use septra or macrobid, maybe keflex, especially if she’s naive to antibiotics.

<Files\\12_19_18_011> - § 2 references coded [5.48% Coverage]

References 1-2 - 5.48% Coverage

Okay, would you choose to treat with antibiotics?

011: Depends on the UA.

J: If it shows that she does have a UTI, which would you choose and what duration?

011: Most of the time, if it’s, the only symptom is dysuria and UA is positive and no allergies, ether keflex or cipro. Usually ask what works best for them, because the majority of patients I see, it’s not their first UTI. And so those are the main 2 that i go with. If there’s allergies then sometimes bactrim. Majority of the time treatment is 3-5 days depending on their past history and severity of symptoms.

<Files\\12_20_18_012> - § 3 references coded [9.83% Coverage]

References 1-2 - 4.09% Coverage

Are there certain antibiotics which you consider better or worse than others?

012: It depends, it they’re having recurrent UTIs then I’d get a culture and it would tell you which antibiotic is better for that strain of bacteria, but I’d usually go with bactrim or cipro or possibly macrobid depending on drug allergies.

Reference 3 - 5.74% Coverage

if certain patients have certain risk factors, would you treat them differently?

012: Yeah absolutely. If there’s a nursing home patient, or that population that seems to get UTIs more, I definitely would treat them differently.

J: Do you consider different antibiotics for patients who have different risk factors?

012: For like older patients, you have to know what their kidney function looks like before you can describe certain antibiotics.

<Files\\8_13_18_001> - § 4 references coded [10.49% Coverage]

Reference 1 - 2.63% Coverage

. So can you kind of approach how you would deal with that in a clinic setting if you saw that patient?

001-- so obviously we check a urine dip in the office make sure she is not pregnant. You know if she doesn't have any allergies, check for other medications and usually I'll give a 3-day course of Cipro if I'm not concerned that somebody is pregnant

References 2-3 - 3.80% Coverage

you generally do Cipro, do you have a preference or why do you do Cipro over antibiotics?

001—I will use Macrobid if it's somebody that has drug interactions or I'm worried they're pregnant. I really don't use sulfa a whole lot just because there's so many people with allergies and I have had a lot of cases in the past year of older women they go to the Urgent Care and get sulfa and I'm not kidding I've had 5 people end up in the hospital with AKI or kidney failure, so for older women I just don't like it.

Reference 4 - 4.06% Coverage

when people are covering or exchanges sending in antibiotics or something, the drug interactions popped up automatically and now that doesn't happen you have to click on new interactions to see, so will frequently see people get sulfa, Cipro and there's probably at least of possibly serious drug interaction and nobody looks at that because it doesn't pop up automatically anymore so I think that's another thing people really need to be cognizant of is always clicking that button because there's a lot of people who can't take these things.

Antibiotic coverage

<Files\\01_08_19_013> - § 7 references coded [18.12% Coverage]

Reference 1 - 1.71% Coverage

Low level symptoms no fever, no risk of sepsis, mildly irritating symptoms

M:Then what antibiotics do you prefer to use in this circumstance?

013: I would try and use sulfa drug or ampicillin first, escalate up as needed.

References 2-3 - 3.15% Coverage

What are the other antibiotics you usually think about beyond sulfa and ampicillin?

013: Nitrofurantoin for younger people with good or adequate renal function. Ciprofloxacin for third line.

M: What makes you choose one agent over another one?

013: I’ll try and do simple medicines first. Sulfa is sort of an old stand by. It can be so much resistance to ciprofloxacin, it’s a more potent medicine theoretically.

References 4-5 - 4.58% Coverage

You mentioned fluoroquinolones are usually your last line. What makes you reluctant to use them?

013: Increasing resistance pattern is probably number 1, cost is somewhat a factor, they’re not that expensive. Really up there in the hierarchy of antibiotics use they shouldn’t be used for this, try to reserve them for more resistant infections.

M: Do you see a lot of resistance to trimethaprim sulfa then?

013: In older patients really, in younger patients it’s still a pretty good medicine. Or I’ll say non hospital non medically ill. I’ve got some 40 year olds that are 100 years old physiologically.

Reference 6 - 5.78% Coverage

In hospital setting we use antibiograms, in clinic settings I never see that being used. One of the thoughts I’m kicking around, and I’d like your thoughts on it, do you think antibiograms should be incorporated in outpatient settings, do you think it’s appropriate? Some of the guidelines use cutoff points for when a firstline antibiotic should be used based on local susceptibilities. Is that info ever sent to you, are you aware of local susceptibilities?

013: If i were just office setting we would have some trouble some trouble. I think it’s more about resistant infections, I don’t how much an antibiogram would change my opinion about what abx I’m using for a UTI, because you can always get a culture. For a respiratory infection, that’s a little different.

Reference 7 - 2.90% Coverage

That’s true, it might help with empiric antibiotic selection before culture are back if u think they need it.

013: I know there’s a lot of resistant to sulfa and ampicillin basically because that’s what I should be using to start with.

M: That will be the goal. None of the outpatient providers I talked to are aware of antibiograms. I am not sure that should be shared or not.

<Files\\01_10_19_014> - § 1 reference coded [7.15% Coverage]

Reference 1 - 7.15% Coverage

Do you use any specific resources or guidelines when picking antibiotics and treatment duration?

014: Because it’s easy on epic to get to up date, i look at what they’ve got. I used to use the Sanford guide, but my exposure to lots of different stuff is not there. I’m not an infectious disease person, I don’t have all those bacterias like you guys do. So havent used Stanford too much. It just didn’t seem necessary. I will use up to date, that’s the one I really go with.

J: Have you ever used antibiograms to study local resistance in your area?

014: No I haven’t.

J: Do you think that would be a good resource if that was made easily available?

014: I do, when you read up to date, we have a lot of strep throat, they always say try this, but if you have local resistance. I do see a fair amount of STDs, and I don’t know the local resistance. I think that would be wonderful.

<Files\\02_07_19_015> - § 2 references coded [4.24% Coverage]

Reference 1 - 2.79% Coverage

..you're convinced that it's a urinary tract infection and you're planning on treating her, what antibiotics do you typically go to?

015: Bactrim is the first one.

M: Ok.

015: Keflex if they are allergic to Sulfa.

M: Ok.

015: Keflex. And then if they are allergic to Sulfa and the Penicillin then I try Cipro.

M: Cipro. Ok. Do you feel like some antibiotics work better than others?

015: Yes.

M: Ok.

015: I think that in my experience, Bactrim is better than Keflex. Bactrim and Keflex are better than Cipro.

M: Ok.

015: And for some bacteria, especially the E. Coli.

M: Sure.

015: The E. Coli we're seeing a little more resistance now. We were promised they would not be resistant. Before you were born they said there would never... you would never see resistance. Now it is happening.

M: For sure. Seeing it more and more and more.

015: Yeah, so I don't like the quinolones

Reference 2 - 1.45% Coverage

how do you think they should be shared with primary care doctors?

015: I think they should. It would help a lot because a lot of times when the patient comes to you ___ 13:25 and unfortunately before the trend of the antibiotics… to a multi resistant antibiotic resistant infection, we just gave the antibiotic because the patient wanted it and it has been very difficult now to show the patient that you don't have to get antibiotics every time that you want it.

<Files\\08_15_18_002> - § 2 references coded [6.87% Coverage]

Reference 1 - 2.00% Coverage

if they don't have a PCP but in that case someone who has no medical problems 2 days of symptoms. that urine dip, the UA comes back showing leukocytes, nitrites, even trace of blood but I would probably still then that is probably give Bactrim it's inexpensive it's well tolerated it usually works for most people so it makes good sense in all the cultures most of the cultures we had have come back sensitive to bactrim so it's nice and easy.

Reference 2 - 4.86% Coverage

what are the other choices that you usually think about when u cannot use Bactrim?

002-- I am very fond of Macrobid, also very inexpensive , very well tolerated, test if there's a chance they could be pregnant nice to know that it's safe as beyond that I used to do Cipro but the one of the primaries that I worked with she would do super 250 for 3 days never worked. I would get the calls saying I saw this doctor so an so and I've been taking the antibiotic and it didn't get better It didn't change and I look in the chart its Cipro 250th 3 days it didn't work, so I would do Cipro 500 but usually for 5 to 7 days but then this whole thing with the fluoroquinolones in the Black Box warning and then and now try to stay away from any of the fluoroquinolones if I can. So ironically especially if they've already done bactrim or Macrobid go to one of the cephalosporins or even amoxicillin not the best choice but still would hit a lot of the possible bacteria and again very well tolerated very inexpensive usually works pretty well not my first choice but it's an option

<Files\\08_15_18_003> - § 3 references coded [8.77% Coverage]

Reference 1 - 4.16% Coverage

you are really convinced or you're really suspicious about the UTI diagnosis does that change your practices for whether you are going to test them or get a culture or do you always regardless of your kind of pretest probability you always get a urine test?

003-- it in my mind in my mind I'm taking okay this is pretty straightforward but we still do it, yeah in this setting, because if for some reason there is antibiotic resistance then we can go back to the …okay this is what we ordered we got to change the antibiotics and then perhaps not going to work and maybe that's why you r not getting better. And since it's a seven-days-a-week operation I may not be here and I dont know the whole story. Someone might be able look at my chart and kinda see what is going on with it, it may be a better safety net if you want to use that term, making sure that the proprietors here when I'm not here they can just easily look at the the culture and say oh he prescribed Cipro and then can change to bactrim.

References 2-3 - 4.61% Coverage

when you're seeing the patient and you won't have the culture results cuz you'll be treating them kind of empirically so what what do you prefer usually when you're when you're seeing them here?

003-- of course for non-pregnant non-nursing moms I want to make sure I use the antibiotics that are safe so let's assume it's that case so I might start Keflex or macrobid or sulfa. Those are my top three unless they say I can’t tolerate that, if they had a rash before, nervous about taking a medication so if there's no contraindications to use then those three and perhaps over the years and I think I probably use Cipro a lot more early on but changed over the years with literature coming out saying maybe we need to use this or seeing more cultures that said was seeing a lot of resistance for this than other. And sometimes it's just kind of like people talking or clinicians talking like seeing or trending a lot more resistance to this and we ought to be using cephalexin for the first line or maybe need to get away from the quinolones or watch out for the sulfa reaction. do you want to know about maybe other…….

<Files\\08_15_18_004> - § 6 references coded [11.54% Coverage]

References 1-2 - 1.56% Coverage

Then you mentioned you like to treat for about 5 to 7 days… what antibiotic do you usually like to give?

004—for years it was Septra ds and then it was Cipro and now there's so much resistance to antibiotics we often go to Nitrofuratoin.

References 3-4 - 3.41% Coverage

--So it is that usually when you say your go to antibiotic or do you are there certain characteristics or things that change your mind about which antibiotic you should prescribed for the patient.

004—Cipro or Macrobid and thats based on allergies you know And that's pretty much what we use

M--And then how do you decide between the two?

004—I would confess it is largely random unless they've recently been treated with Cipro I would probably give that first, in young patient in a community the resistance is so high.

References 5-6 - 6.57% Coverage

That's wonderful I'm happy to hear that. So you mentioned historically that you kind of shifted from Septra to Cipro and now more Macrobid antibiotic prescribing… what what's influenced your decisions in terms of your evolution about selecting specific antibiotics?

004—When I started out there was no Cipro there was only Septra. Cipro, levoquin came along and they were better tolerated and carried a little less risk in terms of kidney and no hyperkalemia and the other stuff, allergic reactions. And then more recently using a lot of macrobid. So you asked about a young patient I would definitely use the nitrofurantoin and in older population certainly in a nursing home population because of the high incidence of Cipro or quinolone resistance that we are seeing.I don't have a statistic but we see it a lot.So we've got using a lot more nitrofurantoin.

M--What about the Septra ? You see a lot of resistance to Septra too?.

004--There's resistance to septra, we just moved away from using it much.

<Files\\08_23_18_005> - § 2 references coded [6.20% Coverage]

Reference 1 - 3.45% Coverage

Yeah. And then you mentioned that you like to use macrobid as your first line agent. Can you tell me what your thoughts are about different antibiotics, and why you like macrobid compared to others?

005: I just feel like macrobid has a lower side effect profile, less people are allergic to macrobid for some reason, cause there's no other drugs in that class, I feel like unless they’re menopausal, after 50, I feel like most are not resistant to it and it’s just kinda the go -to as far as

evidence-based practice from when I was in school that was kinda just what we were taught, especially with cipro, and their recent side effects and the black box warning and stuff, we’ve kind of stayed away from cipro, but I feel like a lot of our practitioners still use it. I just go with macrobid, morning and night for five to seven days, depends on the severity of symptoms, if they’ve had one recently.

Reference 2 - 2.75% Coverage

And then what other antibiotics come to mind for you other than cipro and macrobid, are those the two main ones?

005: Bactrim, double strength, we’ve used a lot. I mean we can use like a cephalosporin if they’ve shown resistance to something in the past. Generally that would be like a last case, or like a keflex, just depends on allergies, like if somebody has an allergy to bactrim, macrobid hasn’t worked for them in the past, we’re going cipro, we’re going keflex, it just kinda depends. Or if they’ve had any past culture that have shown resistance, gotta steer clear of the ones they’ve been resistant to and I just kinda have to go down the list and see on the culture what its been susceptible to in the past.

<Files\\08_23_18_005 (2)> - § 1 reference coded [3.45% Coverage]

Reference 1 - 3.45% Coverage

Yeah. And then you mentioned that you like to use macrobid as your first line agent. Can you tell me what your thoughts are about different antibiotics, and why you like macrobid compared to others?

005: I just feel like macrobid has a lower side effect profile, less people are allergic to macrobid for some reason, cause there's no other drugs in that class, I feel like unless they’re menopausal, after 50, I feel like most are not resistant to it and it’s just kinda the go -to as far as

evidence-based practice from when I was in school that was kinda just what we were taught, especially with cipro, and their recent side effects and the black box warning and stuff, we’ve kind of stayed away from cipro, but I feel like a lot of our practitioners still use it. I just go with macrobid, morning and night for five to seven days, depends on the severity of symptoms, if they’ve had one recently.

<Files\\08_23_18_006> - § 2 references coded [8.12% Coverage]

References 1-2 - 8.12% Coverage

1. Got it, alright. If you’re thinking about alternatives or other agents, what are the other antibiotics you usually think of?
2. Keflex, I’ll do Keflex 4 times a day for 10 days. Sometimes I’ll use bactrim, that one does

seem to come up more often as one that has resistance so I try to do keflex over it but if I look at somebody and they tell me they’re not gonna take it 4 times a day, okay bactrim it is. I don’t do cipro unless I don’t feel that there’s any other option.

1. Got it. And why do you avoid cipro?
2. It has a black box warning, I don’t like to prescribe things like that if I don’t have to.
3. And is it, why does the black box warning bother you?
4. Because then you get people that get home, they read the side effects, and now they have it, and now it’s your fault. There’s a lot of public distrust of it, and I understand that. I also don’t like the idea, if I start with that where do I go if it doesn’t work?
5. Got it. So if someone’s not being treated with macrobid, or you feel like that’s not effective, then what’s the next antibiotic you go with?
6. If i don’t do macrobid it’ll be the keflex and then the bactrim is kind of my way, and then I’ve done Levaquin a couple times, because people will come in and insist on it. I don’t know why they do that, but they swear that’s what they got the last time and the only thing that works for them.

<Files\\09_05_18_007> - § 2 references coded [5.49% Coverage]

Reference 1 - 4.26% Coverage

1. Do you have any specific opinions on which ones you prefer when, do you think certain antibiotics are stronger than others, or tend to work better than others in terms of your historical practices? I know that there’s some concern about increasing resistance and things like that, so some providers tell me that resistance rates play a role. What are your thoughts about, how do you decide?
2. RIght, so, you know for the most part we know patients who are low risk without multi-drug resistant infection, like simple cystitis, e.coli is the most common, I personally think that my choice is bactrim, nitrofurantoin, it should take care of them. Either I use nitrofurantoin or bactrim, in terms of which one is my favorite, I guess I think it’s all about allergies, if there’s an allergy I don’t prescribe bactrim obviously. I have not looked into, honestly, if bactrim resistance or e coli is more than nitrofurantoin. I have a habit of looking back, even in young patients, if they ever had a cultures in the system, if I have time to pull that up and see if there is any bacteria that they had a culture for an unknown reason and what was the sensitivity of that. Sometimes I do that, but either both of them is my first choice, not sure which one is more sensitive to e coli.

Reference 2 - 1.23% Coverage

1. And what are your thoughts about a patient with multidrug resistant UTI who is simply coming with a simple symptom not generalized symptoms or red flags. There are some infections where it’s okay to treat them, with 10 days of whatever sensitivity they have, or there are some infections doctors that no, it treated be treated with antibiotics, preferably meropenem.

<Files\\10_10_18_008> - § 1 reference coded [2.68% Coverage]

Reference 1 - 2.68% Coverage

And then, you mentioned that you use empiric antibiotics. Which ones do you typically like to use and why?

8: I would typically use Cipro or Bactrim. I believe that that Bactrim probably has a better E coli coverage currently than Cipro and Cipro has some nasty side effects. I've actually given someone an achilles tendon rupture, but those would be my two initial thoughts.

<Files\\10_10_18_008 (2)> - § 1 reference coded [5.87% Coverage]

Reference 1 - 5.87% Coverage

you mentioned that you use empiric antibiotics. Which ones do you typically like to use and why?

8: I would typically use Cipro or Bactrim. I believe that that Bactrim probably has a better E coli coverage currently than Cipro and Cipro has some nasty side effects. I've actually given someone an achilles tendon rupture, but those would be my two initial thoughts.

M: Got it. And when you're trying to decide between those two, do you think one is stronger than the other or do you think, you mentioned some of the side effects with Cipro that you get worried about. So do you avoid one or some of the antibiotics over the other one, based on, certain patient factors, or what goes into your decision making about the antibiotic? And it's okay if you tell me, you know, and it's completely arbitrary too. That's fine as well.

<Files\\12_18_18_010> - § 1 reference coded [4.34% Coverage]

Reference 1 - 4.34% Coverage

I would start either Macrobid or Septra depending on their allergies. I would not reach for Cipro right away, especially if its somebody who doesn’t have a history of recurrence. She’s got a short history of it, it doesn’t sound like. If her dip is negative, I wouldn’t start her on an antibiotic, I’d say to her we’re just gonna wait for the culture to come back. BUt, i wouldn’t use Cipro or anything like that to start out, i’d use septra or macrobid, maybe keflex, especially if she’s naive to antibiotics.

<Files\\8_13_18_001> - § 2 references coded [2.62% Coverage]

References 1-2 - 2.62% Coverage

Do u have a sense about some abx being more potent than others or when you compare Macrobid and Cipro do you have any.

001- Well I think there is some resistance to Macrobid or obviously it takes a longer course so I would use that second choice but I don't obviously and possibly pregnant women or older people can't take Cipro for the medications.

Antibiotic selection

<Files\\01_08_19_013> - § 4 references coded [13.74% Coverage]

Reference 1 - 3.11% Coverage

do you ever prescribe antibiotics kind of, do you wait ever to prescribe Abx while you’re waiting for the culture to come back? It sounds like you do, what circumstances is that?

013: Low level symptoms no fever, no risk of sepsis, mildly irritating symptoms

M:Then what antibiotics do you prefer to use in this circumstance?

013: I would try and use sulfa drug or ampicillin first, escalate up as needed.

Reference 2 - 3.15% Coverage

What are the other antibiotics you usually think about beyond sulfa and ampicillin?

013: Nitrofurantoin for younger people with good or adequate renal function. Ciprofloxacin for third line.

M: What makes you choose one agent over another one?

013: I’ll try and do simple medicines first. Sulfa is sort of an old stand by. It can be so much resistance to ciprofloxacin, it’s a more potent medicine theoretically.

Reference 3 - 4.58% Coverage

You mentioned fluoroquinolones are usually your last line. What makes you reluctant to use them?

013: Increasing resistance pattern is probably number 1, cost is somewhat a factor, they’re not that expensive. Really up there in the hierarchy of antibiotics use they shouldn’t be used for this, try to reserve them for more resistant infections.

M: Do you see a lot of resistance to trimethaprim sulfa then?

013: In older patients really, in younger patients it’s still a pretty good medicine. Or I’ll say non hospital non medically ill. I’ve got some 40 year olds that are 100 years old physiologically.

Reference 4 - 2.90% Coverage

That’s true, it might help with empiric antibiotic selection before culture are back if u think they need it.

013: I know there’s a lot of resistant to sulfa and ampicillin basically because that’s what I should be using to start with.

M: That will be the goal. None of the outpatient providers I talked to are aware of antibiograms. I am not sure that should be shared or not.

<Files\\01_10_19_014> - § 4 references coded [26.53% Coverage]

Reference 1 - 6.64% Coverage

How will you handle the case?

014: With somebody like that, we do the Udip in the office. If there’s anything that seems generally positive, leukocytes or nitrates or anything like that, we’ll go ahead and treat them, if it’s uncomplicated and they don’t do it all the time, I usually do 3-5 days of macrobid. It kinda depends, I stick more towards 5. It might be overkill, but since they usually aren’t in my setting, we’re primary care but we also do walk in, and we’re in a tourist area so we get people we’re never gonna see again. We kinda wanna send them off adequately set up, and then we’ll send that urine for culture. FOr everybody that has insurance we send the culture, sometimes if they don’t we give them the choice. Otherwise we just go with it and tell them to follow up if they don’t have resolution of symptoms.

Reference 2 - 2.05% Coverage

You mentioned macrobid, are there other antibiotics that you consider?

014: I do, usually it’s between macrobid and bactrim for those that age group. You said 27? Yeah, those 2 for that age group, especially if they don’t have a lot of recurrent UTIs.

Reference 3 - 7.44% Coverage

So you mentioned a couple antibiotics. Do you consider certain ones better or worse?

014: I guess, we used to give cipro all the time, and then with all the different considerations about the fluoroquinolones now, we’ve really backed off of those. I don’t really have one that I call on more than others, it really kinda depends on the age and whether I think it’s a recurrent complicated thing or no. So, no.

J: So you just mentioned fluoroquinolones and that you’ve backed off using them. Can you elaborate?

014: Sure, really because with the tendonopathy people can get, and it seems like there were more cardiac concerns. Every time I turned around, it seemed like somebody was saying something bad about the fluoroquinolones. I think it’s just that they seem to be, more of a last resort. Or I do still use them if I feel like somebody, if they have kidney stone history or something that doesn’t just seem like a UTI.

Reference 4 - 10.40% Coverage

Patient factors, symptoms, age, sex, etc

014: I think I’ll always, even if I don’t have the culture and the Udip is kinda eh, means to me the leuks are trace. If they’re my age and up, maybe even 55, because those sensors don’t work as well and you don’t get as much dysuria, but you can really get sick. If they act sick I’ll treat them. Even if they kinda don’t act sick, but that looks like a UTI even if I don’t have all the information. For a younger group, if it doesn’t look like a UTI and they’re sexually active and have things that could be more irritants, cause younger women have just more stuff going on. They’re exercising, they're wearing tight clothes. I’ll tell them I wanna wait until the culture is back, unless they have a fever. Sometimes, I see under 21s, so with that crowd if they have a fever I’ll start them on something.

J: How about males and females?

014: I get so so few males with UTIs in this current practice that I kind of forget about them. When I was dealing more with older adults, I’d get what sounded like a UTI but probably was prostatitis. I’d give them cipro, but I’d probably have to look it up now. It seems like in my memory the males that complained of something usually acted like they needed medicine and we just went ahead and took care of it.

<Files\\02_07_19_015> - § 6 references coded [5.72% Coverage]

Reference 1 - 1.68% Coverage

And it's a young person. First time maybe. We don't even know what it is exactly. We think that it's uncomplicated and so if I have a urinalysis that sort of, changes her mind, I could talk to her into just waiting to see what happens and see just symptomatically, and hydration, over the counter medications and wait before we start antibiotics.

M: Ok. Perfect. And what over the counter medications do you typically recommend?

015: The AZO.

M: Which one is that?

015: The AZO. It's called AZO. (spells out) A- Z- O. It's very common.

References 2-3 - 0.52% Coverage

They will try AZO. No doctor, I tried, didn't work. Ok. Then in that case I would give just a simple dose of Bactrim DS.

M: Ok. Of Bactrim DS, you said?

015: Yes.

References 4-5 - 3.03% Coverage

Ok. Alright, and so say you've gotten the urine studies back and you are....you're convinced that it's a urinary tract infection and you're planning on treating her, what antibiotics do you typically go to?

015: Bactrim is the first one.

M: Ok.

015: Keflex if they are allergic to Sulfa.

M: Ok.

015: Keflex. And then if they are allergic to Sulfa and the Penicillin then I try Cipro.

M: Cipro. Ok. Do you feel like some antibiotics work better than others?

015: Yes.

M: Ok.

015: I think that in my experience, Bactrim is better than Keflex. Bactrim and Keflex are better than Cipro.

M: Ok.

015: And for some bacteria, especially the E. Coli.

M: Sure.

015: The E. Coli we're seeing a little more resistance now. We were promised they would not be resistant. Before you were born they said there would never... you would never see resistance. Now it is happening.

M: For sure. Seeing it more and more and more.

015: Yeah, so I don't like the quinolones.

Reference 6 - 0.48% Coverage

If they want to do something like….. you can use, let’s say Bactrim or sulfa, and my patient is has allergies to sulfa I would say... I won’t use it, so….

<Files\\08_15_18_002> - § 8 references coded [15.76% Coverage]

Reference 1 - 1.57% Coverage

that urine dip, the UA comes back showing leukocytes, nitrites, even trace of blood but I would probably still then that is probably give Bactrim it's inexpensive it's well tolerated it usually works for most people so it makes good sense in all the cultures most of the cultures we had have come back sensitive to bactrim so it's nice and easy.

Reference 2 - 4.86% Coverage

what are the other choices that you usually think about when u cannot use Bactrim?

002-- I am very fond of Macrobid, also very inexpensive , very well tolerated, test if there's a chance they could be pregnant nice to know that it's safe as beyond that I used to do Cipro but the one of the primaries that I worked with she would do super 250 for 3 days never worked. I would get the calls saying I saw this doctor so an so and I've been taking the antibiotic and it didn't get better It didn't change and I look in the chart its Cipro 250th 3 days it didn't work, so I would do Cipro 500 but usually for 5 to 7 days but then this whole thing with the fluoroquinolones in the Black Box warning and then and now try to stay away from any of the fluoroquinolones if I can. So ironically especially if they've already done bactrim or Macrobid go to one of the cephalosporins or even amoxicillin not the best choice but still would hit a lot of the possible bacteria and again very well tolerated very inexpensive usually works pretty well not my first choice but it's an option

Reference 3 - 1.00% Coverage

I would say okay with Bactrim DS 1 PO BID 7 Days sent to the pharmacy and then the MA would call them and tell them okay doctor sent antibiotics to the pharmacy, if you dont get better you need to make an appointment.

Reference 4 - 0.45% Coverage

I usually go to Macrobid and it still reasonably priced and do not give too much trouble about that

Reference 5 - 1.85% Coverage

guys don't usually get UTI so when are coming in we're also and what we they may have bactrim as well but we usually also do what's called a dirty urine which is looking for gonorrhea and chlamydia and anything else because guys don't usually get you don't usually see it in the young guys, older guy certainly could be prostate related but we don't have the ability to do labs here so I didn't check the PSA.

Reference 6 - 1.30% Coverage

I used to use a lot of Cipro. Cipro is good for older guys because of prostatitis or anything more than just a typical UTI if it was anything else Cipro the nice option and I still may even go to that on occasion but yeah the Black Box warning for the FQ really did back my use of cipro

Reference 7 - 3.51% Coverage

as far as resources honestly Dr Morris? is a good resource. He stays on top of everything and he does research , he has articles and they have all kinds of stuff and especially when I moved over here there were a lot of I was set in my ways for antibiotics as a PCP I usually have my same ones for this and this is for UTIs. came over here and people would be allergic to Cipro and Macrobid and Bactrim and I'm like what in the world do you use and he's like well… and so he's a huge resource and Cassie and Gina the nurse practitioners they've been doing acute care even longer than I have so you know I consider my acute care started back in January when I moved over here so their resources as well so I'll forget certain antibiotics so and I realize I could use those.

Reference 8 - 1.23% Coverage

just so many unknowns and so many questions that you can't say you strep is going to be treated by amoxicillin because they're even him for allergic to Amoxicillin you're still not going to do azithromax everytime and people don't like that azithromax for lots of reasons.

<Files\\08_15_18_003> - § 6 references coded [19.17% Coverage]

Reference 1 - 4.16% Coverage

you are really convinced or you're really suspicious about the UTI diagnosis does that change your practices for whether you are going to test them or get a culture or do you always regardless of your kind of pretest probability you always get a urine test?

003-- it in my mind in my mind I'm taking okay this is pretty straightforward but we still do it, yeah in this setting, because if for some reason there is antibiotic resistance then we can go back to the …okay this is what we ordered we got to change the antibiotics and then perhaps not going to work and maybe that's why you r not getting better. And since it's a seven-days-a-week operation I may not be here and I dont know the whole story. Someone might be able look at my chart and kinda see what is going on with it, it may be a better safety net if you want to use that term, making sure that the proprietors here when I'm not here they can just easily look at the the culture and say oh he prescribed Cipro and then can change to bactrim.

Reference 2 - 4.61% Coverage

when you're seeing the patient and you won't have the culture results cuz you'll be treating them kind of empirically so what what do you prefer usually when you're when you're seeing them here?

003-- of course for non-pregnant non-nursing moms I want to make sure I use the antibiotics that are safe so let's assume it's that case so I might start Keflex or macrobid or sulfa. Those are my top three unless they say I can’t tolerate that, if they had a rash before, nervous about taking a medication so if there's no contraindications to use then those three and perhaps over the years and I think I probably use Cipro a lot more early on but changed over the years with literature coming out saying maybe we need to use this or seeing more cultures that said was seeing a lot of resistance for this than other. And sometimes it's just kind of like people talking or clinicians talking like seeing or trending a lot more resistance to this and we ought to be using cephalexin for the first line or maybe need to get away from the quinolones or watch out for the sulfa reaction. do you want to know about maybe other…….

Reference 3 - 1.70% Coverage

reduce your FQ use…What was what influenced you to change this practice?

003-- Yeah I think the medical letter, reading in journals, hearsay like some urologists are saying don’t use this or that for resistance, haven’t done a lot of Cochrane reviews or other research but it may be the medical letters pretty non biased so I like to use that as a resource and maybe just some of the journals kind of sway me …

Reference 4 - 4.21% Coverage

the FDA black box warning? Did that change our decisions at all or not some people that can influence them some people in a left so if some of the side effects they mentioned in the black box yet and they're pretty low too?

003---they are so yeah they influenced patients calling back and saying ….maybe retrospect maybe it did influence me I didn’t want to get that call back and asking me to switch because they didn’t want their tendons to rupture so maybe looking back maybe that did it have some influence on ya on me but more because of the patients are going to read it and call/come back. So as far as that on the quinolones the only thing I was noticing too maybe I'll ask you later when we're done is that the lower dose of the quinolones were failing, like the 250s for minor UTI that is 250 BID for five days and many came back with recurrence and the same with Levaquin 250. One of the Levaquin reps told us we don’t want to talk about levoquin 250 anymore, so maybe those are some influences over the years.

Reference 5 - 3.36% Coverage

-Of the top three that you mentioned at what goes in your decision-making process about prescribing a cephalosporin or Macrobid or bactrim ?

003--Right….It if I'm if someone's going to really struggle with compliance I want to use twice a day or once a day if I can. I usually take a moment to ask about can you swallow pills, do you think you could do four times a day and get an idea most people are confident oh ya I can remember but reality is not….

If I get this impression that someone is really not compliant I go with twice a day, So I might go with the macrobid or Bactrim in those cases or I might do a different cephalosporin like cefdinir or Augmentin twice a day so if I'm more confident they can handle the four times a day regimen and then go with Keflex or ampicillin sometimes that pretty rare.

Reference 6 - 1.13% Coverage

Can manage with follow up if not getting better, if severe complications then go to the ER and may want to prescribe something maybe a little bit safer if the patient could be potentially pregnant so I might go to those that may be keflex or macrobid for three to five days.

<Files\\08_15_18_004> - § 4 references coded [16.46% Coverage]

Reference 1 - 1.54% Coverage

Then you mentioned you like to treat for about 5 to 7 days… what antibiotic do you usually like to give?

004—for years it was Septra ds and then it was Cipro and now there's so much resistance to antibiotics we often go to Nitrofuratoin

Reference 2 - 3.41% Coverage

--So it is that usually when you say your go to antibiotic or do you are there certain characteristics or things that change your mind about which antibiotic you should prescribed for the patient.

004—Cipro or Macrobid and thats based on allergies you know And that's pretty much what we use

M--And then how do you decide between the two?

004—I would confess it is largely random unless they've recently been treated with Cipro I would probably give that first, in young patient in a community the resistance is so high.

Reference 3 - 6.57% Coverage

That's wonderful I'm happy to hear that. So you mentioned historically that you kind of shifted from Septra to Cipro and now more Macrobid antibiotic prescribing… what what's influenced your decisions in terms of your evolution about selecting specific antibiotics?

004—When I started out there was no Cipro there was only Septra. Cipro, levoquin came along and they were better tolerated and carried a little less risk in terms of kidney and no hyperkalemia and the other stuff, allergic reactions. And then more recently using a lot of macrobid. So you asked about a young patient I would definitely use the nitrofurantoin and in older population certainly in a nursing home population because of the high incidence of Cipro or quinolone resistance that we are seeing.I don't have a statistic but we see it a lot.So we've got using a lot more nitrofurantoin.

M--What about the Septra ? You see a lot of resistance to Septra too?.

004--There's resistance to septra, we just moved away from using it much.

Reference 4 - 4.94% Coverage

I guess specifically the FDA Black Box warning is that something that influenced your prescribing it or not?

004—A little probably not a lot. We think a lot in the lines of and sort of balance guidelines from you know like the infectious disease society and other things, what their guidelines say and what the practical environment is. You know actually we looked away from inviting people into the office in the days of the close of the capitated HMO patients, so just honestly its an economic thing, it doesn't seem like a bad thing to go ahead and treat and it was cost effective to give someone a $5 antibiotic and not ask them to go through a whole lot of testing or evaluation. Its less expensive and then we just continue to do it or I have anyway.

<Files\\08_23_18_005> - § 5 references coded [14.26% Coverage]

Reference 1 - 0.93% Coverage

And go from there, but first thought that goes through your mind is, she’s not pregnant, she’s 27, could it be an STI? Definitely ask history of kidney stones, history of any new sexual partners, and then macrobid is my first thought after that.

Reference 2 - 3.45% Coverage

Yeah. And then you mentioned that you like to use macrobid as your first line agent. Can you tell me what your thoughts are about different antibiotics, and why you like macrobid compared to others?

005: I just feel like macrobid has a lower side effect profile, less people are allergic to macrobid for some reason, cause there's no other drugs in that class, I feel like unless they’re menopausal, after 50, I feel like most are not resistant to it and it’s just kinda the go -to as far as

evidence-based practice from when I was in school that was kinda just what we were taught, especially with cipro, and their recent side effects and the black box warning and stuff, we’ve kind of stayed away from cipro, but I feel like a lot of our practitioners still use it. I just go with macrobid, morning and night for five to seven days, depends on the severity of symptoms, if they’ve had one recently.

Reference 3 - 5.54% Coverage

And then what other antibiotics come to mind for you other than cipro and macrobid, are those the two main ones?

005: Bactrim, double strength, we’ve used a lot. I mean we can use like a cephalosporin if they’ve shown resistance to something in the past. Generally that would be like a last case, or like a keflex, just depends on allergies, like if somebody has an allergy to bactrim, macrobid hasn’t worked for them in the past, we’re going cipro, we’re going keflex, it just kinda depends. Or if they’ve had any past culture that have shown resistance, gotta steer clear of the ones they’ve been resistant to and I just kinda have to go down the list and see on the culture what its been susceptible to in the past.

M: Got it, so they have like an intolerance to macrobid, but they’re okay with everything else, do you have a second line agent that you like to use?

005: Generally I go with Bactrim double strength. I don’t, like, no idea why I’ve just gotten good coverage over the years and nobody’s called back and said like hey this hasn’t worked. And then once again a lot of patients see the cipro warnings when the pharmacist goes over the side effects with them we get a lot of calls back for cipro from pharmacists like “hey do you realize you put them on cipro” and then the patient calls back with a lot of questions. Bactrim double strength generally doesn’t give me that kickback. We don’t get a lot of phone calls back for it.

Reference 4 - 3.33% Coverage

Alright. And when did you start getting the phone calls, was it recently, or you’ve been getting them for several years?

005: When I worked, prior to here I worked for CVS as a nurse practitioner, we were not allowed to prescribe, we had guidelines that we had to follow, and cipro was like worst case scenario

unless the patient was allergic to everything else. Cipro was our last antibiotic that we went to, and if we used Cipro we had to type a narrative in our note why we chose it like if the patient requested it. It was a very low use antibiotic, because they went off of everything evidence based, and ever since then its kinda been my last antibiotic used, and I feel like we’ve gotten several callbacks here starting in December ‘17, and I feel like we’ve gotten a couple based on, I don’t use it that often, but when I do I feel like we get pharmacy calls a lot.

Reference 5 - 1.02% Coverage

It changes based on the antibiotic. I mean I think macrobid you can get away with 5 on the lesser strength, bactrim 5 to 7, and then cipro I mean if I’m gonna do it on cipro then generally 3 to 5, I don’t think cipro needs as long of a course unless there’s a history.

<Files\\08_23_18_005 (2)> - § 5 references coded [14.27% Coverage]

Reference 1 - 0.93% Coverage

And go from there, but first thought that goes through your mind is, she’s not pregnant, she’s 27, could it be an STI? Definitely ask history of kidney stones, history of any new sexual partners, and then macrobid is my first thought after that.

Reference 2 - 3.45% Coverage

Yeah. And then you mentioned that you like to use macrobid as your first line agent. Can you tell me what your thoughts are about different antibiotics, and why you like macrobid compared to others?

005: I just feel like macrobid has a lower side effect profile, less people are allergic to macrobid for some reason, cause there's no other drugs in that class, I feel like unless they’re menopausal, after 50, I feel like most are not resistant to it and it’s just kinda the go -to as far as

evidence-based practice from when I was in school that was kinda just what we were taught, especially with cipro, and their recent side effects and the black box warning and stuff, we’ve kind of stayed away from cipro, but I feel like a lot of our practitioners still use it. I just go with macrobid, morning and night for five to seven days, depends on the severity of symptoms, if they’ve had one recently.

Reference 3 - 5.55% Coverage

And then what other antibiotics come to mind for you other than cipro and macrobid, are those the two main ones?

005: Bactrim, double strength, we’ve used a lot. I mean we can use like a cephalosporin if they’ve shown resistance to something in the past. Generally that would be like a last case, or like a keflex, just depends on allergies, like if somebody has an allergy to bactrim, macrobid hasn’t worked for them in the past, we’re going cipro, we’re going keflex, it just kinda depends. Or if they’ve had any past culture that have shown resistance, gotta steer clear of the ones they’ve been resistant to and I just kinda have to go down the list and see on the culture what its been susceptible to in the past.

M: Got it, so they have like an intolerance to macrobid, but they’re okay with everything else, do you have a second line agent that you like to use?

005: Generally I go with Bactrim double strength. I don’t, like, no idea why I’ve just gotten good coverage over the years and nobody’s called back and said like hey this hasn’t worked. And then once again a lot of patients see the cipro warnings when the pharmacist goes over the side effects with them we get a lot of calls back for cipro from pharmacists like “hey do you realize you put them on cipro” and then the patient calls back with a lot of questions. Bactrim double strength generally doesn’t give me that kickback. We don’t get a lot of phone calls back for it.

Reference 4 - 3.33% Coverage

Alright. And when did you start getting the phone calls, was it recently, or you’ve been getting them for several years?

005: When I worked, prior to here I worked for CVS as a nurse practitioner, we were not allowed to prescribe, we had guidelines that we had to follow, and cipro was like worst case scenario

unless the patient was allergic to everything else. Cipro was our last antibiotic that we went to, and if we used Cipro we had to type a narrative in our note why we chose it like if the patient requested it. It was a very low use antibiotic, because they went off of everything evidence based, and ever since then its kinda been my last antibiotic used, and I feel like we’ve gotten several callbacks here starting in December ‘17, and I feel like we’ve gotten a couple based on, I don’t use it that often, but when I do I feel like we get pharmacy calls a lot.

Reference 5 - 1.02% Coverage

It changes based on the antibiotic. I mean I think macrobid you can get away with 5 on the lesser strength, bactrim 5 to 7, and then cipro I mean if I’m gonna do it on cipro then generally 3 to 5, I don’t think cipro needs as long of a course unless there’s a history.

<Files\\08_23_18_006> - § 2 references coded [11.07% Coverage]

Reference 1 - 6.92% Coverage

1. I would talk to her more about her symptoms, find out if there’s anything else going on, are you sexually active, is there a chance that this could be an STI, is there any chance you could be pregnant, even though you don’t think you are at the moment? Are you having frequency, urgency, hesitancy, is there blood in your urine, you ever have a kidney stone before? Fevers, back pain, abdominal pain, anything else going on? Then I would, the girls are really good about getting the urine-dip, talk to the patient about, well it looks like maybe there might be something, you’ve got some, if she’s got leukocytes or nitrites maybe she’s got something else going on. We can try an antibiotic, I would probably start with macrobid, twice a day for 7 days, unless they wanna balk at it. I would send it for culture no matter what. If I didn’t see anything that was indicative, I would try to talk them out of insisting on an antibiotic. I’d tell them I just really don’t see anything. What’s in the culture, if it comes back we can always start you on something, but it doesn’t really look like that’s where the problem is, so why don’t you follow up with primary or urologist.

Reference 2 - 4.14% Coverage

1. Got it. So you mention macrobid, that's gonna be your first line antibiotic. Can you tell me your thoughts on different antibiotics and why you prefer macrobid for patients?
2. I like macrobid because I know it’s safe in pregnancy, and I know that it’s safe for most age ranges. I know that it’s really good for urine stuff, and it’s not gonna be one of those that I run into too often with people with allergies. I have had a couple claim it, I don’t like reinventing the wheel so if I know it works well, it’s what I’m gonna go with.
3. Got it. Do you think there’s any downsides with macrobid compared to other antibiotics with UTIs?
4. I haven’t run into it, but that doesn’t mean they don’t exist.

<Files\\09_05_18_007> - § 5 references coded [13.95% Coverage]

Reference 1 - 2.48% Coverage

1. in terms of management, if the patient called over the phone, would you have them come in over the phone for a urine dip, or what do you do if they call over the phone?
2. I think I would like to treat patients like this over the phone so that they don’t have to go to the ER, Urgent Care. It can be safely treated by starting some medications that I have favored to treat simple UTIs. I usually use either bactrim DS, if there’s no allergy of course, 1 tablet twice a day. I usually do 5 day course, there are times I tell patients to use it 3 days and their symptoms have subsided within 48 hours they can do a short course of 3 days, but 5 days is what I send the patient a prescription. My second choice is macrobid, twice a day for 5 days.

Reference 2 - 1.79% Coverage

1. Then you mentioned cipro as one of the preferred antibiotics. Do they have antibiotics that they recommend, and if so what do they usually say?
2. The preferred antibiotic in my experience in talking to the nurses is most of the time cipro, and patients have taken it in the past for these things, and they say that. I don’t hear a lot about macrobid, unfortunately, I don’t know why. We have a lot of patients who are elderly in our practice, so the bactrim is not a favored choice. We hear a lot about cipro and the next is macrobid.

Reference 3 - 4.26% Coverage

1. Do you have any specific opinions on which ones you prefer when, do you think certain antibiotics are stronger than others, or tend to work better than others in terms of your historical practices? I know that there’s some concern about increasing resistance and things like that, so some providers tell me that resistance rates play a role. What are your thoughts about, how do you decide?
2. RIght, so, you know for the most part we know patients who are low risk without multi-drug resistant infection, like simple cystitis, e.coli is the most common, I personally think that my choice is bactrim, nitrofurantoin, it should take care of them. Either I use nitrofurantoin or bactrim, in terms of which one is my favorite, I guess I think it’s all about allergies, if there’s an allergy I don’t prescribe bactrim obviously. I have not looked into, honestly, if bactrim resistance or e coli is more than nitrofurantoin. I have a habit of looking back, even in young patients, if they ever had a cultures in the system, if I have time to pull that up and see if there is any bacteria that they had a culture for an unknown reason and what was the sensitivity of that. Sometimes I do that, but either both of them is my first choice, not sure which one is more sensitive to e coli.

Reference 4 - 4.20% Coverage

1. Tell me more about that for urgent cares. So you’ll see them often prescribe for a longer treatment duration, are there certain antibiotics they tend to prescribe, too?
2. Yeah, so I think they do like bactrim, I see a lot patients come on bactrim, 7 ays is the most commonly what they give. Cipro is the second one, 5 day, twice a day, is very common that I see patients coming with the prescription.
3. Do you find that it’s difficult, or how do patients respond when they get a different treatment from urgent care or a different provider than what you’re recommending? It sounds like they often do longer durations, in your opinion, so is there any issue?
4. I think it’s, when you have a relationship to the patient, they trust you. It’s the education to the patient that, I understand that you have this infection, you went to urgent care. If they have done the cultures, I’d like to get that. It takes about 48-72 hours, but in the meanwhile, I would like you to, with your symptoms you should be okay to do 5 day course to the 7 day course. They really appreciate that, because they don’t want it to be on this antibiotic, so for the most common UTI symptoms, I will tell them what else to look for if they start getting worse or something like that.

Reference 5 - 1.23% Coverage

1. And what are your thoughts about a patient with multidrug resistant UTI who is simply coming with a simple symptom not generalized symptoms or red flags. There are some infections where it’s okay to treat them, with 10 days of whatever sensitivity they have, or there are some infections doctors that no, it treated be treated with antibiotics, preferably meropenem.

<Files\\10_10_18_008 (2)> - § 2 references coded [9.50% Coverage]

Reference 1 - 5.87% Coverage

you mentioned that you use empiric antibiotics. Which ones do you typically like to use and why?

8: I would typically use Cipro or Bactrim. I believe that that Bactrim probably has a better E coli coverage currently than Cipro and Cipro has some nasty side effects. I've actually given someone an achilles tendon rupture, but those would be my two initial thoughts.

M: Got it. And when you're trying to decide between those two, do you think one is stronger than the other or do you think, you mentioned some of the side effects with Cipro that you get worried about. So do you avoid one or some of the antibiotics over the other one, based on, certain patient factors, or what goes into your decision making about the antibiotic? And it's okay if you tell me, you know, and it's completely arbitrary too. That's fine as well.

Reference 2 - 3.62% Coverage

, I probably used Cipro more than I should have and I probably should have tried to have used bactrim more. But I think it just tended to become what I was comfortable with. It was just more of an automatic thing more than necessarily looking down at every single factor.

M: Okay. That sounds great. Do you think patients are pretty good at telling you if they have a UTI when you get a message or if they present to you, are they pretty reliable do you think I reporting their symptoms?

8: I think so yes.

<Files\\11_14_18_009> - § 3 references coded [6.60% Coverage]

Reference 1 - 2.34% Coverage

do you have certain antibiotics that you prefer to treat UTIs with over others?

009: Yeah, I do. I think that’s not for any specific reason, I think I was just trained, used to prescribing those antibiotics. But definitely look at the culture, the sensitivity, all that stuff. But traditionally we’d do macrobid or cipro are the two tat we would prescribe most of the time.

Reference 2 - 2.55% Coverage

Then one of the other common antibiotics that people think about for UTIs is Bactrim. What are your thoughts about bactrim?

009: Definitely, but I think cipro and Macrobid I’ve been using more often.

M: Got it, and it sounds like that decision is mostly personal preference and then what the patient prefers too, is that ?

009: Absolutely, there’s no specific indication that I’d use one over the other.

Reference 3 - 1.70% Coverage

How long do you usually treat patients with UTIs for?

009: MOst of the time, 7 to 10 days.

M: Is that for both of them, cipro and macrobid about the same?

009: I think macrobid, I think cipro we do for 10 days, I think macrobid is 7 to 10. I think mostly we do 10.

<Files\\12_18_18_010> - § 2 references coded [12.50% Coverage]

Reference 1 - 6.91% Coverage

I would do a physical exam. I’d ask her if there’s blood in her urine, does she think that she’s, sometimes people can link symptoms to something like sitting in a hot tub or sexual intercourse, see if anything has changed from normal activity. I'd get a urine sample from her, and do a urine dip, and if it’s positive or looks like traces of blood, then definitely send for culture. In a young, healthy female, if I’m convinced she has a UTI, we’ll definitely send for culture as well but put her on 3 days of therapy, oral therapy twice a day, maybe septra, depending on her allergies of course. And I usually don’t bring people back for recheck unless symptoms come back, advise them to drink water/fluid avoid caffeine, alcohol, activities that would irritate the urinary tract. If you have any fever, chills, etc.

Reference 2 - 5.59% Coverage

You mentioned giving 3 days of Septra for this patient once you get the test results, are there any other antibiotics that you would choose?

010: I would start either Macrobid or Septra depending on their allergies. I would not reach for Cipro right away, especially if its somebody who doesn’t have a history of recurrence. She’s got a short history of it, it doesn’t sound like. If her dip is negative, I wouldn’t start her on an antibiotic, I’d say to her we’re just gonna wait for the culture to come back. BUt, i wouldn’t use Cipro or anything like that to start out, i’d use septra or macrobid, maybe keflex, especially if she’s naive to antibiotics.

<Files\\12_19_18_011> - § 3 references coded [12.42% Coverage]

Reference 1 - 4.34% Coverage

which would you choose and what duration?

011: Most of the time, if it’s, the only symptom is dysuria and UA is positive and no allergies, ether keflex or cipro. Usually ask what works best for them, because the majority of patients I see, it’s not their first UTI. And so those are the main 2 that i go with. If there’s allergies then sometimes bactrim. Majority of the time treatment is 3-5 days depending on their past history and severity of symptoms.

Reference 2 - 1.28% Coverage

: Are there certain antibiotics which are better or worse that others according to you?

011: No, it really depends on the patient.

Reference 3 - 6.79% Coverage

And do you think that providers should be evaluated how they appear to these guidelines?

011: I think we are with the whole best in class type stuff.

J: I mean specifically for UTIs.

011: Depends how the guidelines are worded. Obviously if they’re saying you need to use cipro vs bactrim vs keflex or however, if it’s like these are your choices and here are the best ones, or this works best, whatever, I think then something like that is fine. If it’s super specific whenever things change with patients and you have to change something, then our rates will go down whenever it’s actually what’s best for the patient. I feel like that’s a really generalized question for something that can be pretty specific

<Files\\12_20_18_012> - § 3 references coded [14.13% Coverage]

Reference 1 - 2.19% Coverage

: So you said you’d choose to treat with antibiotics, can you tell me which ones and for what duration?

012: I would maybe use probably Bactrim DS 1 twice a day for 7 days

Reference 2 - 4.06% Coverage

Are there certain antibiotics which you consider better or worse than others?

012: It depends, it they’re having recurrent UTIs then I’d get a culture and it would tell you which antibiotic is better for that strain of bacteria, but I’d usually go with bactrim or cipro or possibly macrobid depending on drug allergies.

Reference 3 - 7.88% Coverage

Do you guys ever use antibiograms to look at antibiotics and antibiotic resistance in your area?

012: No, not usually. We have one, but it’s not something I look at very often.

J: How about using that, do you have any thoughts about how that would be helpful for you guys?

012: It’s definitely helpful if you’re not sure which to give. With respect to UTIs, I don’t use it that much You kinda have to start them on something, so I start them on traditional first line treatment, and then I’ll get a culture to see if I need to change the antibiotic. I don’t know that that would really be helpful in this situation.

<Files\\8_13_18_001> - § 4 references coded [13.11% Coverage]

Reference 1 - 2.63% Coverage

. So can you kind of approach how you would deal with that in a clinic setting if you saw that patient?

001-- so obviously we check a urine dip in the office make sure she is not pregnant. You know if she doesn't have any allergies, check for other medications and usually I'll give a 3-day course of Cipro if I'm not concerned that somebody is pregnant

Reference 2 - 3.80% Coverage

you generally do Cipro, do you have a preference or why do you do Cipro over antibiotics?

001—I will use Macrobid if it's somebody that has drug interactions or I'm worried they're pregnant. I really don't use sulfa a whole lot just because there's so many people with allergies and I have had a lot of cases in the past year of older women they go to the Urgent Care and get sulfa and I'm not kidding I've had 5 people end up in the hospital with AKI or kidney failure, so for older women I just don't like it.

Reference 3 - 2.62% Coverage

Do u have a sense about some abx being more potent than others or when you compare Macrobid and Cipro do you have any.

001- Well I think there is some resistance to Macrobid or obviously it takes a longer course so I would use that second choice but I don't obviously and possibly pregnant women or older people can't take Cipro for the medications.

Reference 4 - 4.06% Coverage

when people are covering or exchanges sending in antibiotics or something, the drug interactions popped up automatically and now that doesn't happen you have to click on new interactions to see, so will frequently see people get sulfa, Cipro and there's probably at least of possibly serious drug interaction and nobody looks at that because it doesn't pop up automatically anymore so I think that's another thing people really need to be cognizant of is always clicking that button because there's a lot of people who can't take these things.

Patient Factors

<Files\\01_08_19_013> - § 4 references coded [13.63% Coverage]

Reference 1 - 3.15% Coverage

What are the other antibiotics you usually think about beyond sulfa and ampicillin?

013: Nitrofurantoin for younger people with good or adequate renal function. Ciprofloxacin for third line.

M: What makes you choose one agent over another one?

013: I’ll try and do simple medicines first. Sulfa is sort of an old stand by. It can be so much resistance to ciprofloxacin, it’s a more potent medicine theoretically.

Reference 2 - 3.32% Coverage

So, mostly it sounds like potency and then guidelines are usually what you use to select antibiotics. What do you use for treatment duration?

013: Younger people 3-5 days. Some people i know get by on 3 day courses. 5 is probably the most common. Older people 5-7

M: So for younger ones 3-5, usually 5 and sometimes 3 if their symptoms aren’t too bad?

013: Right and sometimes just a track history of 3 day antibiotics working well.

Reference 3 - 4.58% Coverage

You mentioned fluoroquinolones are usually your last line. What makes you reluctant to use them?

013: Increasing resistance pattern is probably number 1, cost is somewhat a factor, they’re not that expensive. Really up there in the hierarchy of antibiotics use they shouldn’t be used for this, try to reserve them for more resistant infections.

M: Do you see a lot of resistance to trimethaprim sulfa then?

013: In older patients really, in younger patients it’s still a pretty good medicine. Or I’ll say non hospital non medically ill. I’ve got some 40 year olds that are 100 years old physiologically.

Reference 4 - 2.58% Coverage

has also started implementing, e visits now too for UTI. I think they’ve implemented both, a nurse triage algorithm and an e-visit too, where providers then see the patient via communication. I don’t think they skype or anything.

013: Right, we’ve gotta meet the needs of the millennials who don’t want to see a doctor. Everything’s online.

<Files\\01_10_19_014> - § 6 references coded [26.94% Coverage]

Reference 1 - 6.64% Coverage

How will you handle the case?

014: With somebody like that, we do the Udip in the office. If there’s anything that seems generally positive, leukocytes or nitrates or anything like that, we’ll go ahead and treat them, if it’s uncomplicated and they don’t do it all the time, I usually do 3-5 days of macrobid. It kinda depends, I stick more towards 5. It might be overkill, but since they usually aren’t in my setting, we’re primary care but we also do walk in, and we’re in a tourist area so we get people we’re never gonna see again. We kinda wanna send them off adequately set up, and then we’ll send that urine for culture. FOr everybody that has insurance we send the culture, sometimes if they don’t we give them the choice. Otherwise we just go with it and tell them to follow up if they don’t have resolution of symptoms.

Reference 2 - 2.05% Coverage

You mentioned macrobid, are there other antibiotics that you consider?

014: I do, usually it’s between macrobid and bactrim for those that age group. You said 27? Yeah, those 2 for that age group, especially if they don’t have a lot of recurrent UTIs.

References 3-4 - 7.44% Coverage

So you mentioned a couple antibiotics. Do you consider certain ones better or worse?

014: I guess, we used to give cipro all the time, and then with all the different considerations about the fluoroquinolones now, we’ve really backed off of those. I don’t really have one that I call on more than others, it really kinda depends on the age and whether I think it’s a recurrent complicated thing or no. So, no.

J: So you just mentioned fluoroquinolones and that you’ve backed off using them. Can you elaborate?

014: Sure, really because with the tendonopathy people can get, and it seems like there were more cardiac concerns. Every time I turned around, it seemed like somebody was saying something bad about the fluoroquinolones. I think it’s just that they seem to be, more of a last resort. Or I do still use them if I feel like somebody, if they have kidney stone history or something that doesn’t just seem like a UTI.

References 5-6 - 10.81% Coverage

What factors do you consider when deciding to treat? Patient factors, symptoms, age, sex, etc

014: I think I’ll always, even if I don’t have the culture and the Udip is kinda eh, means to me the leuks are trace. If they’re my age and up, maybe even 55, because those sensors don’t work as well and you don’t get as much dysuria, but you can really get sick. If they act sick I’ll treat them. Even if they kinda don’t act sick, but that looks like a UTI even if I don’t have all the information. For a younger group, if it doesn’t look like a UTI and they’re sexually active and have things that could be more irritants, cause younger women have just more stuff going on. They’re exercising, they're wearing tight clothes. I’ll tell them I wanna wait until the culture is back, unless they have a fever. Sometimes, I see under 21s, so with that crowd if they have a fever I’ll start them on something.

J: How about males and females?

014: I get so so few males with UTIs in this current practice that I kind of forget about them. When I was dealing more with older adults, I’d get what sounded like a UTI but probably was prostatitis. I’d give them cipro, but I’d probably have to look it up now. It seems like in my memory the males that complained of something usually acted like they needed medicine and we just went ahead and took care of it.

<Files\\02_07_19_015> - § 8 references coded [10.52% Coverage]

Reference 1 - 1.05% Coverage

There will be difference between she goes three times as opposed ten times when she wakes up at night and what has she done for it? Has she tried the AZO over the counter? Has she been drinking water? Has she been exercising? Is she a runner? Is there something that might have caused an Irritation that could have started this? What else?

References 2-4 - 2.95% Coverage

When you're deciding. When you're trying to decide whether or not to pull the trigger on antibiotics. To treat vs. not treat. You know, we talked about looking at the urine studies. Are there any sort of patient factors that will make you think more about treating or not treating? Do you feel like age matters or gender matters?

015: Yeah. Age. Gender. Comorbid conditions, especially diabetes, previous instrumentations, admissions to the hospital, previous urinary tract infections. I try to see if they have previous infections, something in the records. History of stones, hematuria, fever. Pregnancy. I don't deal with them, but I know that pregnant ______ 10:25 to be treated.

M: Yep.

015: And so I don't deal with them so don't ask me about pregnancy....

M: (Laughing)

015: All I say is just go to the...either to the gynecologist or to the family practice. I think that you need to be treated. (Laughing) because I don't deal with them.

Reference 5 - 1.84% Coverage

Do you think there is anything that the ID societies or the U.S. Preventative Task Force could be doing to help providers like yourself educate their patients? Do you think there is a way that they could be doing more?

015: Yes. Definitely.

M: What sorts of things would be helpful for you?

015: Divide the patients in classes like the… explain the first place that there is a very low chances of having a significant UTI and comorbidity, age, and give us an idea. It may be too specific, but I think it is worth it because UTI is such very common condition. Especially in nursing homes.

Reference 6 - 1.99% Coverage

At that time, they said that you don’t treat just because the patient thinks they have ___16:46 and antiuria in the nursing home. You can actually just give them water, stuff like that and then all of a sudden it’s like well, in these cases you have to treat it but you have to do it this way.

M: Gotcha.

015: At the beginning it was they just don’t treat it just because… unless they have symptoms.

M: Okay.

015: The asymptomatic bacteria, don’t treat it.

M: Right.

015: Except for pregnancy. Don’t give the ______17:09.

M: Sure.

015: Asymptomatic bacteria, only in pregnancy.

M: Gotcha.

015: But eventually this changed.

Reference 7 - 1.50% Coverage

What are your thoughts about nurses or non-physician staff following algorithms for UTI’s.

015: It helps a lot, especially if it’s an outpatient. A patient comes to the clinic and they already follow protocol that makes sense, that saves me time…

M: Okay.

015: I suppose me coming with the patient, figuring out again what the problem is and then ordering that… so it has helped. Especially in the nursing homes. It has reduced significantly the treatment of unnecessary UTI’s.

Reference 8 - 1.20% Coverage

: Recently I had a patient that came to me that I thought he was recurrent of having dementia. I wasn’t sure what was going on. Diabetic. I have not seen for a while because he is very healthy diabetic and he actually got a UTI. The first time as an outpatient that somebody that I think is having dementia. No fever and it was a UTI. He’s a diabetic with UTI. That was unmentioned to me.

<Files\\08_15_18_002> - § 7 references coded [20.69% Coverage]

Reference 1 - 7.73% Coverage

If she was a established patient and seen her in the last several months and was someone I was familiar with wouldnt necessarily make her come in. based on her symptoms and if I knew her and was reasonable and not over dramatic and no other medical problems and nothing to check, I would probably send out antibiotics for her without making her come in especially if I didn't have any openings in my clinic. So instead of making her wait and frequently we did not have openings then we would say send out antibiotics and say if your symptoms don't improve you'll have to come in and make an appointment to see me, so that was Primary Care. Here in Convenient Care they come in with the symptoms everyone gets a UA and based on those symptoms even if the UA came back with negative nitrites and even trace leukocytes still probably would treat but we always here we always send cultures unless they are self-pay, so here they get a culture and if self-pay we will go back and forth and talk to them and this is how much it's going to cost but it'll give us these benefits to make sure it is an infection by using the right medicine to treat for and most of the time they do not want the self-paid do not want the culture. People when it's not self-pay their like okay sure we get the cultures back and I'd say probably the majority of the time there is an infection but there's probably 30 to 35% maybe even 40% when there is not an infection and so will call him back and we'll say stop the antibiotics if you're still having symptoms you need to follow up with your primary care or whoever your OB-GYN sometimes if they don't have a PCP but in that case someone who has no medical problems 2 days of symptoms.

Reference 2 - 4.07% Coverage

M--It sounds like you in your life is a primary care doctor you felt comfort in managing a case over the phone sometimes …

002--especially if I was familiar with them been my patient for a long time , especially in Primary Care you get to know some of their background on their histories and you can pull up and see their picture and go oh yes I remember there was a person she doesn't call in unless there's a problem and usually they have been tested in the past you know if it's a first-time UTI I still might make him come in but usually these are people who had it before and they would call and I'd say this feels exactly like the last time I had 6 months ago and I was and I went on my honeymoon and there are reasons and you know their history and background and not crying wolf and then there was always that caveat of if you're not getting better you have to come in as an appointment.

References 3-6 - 7.59% Coverage

are there certain things where your that change your mind in terms of sex or age or Are there specific patient risk factors that you think of that kind of change medical decision-making?

002- lot of the time with female especially women in child bearing age gotta consider pregnancy we don't always do the pregnancy test and if they're at all …?I usually go to Macrobid and it still reasonably priced and do not give too much trouble about that. guys don't usually get UTI so when are coming in we're also and what we they may have bactrim as well but we usually also do what's called a dirty urine which is looking for gonorrhea and chlamydia and anything else because guys don't usually get you don't usually see it in the young guys, older guy certainly could be prostate related but we don't have the ability to do labs here so I didn't check the PSA. We have had, it comes in waves a test dosent sound like a UTI typical UTI and the way they describe the symptoms that the urine is completely clean the other well hydrated the pH of normal no leukocytes no nitrites it looks beautiful and the way they aaare describing the symptoms just don't sound right and you think it might actually be more bladder spasms so there's a like 30 40 50 year olds usually women who are completely cleaned then I usually do not put them on antibiotics and I talked to them about it might be bladder spasm and then we do like 3 or 4-day course of like one of the methylene blues or something to help with bladder spasms and in the meantime when the culture comes back if it's an infection which is only happened once or twice we are like yes you really have a UTI then we just send out abx.

Reference 7 - 1.30% Coverage

I used to use a lot of Cipro. Cipro is good for older guys because of prostatitis or anything more than just a typical UTI if it was anything else Cipro the nice option and I still may even go to that on occasion but yeah the Black Box warning for the FQ really did back my use of cipro

<Files\\08_15_18_003> - § 4 references coded [10.62% Coverage]

Reference 1 - 4.61% Coverage

when you're seeing the patient and you won't have the culture results cuz you'll be treating them kind of empirically so what what do you prefer usually when you're when you're seeing them here?

003-- of course for non-pregnant non-nursing moms I want to make sure I use the antibiotics that are safe so let's assume it's that case so I might start Keflex or macrobid or sulfa. Those are my top three unless they say I can’t tolerate that, if they had a rash before, nervous about taking a medication so if there's no contraindications to use then those three and perhaps over the years and I think I probably use Cipro a lot more early on but changed over the years with literature coming out saying maybe we need to use this or seeing more cultures that said was seeing a lot of resistance for this than other. And sometimes it's just kind of like people talking or clinicians talking like seeing or trending a lot more resistance to this and we ought to be using cephalexin for the first line or maybe need to get away from the quinolones or watch out for the sulfa reaction. do you want to know about maybe other…….

Reference 2 - 3.36% Coverage

-Of the top three that you mentioned at what goes in your decision-making process about prescribing a cephalosporin or Macrobid or bactrim ?

003--Right….It if I'm if someone's going to really struggle with compliance I want to use twice a day or once a day if I can. I usually take a moment to ask about can you swallow pills, do you think you could do four times a day and get an idea most people are confident oh ya I can remember but reality is not….

If I get this impression that someone is really not compliant I go with twice a day, So I might go with the macrobid or Bactrim in those cases or I might do a different cephalosporin like cefdinir or Augmentin twice a day so if I'm more confident they can handle the four times a day regimen and then go with Keflex or ampicillin sometimes that pretty rare.

Reference 3 - 1.52% Coverage

so duration of symptoms is one one thing you look at when you're deciding how long treating for… what are the what are the other symptoms?

003—Recurrence…if I see this patient 3-4 times in the last 5 years or so or in the last couple years or if they had complications, diabetic, someone may be more frail or ill, or chronic diseases I might go a little bit longer.

Reference 4 - 1.13% Coverage

Can manage with follow up if not getting better, if severe complications then go to the ER and may want to prescribe something maybe a little bit safer if the patient could be potentially pregnant so I might go to those that may be keflex or macrobid for three to five days.

<Files\\08_15_18_004> - § 2 references coded [5.83% Coverage]

Reference 1 - 2.89% Coverage

Can you walk me through how you would approach this case of if they showed up to your clinic?

004--Generally we will just see urine after a history and physical obviously I guess not obviously. Always look for CVA tenderness as its kind of game changer as far as antibiotics. Generally what I'll do is send urine for culture if they're in the office and start him on an antibiotic, probably since you said 27 give 5 to 7 days of antibiotics.

Reference 2 - 2.94% Coverage

The only difference I would say is treating older patients with UTI, Itss a different ball point. But that's not really what you're asking its more about young people with uncomplicated bladder infections its pretty straightforward.

M—The older ones can be a little more challenging I guess related to creatinine clearance and other things taking that into account, other medications, QT prolonging drugs etc.

004—Or sometimes not having symptoms.

<Files\\08_23_18_005> - § 3 references coded [9.45% Coverage]

Reference 1 - 3.45% Coverage

Yeah. And then you mentioned that you like to use macrobid as your first line agent. Can you tell me what your thoughts are about different antibiotics, and why you like macrobid compared to others?

005: I just feel like macrobid has a lower side effect profile, less people are allergic to macrobid for some reason, cause there's no other drugs in that class, I feel like unless they’re menopausal, after 50, I feel like most are not resistant to it and it’s just kinda the go -to as far as

evidence-based practice from when I was in school that was kinda just what we were taught, especially with cipro, and their recent side effects and the black box warning and stuff, we’ve kind of stayed away from cipro, but I feel like a lot of our practitioners still use it. I just go with macrobid, morning and night for five to seven days, depends on the severity of symptoms, if they’ve had one recently.

References 2-3 - 6.01% Coverage

That sounds great. Um, so, I think the next question is, what factors do you consider when you’re treating someone? Are there certain exam things that you look at? Does age or sex matter? Can you walk me through some of that as well? It sounds like you mentioned a little bit about age and sex earlier too.

005: Yes, as far as, let’s start with females. Have they had a UTI before? Are these their typical urinary tract infection symptoms? Does this feel like something else? Do they have a history of kidney stones? New sexual partners? Are the symptoms severe or tolerable? And then, like you said as far as females, I mean there could be interstitial cystitis, they could be having bladder spasms. Atrophic vaginitis has a tendency to cause similar UTI symptoms in postmenopausal or

women of that age group. And then younger females, you always wanna question new sexual partners, is there a concern for any STIs, and stuff like that. Those are kinda the guidelines as far as that goes.

M: I think that’s great.

005: And then males, it depends on age. I mean generally a male comes in with urinary symptoms, of a certain age range, up until the age of 40, 45, we’re looking STI, do we have a history of any new sexual partners, do we have a history of UTIs, kidney stones, any prostate issues, any prostatitis, any pain with bowel movements, any blood with ejaculation, just those kind of symptoms. And you kinda have to mainstream it to see kind of what else they answer and then urine dipstick shows and then if a culture comes back, so it just kinda depends.

<Files\\08_23_18_005 (2)> - § 2 references coded [9.39% Coverage]

Reference 1 - 3.45% Coverage

Yeah. And then you mentioned that you like to use macrobid as your first line agent. Can you tell me what your thoughts are about different antibiotics, and why you like macrobid compared to others?

005: I just feel like macrobid has a lower side effect profile, less people are allergic to macrobid for some reason, cause there's no other drugs in that class, I feel like unless they’re menopausal, after 50, I feel like most are not resistant to it and it’s just kinda the go -to as far as

evidence-based practice from when I was in school that was kinda just what we were taught, especially with cipro, and their recent side effects and the black box warning and stuff, we’ve kind of stayed away from cipro, but I feel like a lot of our practitioners still use it. I just go with macrobid, morning and night for five to seven days, depends on the severity of symptoms, if they’ve had one recently.

Reference 2 - 5.94% Coverage

. Um, so, I think the next question is, what factors do you consider when you’re treating someone? Are there certain exam things that you look at? Does age or sex matter? Can you walk me through some of that as well? It sounds like you mentioned a little bit about age and sex earlier too.

005: Yes, as far as, let’s start with females. Have they had a UTI before? Are these their typical urinary tract infection symptoms? Does this feel like something else? Do they have a history of kidney stones? New sexual partners? Are the symptoms severe or tolerable? And then, like you said as far as females, I mean there could be interstitial cystitis, they could be having bladder spasms. Atrophic vaginitis has a tendency to cause similar UTI symptoms in postmenopausal or

women of that age group. And then younger females, you always wanna question new sexual partners, is there a concern for any STIs, and stuff like that. Those are kinda the guidelines as far as that goes.

M: I think that’s great.

005: And then males, it depends on age. I mean generally a male comes in with urinary symptoms, of a certain age range, up until the age of 40, 45, we’re looking STI, do we have a history of any new sexual partners, do we have a history of UTIs, kidney stones, any prostate issues, any prostatitis, any pain with bowel movements, any blood with ejaculation, just those kind of symptoms. And you kinda have to mainstream it to see kind of what else they answer and then urine dipstick shows and then if a culture comes back, so it just kinda depends.

<Files\\09_05_18_007> - § 1 reference coded [2.66% Coverage]

Reference 1 - 2.66% Coverage

1. Usually, elderly or demented patients. What about pyuria, do you consider it under microscopy to be considered, or because 0-5 is normal, over 5 is what comes up as a red flag in our lab.
2. Yeah, I will usually use symptoms still. So if they have piuria and their culture is positive, but they don’t have symptoms I still won’t treat them, so I’ll still usually base it on symptoms, as long as I think they can mount symptoms. For patients that are immunosuppressed or other things that may be different, but I think that’s one of the areas where knowing if they have symptoms is gonna be an important part in your decision making process. Our lab here uses a cutoff of 10 in hospital, so they won’t do a reflex culture if there’s fewer than 10. But there’s the practices vary from place to place.

<Files\\10_10_18_008> - § 1 reference coded [3.20% Coverage]

Reference 1 - 3.20% Coverage

Great. What factors do you consider when you're deciding to treat a UTI? Are there certain exam findings or do you think age or sex matters? Are there other factors that go into your treatment decisions?

8: So I think age certainly does matter. I think sex does matter of course. … In a male is a whole different issue. Obviously they're having fevers, nausea, vomiting, any systemic symptoms, that's a problem. That's probably my initial factors.

<Files\\10_10_18_008 (2)> - § 1 reference coded [3.14% Coverage]

Reference 1 - 3.14% Coverage

What factors do you consider when you're deciding to treat a UTI? Are there certain exam findings or do you think age or sex matters? Are there other factors that go into your treatment decisions?

8: So I think age certainly does matter. I think sex does matter of course. … In a male is a whole different issue. Obviously they're having fevers, nausea, vomiting, any systemic symptoms, that's a problem. That's probably my initial factors.

<Files\\11_14_18_009> - § 4 references coded [9.00% Coverage]

Reference 1 - 4.07% Coverage

There’s been instances where the culture came back positive, but the dip was negative, for example.

M: So if you have a strong clinical suspicious but the dip is negative you’ll still send a culture out.

009:Yeah, definitely. Like I said, I read in residency we had a form about this in one of our conferences. Whether you would treat somebody regarding this, and the 2 basic things that they were talking about were that you always wanna get a urinalysis for sure, but a woman’s symptoms sometimes aren’t very accurate, and saying if you have a UTI over a dipstick sometimes, you know? Basically the background knowledge I would use to make a decision

Reference 2 - 1.57% Coverage

is there anything that makes you lean one way or the other?

009: Um, you know uh if they have no allergies or anything, not really. I think it’s just how much it’s gonna cost a patient and if they’ve taken something before that they’ve tolerated well.

References 3-4 - 3.35% Coverage

M: Got it. Then what factors do you consider when you’re treating a uti. You mentioned the patient’s symptoms, do age, or comorbidity or sex matter at all?

009: Well yeah. If it’s a male then there’s a totally different thinking. The stuff that I was answering about would primarily be for a female. For a male who has a UTI or symptoms, I’d definitely wanna make sure its not an STD, something that’s causing urethritis, and then after that Id be concerned about a prostate. It’d be a completely different antibiotic course all together.

<Files\\12_18_18_010> - § 5 references coded [26.31% Coverage]

Reference 1 - 6.91% Coverage

I would do a physical exam. I’d ask her if there’s blood in her urine, does she think that she’s, sometimes people can link symptoms to something like sitting in a hot tub or sexual intercourse, see if anything has changed from normal activity. I'd get a urine sample from her, and do a urine dip, and if it’s positive or looks like traces of blood, then definitely send for culture. In a young, healthy female, if I’m convinced she has a UTI, we’ll definitely send for culture as well but put her on 3 days of therapy, oral therapy twice a day, maybe septra, depending on her allergies of course. And I usually don’t bring people back for recheck unless symptoms come back, advise them to drink water/fluid avoid caffeine, alcohol, activities that would irritate the urinary tract. If you have any fever, chills, etc.

References 2-3 - 7.96% Coverage

J: Okay, since we’re talking about treatment, what factors do you consider when deciding when or when not to treat a UTI? Do you consider symptoms, age, sex or other risk factors?

010: Well, when did it start? If it just started this morning, that’s a bit different than if it’s going on for several days. Especially in older women, they can confuse urinary frequency and even dysuria with a UTI, when it can be atrophic vaginitis, in younger women it can be a vaginal infection, so I’d wanna find out do you have any discharge. But, let’s say it’s an 85 year old woman whose had symptoms for several days and is very uncomfortable, and the dip is positive for blood, anything else, then I would start her right away. If she’s elderly woman and the symptoms are convincing, I might give a day or 2 of antibiotics and see what that culture comes back with. I don’t want her to get uroseptic if she’s fragile. Does that answer the question?

Reference 4 - 6.64% Coverage

You talked about getting a urine sample and doing a dip, then if you find certain values you send it for culture. Can you talk more about what kind of lab work do you get done?

010: Depending on the age, if it’s a young healthy person, even if the urine is clean, a lot of times I’ll send it off to make sure we didn’t miss anything. Sometimes something shows up in the lab, micro culture sensitivity, but I wouldn’t routinely like a CBP and a CME unless somebody had severe symptoms, you know or if they’re an older person who I’m worried is dehydrated. If there’s blood in urine, and they’ve got a history of renal stones and blood in urine, then I might order an ultrasound with SDB. If they have painless hematuria, definitely gonna do a CT, with stone protocol, so does that..?

Reference 5 - 4.79% Coverage

If a patient calls in complaining of a UTI to your clinic, who takes the call and how does it get triaged?

010: It goes from phone people, to medical secretary of provider, then forwarded to provider. They find out if the provider wants them to come in, or does the provider want to treat sight unseen. So, I would say 9 times out of 10 I’m gonna say you need to come in so i can get some urine. There’s extenuating circumstances, like if a patient is very immunocompromised and I don’t wanna take a chance, but...so no, that’s how it goes from one to the other.

<Files\\12_19_18_011> - § 5 references coded [13.77% Coverage]

Reference 1 - 5.21% Coverage

Can you manage the case over the phone, or do you always have them come in?

011: It really depends on the patient, if it’s one that they’re just susceptible to UTIs or I’ve treated them before. Like the older population, they’re unable to make it in but they can get the medications delivered, then I may go ahead and call them in things just based on the symptoms, but if it’s a younger patient like the one you mentioned, I’d probably have them come in and discuss, because there could be some other things going on that aren’t just UTI related.

References 2-3 - 4.27% Coverage

Okay, what factors do you consider when deciding when or when not to treat a UTI?

011: Usually it comes down to if it is just a very light leukoesterase, and it’s a younger patient, then I try to see if there’s some other issue for dysuria, whether it be hygiene, a yeast infection, or something else going on there. That’s when I usually don’t treat it as UTI. If lukesterases are the only things that are positive, I try to look at other options.

References 4-5 - 4.29% Coverage

Are there certain patient risk factors that influence your decision about treatments?

011: Yeah, so like I said, the older population i tend to treat based on symptoms of if leuks are slightly elevated,I might treat them more because of their susceptibility because of altered mental status that can happen there, and they don’t always clean as well or they wipe the wrong way. Sometimes i treat them more readily than i do the younger population.

<Files\\12_20_18_012> - § 3 references coded [11.32% Coverage]

References 1-3 - 11.32% Coverage

J: What factors do you consider when deciding to treat a UTI?

012: Mostly patient symptoms, depending on what their UA shows, lab work shows

J: Do you consider, age, sex, stuff like that?

012: I think that you’re looking at just treating with symptoms. I more do that in females because it’s more common in females. In males you do question it because it’s more common in females, maybe they should come in for a visit.

J: And if certain patients have certain risk factors, would you treat them differently?

012: Yeah absolutely. If there’s a nursing home patient, or that population that seems to get UTIs more, I definitely would treat them differently.

J: Do you consider different antibiotics for patients who have different risk factors?

012: For like older patients, you have to know what their kidney function looks like before you can describe certain antibiotics.

<Files\\8_13_18_001> - § 3 references coded [9.35% Coverage]

Reference 1 - 2.93% Coverage

you think that it's probably an uncomplicated urinary tract infection and you usually like to get testing, are there times were you don't get testing or do you always like to get ?

001—if somebody calls in or sends a message through the portal and they have frequent UTIs or I'm pretty sure they have one and we don't have openings that they can get in then I something send out antibiotics.

Reference 2 - 3.80% Coverage

you generally do Cipro, do you have a preference or why do you do Cipro over antibiotics?

001—I will use Macrobid if it's somebody that has drug interactions or I'm worried they're pregnant. I really don't use sulfa a whole lot just because there's so many people with allergies and I have had a lot of cases in the past year of older women they go to the Urgent Care and get sulfa and I'm not kidding I've had 5 people end up in the hospital with AKI or kidney failure, so for older women I just don't like it.

Reference 3 - 2.62% Coverage

Do u have a sense about some abx being more potent than others or when you compare Macrobid and Cipro do you have any.

001- Well I think there is some resistance to Macrobid or obviously it takes a longer course so I would use that second choice but I don't obviously and possibly pregnant women or older people can't take Cipro for the medications.

Patient Preference

<Files\\01_08_19_013> - § 1 reference coded [2.58% Coverage]

Reference 1 - 2.58% Coverage

has also started implementing, e visits now too for UTI. I think they’ve implemented both, a nurse triage algorithm and an e-visit too, where providers then see the patient via communication. I don’t think they skype or anything.

013: Right, we’ve gotta meet the needs of the millennials who don’t want to see a doctor. Everything’s online.

<Files\\02_07_19_015> - § 4 references coded [5.12% Coverage]

Reference 1 - 0.67% Coverage

these patients, they demand antibiotic. I say, "Ok, you want antibiotic, give me culture." and then within three days I will call and say, "Hey, hey, stop the antibiotic, this is not, you cannot be on antibiotics".

Reference 2 - 1.45% Coverage

how do you think they should be shared with primary care doctors?

015: I think they should. It would help a lot because a lot of times when the patient comes to you ___ 13:25 and unfortunately before the trend of the antibiotics… to a multi resistant antibiotic resistant infection, we just gave the antibiotic because the patient wanted it and it has been very difficult now to show the patient that you don't have to get antibiotics every time that you want it.

Reference 3 - 1.11% Coverage

when I show them the negative, this is the evidence. This is what CBC says, but they’re just usually interested for infection. I have not seen a UTI. ____13:54 . Look at this, This is what they say "Look at the television". So I try to educate them but then the problem is going to be this. If I don't give up, in some patients, they go to the Urgent Care.

Reference 4 - 1.89% Coverage

: And when you start practicing you're going to see this…. I get a report every month of how many patients went to the emergency room.

M: Oh wow.

015: How many got admitted and how many did not get admitted, therefore they can come clean with that, that they went to ER and they ___14:38 and that's my fault always. So I have to educate the patient. Don't go to ER, come to me. Come to me. And that’s the point. If I don't do what they want in some patients, they cannot understand what I am saying. They do not want to accept my education. I just do it. I am forced to do it because I don't want to do that.

<Files\\08_15_18_002> - § 1 reference coded [2.27% Coverage]

Reference 1 - 2.27% Coverage

which antibiotic do they typically ask for then ?

002—Typically not with UTI. Usually with sinus infections..or bronchitis or pneumonia. I mean they don't have pneumonia but they come in with I need this because I have these symptoms that says I have pneumonia. I am like I am listening to you and you do not have Pneumonia. Not with UTI they don’t come in specifically. if it were about UTIs I don't think it would be good or bad I mean if it's shown to make a difference then sure give it a three.

<Files\\08_15_18_003> - § 1 reference coded [1.05% Coverage]

Reference 1 - 1.05% Coverage

I didn’t want to get that call back and asking me to switch because they didn’t want their tendons to rupture so maybe looking back maybe that did it have some influence on ya on me but more because of the patients are going to read it and call/come back.

<Files\\08_23_18_005> - § 1 reference coded [5.54% Coverage]

Reference 1 - 5.54% Coverage

And then what other antibiotics come to mind for you other than cipro and macrobid, are those the two main ones?

005: Bactrim, double strength, we’ve used a lot. I mean we can use like a cephalosporin if they’ve shown resistance to something in the past. Generally that would be like a last case, or like a keflex, just depends on allergies, like if somebody has an allergy to bactrim, macrobid hasn’t worked for them in the past, we’re going cipro, we’re going keflex, it just kinda depends. Or if they’ve had any past culture that have shown resistance, gotta steer clear of the ones they’ve been resistant to and I just kinda have to go down the list and see on the culture what its been susceptible to in the past.

M: Got it, so they have like an intolerance to macrobid, but they’re okay with everything else, do you have a second line agent that you like to use?

005: Generally I go with Bactrim double strength. I don’t, like, no idea why I’ve just gotten good coverage over the years and nobody’s called back and said like hey this hasn’t worked. And then once again a lot of patients see the cipro warnings when the pharmacist goes over the side effects with them we get a lot of calls back for cipro from pharmacists like “hey do you realize you put them on cipro” and then the patient calls back with a lot of questions. Bactrim double strength generally doesn’t give me that kickback. We don’t get a lot of phone calls back for it.

<Files\\08_23_18_005 (2)> - § 1 reference coded [5.55% Coverage]

Reference 1 - 5.55% Coverage

And then what other antibiotics come to mind for you other than cipro and macrobid, are those the two main ones?

005: Bactrim, double strength, we’ve used a lot. I mean we can use like a cephalosporin if they’ve shown resistance to something in the past. Generally that would be like a last case, or like a keflex, just depends on allergies, like if somebody has an allergy to bactrim, macrobid hasn’t worked for them in the past, we’re going cipro, we’re going keflex, it just kinda depends. Or if they’ve had any past culture that have shown resistance, gotta steer clear of the ones they’ve been resistant to and I just kinda have to go down the list and see on the culture what its been susceptible to in the past.

M: Got it, so they have like an intolerance to macrobid, but they’re okay with everything else, do you have a second line agent that you like to use?

005: Generally I go with Bactrim double strength. I don’t, like, no idea why I’ve just gotten good coverage over the years and nobody’s called back and said like hey this hasn’t worked. And then once again a lot of patients see the cipro warnings when the pharmacist goes over the side effects with them we get a lot of calls back for cipro from pharmacists like “hey do you realize you put them on cipro” and then the patient calls back with a lot of questions. Bactrim double strength generally doesn’t give me that kickback. We don’t get a lot of phone calls back for it.

<Files\\08_23_18_006> - § 2 references coded [8.59% Coverage]

Reference 1 - 4.48% Coverage

A: And is it, why does the black box warning bother you?
A: Because then you get people that get home, they read the side effects, and now they have it, and now it’s your fault. There’s a lot of public distrust of it, and I understand that. I also don’t like the idea, if I start with that where do I go if it doesn’t work?
A: Got it. So if someone’s not being treated with macrobid, or you feel like that’s not effective, then what’s the next antibiotic you go with?
B: If i don’t do macrobid it’ll be the keflex and then the bactrim is kind of my way, and then I’ve done Levaquin a couple times, because people will come in and insist on it. I don’t know why they do that, but they swear that’s what they got the last time and the only thing that works for them.

Reference 2 - 4.11% Coverage

C: Got it. And then, do you think, and you’ve already been measured by this a little bit, but what are your thoughts about if providers should be evaluated by adherence to guidelines, or best practice guidelines?
D: I think that it’s important to make sure that what you’re doing is appropriate, but there has to be wiggle room. It can’t be strict because there’s always going to be other factors that play a part, especially in a culture that promotes patient satisfaction as highly as they promote positive patient outcomes nd appropriateness of decisions. To measure it solely by adherence to guidelines, then there is going to have to be a shirt in that culture of promoting patient satisfaction.

<Files\\11_14_18_009> - § 5 references coded [5.39% Coverage]

Reference 1 - 1.68% Coverage

Um, you know uh if they have no allergies or anything, not really. I think it’s just how much it’s gonna cost a patient and if they’ve taken something before that they’ve tolerated well.

M: Sure.

009: I’d talk with the patient to figure out which one may be better.

References 2-3 - 1.15% Coverage

Sure, I mean a lot of them have had antibiotics for UTIs before so some of them might have preferences too. Do you see that, where they tell you I prefer this one?

009: Absolutely.

References 4-5 - 2.55% Coverage

Then one of the other common antibiotics that people think about for UTIs is Bactrim. What are your thoughts about bactrim?

009: Definitely, but I think cipro and Macrobid I’ve been using more often.

M: Got it, and it sounds like that decision is mostly personal preference and then what the patient prefers too, is that ?

009: Absolutely, there’s no specific indication that I’d use one over the other.

<Files\\12_19_18_011> - § 2 references coded [5.63% Coverage]

Reference 1 - 4.34% Coverage

which would you choose and what duration?

011: Most of the time, if it’s, the only symptom is dysuria and UA is positive and no allergies, ether keflex or cipro. Usually ask what works best for them, because the majority of patients I see, it’s not their first UTI. And so those are the main 2 that i go with. If there’s allergies then sometimes bactrim. Majority of the time treatment is 3-5 days depending on their past history and severity of symptoms.

Reference 2 - 1.28% Coverage

: Are there certain antibiotics which are better or worse that others according to you?

011: No, it really depends on the patient.

<Files\\12_20_18_012> - § 1 reference coded [4.59% Coverage]

Reference 1 - 4.59% Coverage

If clinical guidelines for UTIs existed, do you think that providers should be evaluated on their adherence to them in their practice?

012: No, I don’t think so.

J: Can you explain more?

012: We run into problems with patients not in agreement with how long, or what antibiotic, which I know shouldn’t dictate practice but in the real world it kinda does.

Treatment duration

<Files\\01_08_19_013> - § 4 references coded [6.47% Coverage]

References 1-2 - 3.14% Coverage

So if they called in, you’d clarify some questions and have them go to the lab. If they showed up, clarify some questions and have them go to the lab. Would treat them empirically, or would you wait for culture to come back?

013: Depends on symptoms, that’s sort of the art of medicine. The more concerning symptoms are, the more I give a 2 day dose of an antibiotics, sometimes 3 days and call it quits after that.

References 3-4 - 3.32% Coverage

So, mostly it sounds like potency and then guidelines are usually what you use to select antibiotics. What do you use for treatment duration?

013: Younger people 3-5 days. Some people i know get by on 3 day courses. 5 is probably the most common. Older people 5-7

M: So for younger ones 3-5, usually 5 and sometimes 3 if their symptoms aren’t too bad?

013: Right and sometimes just a track history of 3 day antibiotics working well.

<Files\\01_10_19_014> - § 2 references coded [6.64% Coverage]

References 1-2 - 6.64% Coverage

How will you handle the case?

014: With somebody like that, we do the Udip in the office. If there’s anything that seems generally positive, leukocytes or nitrates or anything like that, we’ll go ahead and treat them, if it’s uncomplicated and they don’t do it all the time, I usually do 3-5 days of macrobid. It kinda depends, I stick more towards 5. It might be overkill, but since they usually aren’t in my setting, we’re primary care but we also do walk in, and we’re in a tourist area so we get people we’re never gonna see again. We kinda wanna send them off adequately set up, and then we’ll send that urine for culture. FOr everybody that has insurance we send the culture, sometimes if they don’t we give them the choice. Otherwise we just go with it and tell them to follow up if they don’t have resolution of symptoms.

<Files\\02_07_19_015> - § 3 references coded [3.30% Coverage]

References 1-2 - 2.46% Coverage

What duration?

M: Yes.

015: In the simple uncomplicated, would be a single dose.

M: Ok.

015: It could be a single dose. If it's a little more long....some patients don't feel comfortable, if she is older or...then I might give her three to seven days depending on how I submitted the urine and if I feel that it is going to be very easy...the culture is probably going to be negative, I don't give too many antibiotics.

M: Ok.

015: If it is over the weekend then I going to have to extend antibiotics because I don't want them to go to the Emergency Room.

M: Ah, for sure.

015: So, if the culture is done, say on a Thursday, Friday, then I’ll start with giving them more antibiotics, but if it's done on a Monday through Wednesday then I'll feel more comfortable calling ___ 07:38

Reference 3 - 0.84% Coverage

: I know that…. It’s just…. if it's simple, a single dose. If it's a little more complicated, six, seven days. In a ______ 11:50 with comorbid conditions, seven, maybe fourteen days. You just, I don't know it’s just like, depending how the wind is, how I feel about it.

<Files\\08_15_18_002> - § 4 references coded [8.50% Coverage]

Reference 1 - 4.86% Coverage

what are the other choices that you usually think about when u cannot use Bactrim?

002-- I am very fond of Macrobid, also very inexpensive , very well tolerated, test if there's a chance they could be pregnant nice to know that it's safe as beyond that I used to do Cipro but the one of the primaries that I worked with she would do super 250 for 3 days never worked. I would get the calls saying I saw this doctor so an so and I've been taking the antibiotic and it didn't get better It didn't change and I look in the chart its Cipro 250th 3 days it didn't work, so I would do Cipro 500 but usually for 5 to 7 days but then this whole thing with the fluoroquinolones in the Black Box warning and then and now try to stay away from any of the fluoroquinolones if I can. So ironically especially if they've already done bactrim or Macrobid go to one of the cephalosporins or even amoxicillin not the best choice but still would hit a lot of the possible bacteria and again very well tolerated very inexpensive usually works pretty well not my first choice but it's an option

References 2-3 - 2.64% Coverage

then you mentioned too that the you know based on your colleagues experienced with the three days in the low dose of the Cipro that you didn't work very well do you what duration do you usually do for the Macrobid and the Bactrim.

002-- I usually do 7 days if they're really sensitive I might be 5 days ,you know if they're like I take it every time and really making sure I'm drinking cranberry water and I just bought her so much that I pee all the time not because of infection and and they're they're trying to do all the right things and it back it down to 5 days otherwise 7.

Reference 4 - 1.00% Coverage

I would say okay with Bactrim DS 1 PO BID 7 Days sent to the pharmacy and then the MA would call them and tell them okay doctor sent antibiotics to the pharmacy, if you dont get better you need to make an appointment.

<Files\\08_15_18_003> - § 4 references coded [8.95% Coverage]

References 1-3 - 7.81% Coverage

Of the top three that you mentioned at what goes in your decision-making process about prescribing a cephalosporin or Macrobid or bactrim ?

003--Right….It if I'm if someone's going to really struggle with compliance I want to use twice a day or once a day if I can. I usually take a moment to ask about can you swallow pills, do you think you could do four times a day and get an idea most people are confident oh ya I can remember but reality is not….

If I get this impression that someone is really not compliant I go with twice a day, So I might go with the macrobid or Bactrim in those cases or I might do a different cephalosporin like cefdinir or Augmentin twice a day so if I'm more confident they can handle the four times a day regimen and then go with Keflex or ampicillin sometimes that pretty rare.

M—Do you have a specific duration for UTIs?

003—I usually pick 5 to 7 Days, I know 3 days is out there.I guess cuz I see a lot of people come back like 2 to 3 days and it seemed to come back maybe just in my mind three days should be adequate but then there's a range 3 to 5 days, I don't want them calling back and or returning back unsatisfied or thinking she got this thing again what's going on….perhaps if I had treated longer yeah maybe I could have avoided that. Those are some of the influences that make me go longer. I think 7 days will be my real average but Maybe they come in and there's just one and a half days or just sometimes 3 hours of symptoms , maybe just starting… I might go 3 to 5 days.

M-- so duration of symptoms is one one thing you look at when you're deciding how long treating for… what are the what are the other symptoms?

003—Recurrence…if I see this patient 3-4 times in the last 5 years or so or in the last couple years or if they had complications, diabetic, someone may be more frail or ill, or chronic diseases I might go a little bit longer.

Reference 4 - 1.13% Coverage

Can manage with follow up if not getting better, if severe complications then go to the ER and may want to prescribe something maybe a little bit safer if the patient could be potentially pregnant so I might go to those that may be keflex or macrobid for three to five days.

<Files\\08_15_18_004> - § 3 references coded [5.52% Coverage]

References 1-2 - 2.89% Coverage

Can you walk me through how you would approach this case of if they showed up to your clinic?

004--Generally we will just see urine after a history and physical obviously I guess not obviously. Always look for CVA tenderness as its kind of game changer as far as antibiotics. Generally what I'll do is send urine for culture if they're in the office and start him on an antibiotic, probably since you said 27 give 5 to 7 days of antibiotics.

Reference 3 - 2.64% Coverage

Got it and then in terms of the testing do you like to test it everybody or do your testing practices change if they call you on the telephone or send a message to you instead.

004--If they're not in person we would probably just in this age group so younger patient without any other symptoms and recognize symptoms as bladder infection I don't hesitate to treat over the phone with antibiotics 5-7 days

<Files\\08_23_18_005> - § 3 references coded [8.12% Coverage]

Reference 1 - 3.45% Coverage

Yeah. And then you mentioned that you like to use macrobid as your first line agent. Can you tell me what your thoughts are about different antibiotics, and why you like macrobid compared to others?

005: I just feel like macrobid has a lower side effect profile, less people are allergic to macrobid for some reason, cause there's no other drugs in that class, I feel like unless they’re menopausal, after 50, I feel like most are not resistant to it and it’s just kinda the go -to as far as

evidence-based practice from when I was in school that was kinda just what we were taught, especially with cipro, and their recent side effects and the black box warning and stuff, we’ve kind of stayed away from cipro, but I feel like a lot of our practitioners still use it. I just go with macrobid, morning and night for five to seven days, depends on the severity of symptoms, if they’ve had one recently.

References 2-3 - 4.67% Coverage

Got it. And then what about selecting the duration for antibiotics? Is that usually in your existing algorithms, or how do you pick how long to treat someone?

005: It was in existing algorithms as far as antibiotics, but I mean I think less is better with an antibiotic, I mean they give you 5 to 7 days, if you’ve taken this antibiotic before what’s seemed to work, how bad the symptoms are, how long they’ve had it. But generally I try to use the lesser side of things versus the more extreme unless the patient tells me that they’ve had a history or

they put me on it for this long and it didn’t work, just because like I said with antibiotics I’m a little more conservative. I’m not a fan of putting somebody on something for longer than they need it that would get rid of their bacteria, or infection.

M: Do you follow that same 5 to 7 day rules for all the different for all UTI antibiotics or does it change based on the antibiotic?

005: It changes based on the antibiotic. I mean I think macrobid you can get away with 5 on the lesser strength, bactrim 5 to 7, and then cipro I mean if I’m gonna do it on cipro then generally 3 to 5, I don’t think cipro needs as long of a course unless there’s a history.

<Files\\08_23_18_005 (2)> - § 2 references coded [8.12% Coverage]

Reference 1 - 3.45% Coverage

Yeah. And then you mentioned that you like to use macrobid as your first line agent. Can you tell me what your thoughts are about different antibiotics, and why you like macrobid compared to others?

005: I just feel like macrobid has a lower side effect profile, less people are allergic to macrobid for some reason, cause there's no other drugs in that class, I feel like unless they’re menopausal, after 50, I feel like most are not resistant to it and it’s just kinda the go -to as far as

evidence-based practice from when I was in school that was kinda just what we were taught, especially with cipro, and their recent side effects and the black box warning and stuff, we’ve kind of stayed away from cipro, but I feel like a lot of our practitioners still use it. I just go with macrobid, morning and night for five to seven days, depends on the severity of symptoms, if they’ve had one recently.

Reference 2 - 4.67% Coverage

Got it. And then what about selecting the duration for antibiotics? Is that usually in your existing algorithms, or how do you pick how long to treat someone?

005: It was in existing algorithms as far as antibiotics, but I mean I think less is better with an antibiotic, I mean they give you 5 to 7 days, if you’ve taken this antibiotic before what’s seemed to work, how bad the symptoms are, how long they’ve had it. But generally I try to use the lesser side of things versus the more extreme unless the patient tells me that they’ve had a history or

they put me on it for this long and it didn’t work, just because like I said with antibiotics I’m a little more conservative. I’m not a fan of putting somebody on something for longer than they need it that would get rid of their bacteria, or infection.

M: Do you follow that same 5 to 7 day rules for all the different for all UTI antibiotics or does it change based on the antibiotic?

005: It changes based on the antibiotic. I mean I think macrobid you can get away with 5 on the lesser strength, bactrim 5 to 7, and then cipro I mean if I’m gonna do it on cipro then generally 3 to 5, I don’t think cipro needs as long of a course unless there’s a history.

<Files\\08_23_18_006> - § 5 references coded [16.94% Coverage]

References 1-2 - 6.92% Coverage

A: I would talk to her more about her symptoms, find out if there’s anything else going on, are you sexually active, is there a chance that this could be an STI, is there any chance you could be pregnant, even though you don’t think you are at the moment? Are you having frequency, urgency, hesitancy, is there blood in your urine, you ever have a kidney stone before? Fevers, back pain, abdominal pain, anything else going on? Then I would, the girls are really good about getting the urine-dip, talk to the patient about, well it looks like maybe there might be something, you’ve got some, if she’s got leukocytes or nitrites maybe she’s got something else going on. We can try an antibiotic, I would probably start with macrobid, twice a day for 7 days, unless they wanna balk at it. I would send it for culture no matter what. If I didn’t see anything that was indicative, I would try to talk them out of insisting on an antibiotic. I’d tell them I just really don’t see anything. What’s in the culture, if it comes back we can always start you on something, but it doesn’t really look like that’s where the problem is, so why don’t you follow up with primary or urologist.

Reference 3 - 8.03% Coverage

B: If you’re thinking about alternatives or other agents, what are the other antibiotics you usually think of?
A: Keflex, I’ll do Keflex 4 times a day for 10 days. Sometimes I’ll use bactrim, that one does

seem to come up more often as one that has resistance so I try to do keflex over it but if I look at somebody and they tell me they’re not gonna take it 4 times a day, okay bactrim it is. I don’t do cipro unless I don’t feel that there’s any other option.

A: Got it. And why do you avoid cipro?
B: It has a black box warning, I don’t like to prescribe things like that if I don’t have to.
A: And is it, why does the black box warning bother you?
A: Because then you get people that get home, they read the side effects, and now they have it, and now it’s your fault. There’s a lot of public distrust of it, and I understand that. I also don’t like the idea, if I start with that where do I go if it doesn’t work?
A: Got it. So if someone’s not being treated with macrobid, or you feel like that’s not effective, then what’s the next antibiotic you go with?
B: If i don’t do macrobid it’ll be the keflex and then the bactrim is kind of my way, and then I’ve done Levaquin a couple times, because people will come in and insist on it. I don’t know why they do that, but they swear that’s what they got the last time and the only thing that works for them.

References 4-5 - 1.99% Coverage

A: And then you mentioned for the macrobid, I think 5 to 7 days or so is the…
B: I usually do 7 days.
A: 7. And then what about the other antibiotics that we mentioned, do you usually do them for around 7 too, or does it vary?
B: I do the keflex for 10, I do bactrim for 7, and I think I look the cipro up every time I have to do that one.

<Files\\09_05_18_007> - § 8 references coded [10.52% Coverage]

References 1-2 - 2.48% Coverage

A: in terms of management, if the patient called over the phone, would you have them come in over the phone for a urine dip, or what do you do if they call over the phone?
B: I think I would like to treat patients like this over the phone so that they don’t have to go to the ER, Urgent Care. It can be safely treated by starting some medications that I have favored to treat simple UTIs. I usually use either bactrim DS, if there’s no allergy of course, 1 tablet twice a day. I usually do 5 day course, there are times I tell patients to use it 3 days and their symptoms have subsided within 48 hours they can do a short course of 3 days, but 5 days is what I send the patient a prescription. My second choice is macrobid, twice a day for 5 days.

References 3-7 - 6.81% Coverage

C: are there specific guidelines or resources you use when you’re trying to select antibiotics and look into treatment duration?
D: Honestly, obviously I use up to date a lot, time to time I’ll use my AFP journals, reviews and all that. But I do use up to date for, at least I have 2 or 3 times a day or more, up to date is up so I can look up something. So, most of the guidelines it from ED, and it’s from the TMEs I’ve attended, that kind of stuff, some of the experience of that that I follow. There are times when I see patients getting antibiotics for 7 to 10 days, where routinely they come to me and say by the way urgent care gave me that, I do not think it’s necessary to treat patients for longer duration of antibiotics for simple UTIs, so I kind of educate them about that.
A: Tell me more about that for urgent cares. So you’ll see them often prescribe for a longer treatment duration, are there certain antibiotics they tend to prescribe, too?
B: Yeah, so I think they do like bactrim, I see a lot patients come on bactrim, 7 ays is the most commonly what they give. Cipro is the second one, 5 day, twice a day, is very common that I see patients coming with the prescription.
A: Do you find that it’s difficult, or how do patients respond when they get a different treatment from urgent care or a different provider than what you’re recommending? It sounds like they often do longer durations, in your opinion, so is there any issue?
B: I think it’s, when you have a relationship to the patient, they trust you. It’s the education to the patient that, I understand that you have this infection, you went to urgent care. If they have done the cultures, I’d like to get that. It takes about 48-72 hours, but in the meanwhile, I would like you to, with your symptoms you should be okay to do 5 day course to the 7 day course. They really appreciate that, because they don’t want it to be on this antibiotic, so for the most common UTI symptoms, I will tell them what else to look for if they start getting worse or something like that.

Reference 8 - 1.23% Coverage

A: And what are your thoughts about a patient with multidrug resistant UTI who is simply coming with a simple symptom not generalized symptoms or red flags. There are some infections where it’s okay to treat them, with 10 days of whatever sensitivity they have, or there are some infections doctors that no, it treated be treated with antibiotics, preferably meropenem.

<Files\\10_10_18_008> - § 1 reference coded [5.59% Coverage]

Reference 1 - 5.59% Coverage

: Can you tell me about antibiotic treatment duration? Do you have a preferred duration that you give to patients when your prescribing Cipro or Bactrim?

8: Yeah. I would say usually 5 even though I know probably 3 is probably enough, I tend to go five.

M: What are your thoughts on 5 verses 3 or 7? Is it mostly because that's what you're familiar with or are there other things that come to mind when you are trying to make some of those decisions?

8: I think the five is mostly what I was familiar with. Certainly, I guess, if it was more complicated, it would need to potenitally be longer, but I just, based on no good evidence, I felt like three might not be enough, so I always just used to go with five. I'm sure that makes you quake as an infectious disease doctor, I'm sorry.

<Files\\10_10_18_008 (2)> - § 1 reference coded [5.58% Coverage]

Reference 1 - 5.58% Coverage

Can you tell me about antibiotic treatment duration? Do you have a preferred duration that you give to patients when your prescribing Cipro or Bactrim?

8: Yeah. I would say usually 5 even though I know probably 3 is probably enough, I tend to go five.

M: What are your thoughts on 5 verses 3 or 7? Is it mostly because that's what you're familiar with or are there other things that come to mind when you are trying to make some of those decisions?

8: I think the five is mostly what I was familiar with. Certainly, I guess, if it was more complicated, it would need to potenitally be longer, but I just, based on no good evidence, I felt like three might not be enough, so I always just used to go with five. I'm sure that makes you quake as an infectious disease doctor, I'm sorry.

<Files\\11_14_18_009> - § 2 references coded [1.70% Coverage]

References 1-2 - 1.70% Coverage

How long do you usually treat patients with UTIs for?

009: MOst of the time, 7 to 10 days.

M: Is that for both of them, cipro and macrobid about the same?

009: I think macrobid, I think cipro we do for 10 days, I think macrobid is 7 to 10. I think mostly we do 10.

<Files\\12_18_18_010> - § 4 references coded [17.58% Coverage]

References 1-2 - 6.91% Coverage

I would do a physical exam. I’d ask her if there’s blood in her urine, does she think that she’s, sometimes people can link symptoms to something like sitting in a hot tub or sexual intercourse, see if anything has changed from normal activity. I'd get a urine sample from her, and do a urine dip, and if it’s positive or looks like traces of blood, then definitely send for culture. In a young, healthy female, if I’m convinced she has a UTI, we’ll definitely send for culture as well but put her on 3 days of therapy, oral therapy twice a day, maybe septra, depending on her allergies of course. And I usually don’t bring people back for recheck unless symptoms come back, advise them to drink water/fluid avoid caffeine, alcohol, activities that would irritate the urinary tract. If you have any fever, chills, etc.

Reference 3 - 7.89% Coverage

since we’re talking about treatment, what factors do you consider when deciding when or when not to treat a UTI? Do you consider symptoms, age, sex or other risk factors?

010: Well, when did it start? If it just started this morning, that’s a bit different than if it’s going on for several days. Especially in older women, they can confuse urinary frequency and even dysuria with a UTI, when it can be atrophic vaginitis, in younger women it can be a vaginal infection, so I’d wanna find out do you have any discharge. But, let’s say it’s an 85 year old woman whose had symptoms for several days and is very uncomfortable, and the dip is positive for blood, anything else, then I would start her right away. If she’s elderly woman and the symptoms are convincing, I might give a day or 2 of antibiotics and see what that culture comes back with. I don’t want her to get uroseptic if she’s fragile. Does that answer the question?

Reference 4 - 2.78% Coverage

any specific resources or guidelines when picking antibiotics or treatment durations?

010: I do, sometimes I use hypocrates, or up to date, so I’ll access those. Also I get prescriber's’ letter, the guidelines sometimes shift. I know there’s a one day therapy now they’re recommending as well, so I’ll follow those guidelines.

<Files\\12_19_18_011> - § 3 references coded [8.94% Coverage]

References 1-2 - 4.70% Coverage

If it shows that she does have a UTI, which would you choose and what duration?

011: Most of the time, if it’s, the only symptom is dysuria and UA is positive and no allergies, ether keflex or cipro. Usually ask what works best for them, because the majority of patients I see, it’s not their first UTI. And so those are the main 2 that i go with. If there’s allergies then sometimes bactrim. Majority of the time treatment is 3-5 days depending on their past history and severity of symptoms.

Reference 3 - 4.24% Coverage

previous studies have demonstrated that nursing staff can successfully follow simple algorithms for UTIs, freeing up clinician time for more complicated decisions. What are your thoughts about this?

011: I think that’s great. I know that they do that a lot with telvid type stuff and it’s very useful. It’s only a 3 day treatment, so if they’re not better they can definitely come in and make an appointment cause they failed outpatient treatment.

<Files\\12_20_18_012> - § 3 references coded [5.71% Coverage]

References 1-2 - 2.23% Coverage

: So you said you’d choose to treat with antibiotics, can you tell me which ones and for what duration?

012: I would maybe use probably Bactrim DS 1 twice a day for 7 days.

Reference 3 - 3.49% Coverage

Do you use any specific resources or guidelines?

012: Not really, I usually like get a UA with the culture and I’ll treat them, and if we have recurrent UTIs, then I think about treating for a longer duration. I don’t think there’s any specific guidelines that I’m following

<Files\\8_13_18_001> - § 3 references coded [5.27% Coverage]

References 1-2 - 2.65% Coverage

. So can you kind of approach how you would deal with that in a clinic setting if you saw that patient?

001-- so obviously we check a urine dip in the office make sure she is not pregnant. You know if she doesn't have any allergies, check for other medications and usually I'll give a 3-day course of Cipro if I'm not concerned that somebody is pregnant.

Reference 3 - 2.62% Coverage

Do u have a sense about some abx being more potent than others or when you compare Macrobid and Cipro do you have any.

001- Well I think there is some resistance to Macrobid or obviously it takes a longer course so I would use that second choice but I don't obviously and possibly pregnant women or older people can't take Cipro for the medications.

UTI Treatment plan

<Files\\01_08_19_013> - § 2 references coded [8.37% Coverage]

Reference 1 - 5.21% Coverage

I’d ask for more symptoms, see if there’s more evidence for a UTI although I’d probably have enough to get a urinalysis to start. She doesn’t have a lot of symptoms so I’d send of a UA with CNS for 99% of the time then wait for a culture to prescribe antibiotics.

M: Would your management with this be different if they called in vs showed up to clinic?

013: Normally I’d still recommend they go to a local lab and get a UA with CNS. At times I do treat empirically over the phone, but it’s really only if there’s somewhat unusual circumstances that precludes getting a timely UA with CNS.

M:Just to clarify, CNS are you talking about a reflex, or is that a micro?

013: Reflex, yeah.

Reference 2 - 3.15% Coverage

Then, do you ever prescribe antibiotics kind of, do you wait ever to prescribe Abx while you’re waiting for the culture to come back? It sounds like you do, what circumstances is that?

013: Low level symptoms no fever, no risk of sepsis, mildly irritating symptoms

M:Then what antibiotics do you prefer to use in this circumstance?

013: I would try and use sulfa drug or ampicillin first, escalate up as needed.

<Files\\01_10_19_014> - § 2 references coded [8.39% Coverage]

Reference 1 - 6.34% Coverage

With somebody like that, we do the Udip in the office. If there’s anything that seems generally positive, leukocytes or nitrates or anything like that, we’ll go ahead and treat them, if it’s uncomplicated and they don’t do it all the time, I usually do 3-5 days of macrobid. It kinda depends, I stick more towards 5. It might be overkill, but since they usually aren’t in my setting, we’re primary care but we also do walk in, and we’re in a tourist area so we get people we’re never gonna see again. We kinda wanna send them off adequately set up, and then we’ll send that urine for culture. FOr everybody that has insurance we send the culture, sometimes if they don’t we give them the choice. Otherwise we just go with it and tell them to follow up if they don’t have resolution of symptoms.

Reference 2 - 2.05% Coverage

You mentioned macrobid, are there other antibiotics that you consider?

014: I do, usually it’s between macrobid and bactrim for those that age group. You said 27? Yeah, those 2 for that age group, especially if they don’t have a lot of recurrent UTIs.

<Files\\02_07_19_015> - § 1 reference coded [1.09% Coverage]

Reference 1 - 1.09% Coverage

015: And it's a young person. First time maybe. We don't even know what it is exactly. We think that it's uncomplicated and so if I have a urinalysis that sort of, changes her mind, I could talk to her into just waiting to see what happens and see just symptomatically, and hydration, over the counter medications and wait before we start antibiotics.

<Files\\08_15_18_002> - § 1 reference coded [11.92% Coverage]

Reference 1 - 11.92% Coverage

Actually it makes a difference I was a primary care for 18 years and actually approached it different here than there. If she was a established patient and seen her in the last several months and was someone I was familiar with wouldnt necessarily make her come in. based on her symptoms and if I knew her and was reasonable and not over dramatic and no other medical problems and nothing to check, I would probably send out antibiotics for her without making her come in especially if I didn't have any openings in my clinic. So instead of making her wait and frequently we did not have openings then we would say send out antibiotics and say if your symptoms don't improve you'll have to come in and make an appointment to see me, so that was Primary Care. Here in Convenient Care they come in with the symptoms everyone gets a UA and based on those symptoms even if the UA came back with negative nitrites and even trace leukocytes still probably would treat but we always here we always send cultures unless they are self-pay, so here they get a culture and if self-pay we will go back and forth and talk to them and this is how much it's going to cost but it'll give us these benefits to make sure it is an infection by using the right medicine to treat for and most of the time they do not want the self-paid do not want the culture. People when it's not self-pay their like okay sure we get the cultures back and I'd say probably the majority of the time there is an infection but there's probably 30 to 35% maybe even 40% when there is not an infection and so will call him back and we'll say stop the antibiotics if you're still having symptoms you need to follow up with your primary care or whoever your OB-GYN sometimes if they don't have a PCP but in that case someone who has no medical problems 2 days of symptoms. that urine dip, the UA comes back showing leukocytes, nitrites, even trace of blood but I would probably still then that is probably give Bactrim it's inexpensive it's well tolerated it usually works for most people so it makes good sense in all the cultures most of the cultures we had have come back sensitive to bactrim so it's nice and easy. If it is not improving they have to come back and see us. So we will not call out medicine here at Convenient Care, that will not be done even if you were just seeing for that unless they have a reaction to the medication in which case they will call later that day or the next day and say I have a rash all over I just started this, the only thing that changed I need to change my antibiotic. We will probably change their abx unless there is a new symptom.

<Files\\08_15_18_003> - § 3 references coded [3.89% Coverage]

Reference 1 - 1.62% Coverage

M--And then it when you're seeing the patient and you won't have the culture results cuz you'll be treating them kind of empirically so what what do you prefer usually when you're when you're seeing them here?

003-- of course for non-pregnant non-nursing moms I want to make sure I use the antibiotics that are safe so let's assume it's that case so I might start Keflex or macrobid or sulfa.

Reference 2 - 1.27% Coverage

so if there's no contraindications to use then those three and perhaps over the years and I think I probably use Cipro a lot more early on but changed over the years with literature coming out saying maybe we need to use this or seeing more cultures that said was seeing a lot of resistance for this than other

Reference 3 - 1.00% Coverage

kind of like people talking or clinicians talking like seeing or trending a lot more resistance to this and we ought to be using cephalexin for the first line or maybe need to get away from the quinolones or watch out for the sulfa reaction.

<Files\\08_15_18_004> - § 1 reference coded [2.65% Coverage]

Reference 1 - 2.65% Coverage

Got it and then in terms of the testing do you like to test it everybody or do your testing practices change if they call you on the telephone or send a message to you instead.

004--If they're not in person we would probably just in this age group so younger patient without any other symptoms and recognize symptoms as bladder infection I don't hesitate to treat over the phone with antibiotics 5-7 days.

<Files\\08_23_18_006> - § 1 reference coded [2.85% Coverage]

Reference 1 - 2.85% Coverage

A: I would probably start with macrobid, twice a day for 7 days, unless they wanna balk at it. I would send it for culture no matter what. If I didn’t see anything that was indicative, I would try to talk them out of insisting on an antibiotic. I’d tell them I just really don’t see anything. What’s in the culture, if it comes back we can always start you on something, but it doesn’t really look like that’s where the problem is, so why don’t you follow up with primary or urologist.

<Files\\09_05_18_007> - § 2 references coded [2.07% Coverage]

Reference 1 - 0.88% Coverage

B: I know that routinely we do a urine dipstick before seeing a patient. That is still, I do it, however I think that classic symptoms in a patient like her can be treated with a short course of antibiotics. In a real world, in my practice, I do do a urine dipstick.

Reference 2 - 1.19% Coverage

A: Do you get a culture on them too, or no?
B: I don’t think I would do cultures, because she is presenting as very simple cystitis clinical symptoms without any fever, which is about more than 99.8, so 99.8 or 99.9, or 100 degree temperature, malaise, fatigue, or tenderness, or prior history of a urinary tract infection, I don’t think a culture is needed.

<Files\\10_10_18_008> - § 3 references coded [4.39% Coverage]

Reference 1 - 1.32% Coverage

If she was in my clinic I would likely get a UA and depending how symptomatic she was, potentially start her on prophylactic or empiric treatment until I had the results of the UA back.

Reference 2 - 0.91% Coverage

M: So you mentioned you’d get urine tests. What kind of tests do you like to do?

8: Oh, I do the UA with reflex to culture.

Reference 3 - 2.15% Coverage

Got it okay. And would it change if the patient, if you got a message or got a telephone call about a patient having these symptoms? Would you still get testing or would you treat them empirically? Can you walk me through that?

8: If it was on a phone call, I would probably just treat them empirically

<Files\\11_14_18_009> - § 2 references coded [10.35% Coverage]

Reference 1 - 3.60% Coverage

I’d a thorough physical on the patient, then I would get a urinalysis dip in the clinic. And then, based off this history and her symptoms and the urine dip, make a decision going forward. So typically, we don’t treat anybody unless they give us a urinalysis. Except maybe in some very specific cases, you know, that we’d do that without a urinalysis. But the urinalysis results would probably 95% of the time be an indicator to treat or not to treat. But, you know, if there’s somebody who has definitie symptoms that sounds like a UTI, wouldn’t necessarily not treat her either.

Reference 2 - 6.75% Coverage

Got it. And then, you mentioned getting a urinalysis, is it usually a urine dipstick that you get in clinic?

009: Yeah, so we’d do a urine dipstick in the clinic, and then if that is abnormal, then we’d send it to the lab for UA and urine culture. Now if somebody has a UTI, their urinalysis is negative on the dip but I still suspect that it’s a UTI, then I’ll still send the urinalysis off to the lab.

M: Got it.

009: There’s been instances where the culture came back positive, but the dip was negative, for example.

M: So if you have a strong clinical suspicious but the dip is negative you’ll still send a culture out.

009:Yeah, definitely. Like I said, I read in residency we had a form about this in one of our conferences. Whether you would treat somebody regarding this, and the 2 basic things that they were talking about were that you always wanna get a urinalysis for sure, but a woman’s symptoms sometimes aren’t very accurate, and saying if you have a UTI over a dipstick sometimes, you know? Basically the background knowledge I would use to make a decision.

<Files\\12_18_18_010> - § 1 reference coded [4.86% Coverage]

Reference 1 - 4.86% Coverage

I'd get a urine sample from her, and do a urine dip, and if it’s positive or looks like traces of blood, then definitely send for culture. In a young, healthy female, if I’m convinced she has a UTI, we’ll definitely send for culture as well but put her on 3 days of therapy, oral therapy twice a day, maybe septra, depending on her allergies of course. And I usually don’t bring people back for recheck unless symptoms come back, advise them to drink water/fluid avoid caffeine, alcohol, activities that would irritate the urinary tract. If you have any fever, chills, etc.

<Files\\12_19_18_011> - § 2 references coded [7.01% Coverage]

Reference 1 - 1.53% Coverage

: 2 days of dysuria and urinary frequency.

011: Okay, so I’d probably order a UA, initially, and then do we have nay vital signs, is she febrile or anything?

Reference 2 - 5.48% Coverage

Okay, would you choose to treat with antibiotics?

011: Depends on the UA.

J: If it shows that she does have a UTI, which would you choose and what duration?

011: Most of the time, if it’s, the only symptom is dysuria and UA is positive and no allergies, ether keflex or cipro. Usually ask what works best for them, because the majority of patients I see, it’s not their first UTI. And so those are the main 2 that i go with. If there’s allergies then sometimes bactrim. Majority of the time treatment is 3-5 days depending on their past history and severity of symptoms.

<Files\\12_20_18_012> - § 3 references coded [6.14% Coverage]

Reference 1 - 2.30% Coverage

I would probably want to get a UA before doing anything. And then, depending on what that showed, possibly treat her with antibiotics. What else do you want to know? Which antibiotic?

Reference 2 - 0.82% Coverage

I would maybe use probably Bactrim DS 1 twice a day for 7 days.

Reference 3 - 3.02% Coverage

It depends, it they’re having recurrent UTIs then I’d get a culture and it would tell you which antibiotic is better for that strain of bacteria, but I’d usually go with bactrim or cipro or possibly macrobid depending on drug allergies.

<Files\\8_13_18_001> - § 1 reference coded [1.53% Coverage]

Reference 1 - 1.53% Coverage

if somebody calls in or sends a message through the portal and they have frequent UTIs or I'm pretty sure they have one and we don't have openings that they can get in then I something send out antibiotics.

**IMPACT OF GUIDELINES AND TRAINING EFFECT ON UTI MANAGEMENT**

Dissemination of guidelines

<Files\\01_08_19_013> - § 1 reference coded [3.73% Coverage]

Reference 1 - 3.73% Coverage

What about educational materials like handouts or posters for providers?

013: Depends on how you do it. An isolated thing is not gonna work that’s a 1. If it’s part of a campaign, I’d give it a 3.

M: Makes sense. Next is educational materials for patients.

013: Well that’s just don’t ask for an antibiotic in the first place. The trouble is doctors do get mad when patients come in and ask for a certain antibiotic.

M: Unless the materials have the right antibiotic, then maybe useful.

<Files\\01_10_19_014> - § 4 references coded [18.09% Coverage]

Reference 1 - 7.15% Coverage

Do you use any specific resources or guidelines when picking antibiotics and treatment duration?

014: Because it’s easy on epic to get to up date, i look at what they’ve got. I used to use the Sanford guide, but my exposure to lots of different stuff is not there. I’m not an infectious disease person, I don’t have all those bacterias like you guys do. So havent used Stanford too much. It just didn’t seem necessary. I will use up to date, that’s the one I really go with.

J: Have you ever used antibiograms to study local resistance in your area?

014: No I haven’t.

J: Do you think that would be a good resource if that was made easily available?

014: I do, when you read up to date, we have a lot of strep throat, they always say try this, but if you have local resistance. I do see a fair amount of STDs, and I don’t know the local resistance. I think that would be wonderful.

References 2-3 - 5.35% Coverage

If clinical guidelines existed on how to treat uncomplicated UTIs, how do you think they should be shared with primary care providers? Publication, mass email, how do you think it should reach practitioners?

014: I think what we probably, email is good because I find that if we get something like that, we just got something on flu in our email. I went into some other offices, everyone has printed it out and hung on bulletin board so oyu can remind yourself. I think some of those common issues, to have protocols on email would be good. I don’t know if I always have the opportunity to seek it out at the time, but if it had already been handed to me I’d use it.

Reference 4 - 5.59% Coverage

How about educational materials for providers/

014: Again I think, Can I give it a 4? I think it could work as long as there weren’t too many of them. We can’t send everything out on everything. On a few key areas it would work, but people get overloaded and they don’t always read everything.

J: How about educational materials for patients?

014: Um, I would like more education for patients. I can’t really speak to whether they really look at those, but I feel like choosing wisely, where they address specific questions. If that was more accessible to patients, like why they’re waiting, something like that might help. If you expected them to take a paper home, that seems like minimal.

<Files\\02_07_19_015> - § 8 references coded [13.12% Coverage]

Reference 1 - 1.22% Coverage

I think they should. It would help a lot because a lot of times when the patient comes to you ___ 13:25 and unfortunately before the trend of the antibiotics… to a multi resistant antibiotic resistant infection, we just gave the antibiotic because the patient wanted it and it has been very difficult now to show the patient that you don't have to get antibiotics every time that you want it.

References 2-3 - 1.87% Coverage

For Sure. Do you think there is anything that the ID societies or the U.S. Preventative Task Force could be doing to help providers like yourself educate their patients? Do you think there is a way that they could be doing more?

015: Yes. Definitely.

M: What sorts of things would be helpful for you?

015: Divide the patients in classes like the… explain the first place that there is a very low chances of having a significant UTI and comorbidity, age, and give us an idea. It may be too specific, but I think it is worth it because UTI is such very common condition. Especially in nursing homes.

References 4-6 - 2.19% Coverage

So, say all of the bodies at B were to come up with a consensus. Say that they made their minds and they had a consensus on what should be treated and what should not be treated. How do you think they should get that information to primary care doctors?

015: Well, it can be done through the magazines that we read.

M: Ok.

015: It could be done through. Like in ____ 17:39 we have a system that we check communication with the administration. The doctors…. The specialties, give their opinions. There are certain conditions in the hospital, when you do certain stuff, it comes from somewhere so then we read and understand them. So, we get educated that way. _____18:02 that’s the way that can be done.

Reference 7 - 2.41% Coverage

If with the test we did, was the culture done? Did we follow through? Did we talk to the patient?

M: Sure.

015: The patient….. I don’t know that they read every single one but somehow the computer keeps a backdraft.

M: Okay.

015: and we have to do something about it. We have to talk to the patient. If not ______20:27 so that’s the way that they do it, but when we cover the protocols for treatment and I’m not getting ___20:36 they come to us from… there is a doctor that goes from… he’s , from Farmington to Saint Louis and speaks in a group of doctors and they discuss treatment and then they publish them and they bring them to Farmington. So we get that kind of information. That there were medications that were removed, there were protocols that were removed.

Reference 8 - 5.43% Coverage

What about educational materials like handouts or posters that could be given to providers, say, with updated guidelines on what was recommended. Do you think that would be helpful?

015: Start at three, because we don’t read that many of them. Every day compiled with papers. Then we just don’t read the whole thing. You would have to be very, very…… calling my attention.

M: Okay.

015: If this is something that I…. that is urgent, that is important than you will call my attention. If it doesn’t call my attention it’s not going to help.

M: Okay. What about ……

015: If it can be mailed… a person that I don’t know. Sometime you get e-mails… I don’t know what that is.

M: For sure.

015: I don’t even read it.

M: What about educational…. What about educational materials for patients, like handouts, or posters.

015: That we can have in the office, Yeah. That can be given to the patient without us knowing. No. So five. Five, if you… if we have it. It will be zero if the patient gets it and we don’t know.

M: Okay. So you think it would be, you know…. It’s kind of up to the providers discretion to give them the For materials or not?

015: Well, I think the providers should read it and explain to patients. I misunderstood your question I think.

M: Okay.

015: I think you meant, if this educational material could be given to the patients, they cannot get that on television.

M: Okay. Gotcha.

015: Or by mail. I suppose that’s what I mean. If the patient gets it that’s a no.

M: Okay.

015: I suppose if that information comes to me. I read it, filter, and give it to the patient.

M: Okay.

015: I don’t like when the patient come to me and say, “There is something that treat the cancer, can you please refer me to that”.

<Files\\08_15_18_002> - § 4 references coded [9.54% Coverage]

Reference 1 - 1.83% Coverage

educational materials for providers like posters or handout?

002-- we got a lot at my primary care and it just didn't I mean you look at it but then everything else is being thrown at you and you don't want to see it a lot so it's probably two.

M--Yeah what about education materials for patients?

002--Not about specific antibiotics, if about antibiotics I would give negative number if there so one

References 2-3 - 7.00% Coverage

So if someone would go in the offices and be like really non-intrusive and say these are the recommendations the new qualifications and recommendations for treating UTIs here just take this with you you look at it at your convenience that was have bottled water you know just something like that it was so relaxed and so casual and you got to see him so often that you walk in to get bread hey how's it going what's going on with cologuard so he was his low-key approach was the most effective way to get us to do almost anything I can remember in the 5 years that I was at BJC at the new building, it was amazing, so it was it was written printed information it was the protocols of the guidelines and recommendations -here take this with you ,hey do you want to see remember what it's about ,do you want to take another one with you, that might take a couple tries again but it was so low key and so non pressure and that was the best way. He went to several offices and did this but it was almost everybody in the office was ordering cologuard within a month or less. So getting out the information through dinners…there were a lot of people aren't going since they cant bring their spouses and yeah that that's hard to do something after hard work the whole day so I wouldn't do the dinners. Have in the handouts with the information given to them but yeah anything to call someone out or compare them is really more negative reinforcement I don't think that I mean yes it will probably change but I'm certainly going to be a positive change.

Reference 4 - 0.71% Coverage

Giving or sending a positive reinforcement will work better than strict or stern actions like dogs like to be petted and given treats than being spanked.

<Files\\08_15_18_003> - § 4 references coded [15.40% Coverage]

Reference 1 - 3.53% Coverage

Yes because it is a common condition and no one often tries to pull it up to see if there's anything out there just, speaking of that if there were some guidelines out there in terms of what the first-line antibiotic should be or other things like that, how do you think they should be shared with primary care providers?

And how should they be shared……. using the medical literature sufficient or what do you think?

003--That is a good question……… for a lot of things…it should be mandated and should have the freedom to practice individually. Should we have protocols…like should we culture every urine? That’s kind of standard protocol. Should we use this, this and that as first line? Then you would think each

medical group or practice may want to initiate some guidelines and where is that gonna come from…

Your license is on the line kind of thing……

Reference 2 - 3.26% Coverage

If there were some standards set do you think the provider should be evaluated by their adherence to you know if a clinic or somewhere else made a set of internal guidelines or other recommendations?

003-- like other things at like I really kind of like super expensive like CHF you should be having the ACE blocker etc etc cuz we don't want that 30-day readmission yeah it's very expensive and UTI being way way down on the list of high costs, so probably not a priority to compare in comparison to the really expensive type problems. I don’t know the answer, maybe try to create some ideas may be…specialists in the field coming up with practices or suggesting perhaps we should do this and having maybe a conference to talk about this and deciding on this and this might be a best practice

Reference 3 - 3.53% Coverage

M-- what about educational materials for providers like posters and things?

003—More than 4. The initiative to reduce antibiotic use for colds and coughs and I think there was there was a push here recently and I think it did start reducing, I may be wrong but there was an article about that about how we can influence physicians, ourselves and our peers to do that. I think that worked….

M—there were mixed studies, I think some showed it worked they put it into a bundle with bunch of other things I think a lot of its related implementation right so if they if they just put the handouts on the desk and then don't tell anybody about it then I think that would not work

M—What about education materials for patients. Would that work at all or not?

003--Yeah yeah I think it is effective as far as rating I don't know maybe that 3.5 kind of thing.

Reference 4 - 5.07% Coverage

some of the interventions that we talked about would be considered intrusive to for people that are you done Primary Care to think there's anything that kind of strikes the right balance between being helpful for providers and you know being beneficial for optimizing antibiotic prescribing practices?

003—I was trying to think of like the deposition who's like set in his ways and I don't want anybody tell me what to do,done this this for years I know what I'm doing and also those more willing to accept, change or discover that hey I'm not doing it correctly or where is that right balance… but if I know the answer. Also where the money comes from …the insurance companies may say hey doctor…ive seen such reports average person is maintaining blood pressure and diabetes and once in a while prescribing an antibiotic. What's the ad maybe something from the insurance companies saying these are the results between peers and I have seen those and kind of make me think for a moment yeah. Maybe something coming from authority like CDC. I mean if you know someone personally or have respect for someone then a conference or a small group meeting with the practice saying hey lets look at it this way may be more influencial.

<Files\\08_15_18_004> - § 5 references coded [9.33% Coverage]

References 1-2 - 3.10% Coverage

there were recommendations out there how should they be disseminated or shared with primary care providers?

004--That's a really good question cuz I think we're just inundated with all sorts of stuff, most of which we've learned to ignore. Guidelines to help , we have to pay attention the guidelines. And keep up to date with journal watch or another publications but I don't know how else. I would think it's hard to change for all docs to learn new tricks for one thing.

Reference 3 - 2.52% Coverage

What about education materials for providers like handouts or posters.

004--That's probably a one or two…Probably not going to get a four or five out of me.

M--Yeah I know this is this is this is why I want to hear so…

M-- what about educational materials for patients?

004—I will give that a 2 or 3.Depends on how widely disseminated it is but patients pay attention to that stuff.

References 4-5 - 3.70% Coverage

presentations in the form of educational forums….So we have had that you BJC medical group meetings twice a year that everybody comes to and we've had pretty good educational presentations along these lines. Especially if it is a game changer, asking us to do something we haven’t been doing and evidence based presentations. That I find helpful and useful and I pay attention to it. But I do know something written or something which actually invades our space in terms of work done at the end of the day.We are good at deflecting that otherwise we couldn't work.

<Files\\08_23_18_005> - § 4 references coded [7.86% Coverage]

References 1-2 - 5.03% Coverage

M: Got it. Okay. Great. So, if there were clinical guidelines on how to treat uncomplicated urinary tract infections, how do you think they should be shared with people, like in primary care and convenient care settings?

005: I do better with notebooks as far as a paper reference, but I mean something like up to date would be good, like a computer database. But the computer database, like they actually have to have tables within the articles, so there’s evidence based practice, there’s information that they put out, but then within there it was male UTIs, female UTIs, and had very strict easy to read tables, which was nice as far as you could just flip and reference and go if you had read it before.

M: Got it. But what about if it’s just published alone and it’s not really put anywhere else is that good enough? Is it expected that the provider would go read the primary journal, or it sounds like you prefer kind of a reference article or...

005: Yeah, something kinda straight to the point like, “hey we want you to treat UTIs in this way and a table.” I’m just used to all the other information with it, but a table would be sufficient, like you should use this. Evidence based practice and the research behind it is nice to read once, and then just reference the table after that would be nice.

Reference 3 - 1.44% Coverage

What about education materials for providers, like handouts orposters? 005: 4 or 5.

M: Okay, what about education materials for patients?

005: Man, I feel like patients don’t read it. Maybe a 3? It just depends on the person. I feel like I give a lot of people handouts and they just look at me. It’s a lot of information. It depends on how the handout would be formatted.

Reference 4 - 1.39% Coverage

So which interventions do you think would do the best at improving patient safety? Sounds like…

005: Algorithms, and I mean education in general. I just think a lot of the time there’s not the education and after you’ve been in practice for a while you’re not seeing well this antibiotic works better, it's just the lack of education might not be as accessible.

<Files\\08_23_18_005 (2)> - § 3 references coded [7.85% Coverage]

Reference 1 - 5.02% Coverage

Got it. Okay. Great. So, if there were clinical guidelines on how to treat uncomplicated urinary tract infections, how do you think they should be shared with people, like in primary care and convenient care settings?

005: I do better with notebooks as far as a paper reference, but I mean something like up to date would be good, like a computer database. But the computer database, like they actually have to have tables within the articles, so there’s evidence based practice, there’s information that they put out, but then within there it was male UTIs, female UTIs, and had very strict easy to read tables, which was nice as far as you could just flip and reference and go if you had read it before.

M: Got it. But what about if it’s just published alone and it’s not really put anywhere else is that good enough? Is it expected that the provider would go read the primary journal, or it sounds like you prefer kind of a reference article or...

005: Yeah, something kinda straight to the point like, “hey we want you to treat UTIs in this way and a table.” I’m just used to all the other information with it, but a table would be sufficient, like you should use this. Evidence based practice and the research behind it is nice to read once, and then just reference the table after that would be nice.

Reference 2 - 1.44% Coverage

What about education materials for providers, like handouts orposters? 005: 4 or 5.

M: Okay, what about education materials for patients?

005: Man, I feel like patients don’t read it. Maybe a 3? It just depends on the person. I feel like I give a lot of people handouts and they just look at me. It’s a lot of information. It depends on how the handout would be formatted.

Reference 3 - 1.39% Coverage

So which interventions do you think would do the best at improving patient safety? Sounds like…

005: Algorithms, and I mean education in general. I just think a lot of the time there’s not the education and after you’ve been in practice for a while you’re not seeing well this antibiotic works better, it's just the lack of education might not be as accessible.

<Files\\08_23_18_006> - § 3 references coded [3.63% Coverage]

References 1-2 - 2.83% Coverage

A: What are your thoughts about clinical practice guidelines, do they play a role in this, how should they be shared with providers if they exist?
B: They’re important and they do give you a basic framework. It’s always a good starting point, and then you can take other factors for the patient condition into consideration. How to disperse them, I don’t know, but I do think they should be readily and easily available, regardless of whether or not the internet wants to cooperate.

Reference 3 - 0.79% Coverage

C: What about educational materials like posters, handouts, for providers?
A: A 4
A: What about educational materials for patients?
B: 3

<Files\\09_05_18_007> - § 2 references coded [2.37% Coverage]

Reference 1 - 0.85% Coverage

C: What about educational materials for providers, like handouts and posters?
D: Yeah, 3 to 4.
A: What about educational materials for patients, like handouts and posters?
B: 5

A:What about educational materials for patients?

B: 5, I think that’s good.

Reference 2 - 1.51% Coverage

A: So, in my opinion with a very very clear algorithm for providers, that they can just review from time to time. The difference between complicated, uncomplicated, multi drug resistant or simple e coli infection. The algorithm may help to look at that and say this is the right way to do things. A simple way is to just have a picture, I think that’s simpler than sending emails and saying this physicial is giving better care. I think thats a simple way.

<Files\\10_10_18_008> - § 1 reference coded [7.03% Coverage]

Reference 1 - 7.03% Coverage

Oh no. I don't mind this at all, that's why I'm doing these interviews. I'm learning how people make these decisions. No, this is great. If clinical guidelines existed on how to treat urinary tract infections, how do you think they should be shared with primary care doctors?

8: Meaning, like up to date or email or a website?

M: Yeah exactly. Do you think that publication in a journal sufficient or do you think that they should be better disseminated, like via up to date. Or, some people use the choosing wisely website and other things too. Although I don't know how many physicians actually look at that.

8: I would say certainly I'm not sure that a journal is enough, unfortunately. So I would say that either choosing wisely, or certainly up-to-date to have some sort of guidelines. I think up to date would honestly be the, unfortunately, most reviewed maybe. But I do think that a journal is probably not enough and some sort of up-to-date or another website would be necessary.

<Files\\10_10_18_008 (2)> - § 3 references coded [13.45% Coverage]

Reference 1 - 6.05% Coverage

If clinical guidelines existed on how to treat urinary tract infections, how do you think they should be shared with primary care doctors?

8: Meaning, like up to date or email or a website?

M: Yeah exactly. Do you think that publication in a journal sufficient or do you think that they should be better disseminated, like via up to date. Or, some people use the choosing wisely website and other things too. Although I don't know how many physicians actually look at that.

8: I would say certainly I'm not sure that a journal is enough, unfortunately. So I would say that either choosing wisely, or certainly up-to-date to have some sort of guidelines. I think up to date would honestly be the, unfortunately, most reviewed maybe. But I do think that a journal is probably not enough and some sort of up-to-date or another website would be necessary.

Reference 2 - 3.81% Coverage

What other things did you use? Sometimes there's emails that come out on news articles about infectious disease things. I think I get the journal watch periodically emails. Do you get those?

8: Oh I get journal watch every day actually. And I do think that those are kind of helpful. And I actually read them everyday. I'm not exactly sure how great of a resource that would be. Like remember long-term. Whether it was on up-to-date, I know it would be there and I would just go and look there. I think that would be the easiest thing.

Reference 3 - 3.60% Coverage

Okay. What about educational materials for providers like handouts or posters?

8: Unfortunately, I think that may be less helpful only because they are just so busy and it's just so hard to. It's information overload and mail overload and I think it would just get pitched potentially.

M: So what number would you give it?

8: I'm sorry, um, 2.

M: All right. What about educational materials for patients like handouts and posters?

8: I'm not sure that would be a very helpful there let's go, 2.

<Files\\11_14_18_009> - § 6 references coded [12.85% Coverage]

References 1-2 - 4.85% Coverage

: If there were guidelines on how to treat uncomplicated UTIs, how do you think they should be shared with frontline providers?

009: I mean, I think if there’s a nice algorithm, that could be made that shows the flow to how things are preferred to be done, I think that would make it easy to follow. Something with a lot of reading, or that’s really complicated, I don’t think that’s gonna be practical on a daily basis.

M: Do you think that publication in a journal is sufficient?

009: I think if you get it in a journal that’s a great thing, but definitely in that journal include something where it can kind of visually look and understand what you need to do. That way it becomes routine for the staff, becomes standard throughout the clinic so it’s very easy to execute.

References 3-4 - 3.20% Coverage

M: If you’re gonna spread them out throughout the clinic, who should be responsible for disseminating them in the clinic?

009: Well it depends on how management is set up. Different systems in different clinics are set up differently. I think this is a good topic that leaders can take that information, share it with doctors, and have it kinda start there and trickle down to staff and management. Make it a lcinic pliciy to implement it on that side of things, come up with and managed by the physician leaders.

Reference 5 - 3.62% Coverage

I think this is a nice, simple, algorithm, that would be great. If it’s just gonna be a 5 page article explaining everything, primary care doctors are so busy, it’s hard to make time during the day to do something that is gonna take a lot of your time. A lot of family medicine doctors that have been practicing medicine for a long time, they kinda do things their way and it can be kinda difficult to bring change, you know? So making it simple and flexible is the best way to have adherence to whatever you’re trying to accomplish?

M: Kind of a lower number then?

009: Yeah, 3.

Reference 6 - 1.18% Coverage

i think any type of educational material for patient is good, because there’s a lot of misinformation out there. It needs to be short, to the point, easy to understand. Let’s give that a 4.

<Files\\12_18_18_010> - § 6 references coded [20.60% Coverage]

References 1-2 - 6.30% Coverage

J: Okay. So this is like another question about guidelines. If clinical guidelines existed on how to treat uncomplicated UTIs, how should they be shared with primary care providers? What is most effective way to share those guidelines?

010: Well I would hope that clinicians would subscribe to something like I do, like prescriber's letter where you get monthly clinical information, or if you wanted to get to an entire medical system you could do it from the top down. You could have medical directors send out a little FYI. I think that having good resources with good research is important, so reading up each month on new information, trying to stay current. I don’t know that people do paper journals anymore, I think electronic media.

Reference 3 - 5.27% Coverage

Who do you think should be responsible for disseminating these guidelines?

010: I kinda think it’s incumbent on providers to stay up to date, but in a giant medical system, that’s kinda tough. Wow, I didn’t think about that. Medical directors are usually concerned with running the organization.

J: So you think clinical leadership should have more of a role in disseminating them?

010: I think so. Wouldn’t it be nice if there was a clinical director in a giant medical group whose sole role was to make sure everybody gets the most up to date tidbits every month? Sometimes that’s tough. Wouldn’t that be cool?

Reference 4 - 5.65% Coverage

: Okay, how about educational materials for providers like handouts and posters?

010: Yes, that would be good. Education, like for patients?

J: For providers.

010: Yeah, I think some educational handouts would be good.

J: So on a scale of 1-5?

010: Maybe a 3.

J: Educational materials for patients?

010: That would be very good, I’d put that way up at the top.E Everybody marches in and expects antibiotics at the drop of a hat. Sometimes if I can’t convince somebody to not take an antibiotic, I’ll handwrite a script and say do not take this until we have the information back. So sometimes I can convince them that way. I think that’d be helpful.

References 5-6 - 3.38% Coverage

: Trying to think...No, I think I can’t think of anything. Maybe just doing presentation to entire group at one of those meetings we have a couple times a year. That might be helpful, hey these are the new guidelines, just want to let you know, this is the data we’re seeing on people coming in for UTIs, unintended consequences, etc etc, Maybe a 10 or 15 minute update would be very nonthreatening.

<Files\\12_19_18_011> - § 4 references coded [13.34% Coverage]

References 1-2 - 6.38% Coverage

If clinical guidelines existed on how to treat uncomplicated UTIs, how should they be shared with primary care providers?

011: Initially, it definitely think email is great. In the mornings when i get here i look at all the emails that come in, whether it be weekly or monthly that would be a good thing to be brought up. I’d say that would be the best way. I know people do meetings and things like that, but everybody gets annoyed at those. Probably email would be a great way, I know they do those newsletters, I don’t remember whether weekly or monthly, maybe a section in there that's something about guideline updates and things that are like that would be helpful.

Reference 3 - 3.18% Coverage

What do you think should be the role of clinic leadership?

011: They do send out those newsletters, so if leadership wanted to create that little section about guidelines, maybe somebody. Maybe it would be easier for researchers to take those guidelines to leadership and then leadership could disseminate that information to the PCPs

Reference 4 - 3.79% Coverage

How about educational materials for providers like handouts or posters?

011: Probably a 2.

J: Why do you think it doesn't’ work?

011: People don’t take the time to read those.

J: What about educational material for patients?

011: I think that would be beneficial like a 4.

J: Does your patient population ask a lot of questions about treatment?

011: It’s about 50/50 right now.

<Files\\12_20_18_012> - § 7 references coded [21.83% Coverage]

References 1-3 - 3.36% Coverage

If certain clinical guidelines existed on how to treat uncomplicated UTIs, how should they be shared with primary care providers?

012: I guess with continuing education classes.

J: Do you think publications or newsletters would be helpful?

012: I think so

Reference 4 - 10.38% Coverage

Who do you think should be responsible for disseminating these guidelines? Like the people who wrote it, or clinic leadership? Who does the responsibility lie with?

012: I honestly don’t know. Like other guidelines, like new blood pressure or cholesterol, a lot of times it’s drug reps that mention it, I don’t know whose job it is to get the information out.

J: Do you think that providers should be responsible because they’re the ones that are prescribing them?

012: Yes, we are responsible to be knowledgeable on the latest guidelines, but at the same time there’s so many guidelines out there for so many different things it’s almost impossible to keep up with. And different places, they don’t always agree on what is best practice. You have one set from this organization, then another, they might conflict.

Reference 5 - 2.40% Coverage

Educational material for providers like handouts or posters about most recent guidelines.

012: 5, that would be really nice.

J: How about educational materials for patients?

012: 3

Reference 6 - 3.65% Coverage

If you were designing a study to improve adherents to best practice guidelines, which intervention or interventions would strike the best balance between improving quality of care and being helpful for providers?

012: Like what they talk about with posters and handouts, I like that one

Reference 7 - 2.04% Coverage

Can you think of anything I didn’t list?

012: Like continuing education hours.

J: You think that would be most helpful.

012: I think that would help.

<Files\\8_13_18_001> - § 4 references coded [12.52% Coverage]

References 1-2 - 6.17% Coverage

and then if there were clinical practice guidelines about how to treat UTIs how do you think they should be shared and do u think providers should be evaluated by adherence to them or anything like that. You guys are already evaluated by so many metrics it’s crazy..

001—I would be okay to be evaluated by that than if my patient got a diabetic eye exam or overweight id rather be. You know everyone is so so busy will be be hard to say everyone to attend a lecture about UTI but it would not be nice if you could send it out in an email email for me to know these are the new practice guidelines for this and then as long as I think as long as people knew that there was going to be a metric and they were going to be evaluated or you know you had a certain amount of people that wanted to participate and I think that's fine.

Reference 3 - 2.18% Coverage

Educational material?

001--I think if it was in an email it would probably be a four, if it was paper you know how that…

M-- and what about education materials for patients?

001-- that might be good to have like pamphlets in the room that they could grab and read about or something…three

Reference 4 - 4.17% Coverage

--It's hard to say and the reason I said is everybody you know we have a big practice here and I've known those people and everybody is so different so some people are really into you know the following these guidelines and these metrics and there's some people that are like I'm going to do things my way and I don't care and so its difficult to say what is the best way but I do think if there were emails educational you know opportunities and as a practice you could say you know we're going to try and set up these guidelines to follow, its just difficult.

Guidelines role in selection

<Files\\01_08_19_013> - § 1 reference coded [5.07% Coverage]

Reference 1 - 5.07% Coverage

Yeah I can picture some of those! What specific resources or guidelines do you use when picking antibiotics or treatment duration?

013: Well heck I wrote some of them for long term care, so I’m biased there. Yeah, your criteria, what you were trying to get everyone to use in our long term care world, infectious disease society guidelines, are sort of go to. Try to go to national society guidelines.

M: You’re one of the first people I’ve talked to that goes straight to the guidelines, rather than up to date. Most of the providers I chat with go to up to date.

013: I just haven’t had much need. Guidelines don’t change much with UTIs, so I haven't’ check it much.

<Files\\08_15_18_003> - § 3 references coded [5.00% Coverage]

Reference 1 - 1.37% Coverage

Yeah I think the medical letter, reading in journals, hearsay like some urologists are saying don’t use this or that for resistance, haven’t done a lot of Cochrane reviews or other research but it may be the medical letters pretty non biased so I like to use that as a resource and maybe just some of the journals kind of sway me …

Reference 2 - 1.75% Coverage

So as far as that on the quinolones the only thing I was noticing too maybe I'll ask you later when we're done is that the lower dose of the quinolones were failing, like the 250s for minor UTI that is 250 BID for five days and many came back with recurrence and the same with Levaquin 250. One of the Levaquin reps told us we don’t want to talk about levoquin 250 anymore, so maybe those are some influences over the years.

Reference 3 - 1.88% Coverage

M-- got it and then.It sounds like you use kind of several resources in terms of helping to select antibiotics for you mentioned that FDA Black Box warning you mentioned the medical letter aafp and it sounds like jama too, any other ones or is that mostly it?

003---I use uptodate. Use information from uptodate so that’s another resource and like I said Cochrane review I haven't used it as a resource for UTI as I haven’t really investigated around it.

<Files\\08_23_18_005> - § 1 reference coded [5.02% Coverage]

Reference 1 - 5.02% Coverage

Got it. And then, you mentioned some resources that you use when picking antibiotics, your experience with CVS’s algorithm, then your training you mentioned too. Are there others that you use or that you look at periodically?

005: I mean I use epocrates as far as reference, what’s the little book it starts with a...There’s an antibiotic reference guideline.

M: Sanford?

005: Yes, it is Sanford, we have a Sanford guideline on the desk, and they have a current one so we kinda reference that, and then as far as that goes, a lot of patients will come in and say “Oh I get these all the time this one doesn’t work.” And so it’s like okay, that one doesn’t work, and then obviously epoc allows us to see culture reports, so i can go back in and make sure if

there’s no resistance then I start basically and then if there is resistance that kinda changes where my mindset goes bc that will kinda allow me to choose different antibiotics based on past culture reports.

M: Got it. And then, I think other resources too, some people will use up to date, something like that, do you ever use those too?

005: I do have up to date, I’m not an up to date fan, I feel like a lot of those articles are really complicated to get to, so no, generally I don’t use up to date. Have I used it in the past? Yes.

<Files\\08_23_18_006> - § 2 references coded [16.39% Coverage]

Reference 1 - 0.52% Coverage

A: Got it, and where do you look up the cipro one?
B: I either use epocrates or Tarascan.

Reference 2 - 15.87% Coverage

A: Alright, and then in terms of, I think that answered all the questions that we had, and the next one too. Moving along nicely. You mentioned some resources that you look at when selecting antibiotics. Can you tell me how you got into practicing macrobid, where that initially came form?
A: I worked at CVS Medical Clinic prior to here and that was part of the guideline, so that was the only option I had. They had very specific ways that you went through it, so that just became, okay that’s what I do. It usually worked pretty well and people didn’t come back.
A: How did you feel about following this somewhat rigid algorithm? Did you like it, did it bother you?
A: It bother - I don’t mind it, but I like to have the freedom to step outside the box if I need to.
A: Okay, and tell me about stepping outside the box. Gina mentioned a little bit earlier about how that would be harder at CVS before?
B: Correct, you had to be able to document a company accepted reason for not following the guideline or risk, you know, the chart police coming after you. So, you know if you can’t say patient refuses or sometimes you just, think that maybe this might be a better option for this patient, or the patient is just insistent. You don’t always have access to their records, but if someone comes in and says the last time I took macrobid it didn’t work, I’m gonna take your word for that. I’m not gonna argue with you on that point because I wouldn’t want someone to argue with me on that point. So I’ll prescribe you something different. Sometimes they come in with I want this, and then there’s not a lot of room for negotiation, and sometimes it’s, well let’s consider everything you have going on. Sometimes they come in with multiple complaints: Oh I have a UTI, and a URI. Okay, what can i give you that’s gonna take care of both without putting you on multiple antibiotics.
A: Got it, okay, great. And then, in terms of the algorithm that you followed at CVS, you probably don’t have access to it anymore, I think usually they keep them.
A: Correct
A: You just have that algorithm kinda memorized now and you tend to use it as a template?
B: Yes
A: Got it. Okay then, do you ever look at other, beyond the 2 that you mentioned, do you look at other resources like up to date, or do you ever pull up primary literature, or anything like that?
A: If I have time then sometimes I will. Up to date tends to be, the internet doesn’t always cooperate and allow me to use it. It will time out multiple times and then I give up on that. It just depends on time and availability of the resource. Sometimes I’ll ask another provider, alright you’ve been doing this longer than I have, what do you think?

<Files\\09_05_18_007> - § 1 reference coded [2.18% Coverage]

Reference 1 - 2.18% Coverage

B: Honestly, obviously I use up to date a lot, time to time I’ll use my AFP journals, reviews and all that. But I do use up to date for, at least I have 2 or 3 times a day or more, up to date is up so I can look up something. So, most of the guidelines it from ED, and it’s from the TMEs I’ve attended, that kind of stuff, some of the experience of that that I follow. There are times when I see patients getting antibiotics for 7 to 10 days, where routinely they come to me and say by the way urgent care gave me that, I do not think it’s necessary to treat patients for longer duration of antibiotics for simple UTIs, so I kind of educate them about that.

<Files\\10_10_18_008> - § 1 reference coded [3.80% Coverage]

Reference 1 - 3.80% Coverage

: What other things did you use? Sometimes there's emails that come out on news articles about infectious disease things. I think I get the journal watch periodically emails. Do you get those?

8: Oh I get journal watch every day actually. And I do think that those are kind of helpful. And I actually read them everyday. I'm not exactly sure how great of a resource that would be. Like remember long-term. Whether it was on up-to-date, I know it would be there and I would just go and look there. I think that would be the easiest thing.

<Files\\11_14_18_009> - § 1 reference coded [3.36% Coverage]

Reference 1 - 3.36% Coverage

Are there specific resources or guidelines that you use on picking antibiotics?

009: Yeah so I go on Up To Date or FP Notebook are the 2 I use the most.

M: I’m not familiar with FP notebook can you tell me a little bit more about that?

009: Yeah so that’s, basically a source that many family medicine residents use as a quick guide to managing medical conditions. It’s kinda like, you know that green book that we had , pocket Medicine, basically it’s like that online. Up to date is more specific, but this is very good quick look.

<Files\\12_18_18_010> - § 1 reference coded [2.94% Coverage]

Reference 1 - 2.94% Coverage

Yeah. Do you use any specific resources or guidelines when picking antibiotics or treatment durations?

010: I do, sometimes I use hypocrates, or up to date, so I’ll access those. Also I get prescriber's’ letter, the guidelines sometimes shift. I know there’s a one day therapy now they’re recommending as well, so I’ll follow those guidelines.

<Files\\8_13_18_001> - § 1 reference coded [0.60% Coverage]

Reference 1 - 0.60% Coverage

Yes uptodate when it works, the internet dosent work here but when it does yes.

Opinion guidelines not useful

<Files\\01_10_19_014> - § 1 reference coded [2.99% Coverage]

Reference 1 - 2.99% Coverage

: What do you think should be the acceptable adherence rate? How much would be acceptable?

014: You know, I think for that, maybe something like 85%, I know that doesn’t sound high, but I see on other things that I pretty well try to follow the book, but sometimes the book doesn’t work for whatever. To try to give a little clinical leeway, but still make it important.

<Files\\02_07_19_015> - § 2 references coded [4.03% Coverage]

Reference 1 - 1.61% Coverage

For conditions other than urinary tract infections, do you have a go to resource that you use to look up information?

015: Like going to be up to date and stuff like that?

M: Sure. Sure. Do you have a favorite that you use?

015: I like to use up to date, but in cases that I'm very possible concerned because it takes longer to see the patient and then to get the answer. You have to read a lot of stuff and usually not everything applies to the patient. That does not describe exactly my patient.

M: Ok. Gotcha.

Reference 2 - 2.42% Coverage

: I hope you address us with that. The nursing home is a pain in the neck.

M: Yes.

015: And Medicare is coming to nursing homes to review UTI’s

M: Yep.

015: And we are… We have to decrease the treatment of unnecessary UTI’s.

M: For sure.

015: So we have the pressure from all these people so when we ask, “Ok, so what criteria”? Oh, you have to make you have to make your own criteria. Then they start looking into what’s doing and whose doing what. What do the companies say…I went to the text, let’s see…about three or four years ago about what the recommendation was and I printed it and then later on apparently that they changed their mind___16:31 (two voices) for the UTI’s in nursing homes. So, that’s for the record, that sometimes they just change their minds.

<Files\\08_23_18_006> - § 1 reference coded [7.72% Coverage]

Reference 1 - 7.72% Coverage

A: How did you feel about following this somewhat rigid algorithm? Did you like it, did it bother you?
A: It bother - I don’t mind it, but I like to have the freedom to step outside the box if I need to.
A: Okay, and tell me about stepping outside the box. Gina mentioned a little bit earlier about how that would be harder at CVS before?
B: Correct, you had to be able to document a company accepted reason for not following the guideline or risk, you know, the chart police coming after you. So, you know if you can’t say patient refuses or sometimes you just, think that maybe this might be a better option for this patient, or the patient is just insistent. You don’t always have access to their records, but if someone comes in and says the last time I took macrobid it didn’t work, I’m gonna take your word for that. I’m not gonna argue with you on that point because I wouldn’t want someone to argue with me on that point. So I’ll prescribe you something different. Sometimes they come in with I want this, and then there’s not a lot of room for negotiation, and sometimes it’s, well let’s consider everything you have going on. Sometimes they come in with multiple complaints: Oh I have a UTI, and a URI. Okay, what can i give you that’s gonna take care of both without putting you on multiple antibiotics.

Opinion guidelines useful

<Files\\08_23_18_005> - § 2 references coded [5.43% Coverage]

Reference 1 - 3.41% Coverage

Got it. So it sounds like pharmacy calls and then your historical practices and training with

CVS about following that algorithm kinda..

005: 100% they gave me an algorithm, and I think that’s why I kinda use, or where my thought process goes.

M: What are your thoughts about the algorithm? When you were at CVS did you like it, or did you not like it?

005: I liked it, I mean I like to know if this happens here’s this, here’s that, what antibiotic do we not use. I mean I was just kinda trained and programmed that way. But I enjoyed the algorithm, and I still use it in current practice today. I’m sure, I don’t think it's changed that much since then, I keep up, I do some reading and research just to make sure we’re still there. But I liked it.

M: Do you still keep a copy of it with you?

005: It’s very possible I might still have a copy or something like that.

Reference 2 - 2.02% Coverage

Yeah, of not being too intrusive so that you’re not making people upset about changing their behavior, but still changing it to the degree where they might be prescribing antibiotics for the right treatment duration or…

005: I mean, patient care comes first. So safety of the patient comes first. If it’s evidence based practice proven that it works, if it’s studied, upsetting the provider is the least of my worries. I think it needs to be geared toward patient satisfaction, patient safety, more the patient side of things.

<Files\\08_23_18_005 (2)> - § 2 references coded [5.43% Coverage]

Reference 1 - 3.41% Coverage

Got it. So it sounds like pharmacy calls and then your historical practices and training with

CVS about following that algorithm kinda..

005: 100% they gave me an algorithm, and I think that’s why I kinda use, or where my thought process goes.

M: What are your thoughts about the algorithm? When you were at CVS did you like it, or did you not like it?

005: I liked it, I mean I like to know if this happens here’s this, here’s that, what antibiotic do we not use. I mean I was just kinda trained and programmed that way. But I enjoyed the algorithm, and I still use it in current practice today. I’m sure, I don’t think it's changed that much since then, I keep up, I do some reading and research just to make sure we’re still there. But I liked it.

M: Do you still keep a copy of it with you?

005: It’s very possible I might still have a copy or something like that.

Reference 2 - 2.02% Coverage

Yeah, of not being too intrusive so that you’re not making people upset about changing their behavior, but still changing it to the degree where they might be prescribing antibiotics for the right treatment duration or…

005: I mean, patient care comes first. So safety of the patient comes first. If it’s evidence based practice proven that it works, if it’s studied, upsetting the provider is the least of my worries. I think it needs to be geared toward patient satisfaction, patient safety, more the patient side of things.

<Files\\08_23_18_006> - § 2 references coded [9.60% Coverage]

Reference 1 - 7.72% Coverage

A: How did you feel about following this somewhat rigid algorithm? Did you like it, did it bother you?
A: It bother - I don’t mind it, but I like to have the freedom to step outside the box if I need to.
A: Okay, and tell me about stepping outside the box. Gina mentioned a little bit earlier about how that would be harder at CVS before?
B: Correct, you had to be able to document a company accepted reason for not following the guideline or risk, you know, the chart police coming after you. So, you know if you can’t say patient refuses or sometimes you just, think that maybe this might be a better option for this patient, or the patient is just insistent. You don’t always have access to their records, but if someone comes in and says the last time I took macrobid it didn’t work, I’m gonna take your word for that. I’m not gonna argue with you on that point because I wouldn’t want someone to argue with me on that point. So I’ll prescribe you something different. Sometimes they come in with I want this, and then there’s not a lot of room for negotiation, and sometimes it’s, well let’s consider everything you have going on. Sometimes they come in with multiple complaints: Oh I have a UTI, and a URI. Okay, what can i give you that’s gonna take care of both without putting you on multiple antibiotics.

Reference 2 - 1.88% Coverage

C: What are your thoughts about clinical practice guidelines, do they play a role in this, how should they be shared with providers if they exist?
D: They’re important and they do give you a basic framework. It’s always a good starting point, and then you can take other factors for the patient condition into consideration

Provider resources

<Files\\01_08_19_013> - § 5 references coded [14.04% Coverage]

References 1-2 - 5.07% Coverage

Yeah I can picture some of those! What specific resources or guidelines do you use when picking antibiotics or treatment duration?

013: Well heck I wrote some of them for long term care, so I’m biased there. Yeah, your criteria, what you were trying to get everyone to use in our long term care world, infectious disease society guidelines, are sort of go to. Try to go to national society guidelines.

M: You’re one of the first people I’ve talked to that goes straight to the guidelines, rather than up to date. Most of the providers I chat with go to up to date.

013: I just haven’t had much need. Guidelines don’t change much with UTIs, so I haven't’ check it much.

References 3-5 - 8.96% Coverage

Oh one other question I forgot. In hospital setting we use antibiograms, in clinic settings I never see that being used. One of the thoughts I’m kicking around, and I’d like your thoughts on it, do you think antibiograms should be incorporated in outpatient settings, do you think it’s appropriate? Some of the guidelines use cutoff points for when a firstline antibiotic should be used based on local susceptibilities. Is that info ever sent to you, are you aware of local susceptibilities?

013: If i were just office setting we would have some trouble some trouble. I think it’s more about resistant infections, I don’t how much an antibiogram would change my opinion about what abx I’m using for a UTI, because you can always get a culture. For a respiratory infection, that’s a little different.

M: That’s true, it might help with empiric antibiotic selection before culture are back if u think they need it.

013: I know there’s a lot of resistant to sulfa and ampicillin basically because that’s what I should be using to start with.

M: That will be the goal. None of the outpatient providers I talked to are aware of antibiograms. I am not sure that should be shared or not.

<Files\\01_10_19_014> - § 5 references coded [21.18% Coverage]

References 1-2 - 7.21% Coverage

Okay. Do you use any specific resources or guidelines when picking antibiotics and treatment duration?

014: Because it’s easy on epic to get to up date, i look at what they’ve got. I used to use the Sanford guide, but my exposure to lots of different stuff is not there. I’m not an infectious disease person, I don’t have all those bacterias like you guys do. So havent used Stanford too much. It just didn’t seem necessary. I will use up to date, that’s the one I really go with.

J: Have you ever used antibiograms to study local resistance in your area?

014: No I haven’t.

J: Do you think that would be a good resource if that was made easily available?

014: I do, when you read up to date, we have a lot of strep throat, they always say try this, but if you have local resistance. I do see a fair amount of STDs, and I don’t know the local resistance. I think that would be wonderful.

Reference 3 - 5.39% Coverage

Who should be responsible for disseminating guidelines?

014: Well, you, of course! Not me. Because I’m in the medical group, it seems like there must be somebody in the group who could be charged with doing that, even if the information came from an outside source like infectious disease. I know we have nurses, it seems like a lot of things come out with Dr. Pog’s name on them. I think the medical group should do the sending. I don’t know if they have anybody in place that collects that information. I think a nurse would be fine with that, as long as they were given the right resources. Talk to the health department or whoever collects that epidemiology kinda thing.

Reference 4 - 2.99% Coverage

: What do you think should be the acceptable adherence rate? How much would be acceptable?

014: You know, I think for that, maybe something like 85%, I know that doesn’t sound high, but I see on other things that I pretty well try to follow the book, but sometimes the book doesn’t work for whatever. To try to give a little clinical leeway, but still make it important.

Reference 5 - 5.59% Coverage

How about educational materials for providers/

014: Again I think, Can I give it a 4? I think it could work as long as there weren’t too many of them. We can’t send everything out on everything. On a few key areas it would work, but people get overloaded and they don’t always read everything.

J: How about educational materials for patients?

014: Um, I would like more education for patients. I can’t really speak to whether they really look at those, but I feel like choosing wisely, where they address specific questions. If that was more accessible to patients, like why they’re waiting, something like that might help. If you expected them to take a paper home, that seems like minimal.

<Files\\02_07_19_015> - § 12 references coded [12.83% Coverage]

Reference 1 - 1.12% Coverage

For sure. For sure. Do you use any specific resources or guidelines when you are picking your antibiotics and treatment duration? Any reference materials that you use.

015: You know, I don't know. I have not read. I cannot talk to anybody. It's just what I have done over time. You know, I have not really read the guidelines, to be honest.

M: Ok. For sure.

References 2-3 - 1.69% Coverage

Ok. For sure. For sure. For conditions other than urinary tract infections, do you have a go to resource that you use to look up information?

015: Like going to be up to date and stuff like that?

M: Sure. Sure. Do you have a favorite that you use?

015: I like to use up to date, but in cases that I'm very possible concerned because it takes longer to see the patient and then to get the answer. You have to read a lot of stuff and usually not everything applies to the patient. That does not describe exactly my patient.

M: Ok. Gotcha.

Reference 4 - 1.06% Coverage

it’s based on my experience. I have been doing this for thirty years and I now remember when I came to work with this clinic and the old doctors would say, "My experience the last thirty years." and I would say, "Why don't you read the literature"? After thirty years, believe me, you would say, "I don't want to read anymore, I'm too tired".

References 5-9 - 3.34% Coverage

: All of that because we have to bring dramatic change to education.

M: Sure. Which magazines or journals do you typically read?

015: Well, when I get the New England Journal of Medicine, because it’s too scientific and very rarely it’s applied to what I need.

M: Sure.

015: I read just the abstract and the conclusion, the results.

M: Sure.

015: When I’m interested, but I read from first page to the last page of the Internal Medicine News. I like to read that one. Some of the magazines, newest magazines, they keep you informed with what’s going on. They may not be specific, but it gives you an idea.

M: For sure.

015: Because the other one here it’s called internal Medicine News. Yes. I think that’s the one that I like the most.

M: Internal Medicine News. Yeah.

015: It keeps me updated on everything and I don’t have to go to…. I also get the JAMA and I look to see what things I like and then throw them away.

M: Gotcha.

015: I don’t like to read American Family Physicians. It’s really only credit to children, to women. That’s not in my field.

Reference 10 - 3.90% Coverage

: Yes. About fifteen years ago or so, there was an insurance who used to publish the practice guidelines and it was great. It’s a book.

M: They used to publish them?

015: And that book… it was what?

M: You said they used to publish them in a book?

015: Yeah. It was a very nice book and we all had it and then if you wanted to do, let’s say, an MRI, you go there _____27:36 MRI and they will tell you, you need to train first and get the evidence, show that the MRI…. It gives you a very succinct idea of why, whatever command is better…. The way you’re thinking.

M: Ah, interesting.

015: So, those books are no longer provided and I saw it a couple of days ago….. one of those books in the hospital and I said, “I wish they still send those”. I don’t know why they stopped. It really helped a lot of the time.

M: That’s really interesting. You said that it was published by the insurance companies?

015: Yeah, It was published by the insurance company. I don’t remember. I wish I could remember the name of the company or had taken that book from the hospital because no one was using it anyway. I think it was called something like, practice guidelines, or something like that. If I had somebody with a sore throat and I wanted to do a rapid strep

Reference 11 - 0.76% Coverage

it would give me all the information that I needed as opposed to reading the whole big paper.

M: Right. Instead of reading the latest research or publications.

015: It gives a short ___28.52 and the pros and cons… yeah, that was much better.

Reference 12 - 0.95% Coverage

. If we don’t look into that then we look into that they would not have the practice guidelines that are available to us. It would be nice if we had…..like that insurance policy…. the book that was the best. I think that was in the late 1990’s. It was great. We had this book with the practice guidelines.

<Files\\08_15_18_002> - § 2 references coded [3.51% Coverage]

References 1-2 - 3.51% Coverage

as far as resources honestly Dr Morris? is a good resource. He stays on top of everything and he does research , he has articles and they have all kinds of stuff and especially when I moved over here there were a lot of I was set in my ways for antibiotics as a PCP I usually have my same ones for this and this is for UTIs. came over here and people would be allergic to Cipro and Macrobid and Bactrim and I'm like what in the world do you use and he's like well… and so he's a huge resource and Cassie and Gina the nurse practitioners they've been doing acute care even longer than I have so you know I consider my acute care started back in January when I moved over here so their resources as well so I'll forget certain antibiotics so and I realize I could use those.

<Files\\08_15_18_003> - § 4 references coded [7.37% Coverage]

Reference 1 - 1.70% Coverage

reduce your FQ use…What was what influenced you to change this practice?

003-- Yeah I think the medical letter, reading in journals, hearsay like some urologists are saying don’t use this or that for resistance, haven’t done a lot of Cochrane reviews or other research but it may be the medical letters pretty non biased so I like to use that as a resource and maybe just some of the journals kind of sway me …

References 2-3 - 1.88% Coverage

M-- got it and then.It sounds like you use kind of several resources in terms of helping to select antibiotics for you mentioned that FDA Black Box warning you mentioned the medical letter aafp and it sounds like jama too, any other ones or is that mostly it?

003---I use uptodate. Use information from uptodate so that’s another resource and like I said Cochrane review I haven't used it as a resource for UTI as I haven’t really investigated around it.

Reference 4 - 3.79% Coverage

was trying to think of like the deposition who's like set in his ways and I don't want anybody tell me what to do,done this this for years I know what I'm doing and also those more willing to accept, change or discover that hey I'm not doing it correctly or where is that right balance… but if I know the answer. Also where the money comes from …the insurance companies may say hey doctor…ive seen such reports average person is maintaining blood pressure and diabetes and once in a while prescribing an antibiotic. What's the ad maybe something from the insurance companies saying these are the results between peers and I have seen those and kind of make me think for a moment yeah. Maybe something coming from authority like CDC. I mean if you know someone personally or have respect for someone then a conference or a small group meeting with the practice saying hey lets look at it this way may be more influencial.

<Files\\08_15_18_004> - § 1 reference coded [4.94% Coverage]

Reference 1 - 4.94% Coverage

I guess specifically the FDA Black Box warning is that something that influenced your prescribing it or not?

004—A little probably not a lot. We think a lot in the lines of and sort of balance guidelines from you know like the infectious disease society and other things, what their guidelines say and what the practical environment is. You know actually we looked away from inviting people into the office in the days of the close of the capitated HMO patients, so just honestly its an economic thing, it doesn't seem like a bad thing to go ahead and treat and it was cost effective to give someone a $5 antibiotic and not ask them to go through a whole lot of testing or evaluation. Its less expensive and then we just continue to do it or I have anyway.

<Files\\08_23_18_005> - § 5 references coded [13.44% Coverage]

Reference 1 - 3.41% Coverage

Got it. So it sounds like pharmacy calls and then your historical practices and training with

CVS about following that algorithm kinda..

005: 100% they gave me an algorithm, and I think that’s why I kinda use, or where my thought process goes.

M: What are your thoughts about the algorithm? When you were at CVS did you like it, or did you not like it?

005: I liked it, I mean I like to know if this happens here’s this, here’s that, what antibiotic do we not use. I mean I was just kinda trained and programmed that way. But I enjoyed the algorithm, and I still use it in current practice today. I’m sure, I don’t think it's changed that much since then, I keep up, I do some reading and research just to make sure we’re still there. But I liked it.

M: Do you still keep a copy of it with you?

005: It’s very possible I might still have a copy or something like that.

References 2-4 - 5.02% Coverage

Got it. And then, you mentioned some resources that you use when picking antibiotics, your experience with CVS’s algorithm, then your training you mentioned too. Are there others that you use or that you look at periodically?

005: I mean I use epocrates as far as reference, what’s the little book it starts with a...There’s an antibiotic reference guideline.

M: Sanford?

005: Yes, it is Sanford, we have a Sanford guideline on the desk, and they have a current one so we kinda reference that, and then as far as that goes, a lot of patients will come in and say “Oh I get these all the time this one doesn’t work.” And so it’s like okay, that one doesn’t work, and then obviously epoc allows us to see culture reports, so i can go back in and make sure if

there’s no resistance then I start basically and then if there is resistance that kinda changes where my mindset goes bc that will kinda allow me to choose different antibiotics based on past culture reports.

M: Got it. And then, I think other resources too, some people will use up to date, something like that, do you ever use those too?

005: I do have up to date, I’m not an up to date fan, I feel like a lot of those articles are really complicated to get to, so no, generally I don’t use up to date. Have I used it in the past? Yes.

Reference 5 - 5.02% Coverage

Got it. Okay. Great. So, if there were clinical guidelines on how to treat uncomplicated urinary tract infections, how do you think they should be shared with people, like in primary care and convenient care settings?

005: I do better with notebooks as far as a paper reference, but I mean something like up to date would be good, like a computer database. But the computer database, like they actually have to have tables within the articles, so there’s evidence based practice, there’s information that they put out, but then within there it was male UTIs, female UTIs, and had very strict easy to read tables, which was nice as far as you could just flip and reference and go if you had read it before.

M: Got it. But what about if it’s just published alone and it’s not really put anywhere else is that good enough? Is it expected that the provider would go read the primary journal, or it sounds like you prefer kind of a reference article or...

005: Yeah, something kinda straight to the point like, “hey we want you to treat UTIs in this way and a table.” I’m just used to all the other information with it, but a table would be sufficient, like you should use this. Evidence based practice and the research behind it is nice to read once, and then just reference the table after that would be nice.

<Files\\08_23_18_005 (2)> - § 4 references coded [13.42% Coverage]

Reference 1 - 3.41% Coverage

Got it. So it sounds like pharmacy calls and then your historical practices and training with

CVS about following that algorithm kinda..

005: 100% they gave me an algorithm, and I think that’s why I kinda use, or where my thought process goes.

M: What are your thoughts about the algorithm? When you were at CVS did you like it, or did you not like it?

005: I liked it, I mean I like to know if this happens here’s this, here’s that, what antibiotic do we not use. I mean I was just kinda trained and programmed that way. But I enjoyed the algorithm, and I still use it in current practice today. I’m sure, I don’t think it's changed that much since then, I keep up, I do some reading and research just to make sure we’re still there. But I liked it.

M: Do you still keep a copy of it with you?

005: It’s very possible I might still have a copy or something like that.

Reference 2 - 3.73% Coverage

Got it. And then, you mentioned some resources that you use when picking antibiotics, your experience with CVS’s algorithm, then your training you mentioned too. Are there others that you use or that you look at periodically?

005: I mean I use epocrates as far as reference, what’s the little book it starts with a...There’s an antibiotic reference guideline.

M: Sanford?

005: Yes, it is Sanford, we have a Sanford guideline on the desk, and they have a current one so we kinda reference that, and then as far as that goes, a lot of patients will come in and say “Oh I get these all the time this one doesn’t work.” And so it’s like okay, that one doesn’t work, and then obviously epoc allows us to see culture reports, so i can go back in and make sure if

there’s no resistance then I start basically and then if there is resistance that kinda changes where my mindset goes bc that will kinda allow me to choose different antibiotics based on past culture reports.

Reference 3 - 1.26% Coverage

Got it. And then, I think other resources too, some people will use up to date, something like that, do you ever use those too?

005: I do have up to date, I’m not an up to date fan, I feel like a lot of those articles are really complicated to get to, so no, generally I don’t use up to date. Have I used it in the past? Yes.

Reference 4 - 5.02% Coverage

Got it. Okay. Great. So, if there were clinical guidelines on how to treat uncomplicated urinary tract infections, how do you think they should be shared with people, like in primary care and convenient care settings?

005: I do better with notebooks as far as a paper reference, but I mean something like up to date would be good, like a computer database. But the computer database, like they actually have to have tables within the articles, so there’s evidence based practice, there’s information that they put out, but then within there it was male UTIs, female UTIs, and had very strict easy to read tables, which was nice as far as you could just flip and reference and go if you had read it before.

M: Got it. But what about if it’s just published alone and it’s not really put anywhere else is that good enough? Is it expected that the provider would go read the primary journal, or it sounds like you prefer kind of a reference article or...

005: Yeah, something kinda straight to the point like, “hey we want you to treat UTIs in this way and a table.” I’m just used to all the other information with it, but a table would be sufficient, like you should use this. Evidence based practice and the research behind it is nice to read once, and then just reference the table after that would be nice.

<Files\\08_23_18_006> - § 5 references coded [16.80% Coverage]

References 1-5 - 16.80% Coverage

A: I think I look the cipro up every time I have to do that one.
A: Got it, and where do you look up the cipro one?
B: I either use epocrates or Tarascan.
A: Alright, and then in terms of, I think that answered all the questions that we had, and the next one too. Moving along nicely. You mentioned some resources that you look at when selecting antibiotics. Can you tell me how you got into practicing macrobid, where that initially came form?
A: I worked at CVS Medical Clinic prior to here and that was part of the guideline, so that was the only option I had. They had very specific ways that you went through it, so that just became, okay that’s what I do. It usually worked pretty well and people didn’t come back.
A: How did you feel about following this somewhat rigid algorithm? Did you like it, did it bother you?
A: It bother - I don’t mind it, but I like to have the freedom to step outside the box if I need to.
A: Okay, and tell me about stepping outside the box. Gina mentioned a little bit earlier about how that would be harder at CVS before?
B: Correct, you had to be able to document a company accepted reason for not following the guideline or risk, you know, the chart police coming after you. So, you know if you can’t say patient refuses or sometimes you just, think that maybe this might be a better option for this patient, or the patient is just insistent. You don’t always have access to their records, but if someone comes in and says the last time I took macrobid it didn’t work, I’m gonna take your word for that. I’m not gonna argue with you on that point because I wouldn’t want someone to argue with me on that point. So I’ll prescribe you something different. Sometimes they come in with I want this, and then there’s not a lot of room for negotiation, and sometimes it’s, well let’s consider everything you have going on. Sometimes they come in with multiple complaints: Oh I have a UTI, and a URI. Okay, what can i give you that’s gonna take care of both without putting you on multiple antibiotics.
A: Got it, okay, great. And then, in terms of the algorithm that you followed at CVS, you probably don’t have access to it anymore, I think usually they keep them.
A: Correct
A: You just have that algorithm kinda memorized now and you tend to use it as a template?
B: Yes
A: Got it. Okay then, do you ever look at other, beyond the 2 that you mentioned, do you look at other resources like up to date, or do you ever pull up primary literature, or anything like that?
A: If I have time then sometimes I will. Up to date tends to be, the internet doesn’t always cooperate and allow me to use it. It will time out multiple times and then I give up on that. It just depends on time and availability of the resource. Sometimes I’ll ask another provider, alright you’ve been doing this longer than I have, what do you think?

<Files\\09_05_18_007> - § 2 references coded [2.61% Coverage]

References 1-2 - 2.61% Coverage

B: are there specific guidelines or resources you use when you’re trying to select antibiotics and look into treatment duration?
C: Honestly, obviously I use up to date a lot, time to time I’ll use my AFP journals, reviews and all that. But I do use up to date for, at least I have 2 or 3 times a day or more, up to date is up so I can look up something. So, most of the guidelines it from ED, and it’s from the TMEs I’ve attended, that kind of stuff, some of the experience of that that I follow. There are times when I see patients getting antibiotics for 7 to 10 days, where routinely they come to me and say by the way urgent care gave me that, I do not think it’s necessary to treat patients for longer duration of antibiotics for simple UTIs, so I kind of educate them about that.

<Files\\10_10_18_008> - § 1 reference coded [3.80% Coverage]

Reference 1 - 3.80% Coverage

: What other things did you use? Sometimes there's emails that come out on news articles about infectious disease things. I think I get the journal watch periodically emails. Do you get those?

8: Oh I get journal watch every day actually. And I do think that those are kind of helpful. And I actually read them everyday. I'm not exactly sure how great of a resource that would be. Like remember long-term. Whether it was on up-to-date, I know it would be there and I would just go and look there. I think that would be the easiest thing.

<Files\\10_10_18_008 (2)> - § 2 references coded [9.86% Coverage]

Reference 1 - 6.05% Coverage

If clinical guidelines existed on how to treat urinary tract infections, how do you think they should be shared with primary care doctors?

8: Meaning, like up to date or email or a website?

M: Yeah exactly. Do you think that publication in a journal sufficient or do you think that they should be better disseminated, like via up to date. Or, some people use the choosing wisely website and other things too. Although I don't know how many physicians actually look at that.

8: I would say certainly I'm not sure that a journal is enough, unfortunately. So I would say that either choosing wisely, or certainly up-to-date to have some sort of guidelines. I think up to date would honestly be the, unfortunately, most reviewed maybe. But I do think that a journal is probably not enough and some sort of up-to-date or another website would be necessary.

Reference 2 - 3.81% Coverage

What other things did you use? Sometimes there's emails that come out on news articles about infectious disease things. I think I get the journal watch periodically emails. Do you get those?

8: Oh I get journal watch every day actually. And I do think that those are kind of helpful. And I actually read them everyday. I'm not exactly sure how great of a resource that would be. Like remember long-term. Whether it was on up-to-date, I know it would be there and I would just go and look there. I think that would be the easiest thing.

<Files\\11_14_18_009> - § 4 references coded [9.58% Coverage]

Reference 1 - 4.07% Coverage

There’s been instances where the culture came back positive, but the dip was negative, for example.

M: So if you have a strong clinical suspicious but the dip is negative you’ll still send a culture out.

009:Yeah, definitely. Like I said, I read in residency we had a form about this in one of our conferences. Whether you would treat somebody regarding this, and the 2 basic things that they were talking about were that you always wanna get a urinalysis for sure, but a woman’s symptoms sometimes aren’t very accurate, and saying if you have a UTI over a dipstick sometimes, you know? Basically the background knowledge I would use to make a decision

References 2-3 - 3.36% Coverage

Are there specific resources or guidelines that you use on picking antibiotics?

009: Yeah so I go on Up To Date or FP Notebook are the 2 I use the most.

M: I’m not familiar with FP notebook can you tell me a little bit more about that?

009: Yeah so that’s, basically a source that many family medicine residents use as a quick guide to managing medical conditions. It’s kinda like, you know that green book that we had , pocket Medicine, basically it’s like that online. Up to date is more specific, but this is very good quick look.

Reference 4 - 2.15% Coverage

And then UTIs are common enough, do you feel like you go to up to date to look at UTIs or do you feel like you have it down pat?

009: No, like I said, if there’s a complicated case or allergies, or something that doesn’t fit my standard to treat UTIs, then I’d use those as my next step. The frequency of UTI treatment has become pretty routine.

<Files\\12_18_18_010> - § 2 references coded [2.94% Coverage]

References 1-2 - 2.94% Coverage

Yeah. Do you use any specific resources or guidelines when picking antibiotics or treatment durations?

010: I do, sometimes I use hypocrates, or up to date, so I’ll access those. Also I get prescriber's’ letter, the guidelines sometimes shift. I know there’s a one day therapy now they’re recommending as well, so I’ll follow those guidelines.

<Files\\12_19_18_011> - § 4 references coded [15.73% Coverage]

References 1-3 - 7.86% Coverage

J: do you use any specific resources or guidelines when picking antibiotics and durations?

011: Most of the time I check up to date, but the ultimate decision ends up being with patient history, what they react best to.

J: Did you ever you an antibiogram to look at antibiotic resistance in your area?

011: No I haven’t.

M: Do you think that would be useful if there was one that existed in outpatient settings? Historically we’ve looked at it just in the hospital.

011: It would be useful, if it could be easily accessible. Up to date is super easy to access, but if its something within epic that automatically comes up with a suggestion that would be super helpful. Otherwise if it’s something that comes in our email and it’s not right in front of me I’m not gonna remember to use it.

M: That’s a good point.

Reference 4 - 7.87% Coverage

who should be responsible for disseminating them to providers?

011: That’s a good question.

J: Do you think providers should be responsible for looking them up and using them, or the person who writes them?

011: I think it needs to be a little bit of both. That’s part of the reason why we use Up to date and things like that. Just to have the most up to date information, so if it’s given to them then those things should be updated as we have access to them as far as primary care is concerned. As far as ultimate responsibility, whoever is coming up with them should have it, but with privilege comes responsibility, so it’s our privilege to have this information, so we need to be able to access it. It has to be a little bit of both. PCPs, if we’re not making the guidelines, we need to know at least where to find it.

<Files\\12_20_18_012> - § 3 references coded [11.37% Coverage]

Reference 1 - 3.49% Coverage

Do you use any specific resources or guidelines?

012: Not really, I usually like get a UA with the culture and I’ll treat them, and if we have recurrent UTIs, then I think about treating for a longer duration. I don’t think there’s any specific guidelines that I’m following

References 2-3 - 7.89% Coverage

Do you guys ever use antibiograms to look at antibiotics and antibiotic resistance in your area?

012: No, not usually. We have one, but it’s not something I look at very often.

J: How about using that, do you have any thoughts about how that would be helpful for you guys?

012: It’s definitely helpful if you’re not sure which to give. With respect to UTIs, I don’t use it that much You kinda have to start them on something, so I start them on traditional first line treatment, and then I’ll get a culture to see if I need to change the antibiotic. I don’t know that that would really be helpful in this situation.

<Files\\8_13_18_001> - § 2 references coded [3.92% Coverage]

References 1-2 - 3.92% Coverage

factors. in terms of I know that it's I have several friends that are primary care doctors and I know it's very hard to keep up with everything do you use specific guidelines or how do you keep up with things do you think you like especially with some of the antibiotic treatment recommendations,do you do use like up –to- date to look at?

001-Yes uptodate when it works, the internet dosent work here but when it does yes.

M-- that you use on your phone or do you use it…

001—Use one of those portable ?? when it works….

Provider training

Files\\01_08_19_013> - § 1 reference coded [2.63% Coverage]

Reference 1 - 2.63% Coverage

What about electronic order sets or pathways?

013: You know for UTIs that works well, there’s not much of a pathway to follow.

M: You know I think you’re right. When I talk to the residents, cause we ask them the same question, they love the idea of order sets, but I don’t think they think about how it would be conducted. IT’d be pretty short.

<Files\\02_07_19_015> - § 2 references coded [1.08% Coverage]

References 1-2 - 1.08% Coverage

: And it’s based on my experience. I have been doing this for thirty years and I now remember when I came to work with this clinic and the old doctors would say, "My experience the last thirty years." and I would say, "Why don't you read the literature"? After thirty years, believe me, you would say, "I don't want to read anymore, I'm too tired".

<Files\\08_23_18_005> - § 3 references coded [11.46% Coverage]

Reference 1 - 2.65% Coverage

I just feel like macrobid has a lower side effect profile, less people are allergic to macrobid for some reason, cause there's no other drugs in that class, I feel like unless they’re menopausal, after 50, I feel like most are not resistant to it and it’s just kinda the go -to as far as

evidence-based practice from when I was in school that was kinda just what we were taught, especially with cipro, and their recent side effects and the black box warning and stuff, we’ve kind of stayed away from cipro, but I feel like a lot of our practitioners still use it. I just go with macrobid, morning and night for five to seven days, depends on the severity of symptoms, if they’ve had one recently.

Reference 2 - 6.28% Coverage

Alright. And when did you start getting the phone calls, was it recently, or you’ve been getting them for several years?

005: When I worked, prior to here I worked for CVS as a nurse practitioner, we were not allowed to prescribe, we had guidelines that we had to follow, and cipro was like worst case scenario

unless the patient was allergic to everything else. Cipro was our last antibiotic that we went to, and if we used Cipro we had to type a narrative in our note why we chose it like if the patient requested it. It was a very low use antibiotic, because they went off of everything evidence based, and ever since then its kinda been my last antibiotic used, and I feel like we’ve gotten several callbacks here starting in December ‘17, and I feel like we’ve gotten a couple based on, I don’t use it that often, but when I do I feel like we get pharmacy calls a lot.

M: Got it. So it sounds like pharmacy calls and then your historical practices and training with

CVS about following that algorithm kinda..

005: 100% they gave me an algorithm, and I think that’s why I kinda use, or where my thought process goes.

M: What are your thoughts about the algorithm? When you were at CVS did you like it, or did you not like it?

005: I liked it, I mean I like to know if this happens here’s this, here’s that, what antibiotic do we not use. I mean I was just kinda trained and programmed that way. But I enjoyed the algorithm, and I still use it in current practice today. I’m sure, I don’t think it's changed that much since then, I keep up, I do some reading and research just to make sure we’re still there. But I liked it.

Reference 3 - 2.53% Coverage

Got it. And then if you weren’t working in a convenient care setting and you got a phone call from a patient let’s say, would you want them to come in to see you still to get a urine dipstick, or would you feel comfortable treating over the phone, what are your thoughts about that?

005: How I’ve been trained, with my experience, mostly I have been walk-in clinic based, I want the dipstick. If it’s a patient and I’m in a family office and I know them and see their history and it’s something I would feel comfortable saying hey we can do this antibiotic if symptoms aren’t better, follow up. It goes against my gut, but have I done it and will I do it? Yes.

<Files\\08_23_18_005 (2)> - § 1 reference coded [2.53% Coverage]

Reference 1 - 2.53% Coverage

Got it. And then if you weren’t working in a convenient care setting and you got a phone call from a patient let’s say, would you want them to come in to see you still to get a urine dipstick, or would you feel comfortable treating over the phone, what are your thoughts about that?

005: How I’ve been trained, with my experience, mostly I have been walk-in clinic based, I want the dipstick. If it’s a patient and I’m in a family office and I know them and see their history and it’s something I would feel comfortable saying hey we can do this antibiotic if symptoms aren’t better, follow up. It goes against my gut, but have I done it and will I do it? Yes.

<Files\\11_14_18_009> - § 4 references coded [16.79% Coverage]

Reference 1 - 4.07% Coverage

There’s been instances where the culture came back positive, but the dip was negative, for example.

M: So if you have a strong clinical suspicious but the dip is negative you’ll still send a culture out.

009:Yeah, definitely. Like I said, I read in residency we had a form about this in one of our conferences. Whether you would treat somebody regarding this, and the 2 basic things that they were talking about were that you always wanna get a urinalysis for sure, but a woman’s symptoms sometimes aren’t very accurate, and saying if you have a UTI over a dipstick sometimes, you know? Basically the background knowledge I would use to make a decision

References 2-3 - 2.72% Coverage

Got it. And then, when you were thinking about treatin them,do you have certain antibiotics that you prefer to treat UTIs with over others?

009: Yeah, I do. I think that’s not for any specific reason, I think I was just trained, used to prescribing those antibiotics. But definitely look at the culture, the sensitivity, all that stuff. But traditionally we’d do macrobid or cipro are the two tat we would prescribe most of the time.

Reference 4 - 10.00% Coverage

. First one is provider benchmarking, so showing your antibiotic prescribing adherence compared to peers.

009: Yeah I think that is one of the best ways to do it, just shame everyone into doing better. I used to work for a different system before I moved back home to St. Louis, and that’s what they used to do. I think they send out one for Upper Respiratory Infections, and nobody wants to be at the bottom of the list. Everybody went to medical school, everybody is competitive. I think it’s a good way to get people to think in the right direction, so I think that would be a 5 for me.

M: Yeah, so did you think there were any downsides to that?

009: Obviously, some people may take those things personally. Some people take it a little deeper than it really is. THat can create problems in work environments. That you have to be cautious about, but I think overall it does get people to understand that it’s important and we need to start paying attention to is as physicians. If that’s the message then it’s okay.

M: Did they share those results blinded, or were they unblinded?

009: No your name was on there, your score, your rank. They’d send that out every month.

M: What were other people’s opinions about it. Did you hear?

009: I think most people were okay with it, but there may be some individuals who aren’t gonna be concerned about it, and if they continue to have lower marks other people may think negatively about them. Or make a judgment on how they practice medicine. As long as the message is clear and everybody understands the purpose I think it’s not that big of an issue.

**RESPONSIBILITY AND BENCHMARKING**

Perception of evaluation

<Files\\01_08_19_013> - § 1 reference coded [5.07% Coverage]

Reference 1 - 5.07% Coverage

. Provider benchmarking, this would be reporting performance and adherence to guidelines compared to peers.

013: Yeah 4.

M: Got it. So that’s pretty good, you think people look at benchmarking data when it’s given?

013: Yeah, I do medical directory work and that’s what I count on, it seems to be the most non insulting, non invasive, people tend to wanna follow the curve, be the same as other doctors, at least know what other doctors do.

M: That’s fascinating, that’s great, because some people we talk to found it a little insulting about hearing that they fell lower on the benchmark or felt like their patients were sicker, those are excuses we typically here.

<Files\\01_10_19_014> - § 4 references coded [10.68% Coverage]

References 1-2 - 6.41% Coverage

if guidelines existed, do you think providers should be evaluated by adherence to them/

014: Yeah, I do. Because, if we’re gonna honestly say we’re following evidence-based practices, if nobody checks back to see on something that’s pretty concrete like UTIs, then...If you say you are but nobody checks, how can you say you are? Something like that I think would be okay. We get checked off whether we counsel people on their weight, why not on this? It’d probably be better for the community if people weren’t treated for things they didn’t need. I get a lot of “well my last Dr didn’t do it like that” and I’m thinking, yeah they did it wrong! I’m not gonna say that, but it’s a little squirmy for me to treat the way that I believe based on evidence, but other people are doing it differently.

Reference 3 - 2.99% Coverage

: What do you think should be the acceptable adherence rate? How much would be acceptable?

014: You know, I think for that, maybe something like 85%, I know that doesn’t sound high, but I see on other things that I pretty well try to follow the book, but sometimes the book doesn’t work for whatever. To try to give a little clinical leeway, but still make it important.

Reference 4 - 1.28% Coverage

Provider benchmarking, this would be reporting performance and adherence to guidelines compared to peers.

014: I think that would work, I think I could say 5.

<Files\\02_07_19_015> - § 6 references coded [8.78% Coverage]

Reference 1 - 2.42% Coverage

: I hope you address us with that. The nursing home is a pain in the neck.

M: Yes.

015: And Medicare is coming to nursing homes to review UTI’s

M: Yep.

015: And we are… We have to decrease the treatment of unnecessary UTI’s.

M: For sure.

015: So we have the pressure from all these people so when we ask, “Ok, so what criteria”? Oh, you have to make you have to make your own criteria. Then they start looking into what’s doing and whose doing what. What do the companies say…I went to the text, let’s see…about three or four years ago about what the recommendation was and I printed it and then later on apparently that they changed their mind___16:31 (two voices) for the UTI’s in nursing homes. So, that’s for the record, that sometimes they just change their minds.

References 2-3 - 1.66% Coverage

For sure. Do you think that primary care providers should be evaluated based on adherence to best practice guidelines?

015: Yeah, I think that at the beginning we got very ___19:33 because we thought that it was going to be used against us where we have learned that actually, they are improving the quality of care and I like that.

M: Oh, great. What sort of… yeah.

015: Because somebody is doing the reading. Somebody will be reading and doing work for me so I can understand why I’m doing what I am doing. So I don’t mind that.

Reference 4 - 2.41% Coverage

If with the test we did, was the culture done? Did we follow through? Did we talk to the patient?

M: Sure.

015: The patient….. I don’t know that they read every single one but somehow the computer keeps a backdraft.

M: Okay.

015: and we have to do something about it. We have to talk to the patient. If not ______20:27 so that’s the way that they do it, but when we cover the protocols for treatment and I’m not getting ___20:36 they come to us from… there is a doctor that goes from… he’s , from Farmington to Saint Louis and speaks in a group of doctors and they discuss treatment and then they publish them and they bring them to Farmington. So we get that kind of information. That there were medications that were removed, there were protocols that were removed.

Reference 5 - 1.08% Coverage

: And that is because doctors, they have done the studies or they have the experience. They feel that it’s better and we just have to abide by their findings.

M: For sure. For sure.

015: So like I said, I don’t find that as a…. I don’t find it any more…. It doesn’t make me fear that they are doing that. In the beginning it was very defensive.

Reference 6 - 1.22% Coverage

So this idea of provider benchmarking. So the idea that you would get a report every, you know, every quarter or something like that, rating your performance in adherence to guidelines compared to your peers. Do you feel like that would be helpful, or not helpful in improving adherence to guidelines?

015: I feel strongly. Five.

M: Okay. You think that would be very, very helpful. Good.

<Files\\08_15_18_002> - § 3 references coded [7.44% Coverage]

References 1-2 - 5.92% Coverage

that's hard that's difficult to say because each situation is different ,so if there's clinical guidelines we should be following them yes absolutely the problem is how can you say this person was wrong when you don't know the background was what the history was why did they choose that I mean I supposed you know that better documentation in the notice to why we chose certain things might be helpful but even then if there were clinical guidelines on could say based on clinical guidelines this is the best choice perhaps even though clinical guidelines recommend this as the first choice I would choose this because of this reason maybe that would be an option. should you be graded on it though I think would be difficult to say because there's just so many unknowns and so many questions that you can't say you strep is going to be treated by amoxicillin because they're even him for allergic to Amoxicillin you're still not going to do azithromax everytime and people don't like that azithromax for lots of reasons. So the guidelines may say A and B and you're like with B had a horrible experience and allergic to A so I'm going to C and then why didn't you use B because my 20 yrs experience shows that’s not a good choice I've had problems with that other people have reactions or too expensive.

Reference 3 - 1.52% Coverage

so provider Benchmark benchmarking….reporting performance comparison to peers?

002—Esp as a PCP rating or comparing like the big scores and things didn't start making a difference with us until they start affecting our pay to be honest. If it effected pay yeah I think that'd be affective but in general I would say probably ---two

<Files\\08_15_18_003> - § 2 references coded [4.53% Coverage]

Reference 1 - 3.28% Coverage

M—If there were some standards set do you think the provider should be evaluated by their adherence to you know if a clinic or somewhere else made a set of internal guidelines or other recommendations?

003-- like other things at like I really kind of like super expensive like CHF you should be having the ACE blocker etc etc cuz we don't want that 30-day readmission yeah it's very expensive and UTI being way way down on the list of high costs, so probably not a priority to compare in comparison to the really expensive type problems. I don’t know the answer, maybe try to create some ideas may be…specialists in the field coming up with practices or suggesting perhaps we should do this and having maybe a conference to talk about this and deciding on this and this might be a best practice.

Reference 2 - 1.25% Coverage

so provider benchmarking if you were to compare your antibiotic prescribing for UTIs compared to peers and you got the benchmarking data is that useful or not useful using to change behavior? On a scale 1 to 5 One being not useful and five being most most likely to change behavior

003--maybe a 3.5

<Files\\08_15_18_004> - § 3 references coded [6.98% Coverage]

References 1-2 - 5.46% Coverage

M-- so I know that you guys are already evaluated by a lot of other metrics but if if there were metrics on treating urinary tract infections , how they help I guess one is how should they be disseminated to Primary Care Providers and two should those metrics be be used as the quality indicator for providers?

004—Depends how much I agreed with the metrics. I think we have clear cut some of the things we are evaluated on like if hard to see you should be on an aspirin or on the statin. That's pretty clear-cut and hard to dispute. I don’t know that treatment of urinary tract infections that says hard and fast in terms of evidence based but it's one that I would I am very comfortable with what I have been doing, hasn’t changed much in the last decade. So I would not be real enthusiastic about yet another metric to be truthful.

Reference 3 - 1.52% Coverage

So one would be a provider benchmarking, so hearing about what your practices are compared to your peers does that change people's behavior on a scale of 1 to 5?

004--Probably I could give 3 would you pay attention to that stuff

<Files\\08_23_18_005> - § 5 references coded [12.05% Coverage]

References 1-2 - 3.37% Coverage

M: And then for UTIs you mentioned that some antibiotics are better than others. Do you think that primary care providers should be evaluated based on their antibiotic prescribing practices? Should they be measured based on their adherence to them?

005:If there are guidelines to follow or just antibiotics general?

M: If there were guidelines to follow, for example.

005: Yes, and no, because the guidelines don’t work for everybody. Even though there’s a black and white, you should use this and that, you have a patient that goes in and is like there’s no way I’m taking that, and so I mean if you were to provide the evidence in the note or the narratotion that patient refuses this, thus patient was placed on this secondary antibiotic. As

long as there would be a way around the algorithm, yes, I think that would be allowed as long as there were exceptions to the rules.

References 3-4 - 6.89% Coverage

your practice at CVS is that right? 005: Yes.

M: Did they track your adherence to guidelines and algorithms?

005: They did. I don’t know how many percentage of our charts were audited by a nurse practitioner that sat and read, the collaborative physician didn’t necessarily take a look at it. But as far as somebody went and chart reviewed and if they saw something that was out of the algorithm, something that was treated that way, unless there was a narrative note that explained why, they would come back to you. It wouldn’t be like a hand slap, but it would be like you need to put in why, why didn’t you follow the algorithm and kinda bring it to your attention.

M: Did you like that or did you find it intrusive, or can you share your thoughts about that practice?

005: If you’re having a bad day and forgot to chart it you were like come on now. But other than that generally it wasn’t offensive because I expected it. After the first couple of times I got used to it. You were like oh I forgot to put it in there or, cause usually 98% of the time you followed the algorithm to a T unless you had your exceptions.

M: Okay. And what do you think the exceptions were, just that people forgot to document, and at that point if you’re already so adherent to it did you ever actually find opportunities for improvement when they came and gave you feedback or was it mostly just related to lack of documentation in which case you have to kinda wonder if you’re just documenting for documentation?

005: Yeah, I think it was mostly bc I forgot to write in the note why I strayed off of the algorithm. It was like oh I had a busy day, let me go back and make an addendum and throw in why I used that that way, there’s a reason in the chart, but it was documentation just for documentation.

Reference 5 - 1.79% Coverage

The first one is provider benchmarking, so reporting on performance and looking at adherence compared to their peers. So is it 1, not useful, or 5, you think the most useful, or any of the numbers in between.

005: I mean I’d go with a 3 or a 4.

M: Got it.

005: I think that you find a lot of kickback from people bc you find they’re like oh I’ve been practicing thing way for so long, and now you want me to change that, so I think there would be some resistance.

<Files\\08_23_18_005 (2)> - § 3 references coded [11.76% Coverage]

Reference 1 - 3.06% Coverage

Do you think that primary care providers should be evaluated based on their antibiotic prescribing practices? Should they be measured based on their adherence to them?

005:If there are guidelines to follow or just antibiotics general?

M: If there were guidelines to follow, for example.

005: Yes, and no, because the guidelines don’t work for everybody. Even though there’s a black and white, you should use this and that, you have a patient that goes in and is like there’s no way I’m taking that, and so I mean if you were to provide the evidence in the note or the narratotion that patient refuses this, thus patient was placed on this secondary antibiotic. As

long as there would be a way around the algorithm, yes, I think that would be allowed as long as there were exceptions to the rules.

Reference 2 - 6.90% Coverage

your practice at CVS is that right? 005: Yes.

M: Did they track your adherence to guidelines and algorithms?

005: They did. I don’t know how many percentage of our charts were audited by a nurse practitioner that sat and read, the collaborative physician didn’t necessarily take a look at it. But as far as somebody went and chart reviewed and if they saw something that was out of the algorithm, something that was treated that way, unless there was a narrative note that explained why, they would come back to you. It wouldn’t be like a hand slap, but it would be like you need to put in why, why didn’t you follow the algorithm and kinda bring it to your attention.

M: Did you like that or did you find it intrusive, or can you share your thoughts about that practice?

005: If you’re having a bad day and forgot to chart it you were like come on now. But other than that generally it wasn’t offensive because I expected it. After the first couple of times I got used to it. You were like oh I forgot to put it in there or, cause usually 98% of the time you followed the algorithm to a T unless you had your exceptions.

M: Okay. And what do you think the exceptions were, just that people forgot to document, and at that point if you’re already so adherent to it did you ever actually find opportunities for improvement when they came and gave you feedback or was it mostly just related to lack of documentation in which case you have to kinda wonder if you’re just documenting for documentation?

005: Yeah, I think it was mostly bc I forgot to write in the note why I strayed off of the algorithm. It was like oh I had a busy day, let me go back and make an addendum and throw in why I used that that way, there’s a reason in the chart, but it was documentation just for documentation.

Reference 3 - 1.80% Coverage

The first one is provider benchmarking, so reporting on performance and looking at adherence compared to their peers. So is it 1, not useful, or 5, you think the most useful, or any of the numbers in between.

005: I mean I’d go with a 3 or a 4.

M: Got it.

005: I think that you find a lot of kickback from people bc you find they’re like oh I’ve been practicing thing way for so long, and now you want me to change that, so I think there would be some resistance.

<Files\\08_23_18_006> - § 5 references coded [11.84% Coverage]

References 1-2 - 4.11% Coverage

A: Got it. And then, do you think, and you’ve already been measured by this a little bit, but what are your thoughts about if providers should be evaluated by adherence to guidelines, or best practice guidelines?
B: I think that it’s important to make sure that what you’re doing is appropriate, but there has to be wiggle room. It can’t be strict because there’s always going to be other factors that play a part, especially in a culture that promotes patient satisfaction as highly as they promote positive patient outcomes nd appropriateness of decisions. To measure it solely by adherence to guidelines, then there is going to have to be a shirt in that culture of promoting patient satisfaction.

Reference 3 - 0.79% Coverage

C: So the first one is provider benchmarking, so this would be reporting performance or adherence to guidelines compared to peers.
A: 4

Reference 4 - 4.24% Coverage

B: I don’t think anybody likes being, especially as providers, having someone look and say shame on you, you’re not doing this this way, we’re gonna slap you on the hand and tell you no, that we’re not gonna give you a high ranking here. That makes them feel that they’re being judged or treated as a child, so approaching that in a way that doesn’t make it seem as though its punitive, or even making it seem more like a peer to peer type assessment, instead of corporate you’re not doing what I want kind of thing, and making it a professional assessent instead of making it seem more like being treated like a child. I think that’s a big thing that comes in like, I’m gonna do what I want, and you get that stubbornness.

Reference 5 - 2.69% Coverage

C: I just hear other people talk about best in class and some of those other standards that primaries are held to and it’s just, I know they get annoyed with it. It feels like it doesn’t matter what I do, it doesn’t matter that I’m trying to do what’s best for the patient, if I don’t do it just the way somebody else wants me to do, it doesn’t matter, and that puts a lot of stress on everyone trying to meet a benchmark instead of making it patient-centered.

<Files\\09_05_18_007> - § 3 references coded [3.02% Coverage]

References 1-2 - 2.54% Coverage

D: if clinical guidelines for uTIs existed, do you think providers should be evaluated by their adherence to them?
A: Um, providers should be evaluated, I guess, it’s kind of complicated answer to that. I mean, there are guidelines for everything, you know, and we should be, there are guidelines we should be looking into that and making sure we are following them. Time to time, not every patient has a clear picture that fits into the guidelines, so...But I think about 90% of the time with UTIs we

should follow, and I think I’m okay to say that to be evaluated, because I feel like there are unnecessary antibiotics, there are resistance going on, lots and lots of complications with that, and we should be all responsible and have no harm, better stewardship.

Reference 3 - 0.47% Coverage

B: Provider benchmarking, so getting information about your performance compared to your peers about UTI prescribing.
C: I’d say most likely 5.

<Files\\10_10_18_008> - § 1 reference coded [8.23% Coverage]

Reference 1 - 8.23% Coverage

Do you think that providers should be evaluated by their adherence to guidelines, let's say for urinary tract infections?

8: I think in a perfect world, yes.

M: Tell me about in the real world.

8: I don't know. The real world is such a hard job. Evaluated how?

M: That's a good question. There's a lot of things, right? What would be an acceptable adhera

nce rate? How would deviations be captured? I guess other questions are like how Primary Care Providers have so many other competing responsibilities? So, where would this fall on the list of responsibilities that Primary Care Providers have in terms of importance?

8: I think that a punitive evaluation of * probably wouldn't be ideal. I think that maybe feedback on or some sort of report that could be given to them would be helpful just to get feedback on how well they were adhering or not. I don't know, like 75% of the time I think would be pretty darn good for the adherence if they were doing the guidelines appropriately. But, I think just an educational thing would be the most appropriate way to evaluate them. Not necessarily like punitive or reimbursement or things along those lines.

<Files\\10_10_18_008 (2)> - § 2 references coded [10.83% Coverage]

Reference 1 - 8.24% Coverage

Do you think that providers should be evaluated by their adherence to guidelines, let's say for urinary tract infections?

8: I think in a perfect world, yes.

M: Tell me about in the real world.

8: I don't know. The real world is such a hard job. Evaluated how?

M: That's a good question. There's a lot of things, right? What would be an acceptable appearance rate? How would deviations be captured? I guess other questions are like how Primary Care Providers have so many other competing responsibilities? So, where would this fall on the list of responsibilities that Primary Care Providers have in terms of importance?

8: I think that a punitive evaluation of * probably wouldn't be ideal. I think that maybe feedback on or some sort of report that could be given to them would be helpful just to get feedback on how well they were adhering or not. I don't know, like 75% of though time I think would be pretty darn good for the adherence if they were doing the guidelines appropriately. But, I think just an educational thing would be the most appropriate way to evaluate them. Not necessarily like punitive or reimbursement or things along those lines

Reference 2 - 2.60% Coverage

first one, provider benchmarking. So this would be peer comparison. So you'd get a report of your performance like adherence to guidelines to UTIs compared to other people in your Practice Group. Would that be, 1, unlikely to improve adherence, 3, neutral, or 5 being most likely. Obviously you can choose 2 and 4 too. So it's a scale.

8: I would say four to five.

<Files\\11_14_18_009> - § 3 references coded [13.58% Coverage]

References 1-2 - 3.57% Coverage

: If there were guidelines, do you think providers should be evaluated by adherents to them? You guys get so many metrics and other things that you look at now is that valuable?

009: I think that if we’re worried about antibiotic resistance going into the future, and research shows bad outcomes for the general population, then yes and people should be held accountable to a certain extent, whether or not they’re doing what’s been well documented and proven to be the right things to do. Yeah, I think so. I guess how you do that is a little more complicated than it sounds.

Reference 3 - 10.00% Coverage

. First one is provider benchmarking, so showing your antibiotic prescribing adherence compared to peers.

009: Yeah I think that is one of the best ways to do it, just shame everyone into doing better. I used to work for a different system before I moved back home to St. Louis, and that’s what they used to do. I think they send out one for Upper Respiratory Infections, and nobody wants to be at the bottom of the list. Everybody went to medical school, everybody is competitive. I think it’s a good way to get people to think in the right direction, so I think that would be a 5 for me.

M: Yeah, so did you think there were any downsides to that?

009: Obviously, some people may take those things personally. Some people take it a little deeper than it really is. THat can create problems in work environments. That you have to be cautious about, but I think overall it does get people to understand that it’s important and we need to start paying attention to is as physicians. If that’s the message then it’s okay.

M: Did they share those results blinded, or were they unblinded?

009: No your name was on there, your score, your rank. They’d send that out every month.

M: What were other people’s opinions about it. Did you hear?

009: I think most people were okay with it, but there may be some individuals who aren’t gonna be concerned about it, and if they continue to have lower marks other people may think negatively about them. Or make a judgment on how they practice medicine. As long as the message is clear and everybody understands the purpose I think it’s not that big of an issue.

<Files\\12_18_18_010> - § 3 references coded [7.73% Coverage]

References 1-2 - 4.83% Coverage

Yeah. Okay, and if clinical guidelines for UTIs existed, do you think providers should be evaluated on their adherence rates?

010: No, I think we’re evaluated on so much, Best in Class, does everybody have a mammogram, have you had your colonoscopy, immunizations, I think to put that in as another guideline is, nope. I don’t think so because on the surface it should be easy if you have a 24 year old patient, blah blah blah, but they may not know all the ins and outs of individual patients. It wouldn’t take into account all the idiosyncrasies of each patient.

Reference 3 - 2.90% Coverage

Provider benchmarking, reporting compared to peers in a clinic, maybe. How do you rate that?

010: Lowest,.

J: Wouldn't’ be helpful?

010: No, we’re benchmarked on so many things, I don’t think it would be helpful. Then you would get, that would take the focus away from thinking.

J: So you’d give that a 1.

010: Yeah, the worst.

<Files\\12_19_18_011> - § 4 references coded [14.00% Coverage]

References 1-3 - 12.21% Coverage

And do you think that providers should be evaluated how they appear to these guidelines?

011: I think we are with the whole best in class type stuff.

J: I mean specifically for UTIs.

011: Depends how the guidelines are worded. Obviously if they’re saying you need to use cipro vs bactrim vs keflex or however, if it’s like these are your choices and here are the best ones, or this works best, whatever, I think then something like that is fine. If it’s super specific whenever things change with patients and you have to change something, then our rates will go down whenever it’s actually what’s best for the patient. I feel like that’s a really generalized question for something that can be pretty specific.

J: Okay, so you think that providers should be evaluated on their adherence?

011: Yes and no type thing, it’s that yes because guidelines are there for a reason. BUt it’s one of those things like with hypertension. If something changes, we need to be aware of that, it’s like, are we evaluated with that guideline? Yes and no. It’s important to be aware of it, but we’re not dinged or seen as incompetent because we followed that patient. You see what I’m saying? I think it’s there for a reason but should we be evaluated? Maybe take that on a case by case basis.

Reference 4 - 1.79% Coverage

The first is provider benchmarking like reporting of performance compared to peers in a clinic.

011: I would think that’s probably a 4.

J: You think it would be helpful?

011: Yes

<Files\\12_20_18_012> - § 3 references coded [7.06% Coverage]

References 1-2 - 4.62% Coverage

If clinical guidelines for UTIs existed, do you think that providers should be evaluated on their adherence to them in their practice?

012: No, I don’t think so.

J: Can you explain more?

012: We run into problems with patients not in agreement with how long, or what antibiotic, which I know shouldn’t dictate practice but in the real world it kinda does.

Reference 3 - 2.44% Coverage

The first is provider benchmarking like reporting of performance compared to peers in a clinic.

012: I wouldn’t like that.

J: So you’d give that a 1? How about EMR notifications or alerts?

<Files\\8_13_18_001> - § 3 references coded [7.06% Coverage]

References 1-2 - 6.17% Coverage

and then if there were clinical practice guidelines about how to treat UTIs how do you think they should be shared and do u think providers should be evaluated by adherence to them or anything like that. You guys are already evaluated by so many metrics it’s crazy..

001—I would be okay to be evaluated by that than if my patient got a diabetic eye exam or overweight id rather be. You know everyone is so so busy will be be hard to say everyone to attend a lecture about UTI but it would not be nice if you could send it out in an email email for me to know these are the new practice guidelines for this and then as long as I think as long as people knew that there was going to be a metric and they were going to be evaluated or you know you had a certain amount of people that wanted to participate and I think that's fine.

Reference 3 - 0.89% Coverage

What do you think in terms of these interventions so provider benchmarking?

001-- I think that would probably be a four

Perception of responsibility

<Files\\01_10_19_014> - § 3 references coded [8.38% Coverage]

References 1-2 - 5.41% Coverage

Who should be responsible for disseminating guidelines?

014: Well, you, of course! Not me. Because I’m in the medical group, it seems like there must be somebody in the group who could be charged with doing that, even if the information came from an outside source like infectious disease. I know we have nurses, it seems like a lot of things come out with Dr. Pog’s name on them. I think the medical group should do the sending. I don’t know if they have anybody in place that collects that information. I think a nurse would be fine with that, as long as they were given the right resources. Talk to the health department or whoever collects that epidemiology kinda thing.

Reference 3 - 2.98% Coverage

What do you think should be the acceptable adherence rate? How much would be acceptable?

014: You know, I think for that, maybe something like 85%, I know that doesn’t sound high, but I see on other things that I pretty well try to follow the book, but sometimes the book doesn’t work for whatever. To try to give a little clinical leeway, but still make it important.

<Files\\08_15_18_003> - § 1 reference coded [3.53% Coverage]

Reference 1 - 3.53% Coverage

Yes because it is a common condition and no one often tries to pull it up to see if there's anything out there just, speaking of that if there were some guidelines out there in terms of what the first-line antibiotic should be or other things like that, how do you think they should be shared with primary care providers?

And how should they be shared……. using the medical literature sufficient or what do you think?

003--That is a good question……… for a lot of things…it should be mandated and should have the freedom to practice individually. Should we have protocols…like should we culture every urine? That’s kind of standard protocol. Should we use this, this and that as first line? Then you would think each

medical group or practice may want to initiate some guidelines and where is that gonna come from…

Your license is on the line kind of thing……

<Files\\08_23_18_005> - § 1 reference coded [2.30% Coverage]

Reference 1 - 2.30% Coverage

M: Got it. But what about if it’s just published alone and it’s not really put anywhere else is that good enough? Is it expected that the provider would go read the primary journal, or it sounds like you prefer kind of a reference article or...

005: Yeah, something kinda straight to the point like, “hey we want you to treat UTIs in this way and a table.” I’m just used to all the other information with it, but a table would be sufficient, like you should use this. Evidence based practice and the research behind it is nice to read once, and then just reference the table after that would be nice.

<Files\\08_23_18_006> - § 2 references coded [3.67% Coverage]

References 1-2 - 3.67% Coverage

A: If you had working internet do you think it’s the provider responsibility to look up the guidelines, or do you think an institution like CVS should create an algorithm to assist with guideline adherence?
B: That’s a great question. I think that there’s need to be responsibility on both sides. I think it’s the provider’s responsibility to be familiar with what works well, and to be sure that what you’re doing is appropriate in the situation, but I also think if the institution wants to be promoting better health for all their patients, then they should be playing a part in providing that information to the provider.

<Files\\11_14_18_009> - § 1 reference coded [2.84% Coverage]

Reference 1 - 2.84% Coverage

who should be responsible for disseminating them in the clinic?

009: Well it depends on how management is set up. Different systems in different clinics are set up differently. I think this is a good topic that leaders can take that information, share it with doctors, and have it kinda start there and trickle down to staff and management. Make it a lcinic pliciy to implement it on that side of things, come up with and managed by the physician leaders.

<Files\\12_18_18_010> - § 2 references coded [5.28% Coverage]

References 1-2 - 5.28% Coverage

Who do you think should be responsible for disseminating these guidelines?

010: I kinda think it’s incumbent on providers to stay up to date, but in a giant medical system, that’s kinda tough. Wow, I didn’t think about that. Medical directors are usually concerned with running the organization.

J: So you think clinical leadership should have more of a role in disseminating them?

010: I think so. Wouldn’t it be nice if there was a clinical director in a giant medical group whose sole role was to make sure everybody gets the most up to date tidbits every month? Sometimes that’s tough. Wouldn’t that be cool?

<Files\\12_19_18_011> - § 3 references coded [11.59% Coverage]

References 1-3 - 11.59% Coverage

If you assume that there are guidelines for UTIs, who should be responsible for disseminating them to providers?

011: That’s a good question.

J: Do you think providers should be responsible for looking them up and using them, or the person who writes them?

011: I think it needs to be a little bit of both. That’s part of the reason why we use Up to date and things like that. Just to have the most up to date information, so if it’s given to them then those things should be updated as we have access to them as far as primary care is concerned. As far as ultimate responsibility, whoever is coming up with them should have it, but with privilege comes responsibility, so it’s our privilege to have this information, so we need to be able to access it. It has to be a little bit of both. PCPs, if we’re not making the guidelines, we need to know at least where to find it.

J: What do you think should be the role of clinic leadership?

011: They do send out those newsletters, so if leadership wanted to create that little section about guidelines, maybe somebody. Maybe it would be easier for researchers to take those guidelines to leadership and then leadership could disseminate that information to the PCPs.

<Files\\12_20_18_012> - § 2 references coded [10.38% Coverage]

References 1-2 - 10.38% Coverage

Who do you think should be responsible for disseminating these guidelines? Like the people who wrote it, or clinic leadership? Who does the responsibility lie with?

012: I honestly don’t know. Like other guidelines, like new blood pressure or cholesterol, a lot of times it’s drug reps that mention it, I don’t know whose job it is to get the information out.

J: Do you think that providers should be responsible because they’re the ones that are prescribing them?

012: Yes, we are responsible to be knowledgeable on the latest guidelines, but at the same time there’s so many guidelines out there for so many different things it’s almost impossible to keep up with. And different places, they don’t always agree on what is best practice. You have one set from this organization, then another, they might conflict.

Provider peer behavior

<Files\\01_08_19_013> - § 2 references coded [8.96% Coverage]

Reference 1 - 5.07% Coverage

. Provider benchmarking, this would be reporting performance and adherence to guidelines compared to peers.

013: Yeah 4.

M: Got it. So that’s pretty good, you think people look at benchmarking data when it’s given?

013: Yeah, I do medical directory work and that’s what I count on, it seems to be the most non insulting, non invasive, people tend to wanna follow the curve, be the same as other doctors, at least know what other doctors do.

M: That’s fascinating, that’s great, because some people we talk to found it a little insulting about hearing that they fell lower on the benchmark or felt like their patients were sicker, those are excuses we typically here.

Reference 2 - 3.89% Coverage

. If you were designing a study to improve adherents to best practice guidelines, which intervention or interventions would strike the best balance between improving quality of care and being helpful for providers? Can you think of anything outside of what I mentioned?

013: Boy, I mean the ones I marked as higher. Really you have to do this as a campaign, in a physician group, if you can get a few doctors to champion the cause. You need really a whole campaign to get something done, one thing won’t work well.

<Files\\01_10_19_014> - § 1 reference coded [6.41% Coverage]

Reference 1 - 6.41% Coverage

if guidelines existed, do you think providers should be evaluated by adherence to them/

014: Yeah, I do. Because, if we’re gonna honestly say we’re following evidence-based practices, if nobody checks back to see on something that’s pretty concrete like UTIs, then...If you say you are but nobody checks, how can you say you are? Something like that I think would be okay. We get checked off whether we counsel people on their weight, why not on this? It’d probably be better for the community if people weren’t treated for things they didn’t need. I get a lot of “well my last Dr didn’t do it like that” and I’m thinking, yeah they did it wrong! I’m not gonna say that, but it’s a little squirmy for me to treat the way that I believe based on evidence, but other people are doing it differently.

<Files\\02_07_19_015> - § 2 references coded [3.22% Coverage]

Reference 1 - 1.06% Coverage

it’s based on my experience. I have been doing this for thirty years and I now remember when I came to work with this clinic and the old doctors would say, "My experience the last thirty years." and I would say, "Why don't you read the literature"? After thirty years, believe me, you would say, "I don't want to read anymore, I'm too tired".

Reference 2 - 2.15% Coverage

So, say a prominent doctor in one group came out publically and said that they would follow only best practice guidelines with antibiotic prescribing. Do you think that would be helpful for other practices to see? Do you think that would improve adherence?

015: I personally feel that’s helpful, but I do know doctors that don’t appreciate that. So, my belief is the issue would be a number five, but I can tell you that just…. That we go to the medical division meetings and I can see how some doctors that even though we tell them this is the best way, they’re resistant and the problem is that we always say I cannot force another doctor to practice the medicine that I think that is right.

<Files\\08_15_18_002> - § 2 references coded [7.97% Coverage]

Reference 1 - 4.46% Coverage

I am very fond of Macrobid, also very inexpensive , very well tolerated, test if there's a chance they could be pregnant nice to know that it's safe as beyond that I used to do Cipro but the one of the primaries that I worked with she would do super 250 for 3 days never worked. I would get the calls saying I saw this doctor so an so and I've been taking the antibiotic and it didn't get better It didn't change and I look in the chart its Cipro 250th 3 days it didn't work, so I would do Cipro 500 but usually for 5 to 7 days but then this whole thing with the fluoroquinolones in the Black Box warning and then and now try to stay away from any of the fluoroquinolones if I can. So ironically especially if they've already done bactrim or Macrobid go to one of the cephalosporins or even amoxicillin not the best choice but still would hit a lot of the possible bacteria and again very well tolerated very inexpensive usually works pretty well not my first choice but it's an option

Reference 2 - 3.51% Coverage

as far as resources honestly Dr Morris? is a good resource. He stays on top of everything and he does research , he has articles and they have all kinds of stuff and especially when I moved over here there were a lot of I was set in my ways for antibiotics as a PCP I usually have my same ones for this and this is for UTIs. came over here and people would be allergic to Cipro and Macrobid and Bactrim and I'm like what in the world do you use and he's like well… and so he's a huge resource and Cassie and Gina the nurse practitioners they've been doing acute care even longer than I have so you know I consider my acute care started back in January when I moved over here so their resources as well so I'll forget certain antibiotics so and I realize I could use those.

<Files\\08_15_18_003> - § 3 references coded [6.21% Coverage]

Reference 1 - 1.00% Coverage

kind of like people talking or clinicians talking like seeing or trending a lot more resistance to this and we ought to be using cephalexin for the first line or maybe need to get away from the quinolones or watch out for the sulfa reaction.

Reference 2 - 3.79% Coverage

was trying to think of like the deposition who's like set in his ways and I don't want anybody tell me what to do,done this this for years I know what I'm doing and also those more willing to accept, change or discover that hey I'm not doing it correctly or where is that right balance… but if I know the answer. Also where the money comes from …the insurance companies may say hey doctor…ive seen such reports average person is maintaining blood pressure and diabetes and once in a while prescribing an antibiotic. What's the ad maybe something from the insurance companies saying these are the results between peers and I have seen those and kind of make me think for a moment yeah. Maybe something coming from authority like CDC. I mean if you know someone personally or have respect for someone then a conference or a small group meeting with the practice saying hey lets look at it this way may be more influencial.

Reference 3 - 1.41% Coverage

003---the technology part and then probably the older folks are not gonna and also maybe the younger generation

M—We are still running into the same issues right …how much oversight should be done? what do we do with the people that get kicked out from? The study from UCSF suggests that most of them get kicked out of the algorithm so like…

<Files\\08_15_18_004> - § 1 reference coded [4.18% Coverage]

Reference 1 - 4.18% Coverage

A little probably not a lot. We think a lot in the lines of and sort of balance guidelines from you know like the infectious disease society and other things, what their guidelines say and what the practical environment is. You know actually we looked away from inviting people into the office in the days of the close of the capitated HMO patients, so just honestly its an economic thing, it doesn't seem like a bad thing to go ahead and treat and it was cost effective to give someone a $5 antibiotic and not ask them to go through a whole lot of testing or evaluation. Its less expensive and then we just continue to do it or I have anyway.

<Files\\08_23_18_005> - § 1 reference coded [3.45% Coverage]

Reference 1 - 3.45% Coverage

Yeah. And then you mentioned that you like to use macrobid as your first line agent. Can you tell me what your thoughts are about different antibiotics, and why you like macrobid compared to others?

005: I just feel like macrobid has a lower side effect profile, less people are allergic to macrobid for some reason, cause there's no other drugs in that class, I feel like unless they’re menopausal, after 50, I feel like most are not resistant to it and it’s just kinda the go -to as far as

evidence-based practice from when I was in school that was kinda just what we were taught, especially with cipro, and their recent side effects and the black box warning and stuff, we’ve kind of stayed away from cipro, but I feel like a lot of our practitioners still use it. I just go with macrobid, morning and night for five to seven days, depends on the severity of symptoms, if they’ve had one recently.

<Files\\08_23_18_005 (2)> - § 1 reference coded [3.45% Coverage]

Reference 1 - 3.45% Coverage

Yeah. And then you mentioned that you like to use macrobid as your first line agent. Can you tell me what your thoughts are about different antibiotics, and why you like macrobid compared to others?

005: I just feel like macrobid has a lower side effect profile, less people are allergic to macrobid for some reason, cause there's no other drugs in that class, I feel like unless they’re menopausal, after 50, I feel like most are not resistant to it and it’s just kinda the go -to as far as

evidence-based practice from when I was in school that was kinda just what we were taught, especially with cipro, and their recent side effects and the black box warning and stuff, we’ve kind of stayed away from cipro, but I feel like a lot of our practitioners still use it. I just go with macrobid, morning and night for five to seven days, depends on the severity of symptoms, if they’ve had one recently.

<Files\\08_23_18_006> - § 1 reference coded [3.15% Coverage]

Reference 1 - 3.15% Coverage

A: then, do you ever look at other, beyond the 2 that you mentioned, do you look at other resources like up to date, or do you ever pull up primary literature, or anything like that?
A: If I have time then sometimes I will. Up to date tends to be, the internet doesn’t always cooperate and allow me to use it. It will time out multiple times and then I give up on that. It just depends on time and availability of the resource. Sometimes I’ll ask another provider, alright you’ve been doing this longer than I have, what do you think?

<Files\\09_05_18_007> - § 1 reference coded [1.79% Coverage]

Reference 1 - 1.79% Coverage

B: Then you mentioned cipro as one of the preferred antibiotics. Do they have antibiotics that they recommend, and if so what do they usually say?
C: The preferred antibiotic in my experience in talking to the nurses is most of the time cipro, and patients have taken it in the past for these things, and they say that. I don’t hear a lot about macrobid, unfortunately, I don’t know why. We have a lot of patients who are elderly in our practice, so the bactrim is not a favored choice. We hear a lot about cipro and the next is macrobid.

<Files\\10_10_18_008> - § 1 reference coded [5.35% Coverage]

Reference 1 - 5.35% Coverage

M: Got it. And when you're trying to decide between those two, do you think one is stronger than the other or do you think, you mentioned some of the side effects with Cipro that you get worried about. So do you avoid one or some of the antibiotics over the other one, based on, certain patient factors, or what goes into your decision making about the antibiotic? And it's okay if you tell me, you know, and it's completely arbitrary too. That's fine as well.

8: Honestly, looking back, I probably used Cipro more than I should have and I probably should have tried to have used bactrim more. But I think it just tended to become what I was comfortable with. It was just more of an automatic thing more than necessarily looking down at every single factor.

<Files\\11_14_18_009> - § 2 references coded [13.62% Coverage]

Reference 1 - 10.00% Coverage

. First one is provider benchmarking, so showing your antibiotic prescribing adherence compared to peers.

009: Yeah I think that is one of the best ways to do it, just shame everyone into doing better. I used to work for a different system before I moved back home to St. Louis, and that’s what they used to do. I think they send out one for Upper Respiratory Infections, and nobody wants to be at the bottom of the list. Everybody went to medical school, everybody is competitive. I think it’s a good way to get people to think in the right direction, so I think that would be a 5 for me.

M: Yeah, so did you think there were any downsides to that?

009: Obviously, some people may take those things personally. Some people take it a little deeper than it really is. THat can create problems in work environments. That you have to be cautious about, but I think overall it does get people to understand that it’s important and we need to start paying attention to is as physicians. If that’s the message then it’s okay.

M: Did they share those results blinded, or were they unblinded?

009: No your name was on there, your score, your rank. They’d send that out every month.

M: What were other people’s opinions about it. Did you hear?

009: I think most people were okay with it, but there may be some individuals who aren’t gonna be concerned about it, and if they continue to have lower marks other people may think negatively about them. Or make a judgment on how they practice medicine. As long as the message is clear and everybody understands the purpose I think it’s not that big of an issue.

Reference 2 - 3.62% Coverage

I think this is a nice, simple, algorithm, that would be great. If it’s just gonna be a 5 page article explaining everything, primary care doctors are so busy, it’s hard to make time during the day to do something that is gonna take a lot of your time. A lot of family medicine doctors that have been practicing medicine for a long time, they kinda do things their way and it can be kinda difficult to bring change, you know? So making it simple and flexible is the best way to have adherence to whatever you’re trying to accomplish?

M: Kind of a lower number then?

009: Yeah, 3.

<Files\\8_13_18_001> - § 4 references coded [16.63% Coverage]

Reference 1 - 3.82% Coverage

and then displays of public commitment by providers, so this is a new thing I don't know if you're aware of it… they did a study for viral upper respiratory tract infections where they had paste the posters of the providers and they say I'm committed to prescribing antibiotics only for bacterial infections and that reduced abx treatment for viral infections , whether it's you know clear what the underlying factors are for that but that was kinda the idea as display commitment poster.

001—that will be a four

Reference 2 - 4.17% Coverage

--It's hard to say and the reason I said is everybody you know we have a big practice here and I've known those people and everybody is so different so some people are really into you know the following these guidelines and these metrics and there's some people that are like I'm going to do things my way and I don't care and so its difficult to say what is the best way but I do think if there were emails educational you know opportunities and as a practice you could say you know we're going to try and set up these guidelines to follow, its just difficult.

Reference 3 - 5.65% Coverage

u would get greater resistance from other people.

M--In terms of resistance from other people, what problems do you think that they would have with it?

001--they're still just some physicians that don't love the portal and they would probably freak out that there was going to be some automated thing happening behind the scenes but I think it'd be great for patient care, patients could get things taken care of because you know the story I woke up this morning I'm dying of UTI and I'm leaving town or my daughters having a baby or whatever and I don't have time to go to urgent care, I think it I think it be great and I think you're going to have about half the think it's great and have to think anything that you try to do or to make them do is horrible

Reference 4 - 2.99% Coverage

have been in this practice for 24yrs and you know there's nine of us and you get all 9 of us in the room and

M--Well I guess you could have certain providers that would consent and others who wouldn’t. Any concerns like from a revenue standpoint or anything else?

001—No because we r so busy and our nurse practitioners are super busy so I don't think there's a revenue problem I think it's just….

**PROCESS IMPROVEMENT AND NON-PHYSICIAN PRESCRIBING**

Access to care

<Files\\01_10_19_014> - § 3 references coded [11.88% Coverage]

References 1-2 - 3.52% Coverage

Okay. And does one always need to come into your clinic, or do you manage cases over the phone?

014: Because we’re kind of this setting, we hardly ever manage over the phone. There’s so many other causes of dysuria. People will complain and they’ll call it burning, and then when you get to really investigating with them, it turns out its more of a vaginial problem or just an irritant problem. So we hardly ever manage over the phone.

Reference 3 - 8.36% Coverage

: How do you think the patient would respond if the nursing staff did it all?

014: You know, I think that is kind of hard to say. I can think of examples that people, when they call in the clinic, cause I’m an NP, we say doctors aren’t here today if you wanna see the NP. Probably a 1 in 25 will say no. I think that crowd would really find it unacceptable to just have nurses do it, even if they knew. Of people that are willing to accept an NP, most of them, probably they’re just grateful they got seen, and they’d be okay. There’s always a certain percentage that feel like they need a doctor or somebody that’s not got nursing in their name or PA. So, I don’t know that across the board you could get everybody okay with it, until they were further trained. I’d guess as telemedicine gets further along, this would probably really work in that setting. Or, even if they came in physically, because they were so used to seeing different levels of medical people managing different things. Right now there’s still a group that will fight it.

<Files\\02_07_19_015> - § 3 references coded [6.78% Coverage]

Reference 1 - 3.78% Coverage

Ok. Gotcha. Do you ever get phone calls, say, tasks from patients that call into your staff that pass along a message to you? Do you ever get those sorts of messages that patients are calling complaining of either dysuria or sometimes patients are calling and saying that they have a urinary tract infections? Do you get those messages?

015: Yes. We get by phone call, but we also get it through the Epic. The younger patients use the Epic.

M: Ok. The Epic My Chart Messaging.

015: Yes.

M: How do you typically handle those cases? Do you, do you ever treat over the phone, or via the Epic Messaging?

015: I prefer that they just come...if they want to be treated, especially if they want to save money...whatever. They at least should pay for the urinalysis. I want to make sure that before I treat them I know what I am dealing with.

M: Sure.

015: Because these patients, they demand antibiotic. I say, "Ok, you want antibiotic, give me culture." and then within three days I will call and say, "Hey, hey, stop the antibiotic, this is not, you cannot be on antibiotics".

M: Ok. Do you feel like most of the patients present in clinic with these complaints or do you feel like you get more calls and messages?

Reference 2 - 1.11% Coverage

when I show them the negative, this is the evidence. This is what CBC says, but they’re just usually interested for infection. I have not seen a UTI. ____13:54 . Look at this, This is what they say "Look at the television". So I try to educate them but then the problem is going to be this. If I don't give up, in some patients, they go to the Urgent Care.

Reference 3 - 1.89% Coverage

: And when you start practicing you're going to see this…. I get a report every month of how many patients went to the emergency room.

M: Oh wow.

015: How many got admitted and how many did not get admitted, therefore they can come clean with that, that they went to ER and they ___14:38 and that's my fault always. So I have to educate the patient. Don't go to ER, come to me. Come to me. And that’s the point. If I don't do what they want in some patients, they cannot understand what I am saying. They do not want to accept my education. I just do it. I am forced to do it because I don't want to do that.

<Files\\08_15_18_003> - § 1 reference coded [4.55% Coverage]

Reference 1 - 4.55% Coverage

and then, I know you mostly working in clinic setting but if you had if he had someone that called over the phone or something like that or you get your staff have patients call can you talk about do you think that some of the cases can be managed over the phone at all or do you think they should come in all ways or what are your thoughts about ?

003--yeah and when I was in Private Practice I would do that someone calls in and they can't make it in today and its just the weekend sure I'll be glad to call in. Can manage with follow up if not getting better, if severe complications then go to the ER and may want to prescribe something maybe a little bit safer if the patient could be potentially pregnant so I might go to those that may be keflex or macrobid for three to five days.

M--if you're calling them and managing over the phone do you still have them come in later on for a urine test or do you just say that's your history is enough for it or what could be ?

003--normally I would say you don't have to come back and follow up just if you're not better please return and let's reevaluate.

<Files\\09_05_18_007> - § 2 references coded [16.75% Coverage]

Reference 1 - 1.32% Coverage

A: Great. Then, in terms of management, if the patient called over the phone, would you have them come in over the phone for a urine dip, or what do you do if they call over the phone?
A: I think I would like to treat patients like this over the phone so that they don’t have to go to the ER, Urgent Care. It can be safely treated by starting some medications that I have favored to treat simple UTIs

Reference 2 - 15.43% Coverage

B: Got it. And then, when you’re, can you tell me how most of these patients show up to your clinic? Do they, do you get a phone call about them, or do they usually come in, or how do they engage in care with you?
C: Either way, I do get time to time, not very often, once a week, a message that a patient thinks that she has a UTI. Symptoms are similar to what she had in the past, but I know that she hasn’t had frequent or complicated UTI in the past, so I can tell her that I would send her a prescription, she drinks lots of water, if there is associated burning, she can try azo over the counter, and if I try the antibiotic and symptoms get better within 24 hours or at least 48, then she should just finish the course. If it doesn’t, if she has a fever, pain, getting worse, then she

needs to come and evaluate. There are some patients, I don’t see a lot of sick patients, I have more preventative care and follow up, so my nurse practitioner sees my patients who are sick for the most part, so they see a lot of patients, for these common symptoms.

A: That’s fantastic. Can you tell me, for patients that either message you electronically or call your office over the phone, do they how are they evaluated in terms of some of the symptoms that you mentioned that you try to look out for, or the red flags that you’re concerned about maybe a more severe infection?
B: Sure, so yeah, we have a format for our patients of symptoms that the answering people ask them, when did the symptoms start, what are the symptoms, what have you done so far, have you been having any red flags, like if they’re elderly or young, like confusion or fever and chills, and malaise, and all that. Then the message to me or the nurse practitioner, I ask if there is any question about whether they have antibiotics within the last month or so, I ask and send it back, otherwise, if I know the patient very well, and I have no questions then I send a message to my medical assistant to tell the patient to start the antibiotic and the followup things that I discussed if it does not respond within 48 hours, please follow up with us or go to urgent care. TIme to time I have patients who follow up and say oh by the way I think I have a UTI symptom, would you be able to address that today and I do that. I ask the same questions, how long it’s been going on and so forth.
A: So it sounds like if they call the access center you’ll get this templated note kind of with all the appropriate questions answered. I actually met with some of the access center people yesterday, and it sounds like they have a very thorough algorithm that they follow. Do they give you recommendations, I didn’t see anything specifically about recommendations for what antibiotic to prescribe or anything like that. At the end of their message do they tell you that this person looks like they have a UTI or what do they report to you?
B: So they do kind of send a message that patient is complaining of UTI symptoms, including this, this, this, and please address do you want patient to be seen by you, a nurse practitioner, or go to urgent care, or do you want to call a prescription. Most of the time, the patient is like please call me something, I’m miserable and they expect us to take care of them within 3 to 4 hours.
A: I’ve heard that, that there’s like this high priority task that you’re supposed to do in a faster time frame. But they don’t tell you what antibiotic would be recommended or anything like that?
A: No, because I don’t think we have an algorithm for that for them. Most of the time they are non professional, just taking calls. And time to time, when we get RNs who are after hours, after 5 o’clock or after the weekend, they’ll say is that okay if I send patients cipro? That’s their preferred medication for some reason. Is that okay, patient said it helped the patient in the past, so what would you like me to do. So, I don’t think they any algorithms either, the RNs.
A: So, it sounds like some of the nurse, RN triage nurses, when they call you and let you know during after hours that the patient has a UTI, sometimes they’ll give you recommendations about what antibiotic, or maybe they’ll prompt you and say would you like me to call in an antibiotic prescription, is that what you’re saying?
B: Yes, there are times that nurses tell us that patient has used this medication in the past and it helped them Would you be interested for me to send a prescription? So they just talk to us and we tell them yes or no, then they send it electronically to the patient and then we sign the next day or the same day.

<Files\\11_14_18_009> - § 3 references coded [6.60% Coverage]

References 1-2 - 4.10% Coverage

Got it. And then in terms of patients that don’t show up to your clinic, say they call in with these symptoms, walk me through what you do in those circumstances?

009: Yeah, so let’s say somebody called and said I’ve been going to the dr a lot and it burns. I would basically try to work them in that day, so they could come in, do a urine that day, and get it done. If they’re unable to come in, then typically I’d say you have until the end of the day to go to the lab and give them a sample and we’ll give them antibiotics. So we try to always get a urine sample from them, whatever would be most convenient, but we definitely ask for them to come in first.

Reference 3 - 2.49% Coverage

which interventions do you think would have the best balance between improving care and being helpful to providers?

009: I think making it very easy in your clinic or in your lab to come and give a urine sample. I think that’s key. Making that easy for everybody would be very helpful. Like I said having some flexibility in treatment, based on the patient and their relationship with the physician.

<Files\\12_18_18_010> - § 2 references coded [5.18% Coverage]

References 1-2 - 5.18% Coverage

Yeah. Do you ever call in antibiotics without seeing a patient in the office?

010: Very rarely, we try not to in our practice. We like to at least get a hold of that urine. I have dont it, for instance, they’re out of town and can’t get to us, or that call you love to get at 4:30 on a friday afternoon, sometimes I’ll do that but we discourage it. I try very hard not to. If somebody calls up and says hey I’ve got urinary symptoms, if they’re able bodied and in town. If they can’t get to me, sometimes I’ll say go to urgent care. We try very hard not to knee jerk send out antibiotics if we can avoid it.

<Files\\12_19_18_011> - § 1 reference coded [5.22% Coverage]

Reference 1 - 5.22% Coverage

J: Can you manage the case over the phone, or do you always have them come in?

011: It really depends on the patient, if it’s one that they’re just susceptible to UTIs or I’ve treated them before. Like the older population, they’re unable to make it in but they can get the medications delivered, then I may go ahead and call them in things just based on the symptoms, but if it’s a younger patient like the one you mentioned, I’d probably have them come in and discuss, because there could be some other things going on that aren’t just UTI related.

<Files\\12_20_18_012> - § 1 reference coded [4.89% Coverage]

Reference 1 - 4.89% Coverage

Did you ever have patients just call in, and how do you deal with that?

012: I know that like, UTIs are pretty miserable so I’m pretty flexible if they’re on vacation, I don’t always make them come in, or if they can’t get off work. Some of them will call the office, the girls will take on a note on their system, sometimes I’ll call them in an antibiotic if i feel it’s appropriate.

Communication to provider

<Files\\02_07_19_015> - § 1 reference coded [2.46% Coverage]

Reference 1 - 2.46% Coverage

What duration?

M: Yes.

015: In the simple uncomplicated, would be a single dose.

M: Ok.

015: It could be a single dose. If it's a little more long....some patients don't feel comfortable, if she is older or...then I might give her three to seven days depending on how I submitted the urine and if I feel that it is going to be very easy...the culture is probably going to be negative, I don't give too many antibiotics.

M: Ok.

015: If it is over the weekend then I going to have to extend antibiotics because I don't want them to go to the Emergency Room.

M: Ah, for sure.

015: So, if the culture is done, say on a Thursday, Friday, then I’ll start with giving them more antibiotics, but if it's done on a Monday through Wednesday then I'll feel more comfortable calling ___ 07:38

<Files\\11_14_18_009> - § 1 reference coded [4.00% Coverage]

Reference 1 - 4.00% Coverage

in terms of patients that don’t show up to your clinic, say they call in with these symptoms, walk me through what you do in those circumstances?

009: Yeah, so let’s say somebody called and said I’ve been going to the dr a lot and it burns. I would basically try to work them in that day, so they could come in, do a urine that day, and get it done. If they’re unable to come in, then typically I’d say you have until the end of the day to go to the lab and give them a sample and we’ll give them antibiotics. So we try to always get a urine sample from them, whatever would be most convenient, but we definitely ask for them to come in first.

<Files\\12_18_18_010> - § 1 reference coded [5.11% Coverage]

Reference 1 - 5.11% Coverage

Do you ever call in antibiotics without seeing a patient in the office?

010: Very rarely, we try not to in our practice. We like to at least get a hold of that urine. I have dont it, for instance, they’re out of town and can’t get to us, or that call you love to get at 4:30 on a friday afternoon, sometimes I’ll do that but we discourage it. I try very hard not to. If somebody calls up and says hey I’ve got urinary symptoms, if they’re able bodied and in town. If they can’t get to me, sometimes I’ll say go to urgent care. We try very hard not to knee jerk send out antibiotics if we can avoid it.

Non Physician Prescribing

<Files\\01_08_19_013> - § 5 references coded [31.07% Coverage]

References 1-5 - 31.07% Coverage

M: Are there any we haven’t discussed that might be useful? You mentioned the nurse triage algorithms.

013: Yeah, Drs tend to wanna do the right thing, but UTIs are just not high powered medicine. An NP, a nurse under protocol can handle. It’s good to have certain pathways, if they fall out just call the dr.

M: Some previous studies have demonstrated that nursing staff can successfully follow simple algorithms for UTIs, freeing up clinician time for more complicated decisions. Can you share additional thoughts?

013: It needs to a group that sits down and talks about this, commits to the algorithm. Everyone wants to tweak it, you’ve gotta keep hands off once you make it. It’s good to meet the person doing the triaging, face to face time as part of a team.

M: Do you think there might be any major issues?

013: I think it’s fine because Drs will say that we’re gonna get sued. Well no, you don’t get sued for practicing standard of care medicine. And you make the standard of care by doing this. It’s pretty hard for a plaintiff lawyer to say you screwed up when you followed the algorithm that 300 other doctors are following. An individual doctor can’t, you shift the blame. You have to have enough fail safes in there, call the dr ifs.

M:BJC has started to implement their algorithm. Michelle Thomas has been working with the call center nurses for them to do an algorithm. I think right now physicians sign on, and when they call after hours nurses follow this.

013: I may have signed off and not remembered it, respiratory infections for examples.

M: It sounds like it may have been so seamless that you’re not aware they’re doing it, or potentially they haven’t used any of your patients yet.

013: I’m sorry I’m so darn busy I don’t know what I’m doing some days. I occasionally get calls from the call center about somebody falling out of the box. Most patients are accepting it I think, I haven't gotten any complaints from patients. We still round, our group does. My goodness, you need to trust a nurse to make decisions for a 6 month old, you’ve gotta trust them to make decisions for a 6 year old.

M: I think you’re absolutely right. These are great quotes, I think I’m gonna use them in the study. So, that sounds great. One other thing the BJC has also started implementing, e visits now too for UTI. I think they’ve implemented both, a nurse triage algorithm and an e-visit too, where providers then see the patient via communication. I don’t think they skype or anything.

013: Right, we’ve gotta meet the needs of the millennials who don’t want to see a doctor. Everything’s online.

M: They were starting to deploy UTI e-visits. Have you had experience?

013: No, our group isn’t using it. I know some groups were, and I’m going to the physician leadership committee meeting. I know it’s been talked about but I have no idea where it’s being deployed.

M: Between those two competing things, e visit and nurses triage algorithm… do you have a strong opinion?

013: You know, e-visit is still money. I’m still scratching my head about how much money you charge for an e-visit, in value based medicine down the road, you’re paying for something out of your own pocket when you’re charging. If it’s something simple enough for a nurse to do, why am I charging for an e-visit for? If it’s e-visits Monday through Friday but a nurse Saturday and Sunday, that isn’t justice, so I can’t make up my mind about when you do an e-visit vs a nurse.

M: That’s a good question, it’s still being sorted out I think. I guess for a practice that’s trying to maximize revenue, they might do some e-visits to make more money, but the nurse algorithm would probably be fine. I don’t think it’s as easy to recoup costs for nurse driven algorithms.

013:No, if you’re paid, you just get $7,000 per patient per year, adjusted up and down by complexity of illness, after a while it doesn’t make a difference, you’re just taking care of them.

M: That’s you ultimate goal right, taking care of them? This was good. Do you have any questions about the interview?

013: I think you got most everything down.

<Files\\01_10_19_014> - § 3 references coded [14.58% Coverage]

References 1-3 - 14.58% Coverage

Some previous studies have demonstrated that nursing staff can successfully follow simple algorithms for UTIs, freeing up clinician time for more complicated decisions. Can you share additional thoughts?

014: Even to the point of ordering medications?

J: Yes

014: I, in my setting, because I have MAs and LPNs, I think they pretty well do everything but prescribing. They interview, I confirm it, but I look at the medicines and double check the allergies, and make sure that what they’re gonna take is okay for them. I just do a little more investigation before I click prescribe. If somebody complains of a UTI, they put them in the restroom, they get the dip, they order the culture without my direction. I don’t know that I’d be comfortable having them prescribe.

J: How do you think the patient would respond if the nursing staff did it all?

014: You know, I think that is kind of hard to say. I can think of examples that people, when they call in the clinic, cause I’m an NP, we say doctors aren’t here today if you wanna see the NP. Probably a 1 in 25 will say no. I think that crowd would really find it unacceptable to just have nurses do it, even if they knew. Of people that are willing to accept an NP, most of them, probably they’re just grateful they got seen, and they’d be okay. There’s always a certain percentage that feel like they need a doctor or somebody that’s not got nursing in their name or PA. So, I don’t know that across the board you could get everybody okay with it, until they were further trained. I’d guess as telemedicine gets further along, this would probably really work in that setting. Or, even if they came in physically, because they were so used to seeing different levels of medical people managing different things. Right now there’s still a group that will fight it.

<Files\\02_07_19_015> - § 4 references coded [16.61% Coverage]

References 1-4 - 16.61% Coverage

There’s actually been studies that have been done using pilot programs of nursing staff to follow simple algorithms for UTI’s with the goal of freeing up primary care physicians for more complicated decisions. What are your thoughts about nurses or non-physician staff following algorithms for UTI’s.

015: It helps a lot, especially if it’s an outpatient. A patient comes to the clinic and they already follow protocol that makes sense, that saves me time…

M: Okay.

015: I suppose me coming with the patient, figuring out again what the problem is and then ordering that… so it has helped. Especially in the nursing homes. It has reduced significantly the treatment of unnecessary UTI’s.

M: For sure.

015: Some protocols are given to them and here in my office the protocol is somebody with symptoms send them for a urinalysis and then I will see the patient.

M: Say that one more time. The protocol would be to…. If their having symptoms send them for….

015: In my office, if a patient come with symptoms of UTI, the protocol is the patient is to go to the lab to have a urinalysis. Then I see the patient. So by then I have a better understanding what the problem is. In nursing homes the nurses have a different protocol.

M: Okay.

015: With the patients…. And that is to decrease the amount of unnecessary UTI’s.

M: Ah. That’s great.

015: Treatment of what they believe is a UTI. It’s amazing. It’s from just hydration, hydration, hydration, because most of the time the nurses say the urine smells, it’s an infection, or the patient________30:48....its a UTI and stuff like that.

M: So in some of the nursing homes……

015: Recently I had a patient that came to me that I thought he was recurrent of having dementia. I wasn’t sure what was going on. Diabetic. I have not seen for a while because he is very healthy diabetic and he actually got a UTI. The first time as an outpatient that somebody that I think is having dementia. No fever and it was a UTI. He’s a diabetic with UTI. That was unmentioned to me.

M: Wow. Wow.

015: I assume that the nursing home was just saying no he’s just confused and there’s never any fever. Don’t think of UTI. They do urinalysis though. They treat him with water. Whatever they have. Then do urinalysis on the second or third day and they are testing a significant decrease of the unnecessary…. False UTI treatment.

M: Sure. That’s really interesting. When they do the urinalysis though, do they have someone that is able to interpret the results?

015: Yea. Well, they might call the doctor if they don’t know. I’m not sure. Probably they would call the doctor every time.

M: Okay.

015: Because this came from Medicare. Medicare felt that the nursing homes were over treating and they call it an S doc, I don’t know it, because it’s an S doc….. and the nursing home got an S doc and they have to fix that… and it was fixed within the first two months just by doing the protocol about not thinking of UTI’s just because they have urine smell, color.

M: Sure.

015: That kind of happens then a patient debates, what’s in my urine? It smells, my urine is dark, or I’m peeing dirty or my grandpa is not behaving. They catch a bladder infection and then we don’t have urinalysis.

M: Different groups of…. Well…. Have looked at, say, the clinic nurses being able to follow certain guidelines without the physician being involved at all. Some groups have looked at guidelines, or situations where the nurses had a specific algorithm with questions to ask and if they met so many points on the certain criteria scale, that might give them, you know, enough information to treat with antibiotics. What are your thoughts on those sorts of situations where the nursing staff or someone like that would be able to prescribe without the physician?

015: Based on the points, I don’t know if you tell them what antibiotic is the nurse going to use. If they said ok they need some antibiotics. I would feel more comfortable if the nurse says, “I have this patient with UTI, what antibiotic”? , because I feel that I know more about the patient.

M: Sure.

015: But, the small details on the patient.

M: For sure. Alright. How do you think patients would respond to something like that?

015: If they explain that I’m involved in it, they will love it, but if they know that the nurse is doing it, they may not.

M: For sure.

015: Because that’s why they call. They call because they want antibiotics and then somebody asks question and receive the treatment, they’re going to say great. But if they find out it’s the nurse they’re going to say it’s not you it is the nurse practicing medicine.

M: Ok. Well that is all the questions that I have. Anything else that you think would be helpful or anything else that you have noticed or observed about treating urinary tract infections?

015: Oh man, I think that you have conquered the most important things about medications. If we don’t look into that then we look into that they would not have the practice guidelines that are available to us. It would be nice if we had…..like that insurance policy…. the book that was the best. I think that was in the late 1990’s. It was great. We had this book with the practice guidelines.

M: Yeah. It would be interesting if they could come out with something like that again that would be widely accepted and used.

<Files\\08_15_18_002> - § 4 references coded [22.90% Coverage]

Reference 1 - 5.66% Coverage

so you felt like the nurses were over all able to follow some of these triage algorithms but some weren’t?

002—Correct, the one that I worked with when we initially joined BJC, awesome! , everyone still there but in a different part of the office, fabulous! Because I also felt comfortable with what they knew and what they didn't know they wouldn't make up they would be like hey so I don't know what to do with this and they can come to you and I'd ask him so I felt very comfortable with that. Before I left they hired someone who I don't know how she even there she's not qualified she doesn't know a lot of things and if she sent me something I would probably end up cuz they had a triage Center as well so since the MAs would send it to us most the time I sent back to the MAs but if I knew it was her I would send it to the triage center. They would call the nurse or the patient and they would take care of it. Most people ended up sending to the triage center so something needed to be done send it to the triage Center which was in the office and one OP and 2 MA were in the triage center. So if anything just like a refill medicine you sent it to the MA , if it was anything that needed to be addresses it would go to the triage center.

Reference 2 - 3.51% Coverage

as far as resources honestly Dr Morris? is a good resource. He stays on top of everything and he does research , he has articles and they have all kinds of stuff and especially when I moved over here there were a lot of I was set in my ways for antibiotics as a PCP I usually have my same ones for this and this is for UTIs. came over here and people would be allergic to Cipro and Macrobid and Bactrim and I'm like what in the world do you use and he's like well… and so he's a huge resource and Cassie and Gina the nurse practitioners they've been doing acute care even longer than I have so you know I consider my acute care started back in January when I moved over here so their resources as well so I'll forget certain antibiotics so and I realize I could use those.

References 3-4 - 13.72% Coverage

speaking of positive reinforcement potentially interventions that would make people's lives easier. There's been some studies that have shown that Nurse driven or even kiosk related protocols have been studied to evaluate UTIs to where the nurse asks a series of questions or the patient goes up to a kiosk and punches in a series of questions and it spits out yes, you have an uncomplicated urinary tract infection or no, you know you didn't answer all the right for the questions right way to go see a provider about this..

002-- may be the better with the nurse I wouldn't like the kiosk so much because there are other questions at least with the nurses they've been trained okay if this then we need to go off this Direction that's these questions where for a kiosk may not be able to recognize that okay based on even if you program in the medical diagnostics in the background because its human that this part isn’t right and we need this part further and a kiosk cannot do that but a nurse can do that definitely.

M—What about using patient portals and things like that do you do you see that very much used?

002---At the last office the primary care office, the patient could type in their you… send an email basically the MAs would send it to the primaries. The problem is that I found was that I frequently wouldn't get to those messages until at the end of the day and by that time they are irate because they typed that msg 4 hours ago and why haven’t you done anything??? The patient portals need to have a disclaimer that says this may not be addressed today depending on when it's arrived and you know when they were able to take a look at it and I'm frequently you know they would have their like the phone call and I have these symptoms usually they're they're smart enough to put in the right symptoms and you know this sounds like UTI,going on for four months, had it before and a typical phrase this feels like every UTI I've ever had and I'm okay and also if you’ve seen him in last 4-6 months then its ok to go ahead and do that, sometimes if I had extra question I send it to the MA the MA would call them get the question and send it back. As long as they understand it may not be addressed right away, it's not going to be addressed but not in your expected time.

M--especially if you're having UTI symptoms you might want to get evaluated in a timely fashion.

002-- I get it but still you know if I've got 27 patients and you have an email that I haven't had a chance to sit down and look at yet then yeah and that's one of the other reasons I left primary cares because I would do for you doing messages and 7/8 at night and I'm sending out an antibiotic at 7 in the night while they called in at 2 and they're irritated because they have UTI and I havent addressed their symptoms and what they would not be happy. A portal would work if they had a better understanding of how it works.

M-- that sounds good that's great I don't think I have any other questions, you have anything else to add ?

<Files\\08_15_18_003> - § 6 references coded [38.04% Coverage]

References 1-6 - 38.04% Coverage

M--one of the things that some people of explored is to reduce provider burden among another things so it is to use the treatment algorithm where they'll have a nurse follow an algorithm that will lead them to if someone has a urinary tract infection if they're kind of unsure and it needs further clinical evaluation or where they don't and they've done that both for nurses that will fill out those forms and follow the algorithm. There's actually a group in California that has done it with a computer kiosk to where they'll fill out, patient will go to the kiosk and fill out their symptoms, what are your thoughts about that? Do you think that could be helpful? Are there downsides to it? Any thoughts about how those algorithms would work?

003—I used to not like algorithms but now I think they're good because sometimes when you're tired or your brain just full of information, had a busy day and a lot of things going on, it's nice to someone to think for me so I think that that's helpful during a busy day or if your mind somewhere else or when your concentration is not a hundred percent. I think those algorithms are are very helpful in those situations.

M---What about the nurse algorithms? were you know the algorithm is on a kiosk, where the provider would be less involved. What are your thoughts about something like that?

003—Actually like a kiosk is going to have very specific questions. And someone is more likely to have UTI if this..this.. this… this… actually maybe a little bit more clear than somebody on the phone and then the nurse translating or a medical assistant translating that to the physician in writing or verbally maybe there could be a little bit lost in translation. She's calling again and you just have the raw data and you can say hey this is more likely, this is less likely, this is less likely, this one for sure I think that might be actually helpful

M-- yeah you think it would still require physician input to interpret or do you think that an algorithm could give the end of it or could be involved if they meet the all of the, check all the boxes that a prescription could be sent?

003--Okay that's what you're talking about maybe the computer doing the thinking…I would be thinking my job my job…what am I here for?

M---That is a legitimate concern, the other thing would be can they do this too… and they might not be able to do it……

003—Well in that situation where man I'm busy I'm tired in a day I'm trying to get out cuz I got to go to meet my family for dinner and now I'm stuck at the office again I'm frustrated and they keep calling about their UTI or a bad day in the office and someone complained and yeah and you're just not focused I think those are the times were in really you need it …..but would you require oversight??..... in general should have some kind of oversight in general. Creating the algorithm for the questionnaire should be done very well so that, this symptom…. this symptoms… this symptom is highly predictable of it. Some oversight by someone to check about breast feeding etc, I am assuming those are all questions are in the algorithm…..

M—In the other study they had questions related to that.. they had a lot of questions ….but more than half of them still needed evaluation from physicians… and other things like that so there weren't you know it's far from perfect or replacing positions or anything like that but it it's one we're trying to come up with maybe solutions that would make providers happy that would relay accurate information that may be different than the current you know systems about you know benchmarking or notifications and other things cuz the feedback I've gotten from a lot of other providers that they haven't been as useful they don't like to hear about their benchmarking about how they're doing compared to other people. The goal is to explore how people are prescribing and if there's opportunities to improve and if there are how can we do it in a friendly fashion where we're not making providers upset cuz I'm giving them you know a scorecard saying that they did they're doing a bad job this month so that's something where they be happy to maybe reduce their burden or how much time they're spending on some activities, so that's the goal. I'm looking to you as an expert to get ur guidance on what things may work in clinical settings.

003-- were they Kaiser Permanente out there in California?

M—Oh the ones that did the Kiosks? It was UCSF….so the bottom line is the kiosks were not very successful either so. One of the things they notice with the kiosks was that their issues with intrusiveness and other things they set it up in emergency department and other places and they found that in some clinics it worked okay but the kiosks only function was to for you to screen people for UTIs so it became strange in emergency departments where they would say like you go fill out this kiosk and then that would say do you have some complicated issues so you can't get a rapid evaluation or other ones you know if they met all the criteria they go back in a clinician would review it and sign the antibiotic prescription and then, They felt like it was, the kind of a double standard like there was a fast track to do what they used to in the article they said golden ticket you know kind of as a pun for UTI management and to get to the back of the bypass some of the initial evaluation and then the triage nurses didn't like it at all either obviously so because you know they have no incentive to actually do it. So there's a lot of limitations with the kiosk thing one of the things that I'm trying to explore is like could the information from patients be sent more accurately like the feedback I got from providers is u know they will call the kind of the triage phone line and it will be the MA or someone else it's that initially gets a history and then it'll be a further evaluated and then routed to the providers and that can take a while and if you mention some of the information for Lost in Translation so is there some way to have a more accurate data sent to the provider that could be used. One of the things I'm asking about this isn't part of this part at all is is whether or not like a patient like my portal thing in epic could be used where they could report those symptoms and it wouldn't be as large and intrusive as a a kiosk and may become more universally done and could be rapidly adopted.You have to have people with smartphones , I don't know what is the adaption is for smartphones at all either…

003---the technology part and then probably the older folks are not gonna and also maybe the younger generation

M—We are still running into the same issues right …how much oversight should be done? what do we do with the people that get kicked out from? The study from UCSF suggests that most of them get kicked out of the algorithm so like…

003---most like 80??…..

M—Varies based on the setting, in the emergency department settings it was close to 80% got kicked out I think maybe 60% were kicked out of the algorithm for like primary care setting so then what would you do in those circumstances like would they do e- visit then or would you say come to convenient care cuz your case is more complicated or what would be done after hours with that if that if that happened to so lots of questions that are unanswered but those are kind of the one idea that I've got but I'm trying to get more ideas from people about opportunities…

003---since I'm too tech savvy I suppose something can be developed like you're sending email messages to your doctor in the middle of the day or something then pops up into the questionnaire, I hit a button it's going to bring it to the UTI, I have a UTI complaint and ask those questions and gets submitted to the doctor who makes the decision.

M--If you think it's good?….

003---I don't know

M--you tell me……if it’s a good idea or a bad idea.

003—trying to think for myself on a busy day , it might speed up like I was saying before okay this is clearly this is most likely to have a follow up, I know this person has this complications and I like them to be seen etc so may be for some cases this might work and speed things up and be more efficient and more effective. Probably wanna go to 80% effective…

M—Ya it’s a big problem. It was one of the issues with their algorithm too. Can it be improved? I don’t know….and human component still important too

003--I was thinking maybe a nurse practitioner or something even an RN a little bit higher level of education or clinical skills. can ask a few more questions and after that can be ready for the doctor…

M-- you think there might be some people that would game the system then if they wanted to get an antibiotic and what would your thoughts be.

003--I suppose there are a few out there but majority I think actually have some kind of problem they're worried about it actually. There should be a safety kind of thing that the patient was prescribed in the last whatever cuz if they call every month or whatever that would be a problem.

M—That would be a problem for recurrent urinary tract infections or resistance too

003--Yeah maybe to get more back up questions to help

<Files\\08_15_18_004> - § 5 references coded [30.23% Coverage]

References 1-5 - 30.23% Coverage

M—Absolutely, one of the other things I wanted to ask you about is that there's been some study where there's algorithms that nurses or other providers follow beyond just reporting what symptoms they have but actually an algorithm that helps identify uncomplicated urinary tract infections we're at the end of the algorithm, the nurse would either get approval from the physician for the antibiotic or based on a standing order for example the nurse would then prescribed antibiotic under a clinicians name and there's actually been some studies where they've done it with a kiosk so the patient will actually type in their symptoms and the kiosk will go through an algorithm. And the patient will you know get an antibiotic if they checked the right boxes. what are your thoughts about processes like that?

004---We have a nurse call center that takes your calls in the evening and they have a very sophisticated set of algorithms that they follow and they look at the antibiotics for uncomplicated UTI following the algorithm. Sometimes they call us for permission but mostly they don't and then they just put in EPIC and we see it on Monday morning. So we've been doing that now for probably a decade and it has gotten more sophisticated as the group has gotten bigger and they see every doctor at their preference I know they actually work off of I don't know I don't know where they get em but they recognized algorithm set up and evidence-based and tested and all that kind of stuff so.

M—do u feel comfortable with the existing nurse based algorithm where they would prescribe an antibiotic? do you think it works pretty well for you?

004—I love it, patients especially love it. I love it coz I am not bothered for this kind of stuff which I think doesn’t require, you know once we set up the parameters I think that's fine. What patients get are very friendly nurses as opposed to cranky doctor, so works out really well.

M--what do you think about the idea of something being automated in addition to that in terms of you know the next step potentially you know a kiosk or maybe even something in My chart where the patient would fill out a form?

004—I can see that happening but that’s very dependent on the individual. In my geriatric intense practice I many of my older patients don't even get online. The ones that do I am not sure I can trust them to work an algorithm. For the under 40 crowd that would be fine, they would love that.

M--so it sounds like this is something that you might feel comfortable with it if an algorithm like that was incorporated in the EMR, but it wouldn't be as you go on your clinic though?

004—Yes

M-- Do you have a sense of how what percentage of patients go on to my chart or use use that?

004-- that's one of our metrics so I know every month. We’re approaching 50% at my practice. I’ll have a fresh statistic tomorrow morning. Its surprising at how many are using it, so it's not surprising that they're there more than half adult so.

M--Now that's that's good to know. yeah that's one of the things that were interested in exploring is whether or not we could use something like my chart to to have an algorithm that could bypass you know the nursing triage and inautomatically dispense an antibiotic. so that's kind of the we're trying to see how people how comfortable people feel maybe with something like that, so it's a little exploring. it sounds like you would be comfortable with it though so that's good.

004—I would be more comfortable if someone filled in the information and then it went to a nurse and they double-check. I would feel more comfortable. Someone with eyes on..

M--And then would you feel more comfortable cuz you think patients will make mistakes or what would make you feel more comfortable about a nurse double-checking them.

004--Probably I don’t trust the patient that much.

M--So that they may like try to game the system to to get an antibiotic or…

004—I dont think people want abx like that. Having had the experience of people trying to use My Chart and having difficulty I think that's more to the point.

M-- Do you have any other comments to add on to this discussion or did we pretty much cover all your thoughts about it.

004—The only difference I would say is treating older patients with UTI, Itss a different ball point. But that's not really what you're asking its more about young people with uncomplicated bladder infections its pretty straightforward.

M—The older ones can be a little more challenging I guess related to creatinine clearance and other things taking that into account, other medications, QT prolonging drugs etc.

<Files\\08_23_18_005> - § 5 references coded [25.17% Coverage]

References 1-5 - 25.17% Coverage

So, then the algorithms, that’s a great point. There’s been some studies where they've had different people follow algorithms so I think there’s even some clinics with BJC medical group which they call after hours, where the nurses will follow an algorithm too. What are your thoughts about nurse-driven, inter-staff driven algorithms for UTIs?

005: That’s a tough call. I mean, if you have a patient, I think it depends per patient. I mean antibiotics are something I don’t think that just should be given without a confirmation. If you have somebody that has a significant history, could a nurse use an algorithm? I mean, it decreases the patients that are seen in office, but is an antibiotic necessary that doesn’t have bacteria growth, I mean, I think giving a patient an antibiotic without a for sure point-of-care dipstick that shows bacteria growth is kinda harmful to the patient, but there’s a fine line, our healthcare system is do we have the time to see them all? So I mean, if it’s somebody that you’ve seen in office within the last 6 months, I think a nurse-driven algorithm would be acceptable to a certain extent.

M: And then some places have taken it even a step further, where in California they’ve had people use a kiosk, so patients with UTI symptoms will follow the algorithm in a kiosk.

005: Oh we did it at Marcy, Marcy we did online visits. They sent me, it was called an e-visit. As long as you’re getting the appropriate questions and stuff and they know to follow up if the antibiotic isn’t working, I mean that’s just where it gets scary is lack of education. You give somebody an antibiotic, the symptoms don’t go away, and they just continue on it, I mean, as long as there’s proper follow up, and I don’t know, that’s kinda scary.

M: You think it’s scary because there’s nobody supervising it then?

005: Yes, and you’re not getting the urine dipstick to prove why you’re giving them the antibiotic, you’re just throwing them the antibiotic. And I think that’s one of, it’s like narcotics. Like you have a narcotic overprescription and there’s this big opioid epidemic, like antibiotics, this is just person opinion I feel like antibiotics are that way. Somebody calls, they want what they need, and then you give it to them, not giving them what they really need medically, you’re just giving it to the because the patient thinks that they need them. So, I don’t know I think a need basis versus having proof, the point-of-care testing, is the most solid evidence you can get.

M: Some of these kiosks they’ve even eliminate the health care providers for it, so it’s just the patient that’s typing in their symptoms, and then it’ll print out stating that a provider will review the results, potentially, and then sign the script based on the algorithm.

005: If that's what happened with my care all day long, if it started from 8 a.m. til 8 p.m. and I gave a patient an antibiotic based on what they thought they needed, we’d be in trouble. I mean it just, I feel like everybody thinks that that’s their easy fix.

M: Do you think that’s true for all the conditions, or are there certain ones that they tend to do that more with?

005: I think UTI, upper respiratory, viral upper respiratory, they always feel like they need an antibiotic. UTIs, I mean especially as far as that goes, I would say that 80% of the women that come in with the symptoms are positive. I mean, I would have to see a study for the kiosk, like what percentage of women, if they did a dipstick, what percentage of women approached the kiosk and they did a urinalysis follow up actually needed the antibiotic. I would be curious to see the evidence that went behind that.

M: Got it. It sounds like you’re a little skeptical of patients doing, you just worry that the antibiotics would potentially be overprescribed in those settings, is that right?

005: I think so. I mean, maybe not UTI, that might be a safer one

M: But for viral upper respiratory tract infections you’d be more concerned?

005: Yeah, if you had a kiosk for that we’d be in trouble. They’d be resistant to everything.

M: Yeah, and then there’s also a lot of patients that will communicate to providers electronically too. So, you probably get lots of it with the convenient care, but is there a place you think where patients could type in or follow an algorithm electronically on epic where they would reliably do something similar to a kiosk type approach?

005: I think so. I mean it comes to the point where if they send me a message you can review. This would be more primary care because through here we don’t necessarily do the online. But yes, if it’s somebody you’re in a family practice with, they’ve been seen within the last 6 months, you know them personally, they have the history, I think that’s why primary care providers are so important. You get to see that follow up, they get to know the patient, they get to know their history and the pattern that they follow, and I think that’s a little safer as far as giving them antibiotics versus the walk-in clinics kinda like we are. Epic makes it easier to see that without Epic I think it would not be a good idea just to give antibiotics out like that. But being able to see the history and stuff in the computer I think it’s a little bit safer.

M: Got it, so it sounds like you feel like for the most part you’d wanna make sure that there would ba a lot of adequate follow up and exceptions to the algorithm to insure that the appropriate patients are evaluated.

005: Or even if they’ve had urinary symptoms in the past and the cultures have shown positive, that’s even more promising, versus you see a woman come in and she’s got 3 negative urine cultures, and she’s been given antibiotics every time, that’s kinda where okay, odds of you having this, lets come in and let’s get a dipstick before. I think it just depends on the results.

M: Got it. And then if you weren’t working in a convenient care setting and you got a phone call from a patient let’s say, would you want them to come in to see you still to get a urine dipstick, or would you feel comfortable treating over the phone, what are your thoughts about that?

005: How I’ve been trained, with my experience, mostly I have been walk-in clinic based, I want the dipstick. If it’s a patient and I’m in a family office and I know them and see their history and it’s something I would feel comfortable saying hey we can do this antibiotic if symptoms aren’t better, follow up. It goes against my gut, but have I done it and will I do it? Yes.

<Files\\08_23_18_005 (2)> - § 4 references coded [21.14% Coverage]

Reference 1 - 3.33% Coverage

What are your thoughts about nurse-driven, inter-staff driven algorithms for UTIs?

005: That’s a tough call. I mean, if you have a patient, I think it depends per patient. I mean antibiotics are something I don’t think that just should be given without a confirmation. If you have somebody that has a significant history, could a nurse use an algorithm? I mean, it decreases the patients that are seen in office, but is an antibiotic necessary that doesn’t have bacteria growth, I mean, I think giving a patient an antibiotic without a for sure point-of-care dipstick that shows bacteria growth is kinda harmful to the patient, but there’s a fine line, our healthcare system is do we have the time to see them all? So I mean, if it’s somebody that you’ve seen in office within the last 6 months, I think a nurse-driven algorithm would be acceptable to a certain extent.

Reference 2 - 4.86% Coverage

so patients with UTI symptoms will follow the algorithm in a kiosk.

005: Oh we did it at Marcy, Marcy we did online visits. They sent me, it was called an e-visit. As long as you’re getting the appropriate questions and stuff and they know to follow up if the antibiotic isn’t working, I mean that’s just where it gets scary is lack of education. You give somebody an antibiotic, the symptoms don’t go away, and they just continue on it, I mean, as long as there’s proper follow up, and I don’t know, that’s kinda scary.

M: You think it’s scary because there’s nobody supervising it then?

005: Yes, and you’re not getting the urine dipstick to prove why you’re giving them the antibiotic, you’re just throwing them the antibiotic. And I think that’s one of, it’s like narcotics. Like you have a narcotic overprescription and there’s this big opioid epidemic, like antibiotics, this is just person opinion I feel like antibiotics are that way. Somebody calls, they want what they need, and then you give it to them, not giving them what they really need medically, you’re just giving it to the because the patient thinks that they need them. So, I don’t know I think a need basis versus having proof, the point-of-care testing, is the most solid evidence you can get.

Reference 3 - 6.14% Coverage

Some of these kiosks they’ve even eliminate the health care providers for it, so it’s just the patient that’s typing in their symptoms, and then it’ll print out stating that a provider will review the results, potentially, and then sign the script based on the algorithm.

005: If that's what happened with my care all day long, if it started from 8 a.m. til 8 p.m. and I gave a patient an antibiotic based on what they thought they needed, we’d be in trouble. I mean it just, I feel like everybody thinks that that’s their easy fix.

M: Do you think that’s true for all the conditions, or are there certain ones that they tend to do that more with?

005: I think UTI, upper respiratory, viral upper respiratory, they always feel like they need an antibiotic. UTIs, I mean especially as far as that goes, I would say that 80% of the women that come in with the symptoms are positive. I mean, I would have to see a study for the kiosk, like what percentage of women, if they did a dipstick, what percentage of women approached the kiosk and they did a urinalysis follow up actually needed the antibiotic. I would be curious to see the evidence that went behind that.

M: Got it. It sounds like you’re a little skeptical of patients doing, you just worry that the antibiotics would potentially be overprescribed in those settings, is that right?

005: I think so. I mean, maybe not UTI, that might be a safer one

M: But for viral upper respiratory tract infections you’d be more concerned?

005: Yeah, if you had a kiosk for that we’d be in trouble. They’d be resistant to everything.

Reference 4 - 6.82% Coverage

Yeah, and then there’s also a lot of patients that will communicate to providers electronically too. So, you probably get lots of it with the convenient care, but is there a place you think where patients could type in or follow an algorithm electronically on epic where they would reliably do something similar to a kiosk type approach?

005: I think so. I mean it comes to the point where if they send me a message you can review. This would be more primary care because through here we don’t necessarily do the online. But yes, if it’s somebody you’re in a family practice with, they’ve been seen within the last 6 months, you know them personally, they have the history, I think that’s why primary care providers are so important. You get to see that follow up, they get to know the patient, they get to know their history and the pattern that they follow, and I think that’s a little safer as far as giving them antibiotics versus the walk-in clinics kinda like we are. Epic makes it easier to see that without Epic I think it would not be a good idea just to give antibiotics out like that. But being able to see the history and stuff in the computer I think it’s a little bit safer.

M: Got it, so it sounds like you feel like for the most part you’d wanna make sure that there would ba a lot of adequate follow up and exceptions to the algorithm to insure that the appropriate patients are evaluated.

005: Or even if they’ve had urinary symptoms in the past and the cultures have shown positive, that’s even more promising, versus you see a woman come in and she’s got 3 negative urine cultures, and she’s been given antibiotics every time, that’s kinda where okay, odds of you having this, lets come in and let’s get a dipstick before. I think it just depends on the results.

<Files\\08_23_18_006> - § 2 references coded [24.70% Coverage]

References 1-2 - 24.70% Coverage

A: Got it. There’s been some studies in the past that have also looked at having nurses follow simple algorithms for UTIs as well, which could free up clinician time for more complicated decisions. What are your thoughts about nurses, or staff driven algorithms for UTIs?
A: How far are we going on this algorithm? Are they the ones deciding what gets prescribed? Are they the ones saying…
A: So there’s been some studies where they'll have nurses, if the patient meets all the eligibility criteria, then the nurse would be authorized to release the antibiotic prescription based on a standing order?
A: And whose license is that coming back on?
A: I am not sure if that’s standardized about whose license that is.
A: Cause if that’s coming back on my license absolutely not. People make mistakes, right? Just because this algorithm says that, what if there’s something in that patient’s statement that should trigger another question but the nurse doesn’t know that, so she doesn’t ask that question. Maybe something should have been done differently. I think there's’ a lot of potential, and if I’m taking the fall back for it, I want it to be my fault.
A: Got it.
A: Plus, there’s a reason that I went to school. I had 2 patients today that weren’t sure, do I have a UTI or do I have a kidney stone? I get in and look at it, well I don’t think it’s a UTI so, call your urologist. So, how many instances are gonna happen where it is something else and maybe we’ve missed it because we’ve allowed this algorithm to supercede putting our hands on a patient.
A: And then there’s even been some researchers that have looked at using a kiosk where patient would put in their symptoms as well. What are your thoughts on that type of intervention?
A: Well why don’t we just put a vending machine in there while we’re at it? No. Again, that’s a

computer. You’re missing the human component and allowing for error just by the mere fact that nobody took the time to talk so what if I missed something? There wasn’t a question that was asked, and the person can’t even offer it to the computer. At least they can tell the nurse

something else, whether or not she picks it up. You can’t tell a computer anything other than what it’s asking. And the computer isn’t gonna be able to process any extra information, it’s just gonna say yup, all these boxes are checked we’re good to go. That’s not okay.

A: Then, tell me some of your other thoughts, cause the algorithm and other things between some of these would be similar, are nurse related algorithms and computer related algorithms, can you tell me, is one worse than the other, or…
B: Computer is gonna be worse. Because at least with the nurse, if you know the nurse and you know that the nurse is competent, you can at least kind of coach them some, and give them options for hey, if you hear any hesitation in that patient’s voice you just take the call to me. There’s still training and things that can be changed. You can’t change anything on that computer. What you get is what you get.

M:One of the other things too is with the versatility that you’re asking for, some people will also do something called e-visits before, are you familiar with e-visits?

B: The internet type?

A: Yeah, where they might be interviewing a patient or talking with patients about their symptoms based on that type of visit. Can you give me thoughts about those type of practices?
A: I’m not a fan. I understand that they serve a purpose and healthcare is overwhelmed. There’s something to be said for laying eyes on a patient. How many times have I had a patient tell me I have XYZ symptom but then on exam you can’t reproduce that symptom. Or I’ve had a fever but I can’t take your temperature, you can’t tell me what it was, then I don’t know if you really had a fever. I know you say you have back pain, but you can’t tell me where and I can’t put my hands on you to see. No back pain, where’s your pain? You have abdominal pain? I can’t figure out whether or not that reproducible, do we think maybe you have an appendix thing going on, I don’t know because I couldn’t put my hands on you. There’s something to be said for putting your hands on a patient.

<Files\\09_05_18_007> - § 3 references coded [12.26% Coverage]

References 1-3 - 12.26% Coverage

B: And then my last question, previous studies have shown that simple algorithms can help free up clinician time for more complicated decisions. There’s some that use nurses like the call center, that even go down a path where the nurse at the end, if the patient meets all the inclusion criteria, would automatically receive an antibiotic prescription, for example what are your thoughts about that?
C: I’m all for it. I think that having the clear guidelines to provider or nurses, call center, does help. However, obviously the call center don’t prescribe antibiotics, but they can simply see and say what to ask or what questions, if there’s complicated things and all that.
A: Would you feel comfortable if they could provide antibiotics, do you think they would be competent enough to do it?
B: I don’t think so, I don’t think I’m that confident. One of the patients just recently had a macrobid, not anaphylactic but severe allergy, ended up in ICU with sepsis syndrome. I think there are questions, they could have multiple allergies. I think a physician has to make the decision or at least nurse practitioner. I don’t think the call center, in my opinion, should be given that responsibility to prescribe antibiotics.
A: Yeah, can you tell me more about that episode? So the patient was allergic to the antibiotic?
A: Yeah, it was not a simple UTI, I would say. It was a patient who had a history of multiple UTIs but all her treatment was in India, so we had no history on her about what bacteria. She said

well, I had that, and I had several ones, and I was treated. So when she presented with UTI symptoms she had some complicated features, which include, you know, generalized malaise, a little bit of flank pain, but no fever. So treated with usual course, 7 days of macrobid was sent to the patient, and after 3 doses she ended up with hives and lip swelling and just overall she felt like she was gonna pass out. She came to the ER and she was found to have a blood pressure of 80 over 50. She went to ICU. SHe put on pressures. Her blood cultures remained negative, her urine culture came back with e coli, which was sensitive to macrobid but unfortunately she has some allergic reaction to it. It was not a usual case, but she ended up with 14 days IV nitrofurantoin, a multidrug resistant UTI. So, the urologist who saw her said it was multi drug resistant, and we don’t know what the symptoms were initially, recommended her a prophylaxis daily medication, which nothing against it, I don’t use it very often. Patient said I will think about it and follow up with urologist.

A: Wow that’s quite a story.
A: Yeah it is, I mean it’s a learning curve, obviously, because without the cultures and all that she was given macrobid and she ended up with multidrug resistant. But sometimes patients have prior history, good outcome, she did so, later on.
A: So some of the algorithms that the nurses follow aren’t particularly complicated too. There’s been some groups who’ve looked at patients completing algorithms too. Obviously not making prescribing decisions, but there was a group in california at UCSF where they had patients complete the algorithm themselves and then have a medical provider assess them based on their answers to algorithms. What are your thoughts on something like that, where the patient may prompt or complete algorithms before an assessment by a provider?
A: I think it’s a good idea. In fact, we have in our EMR patients before they come to see us have a list of complaints and onset and prompt us. WHen we see patients instead of asking them again, I think that algorithm is a good one to consider that the provider can go for the next step.

<Files\\10_10_18_008> - § 1 reference coded [24.54% Coverage]

Reference 1 - 24.54% Coverage

No, that is fine. So there's been some other studies that have demonstrated that nursing staff can successfully follow algorithms for UTIs. And that could free up clinician time for more complicated decisions. What are your thoughts about that?

8: Yeah, I think as long as it was straightforward and simple enough, I think that would be reasonable. It makes me a little nervous. I think as long as it was simple and they didn't have allergies and it was literally just a straight algorithm, I think with good education that would be potential.

M: So there are other groups that have looked at computer kiosks where the patient will go up to the emergency department and there's a little kiosk for UTIs or electronic visits and other things like that. The kiosks, the provider wouldnt even… it excludes the nurse all together, they would just complete a series of survey questions and then see the provider, and the provider would review the responses and maybe ask the patient some clarification questions and then sign the prescription. What do you think about that?

8: Uh, that makes me nervous. I mean it probably would makes sense that that would work. I just feel like, I guess it's better than just calling them in a prescription over the phone. I would have to think about that one a little bit more.

M: Tell me why that makes you nervous? What aspect of it makes you nervous?

8: I guess it's just different and new, but if you really think about it, it's better than just calling them in a prescription over the phone. I guess still to some extent, their ability to relate information is sometimes questionable, or interpret things appropriately. I guess it would have potential, I'd have to think about it a little bit more.

M: So tell me about how that would compare to, maybe, the telephone management that you sometimes what do on your own. Like if you got a task from a patient saying that they had a UTI, how does your clinic go through with that? Is there someone who calls them to clarify their symptoms or is the prescription written based on a message?

8: No the prescription would be written based on the I think I have a UTI. I think in that aspect, a kiosk would provide more information that would be appropriate or not. So it would probably be more reliable than just calling them in a prescription over the phone, which of course I used to do all the time. So yeah, it's a decent idea.

M: What do you think about patients? Do you think patients would respond to this in terms of feeling comfortable with it too? It sounds like you could see this being used in the future.

8: I would think that potentially, yes. Especially now in the modern world, everyone just wants it quick and easy and painless.

M: So another thing that we're exploring too is related to using electronic medical record software that patients can access through Epic MyChart. So in Epic, there's a chart you can access, like a patient portal, where you can message your providers with your symptoms and other things too. Is that something? One of the things we’re also kicking around is whether or not Epic MyChart could be utilized to help relay some of the symptoms and other things too or reporting UTIs.

8: I think that's certainly also that's just as reliable as a kiosk would be and it's more reliable potentially then someone just calling and saying I have a UTI. So it would be more information gathering than at baseline. I think it could be useful.

<Files\\10_10_18_008 (2)> - § 3 references coded [18.33% Coverage]

Reference 1 - 8.42% Coverage

So there are other groups that have looked at computer kiosks where the patient will go up to the emergency department and there's a little kiosk for UTIs or electronic visits and other things like that. The kiosks, the provider wouldnt even… it excludes the nurse all together, they would just complete a series of survey questions and then see the provider, and the provider would review the responses and maybe ask the patient some clarification questions and then sign the prescription. What do you think about that?

8: Uh, that makes me nervous. I mean it probably would makes sense that that would work. I just feel like, I guess it's better than just calling them in a prescription over the phone. I would have to think about that one a little bit more.

M: Tell me why that makes you nervous? What aspect of it makes you nervous?

8: I guess it's just different and new, but if you really think about it, it's better than just calling them in a prescription over the phone. I guess still to some extent, their ability to relate information is sometimes questionable, or interpret things appropriately. I guess it would have potential, I'd have to think about it a little bit more.

Reference 2 - 4.76% Coverage

So tell me about how that would compare to, maybe, the telephone management that you sometimes what do on your own. Like if you got a task from a patient saying that they had a UTI, how does your clinic go through with that? Is there someone who calls them to clarify their symptoms or is the prescription written based on a message?

8: No the prescription would be written based on the I think I have a UTI. I think in that aspect, a kiosk would provide more information that would be appropriate or not. So it would probably be more reliable than just calling them in a prescription over the phone, which of course I used to do all the time. So yeah, it's a decent idea.

Reference 3 - 5.14% Coverage

So another thing that we're exploring too is related to using electronic medical record software that patients can access through Epic MyChart. So in Epic, there's a chart you can access, like a patient portal, where you can message your providers with your symptoms and other things too. Is that something? One of the things we’re also kicking around is whether or not Epic MyChart could be utilized to help relay some of the symptoms and other things too or reporting UTIs.

8: I think that's certainly also that's just as reliable as a kiosk would be and it's more reliable potentially then someone just calling and saying I have a UTI. So it would be more information gathering than at baseline. I think it could be useful.

<Files\\11_14_18_009> - § 4 references coded [8.16% Coverage]

References 1-2 - 2.51% Coverage

Oh sorry! So there’s been some studies that show that nursing staff can follow simple algorithms and that can free up physician time/

009: I think that if you give the nurses and support staff some algorithms to follow, that’s fine but the final decision should always be made by a physician. I would say that they should be a part and understand what’s going on, but make sure that a doctor signs off.

References 3-4 - 5.65% Coverage

Got it. There’s even some where they have computer kiosks where the patients enter their symptoms and the kiosk will evaluate it and send that information to a provider. THat’s been done in urgent care and emergency conditiins in califiornia, and then the providers sign off. I guess that’s kind of a hybrid where the providers still evaluate, like you’re talkin about. One thing I forgot to ask, for patients that you have a low suspicion based on symptoms for UTIs, do you still have the get cultures? What’s your threshold for getting a culture or urine dip?

009: I would have to suspect that they have a UTI for me to send it off to culture. If I have a low suspicion and urinalysis is negative, I wouldn’t send the culture.

M: What about a urine dip, if you have a low suspicion? Would you still get a urine dip?

009: Yeah if they come in with some symptoms I would definitely get a urine dip.

M

<Files\\12_18_18_010> - § 2 references coded [13.33% Coverage]

References 1-2 - 13.33% Coverage

Okay, so my last question is some previous studies have demonstrated that nursing staff can successfully follow simple algorithms for UTIs, freeing up clinician time for more complicated decisions. What are your thoughts about this?

010: I don’t like it. So, maybe this is the reason we have non clinical people on our phone, and they make a lot of mistakes. They don’t take the right information, they don’t take the right history. We have people put on the schedule who should’ve been sent to the emergency room, or the other way around. So I find myself having to go through schedule and making sure the right people are on it. I’m not there to do triage when people call in, and I don’t, if you’re talking about nurses, I don’t feel good about that. Maybe that’s not fair, but also I am a nurse, and there are so many levels of education nurses receive. You can be an RN, you can be a MSN, you can be a DR, so i have a real problem delegating that out to nurses, not nurse practitioners but nurses.

J: If a patient calls in complaining of a UTI to your clinic, who takes the call and how does it get triaged?

010: It goes from phone people, to medical secretary of provider, then forwarded to provider. They find out if the provider wants them to come in, or does the provider want to treat sight unseen. So, I would say 9 times out of 10 I’m gonna say you need to come in so i can get some urine. There’s extenuating circumstances, like if a patient is very immunocompromised and I don’t wanna take a chance, but...so no, that’s how it goes from one to the other.

<Files\\12_19_18_011> - § 3 references coded [10.48% Coverage]

References 1-3 - 10.48% Coverage

previous studies have demonstrated that nursing staff can successfully follow simple algorithms for UTIs, freeing up clinician time for more complicated decisions. What are your thoughts about this?

011: I think that’s great. I know that they do that a lot with telvid type stuff and it’s very useful. It’s only a 3 day treatment, so if they’re not better they can definitely come in and make an appointment cause they failed outpatient treatment.

J: How do you think antibiotic prescribing would work in such cases?

011: If the algorithm is made very specifically for the nursing staff then it should be fine, because if they don’t meet that certain criteria then they don’t get antibiotics, so they’d come in and give a urine sample and the provider could make that decision.

J: Is it something you see being used in your clinic in the future?

011: Probably as we fill up. I’m a new NP to this practice, and we have 2 new physicians, so we have room to grow. Maybe as providers and practices get too busy with more complicated cases then I could see that becoming more of a utilization of that.

<Files\\12_20_18_012> - § 2 references coded [8.61% Coverage]

References 1-2 - 8.61% Coverage

: Previous studies have demonstrated that nursing staff can successfully follow simple algorithms for UTIs, freeing up clinician time for more complicated decisions. What are your thoughts about this?

012: I mean, I think it would be okay, cause I feel like there are algorithms and it can be pretty simple. If the patient is more complicated it would be referred to the provider rather than the nurse.

J: Is this something you see being used in your clinic in the future?

012: In my clinic? Probably not, just because, there’s not really a need. I have one RN, an LPN, I’m a nurse practitioner, if I’m prescribing medication for somebody, it’s just not something I’d do.

<Files\\8_13_18_001> - § 4 references coded [29.77% Coverage]

References 1-4 - 29.77% Coverage

M—In terms of other things we kind of eluded it before too. You have treatment pathways and nursing algorithms too to help out with reporting symptoms, do you think the nurses or like help desk staff or other people that are answering the phones, do you think that they are adequately trained or do a good job at evaluating this if there was a simple next step and beyond just reporting symptoms they would follow an algorithm where they say you got two out of three symptoms or you had one of this but you had back pain we need to call the doctor instead?

001-It would be better if there was a more detailed algorithm in or more easily set out because now there's not an algorithm for them to treat or call the provider , it's just these are the questions they ask and sometimes filling it out and sometimes they don’t. when we had a different exchange we had a nurse exchange on the weekend they follow an algorithm and they were allowed to send out antibiotics for UTIs and a couple other things and that was pretty good but I think the access center now not medically trained at all and like some people are not even medical people they just answer the phone so its difficult.

M—Previously they had standing orders for it they could actually if they could fit in the algorithm, you would say send them out with three days of Cipro it'll be at your Pharmacy and then you were notified I guess in the future about this.

001—back in the day we got faxes on weekends

M—Do u think that worked? Concerns for safety etc..

001--When we had that our exchange they're all RNs and they were really great and there was no concerns with that and like I said there's no capability for that now with the access center , the people aren't even medically trained so it's difficult.

M-- and then I guess if we take it one step further if there was a way to have patients to report symptoms or fill out or follow the algorithms themselves would you be comfortable with something like that?

001—Oh absolutely, people could go in the portal and do that I don't that would be even better like I said the stuff I get from patients is much more accurate and better than what we get from access center by far.

M--so if someone were to create an intervention where they had a algorithm that patient would follow in their portal and then at the end if it met criteria for an uncomplicated UTI it would send out the script and you'd be comfortable with it?

001—Ya, but I think u would get greater resistance from other people.

M--In terms of resistance from other people, what problems do you think that they would have with it?

001--they're still just some physicians that don't love the portal and they would probably freak out that there was going to be some automated thing happening behind the scenes but I think it'd be great for patient care, patients could get things taken care of because you know the story I woke up this morning I'm dying of UTI and I'm leaving town or my daughters having a baby or whatever and I don't have time to go to urgent care, I think it I think it be great and I think you're going to have about half the think it's great and have to think anything that you try to do or to make them do is horrible.

M-- even if it may make their lives easier

001—Yes doesn’t matter. I have been in this practice for 24yrs and you know there's nine of us and you get all 9 of us in the room and

M--Well I guess you could have certain providers that would consent and others who wouldn’t. Any concerns like from a revenue standpoint or anything else?

001—No because we r so busy and our nurse practitioners are super busy so I don't think there's a revenue problem I think it's just….

M—Anything else we haven't discussed or that you want to talk about in terms of UTI management that…

001--not really. Obviously there has to be a different algorithm for male because of prostatitis etc. It’s better at first to do only female and start out there because the male UTIs are more complicated.

Tasking process

<Files\\02_07_19_015> - § 2 references coded [3.27% Coverage]

Reference 1 - 2.61% Coverage

Do you ever get phone calls, say, tasks from patients that call into your staff that pass along a message to you? Do you ever get those sorts of messages that patients are calling complaining of either dysuria or sometimes patients are calling and saying that they have a urinary tract infections? Do you get those messages?

015: Yes. We get by phone call, but we also get it through the Epic. The younger patients use the Epic.

M: Ok. The Epic My Chart Messaging.

015: Yes.

M: How do you typically handle those cases? Do you, do you ever treat over the phone, or via the Epic Messaging?

015: I prefer that they just come...if they want to be treated, especially if they want to save money...whatever. They at least should pay for the urinalysis. I want to make sure that before I treat them I know what I am dealing with.

M: Sure.

Reference 2 - 0.66% Coverage

Do you feel like most of the patients present in clinic with these complaints or do you feel like you get more calls and messages?

015: No, fortunately most patients come to the office so we can talk about it.

<Files\\08_15_18_002> - § 5 references coded [20.74% Coverage]

References 1-2 - 6.97% Coverage

If you are managing over the phone…can you walk me through the workflow of how you find out about them having a UTI? does like a secretary tell you the information?

002—Once they call the call goes straight to the call center…so if you call you if it says dr. Blair's office number is 1 2 3 4 5 6 7 you call when two three four five six seven and the person atthe call center picks up because it doesn't get directed to our office specifically, so the first person to call center has zero medical background there just transcriptionist so they take the patient's history and they have like a I think it's sort of protocol that they're supposed to ask specific questions and then a nurse and RN will call them back and get more history and then that is sent through epic that is routed to one of the MAs that goes to my after lot of steps but then it would go to my inbox and then I would click on and pull it up read the history you would see what they told the first person what they told the RN and then it would come to me and then I could always put a note to the MAs in my office so it wouldn't go back out at the office so it would stay within the office and one of the MA would call them ask any extra questions and then come back to me again and but in most cases there was enough information for the UTIs that I would say okay with Bactrim DS 1 PO BID 7 Days sent to the pharmacy and then the MA would call them and tell them okay doctor sent antibiotics to the pharmacy, if you dont get better you need to make an appointment.

Reference 3 - 5.65% Coverage

so you felt like the nurses were over all able to follow some of these triage algorithms but some weren’t?

002—Correct, the one that I worked with when we initially joined BJC, awesome! , everyone still there but in a different part of the office, fabulous! Because I also felt comfortable with what they knew and what they didn't know they wouldn't make up they would be like hey so I don't know what to do with this and they can come to you and I'd ask him so I felt very comfortable with that. Before I left they hired someone who I don't know how she even there she's not qualified she doesn't know a lot of things and if she sent me something I would probably end up cuz they had a triage Center as well so since the MAs would send it to us most the time I sent back to the MAs but if I knew it was her I would send it to the triage center. They would call the nurse or the patient and they would take care of it. Most people ended up sending to the triage center so something needed to be done send it to the triage Center which was in the office and one OP and 2 MA were in the triage center. So if anything just like a refill medicine you sent it to the MA , if it was anything that needed to be addresses it would go to the triage center.

Reference 4 - 5.75% Coverage

What about using patient portals and things like that do you do you see that very much used?

002---At the last office the primary care office, the patient could type in their you… send an email basically the MAs would send it to the primaries. The problem is that I found was that I frequently wouldn't get to those messages until at the end of the day and by that time they are irate because they typed that msg 4 hours ago and why haven’t you done anything??? The patient portals need to have a disclaimer that says this may not be addressed today depending on when it's arrived and you know when they were able to take a look at it and I'm frequently you know they would have their like the phone call and I have these symptoms usually they're they're smart enough to put in the right symptoms and you know this sounds like UTI,going on for four months, had it before and a typical phrase this feels like every UTI I've ever had and I'm okay and also if you’ve seen him in last 4-6 months then its ok to go ahead and do that, sometimes if I had extra question I send it to the MA the MA would call them get the question and send it back. As long as they understand it may not be addressed right away, it's not going to be addressed but not in your expected time.

Reference 5 - 2.37% Coverage

I get it but still you know if I've got 27 patients and you have an email that I haven't had a chance to sit down and look at yet then yeah and that's one of the other reasons I left primary cares because I would do for you doing messages and 7/8 at night and I'm sending out an antibiotic at 7 in the night while they called in at 2 and they're irritated because they have UTI and I havent addressed their symptoms and what they would not be happy. A portal would work if they had a better understanding of how it works.

<Files\\08_15_18_003> - § 1 reference coded [3.25% Coverage]

Reference 1 - 3.25% Coverage

and then, I know you mostly working in clinic setting but if you had if he had someone that called over the phone or something like that or you get your staff have patients call can you talk about do you think that some of the cases can be managed over the phone at all or do you think they should come in all ways or what are your thoughts about ?

003--yeah and when I was in Private Practice I would do that someone calls in and they can't make it in today and its just the weekend sure I'll be glad to call in. Can manage with follow up if not getting better, if severe complications then go to the ER and may want to prescribe something maybe a little bit safer if the patient could be potentially pregnant so I might go to those that may be keflex or macrobid for three to five days.

<Files\\08_15_18_004> - § 3 references coded [17.47% Coverage]

References 1-2 - 8.00% Coverage

-Great and then you mentioned that you don't see them in the clinic as often so can you go walk me through what happens when you receive a message about the patient having a UTI… how does it get to you? What’s the content of the message usually?

004--Nine times out of 10 it is a message it's usually texted to me. Our staff is trained to answer sort of red flag questions like fever, CVA tenderness or other symptoms, hematuria also. But most of my patients are pretty well established right now have had bladder infections, they call up and say I've got another bladder infection …can I have antibiotics? We treat them once and ask them to come in after five days to get cultured.

M-- And then so how useful are the messages that you get from your staff do you think they contain all the key information that allows you to make a decision?

004—90% of the time they do. We have a call center and they have protocols and then my secretary screens all of the calls and she's really good. If we are lacking the right information she will go after it.

M-- Did she ever tell you how often the right information is missing? is it a big burden on on her?

004—This is not a big burden because we hammered this thing in our office.

Reference 3 - 9.47% Coverage

Do you have a sense of how what percentage of patients go on to my chart or use use that?

004-- that's one of our metrics so I know every month. We’re approaching 50% at my practice. I’ll have a fresh statistic tomorrow morning. Its surprising at how many are using it, so it's not surprising that they're there more than half adult so.

M--Now that's that's good to know. yeah that's one of the things that were interested in exploring is whether or not we could use something like my chart to to have an algorithm that could bypass you know the nursing triage and inautomatically dispense an antibiotic. so that's kind of the we're trying to see how people how comfortable people feel maybe with something like that, so it's a little exploring. it sounds like you would be comfortable with it though so that's good.

004—I would be more comfortable if someone filled in the information and then it went to a nurse and they double-check. I would feel more comfortable. Someone with eyes on..

M--And then would you feel more comfortable cuz you think patients will make mistakes or what would make you feel more comfortable about a nurse double-checking them.

004--Probably I don’t trust the patient that much.

M--So that they may like try to game the system to to get an antibiotic or…

004—I dont think people want abx like that. Having had the experience of people trying to use My Chart and having difficulty I think that's more to the point.

<Files\\08_23_18_005> - § 3 references coded [12.18% Coverage]

Reference 1 - 4.00% Coverage

So, it sounds like you think it's pretty straightforward overall. I made an uncomplicated case for you too. You mentioned some testing, do you always get testing on everybody, or what makes you decide about testing, and then can you tell me about culture practices too?

005: As far as here, I mean, we’re a walk-in clinic, so nobody can call in and say “Hey I think I have a UTI can you send me something.” They all have to be seen in-office. So in every single patient I’m gonna test, we’re gonna get a point-of-care urine dipstick and then go from there. As far as urine cultures, it’s kind of our practice here to, because it’s not a primary office, they don’t call and say let’s switch antibiotics, so I want to say generally unless they don’t have insurance that were doing a urine culture on everyone, because if there is resistance we can call them back and say “hey we switched it, it wasn’t a UTI you need to follow up elsewhere and get

follow-up care.” So generally we do a urine culture on most everybody, it’s just our practice here.

Reference 2 - 3.33% Coverage

Alright. And when did you start getting the phone calls, was it recently, or you’ve been getting them for several years?

005: When I worked, prior to here I worked for CVS as a nurse practitioner, we were not allowed to prescribe, we had guidelines that we had to follow, and cipro was like worst case scenario

unless the patient was allergic to everything else. Cipro was our last antibiotic that we went to, and if we used Cipro we had to type a narrative in our note why we chose it like if the patient requested it. It was a very low use antibiotic, because they went off of everything evidence based, and ever since then its kinda been my last antibiotic used, and I feel like we’ve gotten several callbacks here starting in December ‘17, and I feel like we’ve gotten a couple based on, I don’t use it that often, but when I do I feel like we get pharmacy calls a lot.

Reference 3 - 4.85% Coverage

so patients with UTI symptoms will follow the algorithm in a kiosk.

005: Oh we did it at Marcy, Marcy we did online visits. They sent me, it was called an e-visit. As long as you’re getting the appropriate questions and stuff and they know to follow up if the antibiotic isn’t working, I mean that’s just where it gets scary is lack of education. You give somebody an antibiotic, the symptoms don’t go away, and they just continue on it, I mean, as long as there’s proper follow up, and I don’t know, that’s kinda scary.

M: You think it’s scary because there’s nobody supervising it then?

005: Yes, and you’re not getting the urine dipstick to prove why you’re giving them the antibiotic, you’re just throwing them the antibiotic. And I think that’s one of, it’s like narcotics. Like you have a narcotic overprescription and there’s this big opioid epidemic, like antibiotics, this is just person opinion I feel like antibiotics are that way. Somebody calls, they want what they need, and then you give it to them, not giving them what they really need medically, you’re just giving it to the because the patient thinks that they need them. So, I don’t know I think a need basis versus having proof, the point-of-care testing, is the most solid evidence you can get.

<Files\\08_23_18_005 (2)> - § 3 references coded [12.18% Coverage]

Reference 1 - 4.00% Coverage

So, it sounds like you think it's pretty straightforward overall. I made an uncomplicated case for you too. You mentioned some testing, do you always get testing on everybody, or what makes you decide about testing, and then can you tell me about culture practices too?

005: As far as here, I mean, we’re a walk-in clinic, so nobody can call in and say “Hey I think I have a UTI can you send me something.” They all have to be seen in-office. So in every single patient I’m gonna test, we’re gonna get a point-of-care urine dipstick and then go from there. As far as urine cultures, it’s kind of our practice here to, because it’s not a primary office, they don’t call and say let’s switch antibiotics, so I want to say generally unless they don’t have insurance that were doing a urine culture on everyone, because if there is resistance we can call them back and say “hey we switched it, it wasn’t a UTI you need to follow up elsewhere and get

follow-up care.” So generally we do a urine culture on most everybody, it’s just our practice here.

Reference 2 - 3.33% Coverage

Alright. And when did you start getting the phone calls, was it recently, or you’ve been getting them for several years?

005: When I worked, prior to here I worked for CVS as a nurse practitioner, we were not allowed to prescribe, we had guidelines that we had to follow, and cipro was like worst case scenario

unless the patient was allergic to everything else. Cipro was our last antibiotic that we went to, and if we used Cipro we had to type a narrative in our note why we chose it like if the patient requested it. It was a very low use antibiotic, because they went off of everything evidence based, and ever since then its kinda been my last antibiotic used, and I feel like we’ve gotten several callbacks here starting in December ‘17, and I feel like we’ve gotten a couple based on, I don’t use it that often, but when I do I feel like we get pharmacy calls a lot.

Reference 3 - 4.86% Coverage

so patients with UTI symptoms will follow the algorithm in a kiosk.

005: Oh we did it at Marcy, Marcy we did online visits. They sent me, it was called an e-visit. As long as you’re getting the appropriate questions and stuff and they know to follow up if the antibiotic isn’t working, I mean that’s just where it gets scary is lack of education. You give somebody an antibiotic, the symptoms don’t go away, and they just continue on it, I mean, as long as there’s proper follow up, and I don’t know, that’s kinda scary.

M: You think it’s scary because there’s nobody supervising it then?

005: Yes, and you’re not getting the urine dipstick to prove why you’re giving them the antibiotic, you’re just throwing them the antibiotic. And I think that’s one of, it’s like narcotics. Like you have a narcotic overprescription and there’s this big opioid epidemic, like antibiotics, this is just person opinion I feel like antibiotics are that way. Somebody calls, they want what they need, and then you give it to them, not giving them what they really need medically, you’re just giving it to the because the patient thinks that they need them. So, I don’t know I think a need basis versus having proof, the point-of-care testing, is the most solid evidence you can get.

<Files\\09_05_18_007> - § 4 references coded [16.79% Coverage]

Reference 1 - 3.62% Coverage

A: Got it. And then, when you’re, can you tell me how most of these patients show up to your clinic? Do they, do you get a phone call about them, or do they usually come in, or how do they engage in care with you?
B: Either way, I do get time to time, not very often, once a week, a message that a patient thinks that she has a UTI. Symptoms are similar to what she had in the past, but I know that she hasn’t had frequent or complicated UTI in the past, so I can tell her that I would send her a prescription, she drinks lots of water, if there is associated burning, she can try azo over the counter, and if I try the antibiotic and symptoms get better within 24 hours or at least 48, then she should just finish the course. If it doesn’t, if she has a fever, pain, getting worse, then she

needs to come and evaluate. There are some patients, I don’t see a lot of sick patients, I have more preventative care and follow up, so my nurse practitioner sees my patients who are sick for the most part, so they see a lot of patients, for these common symptoms

Reference 2 - 7.35% Coverage

C: Can you tell me, for patients that either message you electronically or call your office over the phone, do they how are they evaluated in terms of some of the symptoms that you mentioned that you try to look out for, or the red flags that you’re concerned about maybe a more severe infection?
D: Sure, so yeah, we have a format for our patients of symptoms that the answering people ask them, when did the symptoms start, what are the symptoms, what have you done so far, have you been having any red flags, like if they’re elderly or young, like confusion or fever and chills, and malaise, and all that. Then the message to me or the nurse practitioner, I ask if there is any question about whether they have antibiotics within the last month or so, I ask and send it back, otherwise, if I know the patient very well, and I have no questions then I send a message to my medical assistant to tell the patient to start the antibiotic and the followup things that I discussed if it does not respond within 48 hours, please follow up with us or go to urgent care. TIme to time I have patients who follow up and say oh by the way I think I have a UTI symptom, would you be able to address that today and I do that. I ask the same questions, how long it’s been going on and so forth.
A: So it sounds like if they call the access center you’ll get this templated note kind of with all the appropriate questions answered. I actually met with some of the access center people yesterday, and it sounds like they have a very thorough algorithm that they follow. Do they give you recommendations, I didn’t see anything specifically about recommendations for what antibiotic to prescribe or anything like that. At the end of their message do they tell you that this person looks like they have a UTI or what do they report to you?
B: So they do kind of send a message that patient is complaining of UTI symptoms, including this, this, this, and please address do you want patient to be seen by you, a nurse practitioner, or go to urgent care, or do you want to call a prescription. Most of the time, the patient is like please call me something, I’m miserable and they expect us to take care of them within 3 to 4 hours.

Reference 3 - 4.37% Coverage

A: I’ve heard that, that there’s like this high priority task that you’re supposed to do in a faster time frame. But they don’t tell you what antibiotic would be recommended or anything like that?
B: No, because I don’t think we have an algorithm for that for them. Most of the time they are non professional, just taking calls. And time to time, when we get RNs who are after hours, after 5 o’clock or after the weekend, they’ll say is that okay if I send patients cipro? That’s their preferred medication for some reason. Is that okay, patient said it helped the patient in the past, so what would you like me to do. So, I don’t think they any algorithms either, the RNs.
A: So, it sounds like some of the nurse, RN triage nurses, when they call you and let you know during after hours that the patient has a UTI, sometimes they’ll give you recommendations about what antibiotic, or maybe they’ll prompt you and say would you like me to call in an antibiotic prescription, is that what you’re saying?
B: Yes, there are times that nurses tell us that patient has used this medication in the past and it helped them Would you be interested for me to send a prescription? So they just talk to us and we tell them yes or no, then they send it electronically to the patient and then we sign the next day or the same day.

Reference 4 - 1.44% Coverage

C: These days, EMR patients see their own results in 24 hours, they just panic, like what is going on with my urine, why is there red flag. So it creates a lot of calls, emails, for out patients, but they understand that we get back to them if there is anything abnormal right away. Time to time we get that.
A: I could imagine that would sometimes be disconcerting for patients when they see that.
B: This is the life of a private care.

<Files\\10_10_18_008 (2)> - § 2 references coded [6.94% Coverage]

Reference 1 - 2.18% Coverage

Got it okay. And would it change if the patient, if you got a message or got a telephone call about a patient having these symptoms? Would you still get testing or would you treat them empirically? Can you walk me through that?

8: If it was on a phone call, I would probably just treat them empirically.

Reference 2 - 4.76% Coverage

So tell me about how that would compare to, maybe, the telephone management that you sometimes what do on your own. Like if you got a task from a patient saying that they had a UTI, how does your clinic go through with that? Is there someone who calls them to clarify their symptoms or is the prescription written based on a message?

8: No the prescription would be written based on the I think I have a UTI. I think in that aspect, a kiosk would provide more information that would be appropriate or not. So it would probably be more reliable than just calling them in a prescription over the phone, which of course I used to do all the time. So yeah, it's a decent idea.

<Files\\12_18_18_010> - § 2 references coded [12.93% Coverage]

Reference 1 - 8.14% Coverage

studies have demonstrated that nursing staff can successfully follow simple algorithms for UTIs, freeing up clinician time for more complicated decisions. What are your thoughts about this?

010: I don’t like it. So, maybe this is the reason we have non clinical people on our phone, and they make a lot of mistakes. They don’t take the right information, they don’t take the right history. We have people put on the schedule who should’ve been sent to the emergency room, or the other way around. So I find myself having to go through schedule and making sure the right people are on it. I’m not there to do triage when people call in, and I don’t, if you’re talking about nurses, I don’t feel good about that. Maybe that’s not fair, but also I am a nurse, and there are so many levels of education nurses receive. You can be an RN, you can be a MSN, you can be a DR, so i have a real problem delegating that out to nurses, not nurse practitioners but nurses.

Reference 2 - 4.79% Coverage

If a patient calls in complaining of a UTI to your clinic, who takes the call and how does it get triaged?

010: It goes from phone people, to medical secretary of provider, then forwarded to provider. They find out if the provider wants them to come in, or does the provider want to treat sight unseen. So, I would say 9 times out of 10 I’m gonna say you need to come in so i can get some urine. There’s extenuating circumstances, like if a patient is very immunocompromised and I don’t wanna take a chance, but...so no, that’s how it goes from one to the other.

<Files\\12_20_18_012> - § 1 reference coded [8.09% Coverage]

Reference 1 - 8.09% Coverage

you said you’d get a UA to confirm your diagnosis and send it further to culture right? Does your patient need to come in or can you manage over the phone?

012: I’d like them to come in because I’d like to do the UA. I’d prefer them to come in.

J: Did you ever have patients just call in, and how do you deal with that?

012: I know that like, UTIs are pretty miserable so I’m pretty flexible if they’re on vacation, I don’t always make them come in, or if they can’t get off work. Some of them will call the office, the girls will take on a note on their system, sometimes I’ll call them in an antibiotic if i feel it’s appropriate.

<Files\\8_13_18_001> - § 8 references coded [30.60% Coverage]

Reference 1 - 2.93% Coverage

you think that it's probably an uncomplicated urinary tract infection and you usually like to get testing, are there times were you don't get testing or do you always like to get ?

001—if somebody calls in or sends a message through the portal and they have frequent UTIs or I'm pretty sure they have one and we don't have openings that they can get in then I something send out antibiotics.

References 2-3 - 4.19% Coverage

what info do u usually get? Like a front desk person get the phone call and then does a relay it to you ..?

001—The access center gets the phone call and hopefully gets adequate information sometimes they do sometimes not, then either I have to call him back or my secretary calls them back and finds out do they have a fever, back pain, blood in urine?, symptoms, allergies, medications they are on you know, how long its been going on, trying to find out a better history than just the message that we sometimes get that is patient has UTI wants antibiotics.

References 4-6 - 7.61% Coverage

staff gets trained what to ask or they just ask questions..

001—they supposedly should, they are all in the access center now and there's a wide variety and they're supposed to use a script but usually I'll get you know they'll have all the little lines of what they're supposed to ask and it'll say n/a, n/a, n/a.

M--and then they can also message you directly ?

001—the patients?? Yes

M—Do u get messages for UTI?

001-- Not a ton but yes I do get them and the people say I think I have another UTI ,pain, go every 5 minutes, usually the information I get through that through the portal is probably a lot more useful than the messages I get from the patients. Its more detailed…I woke up this morning and I think I have the chills or whatever.

M—that’s interesting. The call center staff trained??

001--I know they're trained I mean cuz I helped develop some of the scripts for what they're supposed to ask I think they're just sometimes they don't do it, they're busy ,they're lazy ,they don't care whatever.

Reference 7 - 8.34% Coverage

You have treatment pathways and nursing algorithms too to help out with reporting symptoms, do you think the nurses or like help desk staff or other people that are answering the phones, do you think that they are adequately trained or do a good job at evaluating this if there was a simple next step and beyond just reporting symptoms they would follow an algorithm where they say you got two out of three symptoms or you had one of this but you had back pain we need to call the doctor instead?

001-It would be better if there was a more detailed algorithm in or more easily set out because now there's not an algorithm for them to treat or call the provider , it's just these are the questions they ask and sometimes filling it out and sometimes they don’t. when we had a different exchange we had a nurse exchange on the weekend they follow an algorithm and they were allowed to send out antibiotics for UTIs and a couple other things and that was pretty good but I think the access center now not medically trained at all and like some people are not even medical people they just answer the phone so its difficult.

Reference 8 - 7.53% Coverage

Previously they had standing orders for it they could actually if they could fit in the algorithm, you would say send them out with three days of Cipro it'll be at your Pharmacy and then you were notified I guess in the future about this.

001—back in the day we got faxes on weekends

M—Do u think that worked? Concerns for safety etc..

001--When we had that our exchange they're all RNs and they were really great and there was no concerns with that and like I said there's no capability for that now with the access center , the people aren't even medically trained so it's difficult.

M-- and then I guess if we take it one step further if there was a way to have patients to report symptoms or fill out or follow the algorithms themselves would you be comfortable with something like that?

001—Oh absolutely, people could go in the portal and do that I don't that would be even better like I said the stuff I get from patients is much more accurate and better than what we get from access center by far.
